# Au⋅⋅⋅H−C Hydrogen Bonds as Design Principle in Gold(I) Catalysis

**DOI:** 10.1002/anie.202108581

**Published:** 2021-08-18

**Authors:** Heidar Darmandeh, Julian Löffler, Nikolaos V. Tzouras, Busra Dereli, Thorsten Scherpf, Kai‐Stephan Feichtner, Sofie Vanden Broeck, Kristof Van Hecke, Marina Saab, Catherine S. J. Cazin, Luigi Cavallo, Steven P. Nolan, Viktoria H. Gessner

**Affiliations:** ^1^ Chair of Inorganic Chemistry II Faculty of Chemistry and Biochemistry Ruhr-University Bochum Universitätsstraße 150 44801 Bochum Germany; ^2^ Department of Chemistry and Centre for Sustainable Chemistry Ghent University Krijgslaan 281, S-3 9000 Ghent Belgium; ^3^ Physical Sciences & Engineering Division (PSE) KAUST Catalysis Center (KCC) King Abdullah University of Science and Technology (KAUST) Thuwal 23955-6900 Saudi Arabia

**Keywords:** catalysis, gold, phosphines, secondary interactions, steric and electronic properties

## Abstract

Secondary ligand–metal interactions are decisive in many catalytic transformations. While arene–gold interactions have repeatedly been reported as critical structural feature in many high‐performance gold catalysts, we herein report that these interactions can also be replaced by Au⋅⋅⋅H−C hydrogen bonds without suffering any reduction in catalytic performance. Systematic experimental and computational studies on a series of ylide‐substituted phosphines featuring either a PPh_3_ (^Ph^YPhos) or PCy_3_ (^Cy^YPhos) moiety showed that the arene‐gold interaction in the aryl‐substituted compounds is efficiently compensated by the formation of Au⋅⋅⋅H−C hydrogen bonds. The strongest interaction is found with the C−H moiety next to the onium center, which due to the polarization results in remarkably strong interactions with the shortest Au⋅⋅⋅H−C hydrogen bonds reported to date. Calorimetric studies on the formation of the gold complexes further confirmed that the ^Ph^YPhos and ^Cy^YPhos ligands form similarly stable complexes. Consequently, both ligands showed the same catalytic performance in the hydroamination, hydrophenoxylation and hydrocarboxylation of alkynes, thus demonstrating that Au⋅⋅⋅H−C hydrogen bonds are equally suited for the generation of highly effective gold catalysts than gold‐arene interactions. The generality of this observation was confirmed by a comparative study between a biaryl phosphine ligand and its cyclohexyl‐substituted derivative, which again showed identical catalytic performance. These observations clearly support Au⋅⋅⋅H−C hydrogen bonds as fundamental secondary interactions in gold catalysts, thus further increasing the number of design elements that can be used for future catalyst construction.

## Graphical abstract

Experimental and computational studies on PPh_3_ and PCy_3_‐substituted ylide‐functionalized phosphines as well as on a biaryl and a cyclohexyl‐aryl phosphine demonstrate that Au⋅⋅⋅H−C hydrogen bonds can serve as secondary metal ligand interactions similar to gold–arene interactions often used in ligand design for the stabilization of catalytically active species. Remarkably short Au−H bonds are observed.

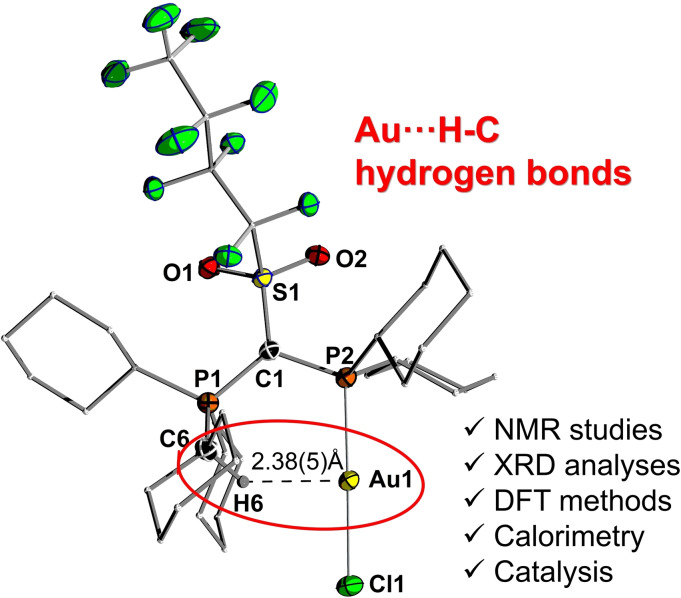

## Introduction

**Figure 1:**
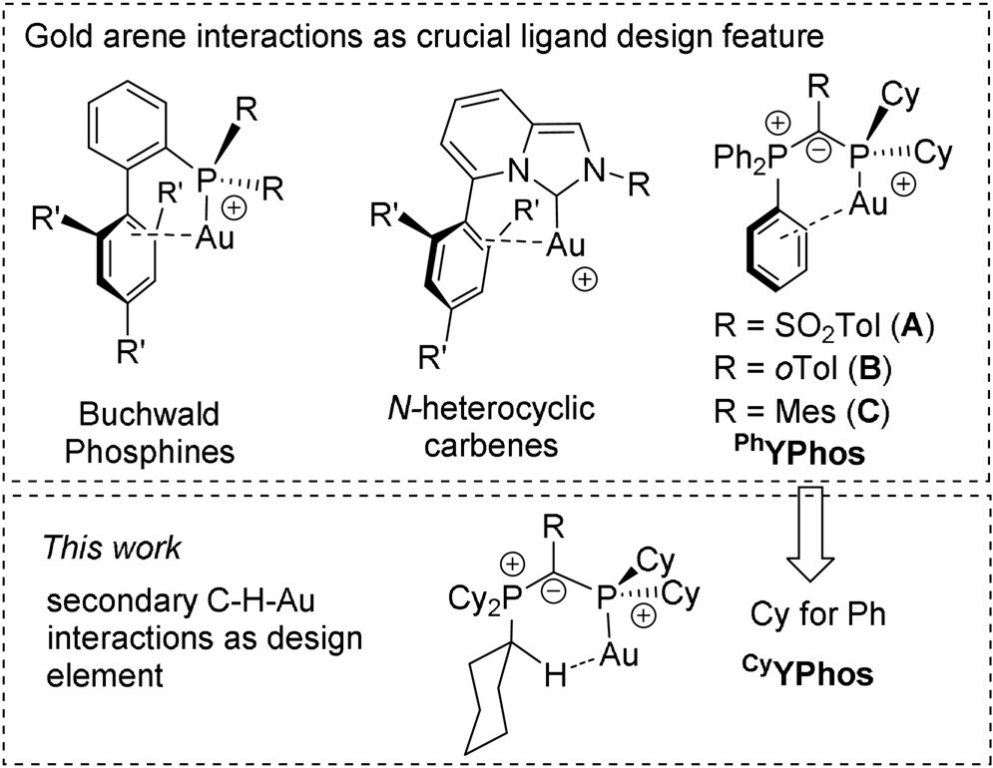
Recent examples of phosphine and carbene gold(I) complexes with postulated gold–arene interactions (top) and ^Ph^YPhos ligands with gold–arene interaction vs. Au⋅⋅⋅H−C(sp^3^) interaction and their novel ^Cy^YPhos analogues described in this work. (R=alkyl or aryl).

Gold catalysis has undergone a rapid development in the past two decades.^[1]^ As is the case for numerous other metal‐catalysed transformations, this success story is oftentimes associated with the development of ligands and the tailoring of their properties to meet the specific requirements of the metal and targeted reaction. In gold(I) catalysis, a cationic LAu^+^ complex is usually the catalytically active species.^[2]^ In order to stabilize these species and to generate highly active and efficient catalysts, secondary ligand metal interactions have been reported as being important in ligand design and have fuelled numerous advances in the field of homogenous catalysis. Particularly gold‐arene interactions have repeatedly been described to being beneficial in gold(I) catalysis.^[3]^ This was, for example, illustrated in the Buchwald biarylphosphine ligands (Figure 1), in which the lateral arene ring is involved in bonding to the metal center.^[4]^ This design principle was adopted in several other phosphines and *N*‐heterocyclic carbenes,^[5]^ such as in the imidazo[1,5‐a]pyridin‐3‐ylidene platform first described by Lassaletta and Glorius^[6]^ or in Alcarazo's *N*‐arylpiperidinophosphines.^[7]^

Recently, we reported on transition metal catalysts based on ylide‐functionalized phosphines (YPhos).^[8]^ In gold(I)‐catalyzed transformations with moderately (**A**)^[8a]^ as well as highly electron‐rich (**B** and **C**)^[9]^ YPhos systems exceptionally high turnover numbers were observed. All of these YPhos catalysts so far have relied on triphenyl phosphonium groups which likewise foster arene–gold interactions, thus contributing to the stability and high catalytic performance of the corresponding LAu(I)^+^ species.

The understanding of such secondary ligand metal interactions is important for future ligand design and for the development of high‐performance catalysts. Thus, we wondered if these arene‐gold interactions were indeed necessary or if equally high activities can also be achieved without such stabilizing interactions. If arene‐gold interactions could be omitted or replaced by other interactions, this would lead to a paradigm shift in ligand design and would significantly broaden the structural scope and thus facilitate the synthesis of efficient ligands in the future. We envisioned that the YPhos ligands would be an ideal ligand platform to systematically address the importance of arene‐gold interactions for catalysis. The PPh_3_ moiety in the YPhos ligands can easily be replaced by a tricyclohexyl phosphonium group (^Cy^YPhos), thus preventing the interaction between the metal and the phenyl group without changing the overall ligand architecture. Herein, we show that indeed the often‐invoked arene‐gold interactions are not necessary, but can be replaced by stabilizing hydrogen bonds, which are equally suited in generating highly active catalysts.

## Results and Discussion

### Ligand Synthesis

To probe the importance of supporting interactions between gold and the phosphonium moiety, we present a detailed study of the performance of the three PCy_3_‐substituted ligands ^Cy^Y_S_PCy_2_ (**L1**), ^Cy^Y_*o*Tol_PCy_2_ (**L2**) and ^Cy^Y_Mes_PCy_2_ (**L3**) as congeners to **A**, **B** and **C**. Ligands **L2** and **L3** have previously been designed for the selective Pd‐catalysed monoarylation of small primary amines.^[10]^ Additionally, the *iso*‐propyl‐derivative of **L1**, ^Cy^Y_S_P*i*Pr_2_ (**L4**) was synthesized to evaluate the influence of the lower steric bulk of the smaller alkyl group on the catalytic ability. **L1** and **L4** were prepared on gram scale as white solids by reaction of the metalated ylide ^Cy^Y_S_‐Li with Cy_2_PCl and *i*Pr_2_PCl in good yields of 75 % and 70 %, respectively.^[11]^

**Scheme 1:**
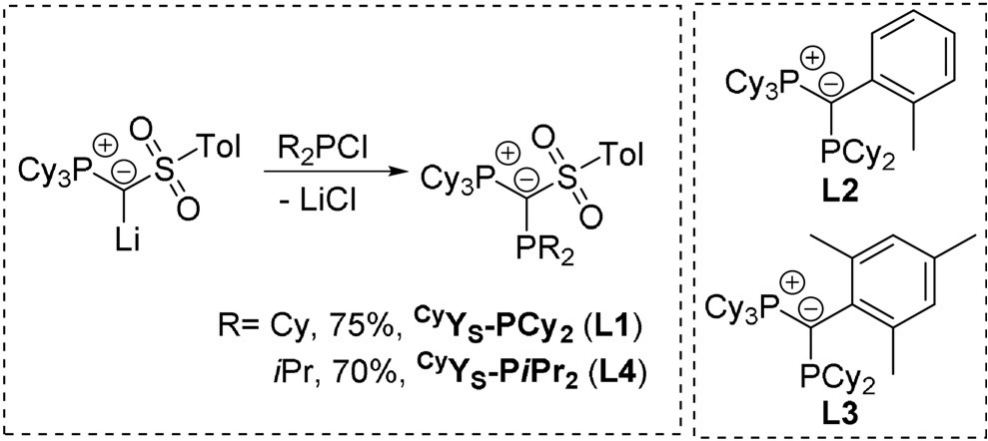
Synthesis of ^Cy^Y_S_‐PR_2_ by reaction of the metalated ylide ^Cy^Y_S_‐Li with dialkyl chlorophosphines (PR_2_Cl with R=*i*Pr or Cy).

**Scheme 2:**
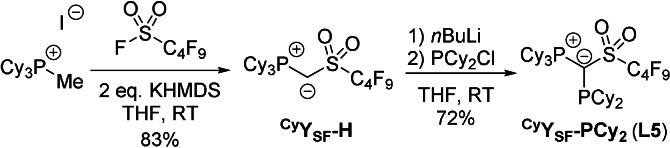
Synthesis of the ylide precursor ^Cy^Y_SF_‐H from perfluorobutyl sulfonyl fluoride, KHMDS and the phosphonium salt [Cy_3_P‐Me]I and subsequent reaction with *n*BuLi and PCy_2_Cl to yield ^Cy^Y_SF_‐PCy_2_ (**L5**).

**L1** features two sets of doublets in the ^31^P{^1^H}‐NMR spectrum at 31.7 and −7.3 ppm, with a coupling constant of ^2^ *J*_PP_=106.8 Hz, while **L4** displays resonances at 31.5 and 1.5 ppm with a slightly smaller coupling constant of ^2^ *J*_PP_=105.7 Hz. In addition to the obtained new ligands, **L1** and **L4**, we targeted a completely arene‐free ^Cy^YPhos derivative, to eliminate any possible metal‐arene interaction. Therefore, we tackled the synthesis of ^Cy^Y_SF_‐PCy_2_ (**L5**), an analogue of **1** in which the *p*‐tolyl motif is replaced by a perfluorobutyl chain. To access this ligand, the metalated ylide ^Cy^Y_SF_‐Li first had to be synthesized. By analogy to the procedure reported for ^Cy^Y_S_‐Li,^[11]^ the protonated precursor ^Cy^Y_SF_‐H was prepared in a one‐pot reaction from the easily accessible phosphonium salt [Cy_3_P‐CH_3_]I and the commercially available perfluorobutanesulfonyl fluoride in the presence of two equiv of KHMDS in a very good yield of 83 % (Scheme 2). ^Cy^Y_SF_‐H was isolated as a pale‐yellow powder and fully characterized (see SI for details).

Deprotonation of ^Cy^Y_SF_‐H with *n*‐butyllithium afforded the metalated ylide ^Cy^Y_SF_‐Li which was used in situ and directly reacted with PCy_2_Cl to yield ^Cy^Y_SF_‐PCy_2_ (**L5**) as a colorless solid in 72 % yield. With *δ*_P_=32.3 and −0.7 ppm, its ^31^P{^1^H} NMR signals are in the same range as observed for **L1** and **L4**, while with ^2^ *J*_PP_=95.0 Hz, the coupling constant is significantly smaller. Important NMR spectroscopic and crystal structure parameters are given in Table 1. XRD analyses confirm the expected connectivity and show the typical arrangement of PCy_3_ substituted YPhos ligands, were the alkyl groups attached to the phosphorus atom point away from the phosphonium group to minimize the steric pressure.^[12]^

**Figure 2:**
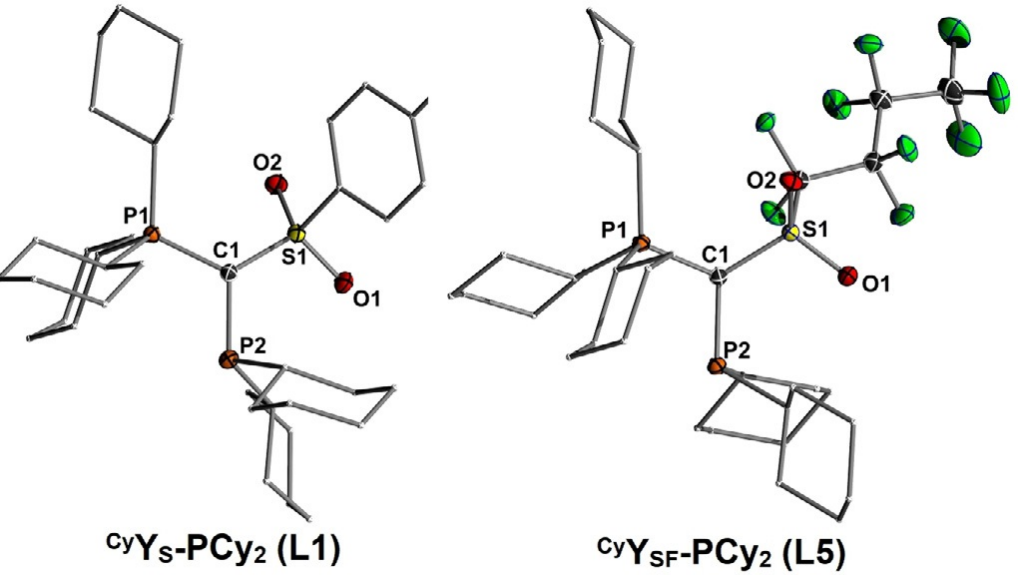
Molecular structures of ^Cy^Y_S_‐PCy_2_ (**L1**) (left) and ^Cy^Y_SF_‐PCy_2_ (**L5**) (right). Thermal displacement ellipsoids of selected atoms drawn at 50 % probability level; hydrogen atoms are omitted for clarity.

**Table 1:** ^31^P{^1^H} NMR spectroscopic data and selected crystal structure details for **L1**, **L4** and **L5**.

	^Cy^Y_S_‐PCy_2_ (**L1**)	^Cy^Y_S_‐PiPr_2_ (**L4**)	^Cy^Y_SF_‐PCy_2_ (**L5**)
*δ*_P_ (PCy_3_) [ppm]	31.7	31.5	32.3
*δ*_P_ (PR_2_) [ppm]	−7.3	1.5	−0.7
^2^ *J*_PP_ [Hz]	106.8	105.7	95.0
P1−C1 [Å]	1.7570(15)	1.7614(14)	1.7688(11)
P1‐C1‐P2 [°]	114.66(8)	115.83(7)	113.97(6)

To evaluate the ligand electronic properties, we calculated the Tolman electronic parameter (TEP) by analyzing the carbonyl stretching frequencies of the corresponding [Rh(^Cy^YPhos)(acac)(CO)] complexes (Table 2). As expected, ^Cy^Y_S_‐PCy_2_ (**L1**), with a TEP_calc_ of 2057.0 cm^−1^, is more electron‐releasing than its isopropyl‐substituted congener **L4** (TEP_calc_ 2058.7 cm^−1^). Furthermore, the stabilizing effect of the sulfonyl moiety becomes evident, by comparison with aryl substituted YPhos ligands **L2** and **L3**, which are significantly more electron‐donating than **L1** and **L4**. Interestingly, ^Cy^Y_SF_‐PCy_2_ (**L5**) with a TEP_calc_ value of 2059.8 cm^−1^ is even less electron‐releasing than the simple alkyl phosphine PCy_3_ (TEP_calc_ 2058.1 cm^−1^). This result clearly demonstrates the further increased electron‐withdrawing nature of the perfluorobutylsulfonyl moiety and thus shows how easily the electronics of YPhos ligands can be tuned via backbone modification.

**Table 2:** Comparison of the TEP values for different alkylphosphines and YPhos ligands.

Ligand	*ν*_Co_ Rh [cm^−1^]	TEP_calc._ [cm^−1^]^[a]^
PPh_3_^[a]^	1978.0	2069.1
PCy_3_^[a]^	1958.7	2058.1
^Ph^Y_S_PCy_2_ (**A**)^[b]^	1953.5	2055.1
^Ph^Y_*o*Tol_PCy_2_ (**B**)^[c]^	1947.5	2051.7
^Cy^Y_*o*Tol_PCy_2_ (**L2**)^[d]^	1941.1	2048.0
^Cy^Y_Mes_PCy_2_ (**L3**)^[d]^	1941.8	2048.4
^Cy^Y_S_PCy_2_ (**L1**)	1956.8	2057.0
^Cy^Y_S_P*i*Pr_2_ (**L4**)	1959.7	2058.7
^Cy^Y_SF_PCy_2_ (**L5**)	1961.7	2059.8

[a] TEPs were determined by *ν*_CO_ in the [Rh(acac)(CO)(L)] complexes using the linear relationship between ν_CO_ for [Ni(CO)_3_(L)] and [Rh(acac)(CO)(L)] reported in ref. [13]. [b] Values taken from reference [8a]. [c] Values taken from reference [9a]. [d] Values taken form reference [10].

### Synthesis and Structures of the Gold Complexes

**Scheme 3:**
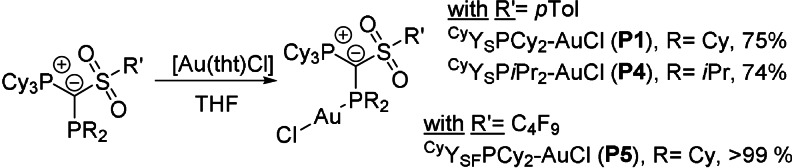
Preparation of [Au(^Cy^Y_S_PR_2_)Cl] complexes (with R=*i*Pr and Cy) from the reaction of **L1**, **L4** and **L5** with [Au(tht)Cl] in THF.

With the novel ligands in hand, we next prepared [Au(^Cy^YPhos)Cl] complexes from the free ligands and [Au(tht)Cl] (Scheme 3). [Au(^Cy^Y_S_PCy_2_)Cl] (**P1**), [Au(^Cy^Y_S_P*i*Pr_2_)Cl] (**P4**) and [Au(^Cy^Y_SF_‐PCy_2_)Cl] (**P5**) were isolated as colorless solids in good to quantitative yield. As expected, the ^31^P{^1^H} NMR resonances are shifted significantly downfield upon complexation together with a decrease of the coupling constant, for example, from 31.7 and −7.3 ppm (^2^ *J*_PP_=106.8 Hz) in **L1** to 40.7 and 29.9 ppm (^2^ *J*_PP_=35.6 Hz) for **P1**. Interestingly, in contrast to the phosphine precursors, the protons of the tertiary carbon atoms in the phosphonium group appeared as a slightly broadened signal in the ^1^H‐NMR spectrum of complexes **P1** and **P4**. The corresponding carbon atom shows a similar behavior in the ^13^C{^1^H} NMR spectrum and appears as a broad doublet at around 36.9 ppm. This broadening of the PC*H* protons of the PCy_3_ group is even more pronounced for complex **P5** and could be caused by an attractive Au⋅⋅⋅H‐C(sp^3^) interaction. Indeed, VT‐NMR studies of a solution of **P5** in DCM‐d^2^ showed a splitting of the signal with one proton signal being downfield shifted from 3.21 ppm to 4.53 ppm at −80 °C (see Figure S27 and S28). The two signals integrate in a 2:1 ratio, suggesting that the downfield shifted signal corresponds to one proton interacting with the metal center. DFT calculations (see SI for details) confirm a downfield shift of the PC*H* proton interacting with gold by 1.24 ppm (5.18 ppm versus 3.89 and 3.99 ppm for the PC*H* signals), which matches well with the experimental observations. Such a downfield shift has also recently been reported for other Au⋅⋅⋅H−C interactions, but has controversially been discussed.^[14]^ In the case of complex **P1**, only a broadening and no splitting of the PC*H* signal was observed thus suggesting a weaker gold hydrogen interaction.

The existence of Au⋅⋅⋅H−X hydrogen bonds has controversially been discussed in the literature,[^15^, ^16^] but were experimentally and computationally proven in recent studies.^[17]^ For example, the groups of Bourissou,^[18]^ Berger and Monkowius^[19]^ as well as Ruližek^[20]^ demonstrated that N^+^‐H ammonium or pyridinium groups are suitable donors for the formation of N−H⋅⋅⋅Au hydrogen bonds. Other strong donors such as O−H, F−H, NH_3_ and HCN were also found to form close‐contact interactions with gold.^[21]^ Au⋅⋅⋅H−C interactions have very recently been reported, but have never been discussed as structural motif for ligand design in catalysis.^[22]^ Despite numerous early discussions, it is now well established that the Au atom in these Au⋅⋅⋅H−X interactions acts as an electron donor and the X−H moiety as an acceptor, which is in contrast to “classical” agostic interactions which rely on the donation of electron density into an empty orbital at the metal center.^[23]^ Accordingly, these interactions were named anagostic interactions or “contra‐electrostatic” hydrogen bonds.^[24]^ The later term emphasises the relation to classical H‐bonds, which however differ in the charge of the C and H atom upon approximation of the donor (gold versus the H‐bond acceptor). Very recent computational studies by Alabugin, Sollogoub and co‐workers demonstrated that these attractive interactions are also present in methane complexes with NHC‐Au‐Cl, thus emphasizing that Au⋅⋅⋅H−C hydrogen bonds are not exclusively the result of steric congestion.^[24a]^

**Figure 3:**
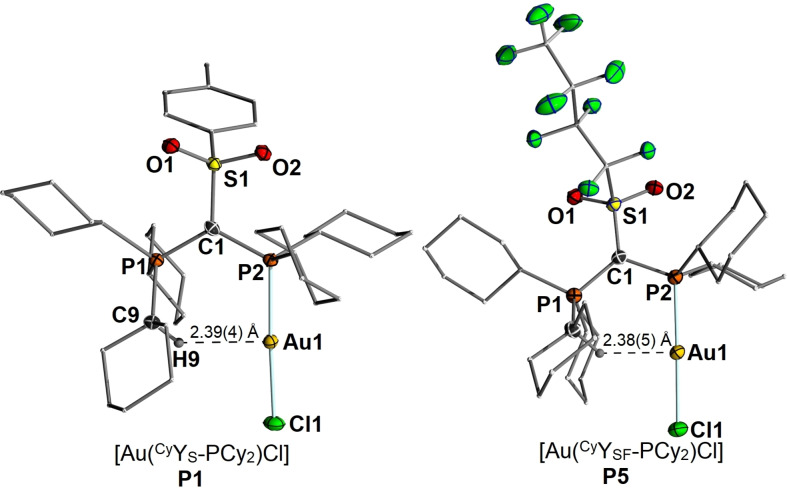
Molecular structures of [Au(^Cy^Y_S_‐PCy_2_)Cl] (**P1**) (left) and [Au(^Cy^Y_SF_‐PCy_2_)Cl] (**P5**) (right). Thermal displacement ellipsoids of selected atoms drawn at 50 % probability level; hydrogen atoms omitted for clarity.

Further proof of the Au⋅⋅⋅H−C interactions in the YPhos‐gold complexes is manifested in their molecular structures (Figure 3). Indeed, with 2.39(4) Å and 2.38(5) Å in **P1** and **P5** respectively, the Au⋅⋅⋅H−C distances are remarkably short and significantly shorter than their sum of Van der Waals radii (2.86 Å)^[25]^ as well as hydrogen bonds previously reported.^[22]^ For example, Koshini and co‐workers reported on Au−H distances between 2.60 and 2.65 Å in gold clusters supported by a phenylene‐bridged diphosphine ligand.^[16a]^ To the best of our knowledge the Au‐H9 distance of 2.38(5) Å in **P5** is the shortest distance reported to date for a Au⋅⋅⋅H−C hydrogen bond.^[26]^ Presumably, this interaction is stronger than those found with other C−H moieties due to the stronger polarization of this entity next the positively charged phosphorus centre.

Of course, one could argue that the interaction might also be caused by steric pressure within the molecule. However, the latter should be less critical in the structure of the complex with *i*Pr‐substituted phosphine **L4**. Here, the smaller alkyl groups should give rise to a more flexible ylide structure and allow a widening of the P‐C‐P angle, which was found to be decisive for the approach of the phosphonium group to the metal center and hence for secondary metal ligand interactions.^[27]^ Unfortunately, no crystals of **P4** of sufficient quality could be obtained to allow for the direct location of the hydrogen atom in the electron density map. However, the observed Au−C distance clearly indicates that also short interactions between gold and the PCy_3_ unit are present in **P4** (Table 3). It is interesting to note, that **P2** and **P3** with aryl groups in the ylide‐backbone showed considerably longer C−H⋅⋅⋅Au distances. Nonetheless, they are shorter than the sum of Van‐der Waals radii, thus suggesting that weak secondary ligand metal interactions are still present in these complexes. These differences in the C−H⋅⋅⋅Au distances can be explained by the different bulk of the sulfonyl and the aryl groups. Whereas the flexible sulfonyl group allows the complexes **P1**, **P4** and **P5** to adopt the preferred geometry with a planar Au‐P‐C‐P unit,^[28]^ the rigid tolyl and mesityl substituents enforce a deformation, which results in the rotation of the PCy_2_ moiety and hence the coordination of AuCl “outside” the center of the pocket formed by the PCy_3_ and PCy_2_ units (see Figure S36). This also results in a slightly reduced steric pressure of the aryl‐substituted ligands directed towards the metal center as measured by the percent buried volume (*V*_bur_%) (Table 3).^[29]^ While all ligands are highly sterically demanding, covering more than half of the sphere defined in the model, it is slightly lower for the YPhos ligands **L2** and **L3** with an aryl group in the ylidic backbone. With 54.8 *V*_bur_% **L1** is slightly bulkier than **L4** (53.2 *V*_bur_%) and **L5** (52.5 *V*_bur_%), but similar demanding than the PPh_3_ substituted analogue **A**. This comparison between **L2** and **L3** and the sulfonyl‐substituted ligands shows that the hydrogen bond is easily affected by steric effects.

**Table 3:** ^31^P{^1^H} NMR data, *V*_bur_% and important crystal structure bond lengths and angles for the novel ^Cy^YPhos complexes.

	**P1**	**P2** ^[a]^	**P3** ^[a]^	**P4** ^[b]^	**P5**
*V*_bur_%	54.8	49.4	50.7	53.2	52.5
C_PCy3_‐H‐Au [Å]	2.39(4)	2.70(3)	2.73(3)	n.d.	2.38(5)
C_PCy3_‐Au [Å]	3.264(3)	3.49(3)	3.407(2)	3.312(15)	3.301(4)
C_PCy3_‐H‐Au [°]	141.1(1)	136.4(1)	128.9(1)	n.d.	138.6(1)
P1‐C1‐P2	122.7(2)	121.2(1)	123.7(1)	124.8(7)	119.6(2)
P2‐Au‐Cl [°]	177.2(1)	177.9(1)	179.5(1)	178.4(1)	178.6(1)

[a] Values taken from reference [10]. [b] crystallographic values taken from one molecule of the asymmetric unit; data was not of sufficient quality for reliable determination of CH‐Au interaction. [c] Calculated with the SambVca 2.1 program for the [Au(L)Cl complexes; M‐P distance=2.28 Å, including H atoms.

### Calorimetric Studies

**Scheme 4:**
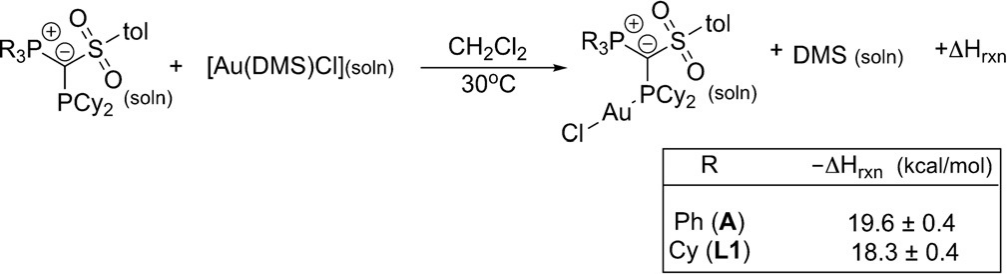
Enthalpy of ligand substitution (kcal mol^−1^) associated with the formation of [Au(^R^Y_S_PCy_2_)Cl] (R=Ph (**A**) and Cy (**L1**)) complexes.

In order to gain further insights into the relative stability of cyclohexyl‐ vs. phenyl‐substituted YPhos ligands bound to gold, we initiated a solution calorimetry study focusing on ligand substitution of the labile dimetylsulfide (DMS) ligand in the common gold synthon, [Au(DMS)Cl]. Results are presented in Scheme 4.

The batch solution calorimetry results clearly show the very similar enthalpy of reaction values (−19.6±0.4 (for **A**) and −18.3±0.4 (for **L1**) kcal mol^−1^) obtained for this simple ligand substitution. These data clearly indicate, that within 1 kcal mol^−1^, both ligands possess similar binding energies to the metal center. The enthalpy values, we will emphasize, reflect all interactions with the metal (σ,π and secondary interactions). The TEP value for **A**, 2055.1 cm^−1^, (vs. 2057.0 cm^−1^ for **L1**) indicates that **A** is a better donor ligand just on an electronic basis. The infrared data suggest that more negative enthalpies of ligand substitution should be expected for the reaction in Scheme 4 involving **A**, and experimental results validate this expectation.

### Catalytic Performance

Next, we turned our attention to the catalytic activity of the [Au(^Cy^YPhos)Cl] complexes. We selected the hydroamination of phenylacetylene with aniline as first test reaction since this would permit a comparison with the previously reported PPh_3_‐substituted YPhos ligands. Furthermore, the effectiveness of gold catalysts in this reaction is severely influenced by the stability of the cationic gold species towards reduction to gold(0), which in turn is affected by secondary metal–ligand interactions, thus making it an ideal test reaction.^[30]^ To this end, the [Au(YPhos)Cl] complexes of **A**, **B** and **C** as well as their direct PCy_3_ analogues [Au(^Cy^Y_S_PCy_2_)Cl] (**P1**), [Au(^Cy^Y_*o*Tol_PCy_2_)Cl] (**P2**) and [Au(^Cy^Y_Mes_PCy_2_)Cl] (**P3**) were compared under identical reaction conditions (0.1 mol % of respective ligand and NaBAr^F^, neat, 50 °C). Additionally, we also tested **P4** to investigate the effect of the size of the alkyl groups attached to the phosphorus (III) atom on the catalytic activity. Furthermore, we also examined complex [Au(^Cy^Y_SF_PCy_2_)Cl] (**P5**) with the perfluorinated “aryl‐free” ligand **L5**, which excludes the presence of any arene‐gold interactions and hence gives direct information about the importance of these secondary interactions in the YPhos ligands.

**Figure 4:**
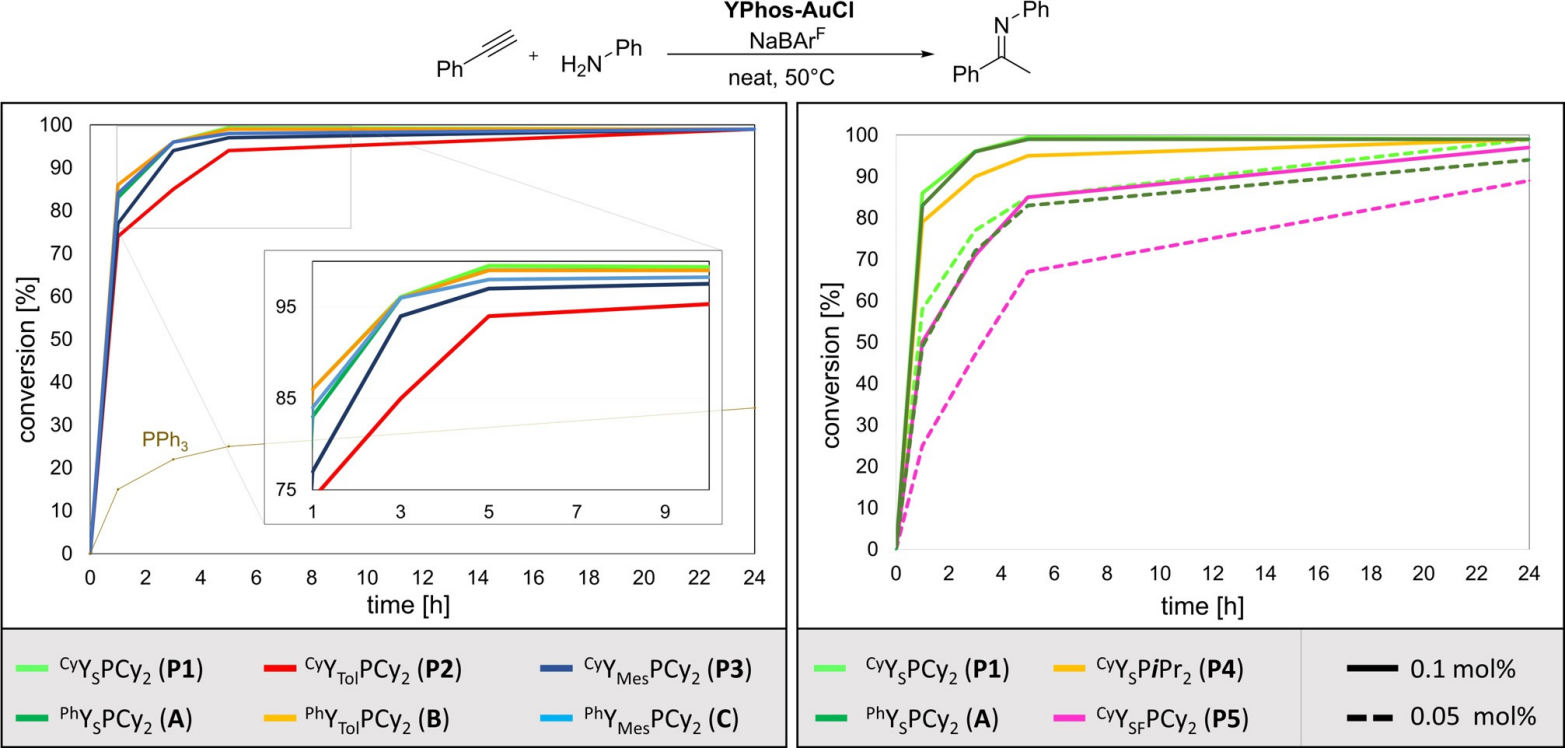
Conversion‐time plots of the catalysis results of the YPhos‐AuCl catalyzed hydroamination of phenyacetylene with aniline. Reaction conditions: 0.1 mol % YPhos ligand and 0.1 mol % NaBAr^F^, neat, 50 °C.

Figure 4 shows the conversion time plots of the comparison between the ^Ph^YPhos and ^Cy^YPhos ligands. Note that without the addition of NaBAr^F^ no conversion was observed. The same holds true for using NaBAr^F^ without addition of any gold complex. To our delight, the cyclohexyl‐substituted complexes proved to be highly active catalysts (Figure 4, left), which performed considerably better than simple PPh_3_ or PCy_3_ (see the SI for further details). Most importantly, they were equally efficient as their PPh_3_‐substituted analogues, (e.g. **A** and **P1**) or only slightly less active (**B**/**C** vs. **P2**/**P3**). Overall **P1** and **P3** performed superbly, leading to almost full conversion to the imine after approx. 5 h of reaction time. Only the tolyl‐substituted catalyst is slightly less effective. Changing the phosphine alkyl groups from cyclohexyl in **P1** to isopropyl in **P4**, led only to a moderate drop in activity (Figure 4, right). Most interestingly, the completely arene‐free ^Cy^YPhos complex **P5** also gave nearly full conversion after 24 h, thus demonstrating that the absence of arene‐metal interaction also leads to highly active gold catalysts. The slightly lower activity of **P5** compared to its tosylate analogue **P1** can be attributed to the more electron‐withdrawing nature of the C_4_F_9_‐substituents. Most importantly, the PCy_3_‐substituted YPhos ligands also preserved their high activity at lower catalyst loadings of only 0.05 mol % (Figure 4, right, dashed lines). Again, **P1** performed equally well compared to **A**. This is an important finding, since ligand design has so far focussed on the introduction of aryl substituents to incorporate arene gold interactions for stabilizing the catalytically active Au^I+^ species. Our results clearly demonstrate that the active species with ^Ph^YPhos and ^Cy^YPhos are equally active thus suggesting that Au⋅⋅⋅H−C interactions are equally suited for the generation of highly active gold catalysts, which even operate at very low catalyst loadings. This observation points to new possibilities for ligand design in gold catalysis.

**Scheme 5:**
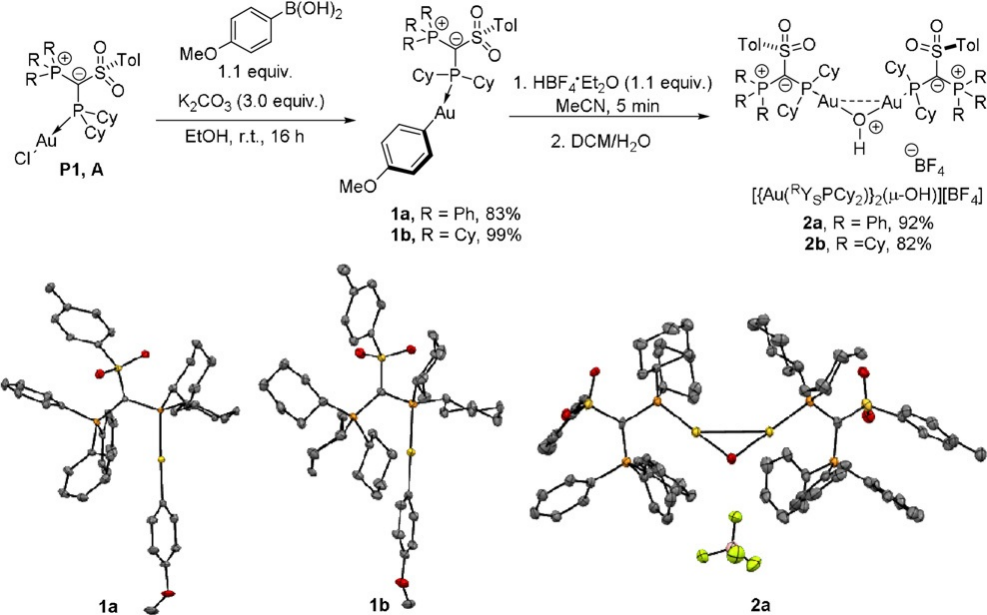
Preparation of well‐defined, cationic [{Au(^R^Y_S_PCy_2_)}_2_(μ‐OH)][BF_4_] complexes (with R=Ph and Cy). Molecular structures of complexes **1 a**, **1 b** and **2 a** are presented, showing thermal displacement ellipsoids at the 50 % probability level and hydrogen atoms omitted for clarity.

To further prove the comparable performance of ^Ph^YPhos and ^Cy^YPhos we synthesized well‐defined, cationic digold complexes bearing these ligands and examined their activity in the hydrophenoxylation and in the hydrocarboxylation of diphenylacetylene. In order to gain access to digold hydroxides, we chose to follow a new route which utilizes Au‐aryl complexes as precursors to cationic complexes (Scheme 5).^[31]^ Therefore, after synthesizing the corresponding Au‐aryl complexes **1 a** and **1 b**, addition of acid to their acetonitrile suspensions, followed by evaporation of the solvent and extractions with DCM/H_2_O led to the desired digold hydroxide complexes **2 a** and **2 b** bearing the two YPhos ligands.[^32^, ^33^] The complexes **1 a**, **1 b** and **2 b** were also characterized by XRD analysis. Most interestingly, **1 b** also exhibited short Au−H interactions (<2.5 Å),^[34]^ thus demonstrating that hydrogen bonds are not limited to the AuCl complexes.

**Scheme 6:**
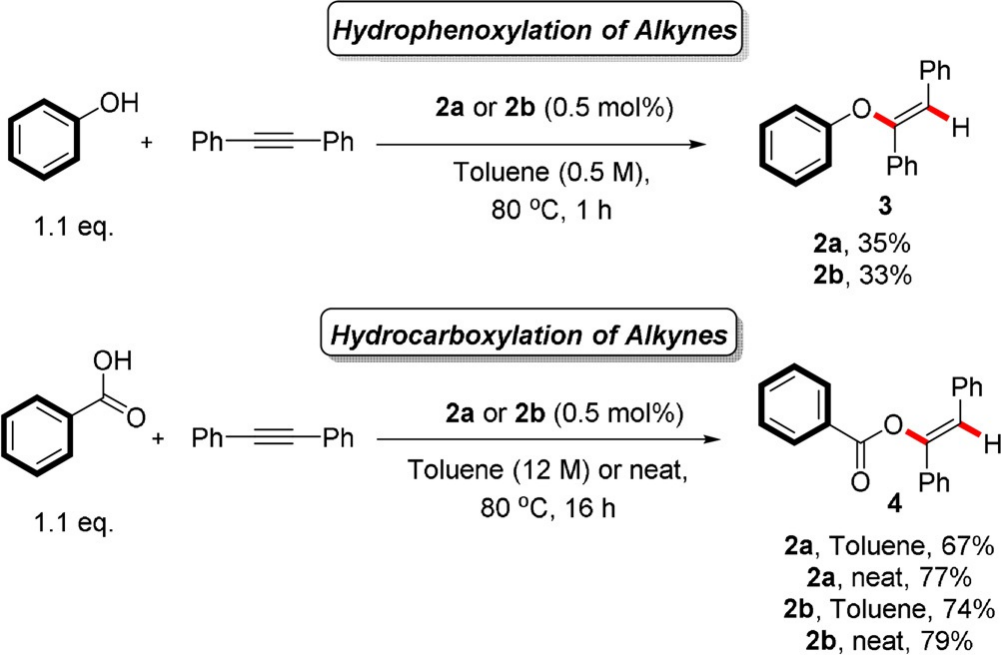
Evaluation of well‐defined, cationic [{Au(^R^Y_S_PCy_2_)}_2_(μ‐OH)][BF_4_] complexes (**2 a** with R=Ph and **2 b** with R=Cy) in the hydrophenoxylation and hydrocarboxylation of diphenylacetylene. Yields were determined by ^1^H‐NMR using 1,3,5‐trimethoxybenzene as the internal standard and were reproduced once.

The complexes were evaluated in terms of their catalytic activity in the hydrophenoxylation and hydrocarboxylation of diphenylacetylene (Scheme 6).[^35^, ^36^] In the hydrophenoxylation to **3**, both complexes displayed lower activity than the state of the art digold complex bearing the IPr ligand (IPr=*N*,*N*′‐bis[2,6‐(di‐isopropyl)phenyl]imidazol‐2‐ylidene).^[31]^ However, their overall performance was essentially the same, irrespective of the structure of the ^R^Y_S_Phos (R=Ph or Cy) ligand. In hydrocarboxylation to **4**, their activity was higher when compared to that in hydrophenoxylation.^[31]^ Again, the same trend is observed with both complexes displaying comparable catalytic activities. It seems that the bulky, electron‐donating YPhos ligands can participate in dual gold catalysis as well, albeit leading to decreased activity in comparison with IPr. This is still significant, considering that both the ligand and the counterion (and their specific combination) are targets for optimization as they markedly affect the outcomes of these reactions. Of note, activation of the corresponding AuCl complexes with NaBAr^F^ did not lead to product formation in either reaction shown in Scheme 6, under various conditions (inert atmosphere, under air, premixing of the gold complex and the chloride abstractor). Synthesis and evaluation of other well‐defined, cationic complexes of this kind will be pursued further.

### Computational Studies

To further evaluate the stability and the nature of the secondary interactions between gold and the different ^Cy^YPhos ligands we performed computational studies on the PW6B95D3/def2tzvp (MWB60 for Au) level of theory. We were particularly interested in answering the following questions: i) Are C−H⋅⋅Au interactions the most favored interactions or can the aryl or the sulfonyl groups in the ylide backbone of the ^Cy^Y_S_‐substituted YPhos ligands also bind and thus stabilize the metal center? ii) Are the short contacts between the CH protons of the PCy_3_ moiety and the gold atom in **P1** and **P5** observed by XRD analysis present in the gas phase and solution structures and iii) what is the nature of these C(sp^3^)‐H⋅⋅⋅Au interactions?

**Geometry optimization, conformers**. To confirm that the conformer of **P1** observed in the crystal structure (**C1**) is also the preferred conformation in the cationic gold complexes a series of structures were optimised (Table 4). Local energy minima were found for the conformers **C2**, in which the ylidic substituent is rotated by ≈160° about the P2−C1 bond and the gold atom is coordinated by the sulfonyl group, and **C3**, in which the ylidic substituent is rotated by ≈180° and an arene–gold interaction can be observed. Energy optimization showed that conformer **C1** with the experimentally observed C(sp^3^)−H⋅⋅⋅Au interaction is thermodynamically preferred over the structures exhibiting an S=O⋅⋅Au (ΔΔ*G*=72 kJ mol^−1^) or arene‐Au interaction (47.6 kJ mol^−1^). This preference is even more pronounced in **P5** with the perfluorobutyl group. It is noteworthy that also for **P3** with the mesityl substituent in the ylide backbone the conformer with the PCy_3_ moiety oriented towards gold is preferred over a **C3** analogue with a mesityl‐Au interaction (ΔΔ*G*=65 kJ mol^−1^, see SI). Hence, the calculations clearly confirm the favourable C−H⋅⋅⋅Au interaction.

**Table 4:** Energies [kJ mol^−1^] of the different possible conformers of **P1** and **P5** and their cations with and without additional coordination of phenylacetylene (**pa**) relative to the energy of conformer **C1**.

Complex	ΔΔ*G* [kJ mol^−1^]	ΔΔ*G* [kJ mol^−1^]	ΔΔ*G* [kJ mol^−1^]
	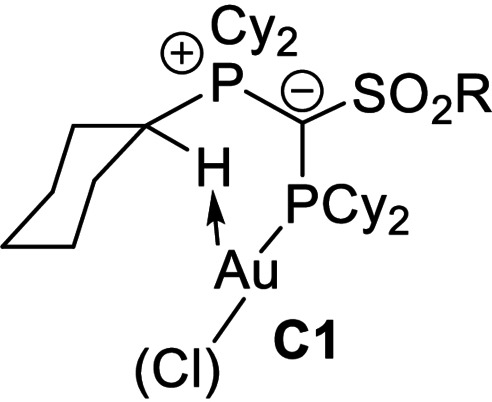	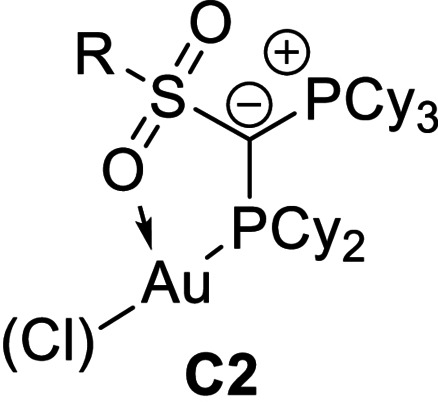	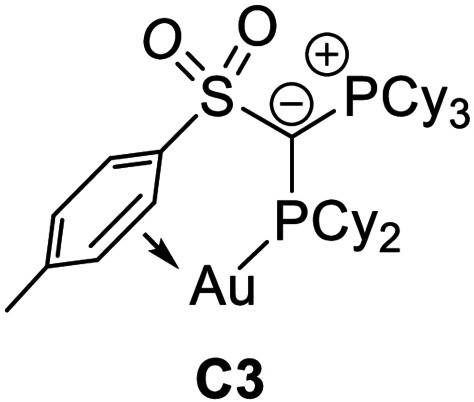
**P1** (R=*p*Tol)	±0.0	+72.3	+47.6
			
**L1⋅Au^+^** (R=*p*Tol)	±0.0	+39.6	+3.5
			
**L1⋅Au(pa)^+^** (R=*p*Tol)	±0.0^[a]^	+29.4^[a,b]^	+19.0^[a,c]^
			
**P5** (R=C_4_F_9_)	±0.0	+116^[b]^	+84.1^[c]^
			
**L5‐Au^+^** (R=C_4_F_9_)	±0.0	+61.5^[b]^	+82.9^[c]^
			
**L5‐Au(pa)^+^** (R=C_4_F_9_)	±0.0^[a]^	+68.8^[a,b]^	+105.8^[a,c]^

[a] including solvent effects using a polarizable continuum model with aniline as solvent. [b] 90° rotation around P2‐C1, S=O⋅⋅Au interaction. [c] 90° rotation around P2‐C1. No additional secondary ligand gold interaction.

Interestingly, the preference of the hydrogen bonded conformer **C1** is also observed for the cationic gold complexes, which are more important in the catalysis. This also holds true for a cationic gold complex with additional coordination of phenylacetylene (pa) including aniline as solvent (PCM model), which reflects the reaction conditions during catalysis. As shown in Table 4 the preference of the hydrogen bonded conformer is still significant for these complexes [LAu(pa)]^+^, albeit being slightly less pronounced than in the neutral LAuCl complexes due to the decreased electron density at gold and the resulting weaker electron donation from Au to the C−H bond (vide infra). To obtain an estimate for the strength of the C−H⋅⋅Au interactions we calculated a conformer of **C1** in which the cyclohexyl group is rotated about the P−C bond to prevent any C−H⋅⋅Au interaction. This conformer revealed to be energetically disfavored over **C1** by 65 kJ mol^−1^.^[37]^ It is also noteworthy, that all attempts to replace the C−H⋅⋅Au interaction by explicit coordination of aniline failed, always resulting in the dissociation of the amine during energy optimization. This clearly underpins the favorable hydrogen bonding.

**Figure 5:**
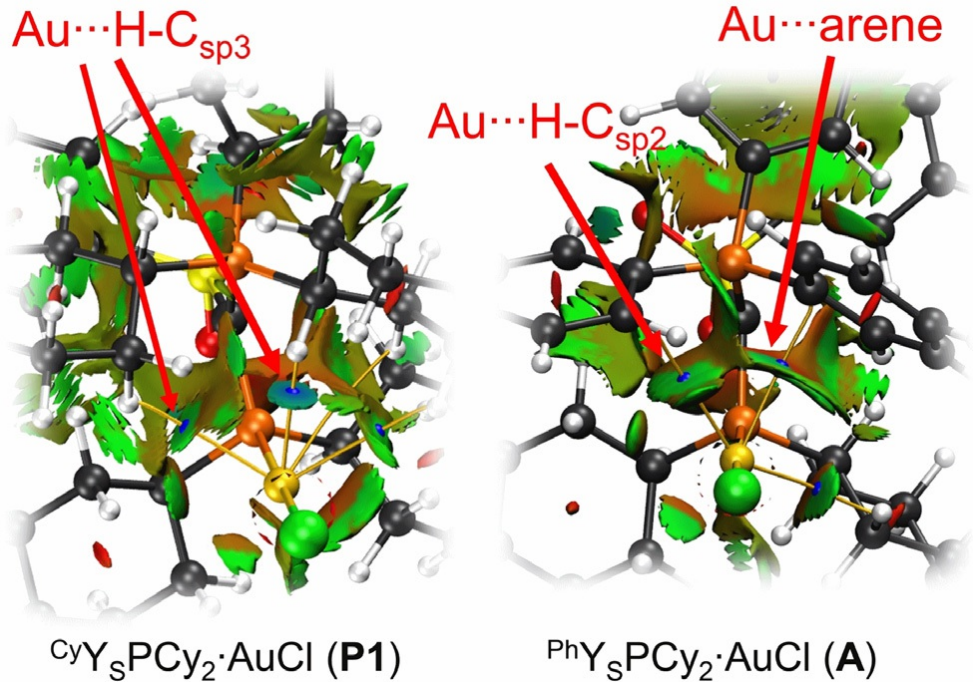
NCI plots for **P1** and **A**. Coloured in a blue‐green‐red scheme over the range of (−0.035 < sign(*λ*_2_)*ρ*<0.02) and isosurface of RDG=0.5. Blue indicates strong attraction, green indicates weak interaction, and red indicates repulsion.

**Bonding analysis**. Having established that the C(sp^3^)−H⋅⋅Au interactions give rise to the thermodynamically most favoured structures, we next turned our attention towards studying the nature of this bonding interaction. To this end, natural bond orbital (NBO), quantum theory of atoms‐in‐molecules (QTAIM) and noncovalent interaction (NCI) analyses on the gold complexes **P1** and **A** were performed (including solvent effects: PCM for aniline). Overall, the computational studies in unison confirm the presence of attractive C−H gold interactions. The QTAIM studies show several bond critical points (BCP) in each of the gold complexes between the Au and the hydrogen atoms of CH and CH_2_ in the PCy_3_ and PCy_2_ moieties. The BCP's with the highest electron density *ρ*(*r*) and Laplacian ∇^2^ *ρ*(*r*) are always found between the Au atom and the PCH group in the PCy_3_ moiety and range between *ρ*(*r*)=0.015–0.024 e bohr^−3^ and ∇^2^ *ρ*(*r*)=0.0432–0.0646 e bohr^−5^ (Table 5).^[38]^ These values are smaller than those reported for an Au⋅⋅⋅H−N hydrogen bond (*ρ*(*r*)=0.033 e bohr^−3^ and ∇^2^ *ρ*(*r*)=0.08 e bohr^−5^),^[20]^ but higher than those of the Au⋅⋅⋅H−C hydrogen bond in a gold cluster with a phenylene‐bridged diphosphine ligand (*ρ*(*r*)=0.016 e bohr^−3^ and ∇^2^ *ρ*(*r*)=0.037 e bohr^−5^).^[22]^ The NCI plots show a blue isosurface around these BCP's, indicating a strong attractive interaction (Figure 5). Furthermore, the calculated C−H bonds involved in the hydrogen bond are slightly elongated compared to the non‐interacting C−H bonds of the PCy_3_ moiety (e.g. from 1.0949 to 1.1004 Å in **P1**). NBO analysis also shows a higher occupancy of the σ*(C−H) orbital of 0.030 e to 0.040 e while the occupancy of the binding σ orbital is not significantly decreased. Furthermore, the NBO analysis (second‐order perturbation theory) revealed an orbital contribution to the Au⋅⋅⋅H−C hydrogen bond, which consists of three donor‐acceptor interactions between occupied orbitals at gold (two d‐orbitals as well as σ_Au‐P_) and the σ*(C−H) orbital. These contributions amount to approx. Δ*E*(2)=14.4 kJ mol^−1^ in **P1**, which is weaker than the Au⋅⋅⋅H−N hydrogen bond reported by Bourissou and co‐workers, but significant (see chapter 4.4 in the SI). Overall, the data clearly argue for weak hydrogen bonds with the Au atom acting as electron donor and the C−H moiety as an acceptor.^[24]^ Most interestingly, the calculations indicate that the strongest Au⋅⋅⋅H−C interaction is present in complex **P5** (e.g. Δ*E*(2)=18.8 kJ mol^−1^) see SI for details), thus being in line with the data obtained from XRD and VT NMR studies (see above). This corroborates with the calculated positive charges at the phosphorus atom in the phosphonium group, which is slightly higher in **P5** (*q*_P_=+1.63) than in **P1** (*q*_P_=+1.62).

**Table 5:** Results of the computational studies on the secondary ligand gold interactions in the **P1**, **A**, **P3** and **P5**. For further details, see the Supporting Information. Calculations were performed on the PW6B95D3/def2tzvp (MWB60 for Au) level of theory including a PCM model with aniline as solvent.

Complex	LAuCl complex						LAu(dpa)^+^ complex			
	*d*(CH‐Au) [Å] exp.	*d*(CH‐Au) [Å] calc.	*ρ*(*r*) [e bohr^−3^]	∇^2^ *ρ*(*r*) [e bohr^−5^]	occ(σ*_C−H_)		*d*(CH‐Au) [Å] calc.	*ρ*(*r*) [e bohr^−3^]	∇^2^ *ρ*(*r*) [e bohr^−5^]	occ(σ*_C−H_)
^Cy^Y_S_‐PCy_2_ (**P1**)	2.39(4)	2.438	0.0219	0.0603	0.0380		2.475	0.0204	0.0563	0.0357
										
^Ph^Y_S_‐PCy_2_ (**A**)	3.238(2)	3.207 (Au‐C)	0.0141	0.0417	–		3.252 (Au‐C)	0.0133	0.0389	–
	2.84(3)	2.844 (to PCy_2_)	0.0121	0.0386	0.0149		2.832	0.0118	0.0359	0.0210
										
^Cy^Y_Mes_‐PCy_2_ (**P3**)	2.73(3)	2.642	0.0153	0.0432	0.0299		2.680	0.0142	0.0407	0.0287
^Cy^Y_SF_‐PCy_2_ (**P5**)	2.38(5)	2.394	0.0238	0.0646	0.0397		2.387	0.0126	0.0329	0.0372

In the past, the observation of bond critical points and deshielding effects have been controversially discussed and also explained by steric compression within a complex rather than Au‐H hydrogen bonding.[^14^, ^39^] To further elaborate on this question, we calculated the C−H vibration in **P5**. While steric compression should result in a stiffer C−H vibration, hydrogen bonding should weaken and thus soften the C−H vibrational mode. Indeed, calculations show the smallest value of the v(C−H) vibrations in the PCy_3_ moiety for the C−H bond interacting with the gold center. This vibration in **P5** is red‐shifted by 82 cm^−1^ compared to the free ligand **L5**, thus supporting the presence of a weak hydrogen bond. This is also consistent with the variation in the C−H bond length upon complexation, with a small elongation, 0.006 Å, only for the PC−H bond involved in the Au⋅⋅⋅H−C interaction (see Table 34 in the SI).

To compare the observed C(sp^3^)−H⋅⋅⋅Au interaction with arene⋅⋅⋅Au interactions, QTAIM and NCI analysis were also performed for the YPhos‐AuCl complex **A** with the PPh_3_ substituted ligand. The calculations revealed an arene⋅⋅⋅Au and a C(sp^3^)H⋅⋅⋅Au interaction between the PPh_3_ phenyl and the PCy_2_ group, respectively, and the gold center. At the corresponding BCP's an electron density of *ρ*(*r*)=0.0141 and 0.0121 e bohr^−3^ is found, which—in contrast to our initial expectation—is considerably lower than the electron density observed at the BCP for the C(sp^3^)H⋅⋅⋅Au interaction in **P1**. Furthermore, the NCI scatterplot (see SI) of **P1** clearly shows the additional spike at sign(*λ*_2_)*ρ*≈0.022, representing the strongly attractive C(sp^3^)H⋅⋅⋅Au interaction, while the attractive interactions in **A** only extend to sign(*λ*_2_)*ρ*≈0.018 further confirming the weaker nature of the arene⋅⋅⋅Au and C(sp^2^)H⋅⋅⋅Au interactions.

We further analysed the corresponding cationic gold complexes with phenylacetylene as additional ligand, which are considered to be the catalytically active species. Most importantly, it was found that the C(sp^3^)H⋅⋅⋅Au interactions still persist, albeit with slightly lower values for *ρ*(*r*), ∇^2^ *ρ*(*r*) and a lower occupancy of the C−H σ* orbitals. This is well in line with the weaker donor capacity of the cationic gold centre which ultimately leads to a weaker hydrogen bond. This was already indicated by the relative energies of the different conformers (Table 4). It is noteworthy that the *ρ*(*r*) and ∇^2^ *ρ*(*r*) values in the cations—albeit being lower than in the neutral complexes—are still higher than those calculated for the arene‐Au interaction in **A**. We also would like to point out that although the arene–gold and C(sp^3^)H⋅⋅⋅Au interactions are both stabilizing effects, the electrons in both interactions flows in opposite directions. Whereas the metal center in the arene‐gold interaction acts as acceptor, it is electron donor in the hydrogen bond. These characteristics should also influence the properties of the metal and thus the catalytic activity.^[40]^

### Proof of concept

**Figure 6:**
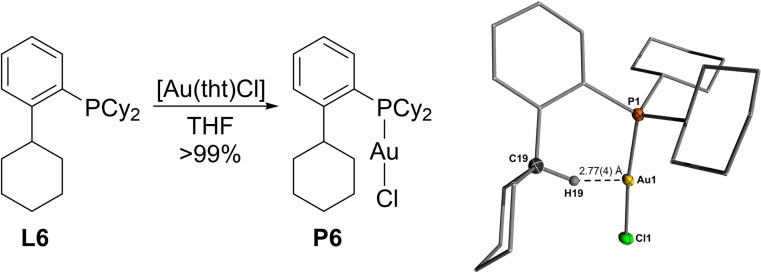
Synthesis of **P6** from [Au(tht)Cl] and free phosphine **L6** (left) and molecular structure of **P6** (right). Hydrogen atoms (except H19) omitted for clarity, ellipsoids drawn at the 50 % probability level.

Having established that gold‐hydrogen interactions are equally well suited for highly efficient YPhos based gold(I) catalysis, we next wanted to explore the generality of the concept. Au‐complexes supported by Buchwald‐type biarylphosphines have shown remarkable performance in gold(I) catalysis. We wondered if substitution of the biaryl moiety with a phenyl‐2‐cyclohexyl group would enable gold‐hydrogen interactions and thus eventually lead to similar high catalytic performance. To this end, we chose ^Cy^JohnPhos as the parent Buchwald‐type phosphine and synthesized its phenyl‐2‐cyclohexyl analogue Cy‐^Cy^JohnPhos (**L6**) according to a previously reported procedure.^[41]^ The corresponding gold complex (**P6**) was prepared from the free phosphine and [Au(tht)Cl] and could be isolated as colourless solid in quantitative yield (Figure 6). Strikingly, elucidation of the molecular structure of **P6** revealed—similar to the YPhos ligands—a relatively short Au⋅⋅⋅H−C distance of 2.77(4) Å, thus indicating the presence of a gold‐hydrogen interaction in the solid‐state structure. Broad signals between 48–25 ppm in the ^31^P{^1^H} NMR spectrum and at *δ*_H_=3.91 ppm in the ^1^H NMR spectrum for the C*H*‐proton of the phenyl‐bound cyclohexyl group also indicate the existence of this interaction in solution. Furthermore, the lower buried volume of **L6** (%V_bur_=38.1) compared to the YPhos ligands suggests that this interaction is not only enforced by steric bulk and hindered conformational changes. The presence of the CH⋅⋅⋅Au hydrogen bond is also confirmed by DFT studies. Interestingly, the energy‐optimized structure of **P6** shows a shorter CH⋅⋅⋅Au distance (2.44 Å) than found in the crystal. This is probably caused by packing effects in the crystal structure resulting in a rather large Au‐P‐C‐CCy dihedral angle of 36.3(1)° (c.f. 9.4 in the calculated structure) due to arene–cyclohexyl interactions in neighboring molecules (see SI). QT/AIM as well as NBO analyses show similar values than those obtained for the YPhos ligands, for instance an electron density of *ρ*(*r*)=0.0204 e bohr^−3^ and a Laplacian of ∇^2^ *ρ*(*r*)=0.0545 e bohr^−5^ at the bond critical point in **P6**.

Having established the presence of the CH⋅⋅⋅Au hydrogen bond in **P6**, we next compared the performance of ^**Cy**^ **JohnPhos⋅AuCl** and **P6** in the hydroamination of phenylacetylene with aniline under exact same conditions (0.1 mol %, 50 °C) as applied above. Based on the results obtained with the YPhos ligands (see above), we expected similar catalytic performance of both complexes. Indeed, both ligands performed equally well (Table 6), giving full conversion to the imine after approx. 5 h reaction time. This observation impressively confirms that arene‐gold interactions are no prerequisite for the design of efficient gold catalysts, but that Au⋅⋅⋅H−C bonds are equally suited as design principle in ligands other than ylide‐substituted phosphines. Most likely, it is also transferable to any other donor ligand, for instance carbenes by replacement of the pending aryl substituent in the Glorius and Lassaletta ligands (Figure 1) with a cyclohexyl group or other alkyl moieties.

**Table 6:** Results of the hydroamination of phenyacetylene with aniline catalyzed by the gold complexes with **L6** and CyJohnPhos. Reaction conditions: 0.1 mol % YPhos ligand and 0.1 mol % NaBAr^F^, neat, 50 °C.

Reaction time [h]	Conversion with **CyJohnPhos** [%]	Conversion with **P6** [%]
1	81	86
3	96	96
5	98	98
24	>99	>99

## Conclusion

In conclusion, we have performed a systematic study on PPh_3_ and PCy_3_‐substituted YPhos ligands to elucidate the importance of different secondary ligand metal interactions for catalysis. Whereas earlier investigations have demonstrated that the PPh_3_ group is involved in gold‐arene interactions, NMR spectroscopic studies as well as XRD analyses of the cyclohexyl‐substituted ligands, ^Cy^YPhos, revealed the presence of remarkably strong Au⋅⋅⋅H−C hydrogen bonds, amongst the shortest Au‐H interaction reported to date. Computational studies further confirmed the bonding interaction between the gold center and the PCy_3_ moiety and clearly showed that the metal acts as electron donor. A direct comparison of the stability of the gold complexes of a Ph_3_P‐substituted ligand and its PCy_3_ analogue by calorimetric studies showed that both ligands bind similarly strong to the metal. Strikingly, the PCy_3_‐substituted ligands delivered highly potent gold catalysts, which showed equal performance to their phenyl‐substituted analogues. The generality of the ability of hydrogen bonds to support stable gold catalysts was demonstrated by means of a cyclohexyl substituted derivative of the widely used biaryl phosphines. Also for this class of phosphine ligands an identical catalytic performance was observed for the biaryl and the cyclohexyl‐substituted system.

Overall, these observations clearly demonstrate that gold‐arene interactions are no prerequisite for the design of highly effective gold catalysts, which has often been assumed in the literature, but can be replaced by hydrogen bonds. Thus, not only flanking arene substituents but also hydrogen bond donors may be introduced as stabilizing moieties in the ligand structures thus further expanding the tools available for future ligand design in gold catalysis.

## Conflict of interest

The authors have filed patent WO2019030304 covering the YPhos ligands and precatalysts discussed, which is held by UMICORE and products will be made commercially available from.

## Supporting information

As a service to our authors and readers, this journal provides supporting information supplied by the authors. Such materials are peer reviewed and may be re‐organized for online delivery, but are not copy‐edited or typeset. Technical support issues arising from supporting information (other than missing files) should be addressed to the authors.

Supporting Information
