# Supplementary material for: Au⋅⋅⋅H−C Hydrogen Bonds as Design Principle in Gold(I) Catalysis
Source: Angew Chem Int Ed Engl. 2021 Aug 18;60(38):21014–24. doi: 10.1002/anie.202108581 (PMC8518757; doi:10.1002/anie.202108581)
Supplement: Supplementary file 1 — Supporting Information [file ANIE-60-21014-s001.pdf]

Angewandte Chemie
Eine Zeitschrift der Gesellschaft Deutscher Chemiker

Supporting Information

Au···H−C Hydrogen Bonds as Design Principle in Gold(I) Catalysis

Heidar Darmandeh, Julian Löffler, Nikolaos V. Tzouras, Busra Dereli, Thorsten Scherpf, Kai-Stephan Feichtner, Sofie Vanden Broeck, Kristof Van Hecke, Marina Saab, Catherine S. J. Cazin, Luigi Cavallo, Steven P. Nolan, and Viktoria H. Gessner**

anie_202108581_sm_miscellaneous_information.pdf

1. Experimental Details **3**
1.1 General methods 3
1.2 Procedure for synthesis of the ligands and gold complexes 3
1.3 Procedure for the determination of TEP values 10
1.4. Solution calorimetric measurement of the reaction enthalpy ΔH_{rxn}. 10
1.5. Procedure for Au(I) catalyzed hydroamination reactions 11
1.6. Procedure for the catalytic hydrophenoxylation of diphenylacetylene 11
1.7. Procedure for the catalytic hydrocarboxylation of diphenylacetylene 12
2. NMR spectra **14**
2.1 $^{Cy}Y_S$-PCy_2 (L1) 14
2.2 $^{Cy}Y_S$-P*i*Pr_2 (L4) 16
2.3 [Au($^{Cy}Y_S$-PCy_2)Cl] (P1) 18
2.4 [Au($^{Cy}Y_S$-PiPr_2)Cl] (P4) 20
2.5 $^{Cy}Y_{SF}$-H 22
2.6 $^{Cy}Y_{SF}$-PCy_2 (L5) 24
2.7 [Au($^{Cy}Y_{SF}$-PCy_2)Cl] (P5) 26
2.8 VT-NMR of P1 and P5 28
2.9 [Au($^{Ph}Y_S$-PCy_2)(4-CH_3O-C_6H_4)] (1a) 31
2.10 [Au($^{Cy}Y_S$-PCy_2)(4-CH_3O-C_6H_4)] (1b) 32
2.11 [{Au($^{Ph}Y_S$$PCy_2$)}$_2$(*μ*-OH)][$BF_4$] (2a) 34
2.12 [{Au($^{Cy}Y_S$$PCy_2$)}$_2$(*μ*-OH)][$BF_4$] (2b) 36
2.13 [Au(Cy-^{Cy}JohnPhos)Cl] (P6) 38
3. Crystal Structure Determination **40**
3.1 General information 40
3.2 Crystal Structure of $^{Cy}Y_s$-PCy_2 (L1) 45
3.3 Crystal Structure of $^{Cy}Y_S$-P*i*Pr_2 (L4) 49
3.4 Crystal Structure of [Au($^{Cy}Y_S$-PCy_2)Cl] (P1) 52
3.5 Crystal Structure of [Au($^{Cy}Y_S$-P*i*Pr_2)Cl] (P4) 55
3.6 Crystal Structure of $^{Cy}Y_{SF}$-H 61
3.7 Crystal Structure of $^{Cy}Y_{SF}$-PCy_2 (L5) 64
3.8 Crystal Structure of [Au($^{Cy}Y_{SF}$-PCy_2)Cl] (P5) 66
3.9 Crystal structure of 1a 69
3.10 Crystal structure of 1b 72
3.11 Crystal structure of 2a 77
3.12 Crystal structure of [Au(Cy-^{Cy}JohnPhos)Cl] (P6) 82
4. Computational Studies **85**
4.1 General remarks 85
4.2 Pictures of the conformers of the gold complexes 85
4.3 Results of the AIM and NBO analyses 87
4.4 NBOs involved in the donor-acceptor bonding with the associated second order pertubation energies 91
4.5 NCI plots 95
4.5.1 LAuCl complexes 95
4.5.2 LAu^+ complexes 96
4.5.3 NCI scatter graphs 97
4.6 Energies and coordinates of the structures 98
4.6.1 Energies and coordinates of LAuCl complexes: 98
4.7 Computational details to the calculations of the NMR chemical shifts and C-H vibration in P5 99
4.6.2 Energies and coordinates of LAu^+ complexes: 110
4.6.3 Energies and coordinates of different conformers 121
4.6.4 Energies and coordinates of the gold complexes including solvent effects 153
5. References **205**

1. Experimental Details

1.1 General methods

All experiments and crystallizations were carried out under a dry, oxygen-free argon atmosphere inside the glovebox or with standard Schlenk techniques. All manipulations regarding compounds **1a**, **1b**, **2a** and **2b** were conducted under air, with no precautions except the neutralization of chlorinated solvents by filtration though basic alumina prior to use. Other involved solvents were dried using an MBraun SPS-800 (THF, hexane, toluene, acetonitrile) or dried in accordance with standard procedures. ^{1}H, $^{13}C\{^{1}H\}$, $^{31}P\{^{1}H\}$ and $^{19}F\{^{1}H\}$ were recorded on Avance-400 and 500 spectrometers at 25 °C. All values of the chemical shift are in ppm regarding the δ-scale. All spin-spin coupling constants (J) are printed in Hertz (Hz). To display multiplicities and signal forms correctly the following abbreviations were used: s = singlet, d = doublet, m = multiplet, br = broad signal. Elemental analyses were performed on an Elementar vario MICRO-cube elemental analyzer, while for **2b** elemental analysis was performed in Université de Namur, Rue de Bruxelles 55, B-5000 Namur, Belgium. **$^{Cy}Y_S$-Li**[1], $^{Cy}Y_{oTol}$-PCy_2 (**L2**)[2], $^{Cy}Y_{Mes}$-PCy_2 (**L3**)[2], $^{Cy}Y_{oTol}$-PCy_2·AuCl (**P2**)[2], $^{Cy}Y_{Mes}$-PCy_2·AuCl (**P3**)[2], [Cy_3P-Me]I[1], PCy_2Cl[3] and (2-cyclohexylphenyl)dicyclohexyl phosphine (**L6**)[3] were prepared by following literature procedures. All other reagents were purchased from Sigma Aldrich, ABCR, Rockwood Lithium or Acros Organics and used without further purification.

1.2 Procedure for synthesis of the ligands and gold complexes

Synthesis of $^{Cy}Y_S$-PCy_2 (L1): $^{Cy}Y_S$-Li (3.00 g, 6.60 mmol, 1.00 eq.) was dissolved in toluene (30 mL) and PCy_2Cl (1.50 mL, 6.73 mmol, 1.02 eq.) was added dropwise at room temperature. The orange reaction mixture was stirred for 1h at room temperature, giving the precipitation of LiCl. The suspension was filtered via a filter cannula and the solvent of the obtained clear solution was removed under reduced pressure. Acetonitrile (20 mL) was added and the waxy-like suspension was stirred over night, during which a white precipitate formed. The white solid was collected with a glass frit, washed with acetonitrile (2 mL) and *n*-hexane (3 x 10 mL) and dried in vacuo, giving $^{Cy}Y_S$-PCy_2 as a white amorphous powder (75%, 3.20 g). Colorless crystals suitable for X-ray structure determination were grown via slow evaporation of a saturated benzene solution.

^{1}H-NMR (400 MHz, C_6D_6): δ (ppm) = 8.19 (d, J_{HH} = 8.0 Hz, 2H, *m*-CH), 6.96 (d, J_{HH} = 7.9 Hz, 2H, *o*-CH), 3.04 – 2.83 (m, 3H, PCy_3-CH), 2.66 – 2.50 (m, 2H, PCy_2-CH), 2.50 – 2.37 (m, 2H; PCy-CH_2), 2.31 – 2.19 (m, 2H, PCy-CH_2), 2.02 (s, 3H, *p*-Tol-CH_3), 1.99 – 1.95 (m, 4H, PCy-CH_2), 1.90 – 1.79 (m, 4H, PCy-CH_2), 1.76 – 1.62 (m, 14H, PCy-CH_2), 1.62 – 1.52 (m, 4H, PCy-CH_2), 1.51 – 1.40 (m, 4H, PCy-CH_2), 1.40 – 1.05 (m, 16H, PCy-CH_2). **^{13}C{^{1}H}-NMR** (101 MHz, C_6D_6): δ (ppm) = 149.1 (*ipso*-CH), 140.2 (*p*-CH) 128.8 (*m*-CH), 127.6 (*o*-CH), 37.6 (dd, J_{CP} = 4.2, 12.9 Hz, PCy_2-CH), 36.0 (dd, $^{1}J_{CP}$ = 61.8, $^{1}J_{CP}$= 87.1 Hz, PCP), 35.2 (dd, $^{3}J_{CP}$ = 11.3, $^{1}J_{CP}$ = 49.0 Hz, PCy_3-CH), 34.5 (d, $^{2}J_{CP}$ = 19.6, PCy-CH_2), 32.5 (d, $^{2}J_{CP}$ = 15.6 PCy-CH_2), 28.6 (d, $^{2}J_{CP}$ = 13.5, PCy-CH_2), 28.7 – 28.4 (m, PCy-CH_2), 28.2 (d, $^{3}J_{CP}$ = 10.4, PCy-CH_2), 27.7 (d, $^{3}J_{CP}$ = 11.9, PCy-CH_2), 27.4 (PCy-CH_2), 26.5 (PCy-CH_2), 21.1 (*p*-Tol-CH_3). **^{31}P{^{1}H}-NMR** (162 MHz, C_6D_6): δ (ppm) = 31.7 (d, $^{2}J_{PP}$ = 106.8 Hz), -7.3 (d, $^{2}J_{PP}$ = 106.8 Hz). **CHNS**: calc. for $C_{38}H_{62}O_2P_2S$: C: 70.77, H: 9.69, S: 4.97, found C: 70.86, H: 9.85, S: 4.72.

Synthesis of ^{Cy}Y$_S$-P*i*Pr$_2$ (L4): ^{Cy}Y$_S$-Li (1.00 g, 2.20 mmol, 1.00 eq.) was dissolved in toluene (30 mL) and P*i*Pr$_2$Cl (0.36 mL, 2.27 mmol, 1.03 eq.) was added dropwise at room temperature. The orange reaction mixture was stirred for 1h at room temperature, giving the precipitation of LiCl. The suspension was filtered via a filter cannula and the solvent of the obtained clear solution was removed under reduced pressure. Acetonitrile (20 mL) was added and the waxy-like suspension was stirred over night, during which a white precipitate formed. The white solid was collected with a glass frit, washed with acetonitrile (2 mL) and *n*-hexane (3 x 10 mL) and dried in vacuo, giving ^{Cy}Y$_S$-P*i*Pr$_2$ as a white amorphous powder (70%, 870 mg). Colorless crystals suitable for X-ray structure determination were grown via slow evaporation of a saturated benzene solution.

^{1}H-NMR (400 MHz, THF-*d*$_8$): δ (ppm) = 7.80 (d, J = 8.3 Hz, 2H, *m*-CH), 7.18 (d, J = 8.0 Hz, 2H, *o*-CH), 2.93 –2.76 (m, PCy$_3$-CH), 2.45 – 2.21 (m, 5H, P*i*Pr$_2$-CH+*p*-Tol-CH$_3$), 2.00 – 1.87 (m, 6H, PCy$_3$-CH$_2$), 1.86 – 1.76 (m, 6H, PCy$_3$-CH$_2$), 1.72 – 1.59 (m, 8H, PCy$_3$-CH$_2$), 1.40 – 1.20 (m, 10H, PCy$_3$-CH$_2$), 1.10 (dd, $^1J_{HH}$ = 6.9, $^2J_{PH}$ = 13.8 Hz, 6H, P*i*Pr$_2$-CH$_3$), 0.98 (dd, $^1J_{HH}$ = 7.2, $^2J_{PH}$ = 16.3 Hz, 6H, P*i*Pr$_2$-CH$_3$). **^{13}C{^{1}H}-NMR** (101 MHz, THF-*d*$_8$): δ (ppm) = 149.5 (*ipso*-CH), 141.1 (*p*-CH), 129.3 (*o*-CH), 128.1 (*m*-CH), 38.3 (dd, $^1J_{CP}$ = 61.8 Hz, $^1J_{CP}$ = 87.3 Hz, PCP), 35.9 (dd, $^3J_{CP}$ = 11.2, $^1J_{CP}$ = 49.0 Hz, PCy$_3$-CH), 29.08 (m, J = 2.9, 2.9 Hz, PCy$_3$-CH$_2$), 29.2 – 28.9 (m, PCy$_3$-CH$_2$), 28.5 (d, J=12.0, PCy$_3$-CH$_2$), 27.4 (dd, $^3J_{CP}$ = 4.7 Hz, $^1J_{CP}$ = 13.4 Hz, P*i*Pr$_2$-CH), 27.3 – 27.1 (m), 24.4 (d, $^2J_{CP}$ =21.7), 21.8 (d, $^2J_{CP}$ =18.4), 21.4 (*p*-Tol-CH$_3$). **^{31}P{^{1}H}-NMR** (162 MHz, THF-*d*$_8$): δ (ppm) = 31.5 (d, $^2J_{PP}$ = 105.7 Hz), 1.5 (d, $^2J_{PP}$ = 105.7 Hz). **CHNS**: calc. for $C_{32}H_{54}O_2P_2S$: C: 68.05, H: 9.64, S: 5.68, found C: 68.33, H: 9.72, S: 5.69.

Synthesis of [Au(^{Cy}Y$_S$-PCy$_2$)Cl] (P1): ^{Cy}Y$_S$-PCy$_2$ (70 mg, 0.11 mmol, 1.02 eq.) and thtAuCl (34 mg, 0.11 mmol, 1.00 eq.) were placed into a schlenk tube, dissolved in 5 mL of toluene and stirred for 30 min at room temperature. The reaction mixture was overlayed with 15 mL of *n*-pentane and left to stand over night, during which colorless crystals formed. The solution was removed via a filter cannula and the residual crystals were washed with a small amount of *n*-pentane (5 mL). The colorless crystals were dried in vacuo, furnishing [Au(^{Cy}Y$_S$-PCy$_2$)Cl] as a white solid. (75%, 72 mg). Colorless crystals were suitable for X-ray structure determination.

^{1}H-NMR (400 MHz, THF-*d*$_8$): δ (ppm) = 7.75 (d, J_{HH} = 8.0 Hz, 2H, *m*-CH), 7.30 (d, J_{HH} = 8.0 Hz, 2H. *o*-CH), 3.45 – 3.29 (brm, 3H, PCy$_3$-CH), 2.85 – 2.78 (m, 2H, PCy$_2$-CH), 2.60 – 2.48 (br, 2H, PCy$_2$-CH$_2$), 2.38 (s, 3H, *p*-Tol-CH$_3$; 2.25 – 2.11 (br, 2H, PCy$_2$-CH$_2$), 1.97 – 1.86 (m, 8H; PCy$_3$-CH$_2$+PCy$_2$-CH$_2$), 1.86 – 1.78 (m, 2H; PCy$_3$-CH$_2$+PCy$_2$-CH$_2$), 1.77 – 1.64 (m, 10H, PCy$_3$-CH$_2$+PCy$_2$-CH$_2$), 1.63 – 1.49 (m, 10H, PCy$_3$-CH$_2$+PCy$_2$-CH$_2$), 1.48 – 1.14 (m, 14H, PCy$_3$-CH$_2$+PCy$_2$-CH$_2$). **^{13}C{^{1}H}-NMR** (101 MHz, THF-*d*$_8$): δ (ppm) = 148.5 (*ipso*-C), 142.5 (*p*-C), 130.0 (*m*-C), 127.5 (*o*-C), 43.9 (d, $^1J_{CP}$ = 38.0 Hz, PCy$_2$-CH) , 38.0 (dd, $^1J_{CP}$ = 37.8 Hz, $^1J_{CP}$ = 73.3 Hz, PCP), 36.9 (brd, $^1J_{CP}$ = 49.4 Hz, PCy$_3$-CH), 29.9 – 28.9 (m, PCy$_3$-CH$_2$/PCy$_2$-CH$_2$), 28.5 (d, J_{CP} = 14.4 Hz, PCy$_3$-CH$_2$/PCy$_2$-CH$_2$), 28.2 (d, J_{CP} = 15.3 Hz, PCy$_3$-CH$_2$/PCy$_2$-CH$_2$), 28.1 (d, J_{CP} = 12.3 Hz, PCy$_3$-CH$_2$/PCy$_2$-CH$_2$), 27.2 (d, J_{CP} = 2.0 Hz, PCy$_3$-CH$_2$/PCy$_2$-CH$_2$), 27.0 (d, J_{CP} = 1.6 Hz, PCy$_3$-CH$_2$/PCy$_2$-CH$_2$), 21.4 (*p*-Tol-CH$_3$**). ^{31}P{^{1}H}-NMR** (162 MHz,

THF-d_8): δ (ppm) = 40.7 (d, $^2J_{PP}$ = 35.6 Hz), 29.9 (d, $^2J_{PP}$ = 35.6 Hz). **CHNS**: calc. for $C_{38}H_{62}AuClO_2P_2S$: C: 52.02, H: 7.12, S: 3.65, found C: 52.25, H: 7.01, S: 3.67.

Synthesis of [Au(^{Cy}Y$_S$-P*i*Pr$_2$)Cl] (P4): ^{Cy}Y$_S$-P*i*Pr$_2$ (100 mg, 0.18 mmol, 1.02 eq.) and thtAuCl (56 mg, 0.17 mmol, 1.00 eq.) were placed into a schlenk tube, dissolved in 5 mL of toluene and stirred for 30 min at room temperature. The reaction mixture was overlayed with 15 mL of *n*-pentane and left to stand over night, during which colorless crystals formed. The solution was removed via a filter cannula and the residual crystals were washed with a small amount of *n*-pentane (5 mL). The colorless crystals were dried in vacuo, furnishing [Au(^{Cy}Y$_S$-P*i*Pr$_2$)Cl] as a white solid. (74%, 102 mg). Colorless crystals were suitable for X-ray structure determination.

^{1}H-NMR (400 MHz, THF-d_8): δ (ppm) = 7.74 (d, J_{HH} = 8.1 Hz, 2H, *m*-CH), 7.26 (d, J_{HH} = 8.0 Hz, 2H, *o*-CH), 3.42– 3.25 (m, 3H, PCy$_3$-CH), 3.12 – 2.95 (m, 2H, P*i*Pr$_2$-CH), 2.34 (s, 3H), 1.93 – 1.80 (m, 6H, PCy$_3$-CH$_2$), 1.71 – 1.62 (m, 10H, PCy$_3$-CH$_2$), 1.62 – 1.50 (m, 6H, PCy$_3$-CH$_2$), 1.47 (d, J = 7.1 Hz, 3H, P*i*Pr$_2$-CH$_3$), 1.43 (d, J = 7.1 Hz, 3H, P*i*Pr$_2$-CH$_3$), 1.37 (d, J = 6.9 Hz, 3H, P*i*Pr$_2$-CH$_3$), 1.32 – 1.02 (m, 12H, P*i*Pr$_2$-CH$_3$ + PCy$_3$-CH$_2$). **^{13}C{^{1}H}-NMR** (101 MHz, THF-d_8): δ (ppm) = 148.2 (*ipso*-C), 142.6 (*p*-C), 130.1 (*m*-C), 127.7 (*o*-C), 39.6 (dd, ^1J = 38.6 Hz, 73.3 Hz, PCP), 36.9 (brd, ^1J = 45.8 Hz, PCy$_3$-CH), 33.9 (d, ^1J = 38.6 Hz, P*i*Pr$_2$-CH) , 29.32 (br, PCy$_3$-CH$_2$), 28.13 (d, ^3J = 12.2 Hz, PCy$_3$-CH$_2$), 27.0 (d, ^4J = 1.7 Hz, PCy$_3$-CH$_2$), 24.42 (P*i*Pr$_2$-CH$_3$), 23.54 (P*i*Pr$_2$-CH$_3$), 21.37 (*p*-Tol-CH$_3$) **^{31}P{^{1}H}-NMR** (162 MHz, THF-d_8): δ (ppm) = 50.8 (d, $^2J_{PP}$ = 35.9 Hz), 29.7 (d, $^2J_{PP}$ = 35.9 Hz). **CHNS**: calc. for $C_{32}H_{54}AuClO_2P_2S$: C: 48.21, H: 6.83, S: 4.02, found C: 48.58, H: 6.93, S: 4.28.

Synthesis of ^{Cy}Y$_{SF}$-H: [Cy$_3$P-Me]I (3.00 g, 7.10 mmol, 1.00 eq.) and KHMDS (3.26 g, 16.34 mmol, 2.30 eq.) were placed into a 100 mL schlenk flask and suspended in 50 mL THF. Perfluorobutane sulfonyl fluoride (1.40 mL, 7.81 mmol, 1.1 eq.) was slowly added to the white suspension via a syringe, upon which a color change to a deep brown occurred. The reaction mixture was stirred for 30 min at room temperature. Subsequently, the solvent was removed under reduced pressure and dichloromethane (30 mL) was added. The suspension was filtered trough a glass frit equipped with a pad of dry celite, giving a clear dark solution. Removal of the solvent under reduced pressure furnished an orange powder, which was further washed with cold *n*-hexane (3 x 10 mL) and dried for 6h at 70°C in vacuo. The title compound was obtained as a pale-yellow powder (83%, 3.40 g). Block shaped, colorless crystals suitable for X-ray structure analyses were obtained by slow evaporation of a saturated benzene solution.

^{1}H-NMR (400 MHz, THF-d_8) δ (ppm) = 2.42 – 2.25 (m, 3H, PCy$_3$-CH), 2.05 – 1.92 (m, 6H, PCy$_3$-CH$_2$), 1.90 (d, $^2J_{PH}$ = 8.4, 1H, PCHS), 1.89 – 1.78 (m, 6H, PCy$_3$-CH$_2$), 1.72 – 1.68 (m, 4H, PCy$_3$-CH$_2$), 1.66 – 1.48 (m, 6H, PCy$_3$-CH$_2$), 1.42 – 1.19 (m, 8H, PCy$_3$-CH$_2$). **^{13}C{^{1}H}-NMR** (101 MHz, THF-d_8): δ (ppm) = 33.7 (d, $^1J_{PC}$ = 49.9 Hz, PCy$_3$-CH), 28.0 (d, $^2J_{PC}$ = 9.8 Hz, PCy$_3$-CH$_2$), 28.0 –27.8 (m, PCy$_3$-CH$_2$), 27.0 (d, $^4J_{PC}$ = 1.8 Hz, PCy$_3$-CH$_2$), 23.8 (d, $^1J_{PC}$ = 105.0 Hz, PCHS). Resonances for fluorine substituted carbon atoms could not be detected. **^{19}F{^{1}H}-NMR** (377 MHz, THF-d_8): δ (ppm) = -81.82 (t, J = 10.1, 10.1 Hz, 3F), -113.61 (t, J = 13.8, 13.8 Hz, 2F), -121.12 (dt, J = 7.6, 7.6, 15.7 Hz, 2F), -126.55 (td, J = 4.7, 13.3, 13.5 Hz, 2F). **^{31}P{^{1}H}-**

NMR (162 MHz, THF-d_8): δ (ppm) = 29.1 (s). **CHNS**: calc. for $C_{23}H_{34}F_9O_2PS$: C: 47.92%, H: 5.94%, S: 5.56%, found C: 47.61%, H: 6.14%, S: 5.23%.

Synthesis of ^{Cy}Y$_{SF}$-PCy$_2$ (L5): ^{Cy}Y$_{SF}$-H (2.00 g, 3.47 mmol, 1.00 eq.) was dissolved in THF (30 mL) and *n*BuLi (1.59 M, 2.20 mL, 1.01 eq) was added dropwise at RT and stirred for 30 min. Then PCy_2Cl (0.80 mL, 3.63 mmol, 1.05 eq.) was added dropwise at room temperature. The orange reaction mixture was stirred for 1h at room temperature. The solvent was removed in vacuo and the residue was redissolved in toluene (50 mL). The suspension was filtered via a filter cannula and the solvent of the obtained clear solution was removed under reduced pressure. Acetonitrile (10 mL) was added, and the waxy-like suspension was stirred overnight, during which a white precipitate formed. The white solid was collected with a glass frit, washed with acetonitrile (2 mL) and *n*-hexane (3 x 10 mL) and dried in vacuo (1 x 10^{-3} mbar, 8 h), giving ^{Cy}Y$_{SF}$-PCy$_2$ as a white amorphous powder (72%, 1.94 g). Block-shaped, colorless crystals suitable for X-ray structure analyses were obtained by slow evaporation of a saturated benzene solution.

^{1}H-NMR (400 MHz, THF-d_8) δ (ppm) = 3.11 – 3.00 (m, 1H, PCy$_2$-CH), 2.83 – 2.71 (m, 4H, PCy$_3$-CH+PCy-CH$_2$), 2.71 – 2.63 (m, 1H, PCy-CH$_2$), 2.24 – 2.13 (m, 1H, PCy-CH$_2$), 2.14 – 2.05 (m, 2H, PCy-CH+ PCy-CH$_2$), 1.97 – 1.84 (m, 6H, PCy-CH$_2$), 1.84 – 1.71 (m, 6H, PCy-CH$_2$), 1.70 – 1.58 (m, 8H, PCy-CH$_2$), 1.57 – 1.37 (m, 16H, PCy-CH$_2$), 1.21 – 0.99 (m, 10H, PCy-CH$_2$). **^{13}C{^{1}H}-NMR** (101 MHz, THF-d_8): δ (ppm) = 42.7 (dd, $^1J_{CP}$= 14.3 Hz, $^3J_{CP}$ = 3.8 Hz, PCy$_2$-CH), 38.9 (dd, $^1J_{CP}$= 80.0, 73.0 Hz, PCP), 36.4 (PCy$_2$-CH$_2$), 36.1 (PCy$_2$-CH$_2$), 36.1 (dd, $^1J_{CP}$= 12.5 Hz, $^3J_{CP}$ = 3.0 Hz, PCy$_2$-CH), 34.3 (dd, $^1J_{CP}$= 47.2 Hz, $^3J_{CP}$ = 9.9 Hz, PCy$_3$-CH), 33.9 (d, $^2J_{CP}$ = 8.4 Hz, PCy$_3$-CH$_2$), 33.6 (PCy$_2$-CH$_2$), 33.3 (PCy$_2$-CH$_2$), 30.6 (PCy$_2$-CH$_2$), 29.3 (PCy$_2$-CH$_2$), 29.1 (PCy$_2$-CH$_2$), 28.4 – 28.2 (m, PCy-CH$_2$), 28.2 – 28.1 (m, PCy-CH$_2$), 28.1 – 28.0 (m, PCy-CH$_2$), 27.9 – 27.7 (m, PCy-CH$_2$), 27.7 – 27.5 (m, PCy-CH$_2$), 27.5 – 27.4 (m, PCy-CH$_2$), 27.4 – 27.2 (m, PCy-CH$_2$), 27.0 (PCy-CH$_2$), 26.3 (PCy-CH$_2$). Resonances for fluorine substituted carbon atoms could not be detected. **^{19}F{^{1}H}-NMR** (377 MHz, THF-d_8): δ (ppm) = -80.8 – -80.9 (m, 3F), -108.8 – -112.2 (m, 2F), -119.1 – -121.8 (m, 2F), -123.9 – -126.8 (m, 2F). **^{31}P{^{1}H}-NMR** (162 MHz, THF-d_8): δ (ppm) = 32.3 (d, $^2J_{PP}$ = 95.0 Hz), -0.7 (d, $^2J_{PP}$ = 95.0 Hz). **CHNS**: calc. for $C_{35}H_{55}F_9O_2P_2S$: C: 54.40, H: 7.17, S: 4.15, found C: 54.29, H: 7.12, S: 4.28.

Synthesis of [Au(^{Cy}Y$_{SF}$-PCy$_2$)Cl]: (P5) ^{Cy}Y$_{SF}$-PCy$_2$ (70 mg, 0.11 mmol, 1.00 eq.) and thtAuCl (29 mg, 0.11 mmol, 1.00 eq.) were placed into a schlenk tube, dissolved in 5 mL of toluene and stirred for 30 min at room temperature. The solvent was removed in vacuo (1 x 10^{-3} mbar, 3 h, 40 °C) furnishing [Au(^{Cy}Y$_{SF}$-PCy$_2$)Cl] as a white solid (quantitative yield, 91 mg). Colorless crystals suitable for X-ray structure analyses were obtained storage of a saturated solution of **P5** in *n*-pentane at -30°C.

^{1}H-NMR (400 MHz, CD_2Cl_2) δ (ppm) = 3.47 (br, 3H, PCy$_3$-CH), 2.82 – 2.75 (m, 1H, PCy-CH), 2.42 – 2.16 (m, 5H, PCy-CH+ PCy-CH$_2$), 2.09 – 2.00 (m, 6H, PCy-CH$_2$), 1.90 – 1.68 (m, 20H, PCy-CH$_2$), 1.49 – 1.23 (m, 20H, PCy-CH$_2$). **^{13}C{^{1}H}-NMR** (101 MHz, CD_2Cl_2): δ (ppm) = 44.6 (dd, $^1J_{CP}$= 34.1, $^3J_{CP}$ = 2.1, PCy$_2$-CH), 41.8 (d, $^1J_{CP}$ =33.8 Hz, PCy$_2$-CH), 40.8 (dd, $^1J_{CP}$ = 67.5, 24.9 Hz, PCP), 38.2 – 37.9 (m, PCy-CH$_2$), 37.4 – 37.1 (m, PCy-CH$_2$), 36.8 (br, PCy$_3$-CH), 32.2

(d, J=1.8 Hz, PCy-CH_2), 31.6 (PCy-CH_2), 30.4 (PCy-CH_2), 29.2 (PCy-CH_2), 28.8 (PCy-CH_2), 28.3 (PCy-CH_2), 28.1 (PCy-CH_2), 27.9 – 27.5 (m, PCy-CH_2), 27.3 (PCy-CH_2), 27.2(PCy-CH_2), 26.8 – 26.6 (m, PCy-CH_2), 26.6 – 26.2 (m, PCy-CH_2), 26.1 (PCy-CH_2). Resonances for fluorine substituted carbon atoms could not be detected. **^{19}F{^{1}H}-NMR** (377 MHz, CD_2Cl_2): δ (ppm) = -80.7 – -81.5 (m, 3F), -106.4 – -109.7 (m, 2F), -120.0 – -122.5 (m, 2F), -124.8 – -126.9 (m, 2F). **^{31}P{^{1}H}-NMR** (162 MHz, CD_2Cl_2): δ (ppm) = 43.3 (d, $^2J_{PP}$ = 26.8 Hz), 34.8 (d, $^2J_{PP}$ = 26.8 Hz). **CHNS**: calc. for $C_{35}H_{55}AuClF_9O_2P_2S$: C: 41.82, H: 5.52, S: 3.19, found C: 41.91, H: 5.75, S: 3.22.

Synthesis of [Au(^{Ph}Y$_S$-PCy$_2$)(4-CH$_3$O-C$_6$H$_4$)] (1a): To a 4.0 mL vial equipped with a stirring bar and septum screw-cap were added [Au(^{Ph}Y$_S$-PCy$_2$)Cl] (200 mg, 0.233 mmol), 4-methoxyphenylboronic acid (38.9 mg, 0.256 mmol) and potassium carbonate (96.5 mg, 0.698 mmol). Ethanol (1.8 mL) was added and the resulting white suspension was stirred under air, at room temperature for 16 hours. Full conversion was determined by NMR analysis of an aliquot and the solvent was removed on a rotary evaporator. THF (10 mL) was added to the residue and the mixture was filtered through a syringe filter and a 2 cm basic alumina plug. The solvent was evaporated under vacuum and the resulting white solid was recrystallized by addition of a minimal amount of THF and rapid addition of 10 mL of pentane. The solvent was decanted and pentane (3x3 mL) was used to wash the product, which was obtained as a white powder in 83% yield (180 mg, 0.193 mmol). Crystals suitable for Xray-diffraction analysis were grown by vapor diffusion of pentane into a saturated solution of the product in dichloromethane.

^{1}H-NMR (400 MHz, CD_2Cl_2) δ (ppm) = 7.76 – 7.69 (m, 6H, *o*-CH PPh_3), 7.54 (dd, J = 7.8, 6.3 Hz, 3H, *p*-CH PPh_3), 7.36 (td, J = 7.8, 3.1 Hz, 6H, *m*-CH PPh_3), 7.13 (d, J = 7.0 Hz, 2H, *o*-CH Tol), 7.00 (d, J = 8.0 Hz, 2H, *m*-CH Tol), 6.65 (brs, 2H, *o*-CH Au-Ar), 6.58 (d, J = 8.0 Hz, 2H, *m*-CH Au-Ar), 3.67 (s, 3H, OCH_3), 2.77 (brs, 2H, PCy-CH), 2.35 (s, 3H, CH_3 Tol), 2.26 (brs, 4H, PCy-CH_2), 1.92 – 1.80 (m, 4H, PCy-CH_2), 1.72 (d, J = 11.4 Hz, 2H, PCy-CH_2), 1.66 – 1.56 (m, 2H, PCy-CH_2), 1.45 – 1.16 (m, 8H, PCy-CH_2). **^{13}C{^{1}H}-NMR** (101 MHz, CD_2Cl_2): δ (ppm) = 166.8 (d, J_{CP} = 106.3 Hz, Au-C), 157.4 (s,*C*-OCH_3), 146.6 (s, C_{Tol}-S), 141.4 (s, *C*-CH_3), 139.7 (s, *o*-*C*H Au-Ar), 135.5 (d, J_{CP} = 9.4 Hz, *o*-*C*H PPh_3), 132.7 (d, J_{CP} = 2.8 Hz, *p*-*C*H PPh_3), 129.3 (s, *m*-*C*H Tol), 128.7 (d, J_{CP} = 12.5 Hz, *m*-*C*H PPh_3), 126.8 (d, J_{CP} = 93.9 Hz, C-P PPh_3), 126.4 (s, *o*-*C*H Tol), 112.9 (d, J_{CP} = 5.9 Hz, *m*-*C*H Au-Ar), 55.3 (s, O*C*H$_3$), 42.8 (dd, J_{CP} = 100.4, 19.0 Hz, PCP), 41.3 (d, J_{CP} = 26.3 Hz, PCy-*C*H), 33.5 (brs, PCy-*C*H$_2$), 32.3 (d, J_{CP} = 3.4 Hz, PCy-*C*H$_2$), 28.2 (s, PCy-*C*H$_2$), 28.0 (s, PCy-*C*H$_2$), 27.9 (s, PCy-*C*H$_2$), 26.8 (d, J = 1.1 Hz, PCy-*C*H$_2$), 21.6 (s, CH_3 Tol). **^{31}P{^{1}H}-NMR** (162 MHz, CD_2Cl_2): δ (ppm) = 46.5 (d, J = 67.5 Hz), 18.4 – 17.9 (brm). **CHN:** calc. for $C_{45}H_{51}AuO_3P_2S$: C: 58.06, H: 5.52; found C: 58.17, H: 5.65.

Synthesis of [Au(^{Cy}Y$_S$-PCy$_2$)(4-CH$_3$O-C$_6$H$_4$)] (1b): To a 4.0 mL vial equipped with a stirring bar and septum screw-cap were added [Au(^{Cy}Y$_S$-PCy$_2$)Cl] (100 mg, 0.114 mmol), 4-methoxyphenylboronic acid (19.1 mg, 0.125 mmol) and potassium carbonate (47.3 mg, 0.342 mmol). Ethanol (0.8 mL) was added and the resulting white suspension was stirred under air, at room temperature for 16 hours. Full conversion was determined by NMR analysis of an aliquot and the solvent was removed on a rotary evaporator. THF (5.0 mL) was added to the residue and the mixture was filtered through a syringe filter and a 2 cm basic alumina plug.

The solvent was evaporated under vacuum and the resulting oil was dried on a Schlenk line under high vacuum. Pentane (4.0 mL) was added to triturate the product and further portions of pentane (2x3 mL) were used to wash the product, which was obtained as a white powder in 99% yield (99.0 mg, 0.112 mmol). Crystals suitable for Xray-diffraction analysis were grown by vapor diffusion of pentane into a saturated solution of the product in dichloromethane.

^{1}H-NMR (400 MHz, CD_2Cl_2) δ (ppm) = 7.76 (d, J = 8.3 Hz, 2H, *o*-CH Tol), 7.37 – 7.31 (m, 2H, *o*-CH Au-Ar), 7.26 (d, J = 8.3 Hz, 2H, *m*-CH Tol), 6.78 (d, J_{HH} = 8.4, 2H, *m*-CH Au-Ar), 3.74 (s, 3H, OCH_3), 3.52 – 343 (m,, 3H, PCy_3-CH), 2.87 – 278 (m, 2H, PCy_2-CH), 2.56 – 2.46 (m, 2H PCy_3-CH_2), 2.39 (s, 3H, CH_3 Tol), 2.26 – 2.16 (m, 2H PCy_2-CH_2), 1.93 – 1.92 (m, 8H PCy-CH_2), 1.82 – 1.67 (m, 16H, PCy-CH_2), 1.63 – 1.45 (m, 9H, PCy-CH_2), 1.43 – 1.13 (m, 16H, PCy-CH_2). **^{13}C{^{1}H}-NMR** (101 MHz, CD_2Cl_2): δ (ppm) = 165.6 (dd, J_{CP} = 104.4, 1.5 Hz,C-Au), 157.8 (s, *C*- OCH_3), 148.3 (s, *o*-*C*H Au-Ar), 141.9 (s, *C*-S Tol), 140.2 (s, *C*-CH_3 Tol), 129.6 (s, , *m*-CH Tol), 126.5 (s, , *o*-CH Tol), 113.4 (d, J_{CP} = 5.8 Hz, *m*-*C*H Au-Ar), 55.3 (s, O*C*H_3), 43.2 (d, J_{CP} = 30.5 Hz, PCy_2-CH), 38.0 – 36.9 (m, overlapping PCP and PCy_3-*C*H), 34.2 (d, J_{CP} = 2.9 Hz, PCy-*C*H_2), 33.4 (d, J_{CP} = 3.8 Hz, PCy-*C*H_2), 28.9 (s, PCy-*C*H_2), 28.3 (d, J_{CP} = 13.9 Hz, PCy-*C*H_2), 28.0 (d, J_{CP} = 13.7 Hz, PCy-*C*H_2), 27.9 (d, J_{CP} = 12.2 Hz, PCy-*C*H_2), 26.9 (d, J_{CP} = 1.5 Hz, PCy-*C*H_2), 26.6 (d, J_{CP} = 1.0 Hz, PCy-*C*H_2), 21.5 (s, *C*H_3 Tol). **^{31}P{^{1}H}-NMR** (162 MHz, CD_2Cl_2): δ (ppm) = 46.8 (d, J = 46.7 Hz), 31.7 (d, J = 46.7 Hz). **CHN:** calc. for $C_{45}H_{69}AuO_3P_2S$: C: 56.95, H: 7.33, found C: 56.32, H: 6.93.

Synthesis of [{Au(^{Ph}Y$_S$PCy$_2$)}$_2$(*μ*-OH)][BF$_4$] (2a): To a 4.0 mL vial equipped with a stirring bar and septum screw-cap was added **1a** (145 mg, 0.156 mmol) and was suspended in acetonitrile (1.6 mL) under air. Tetrafluoroboric acid diethyl etherate (26.5 μL, 0.172 mmol) was quickly added to the stirred white suspension at room temperature and a colourless solution formed immediately. The solution was allowed to stir for 5 minutes and the solvent was evaporated under vacuum. Dichloromethane (5.0 mL) was added and the solution was transferred to a separating funnel. The organic layer was washed with deionized water (3x5 mL), dried over anhydrous magnesium sulphate, filtered through a syringe filter and evaporated to dryness. The resulting colourless solid was recrystallized by addition of a minimal amount of dichloromethane and rapid addition of 10 mL of pentane. The solvent was decanted and pentane (3x3 mL) was used to wash the product, which was obtained as a white powder in 92% yield (125 mg, 0.071 mmol). Crystals suitable for Xray-diffraction analysis were grown by vapor diffusion of pentane into a saturated solution of the product in dichloromethane.

^{1}H-NMR (400 MHz, CD_2Cl_2) δ (ppm) = 7.71 – 7.60 (m, 18H, *o*-CH and *p*-CH PPh_3), 7.46 (td, J = 7.7, 3.2 Hz, 12H, *m*-CH PPh_3), 7.07 (brs, 4H, *o*-CH Tol), 7.00 (d, J = 8.1 Hz, 4H, *m*-CH Tol), 2.34 (s, 6H, CH_3 Tol), 2.20 – 2.10 (m, 4H, PCy_2-CH_2), 2.03 (brs, 4H, PCy_2-CH_2), 1.87 – 1.67 (m, 12H, PCy_2-CH_2), 1.40 – 1.07 (m, 22H, PCy_2-CH_2), 0.26 (s, 1H, OH), the signal from PCy_2-CH could not be detected in 2D NMR. **^{13}C{^{1}H}-NMR** (101 MHz, CD_2Cl_2): δ (ppm) = 145.8 (brs, C-S Tol), 142.3 (s, *C*-CH_3 Tol), 135.4 (d, J_{CP} = 9.5 Hz, *o*-CH PPh_3), 133.4 (d, J_{CP} = 2.6 Hz, *p*-CH PPh_3), 129.4 (s, *m*-*C*H Tol), 129.3 (d, J_{CP} = 12.5 Hz, *m*-CH PPh_3), 126.7 (s, *o*-*C*H Tol), 126.0 (d, J_{CP} = 92.7 Hz, *C*-PPh_3), 41.8 (dd, J_{CP} = 98.9, 46.5 Hz, P*C*P) 40.9 (brd, J_{CP} = 38.0 Hz, PCy_2-*C*H), 33.8 (s, PCy_2-*C*H_2), 33.0 (s, PCy_2-*C*H_2), 27.6 (s, PCy_2-*C*H_2), 27.5 (s, PCy_2-*C*H_2), 27.4 (s, PCy_2-*C*H_2), 26.5 (s, PCy_2-*C*H_2), 21.6 (s, *C*H_3 Tol). **^{31}P{^{1}H}-NMR** (162 MHz, CD_2Cl_2): δ (ppm) = 36.37 (d, J = 44.3 Hz), 20.12 (d, J = 44.3 Hz). **^{19}F{^{1}H}-NMR** (471 MHz,

CD_2Cl_2): δ (ppm) = -153.01, -153.06. **CHN:** calc. for $C_{76}H_{89}Au_2BF_4O_5P_4S_2$: C: 52.12, H: 5.12, found C: 52.77, H: 5.42.

Synthesis of [{Au($^{Cy}Y_SPCy_2$)}$_2$(μ-OH)][BF$_4$] (2b): To a 4.0 mL vial equipped with a stirring bar and septum screw-cap was added **1b** (90.0 mg, 0.103 mmol) and was suspended in acetonitrile (1.0 mL) under air. Tetrafluoroboric acid diethyl etherate (17.3 µL, 0.113 mmol) was quickly added to the stirred white suspension at room temperature and a colourless solution formed immediately. The solution was allowed to stir for 5 minutes and the solvent was evaporated under vacuum. Dichloromethane (5.0 mL) was added and the solution was transferred to a separating funnel. The organic layer was washed with deionized water (3x5 mL), dried over anhydrous magnesium sulphate, filtered through a syringe filter and evaporated to dryness. The resulting solid was recrystallized by addition of a minimal amount of dichloromethane and rapid addition of 5.0 mL of pentane. The solvent was decanted and pentane (3x3 mL) was used to wash the product, which was obtained as a white powder in 82% yield (75.1 mg, 0.042 mmol). Crystals suitable for Xray-diffraction analysis could not be grown despite repeated attempts.

^{1}H-NMR (400 MHz, CD_2Cl_2) δ (ppm) = 7.70 (d, J = 8.2 Hz, 4H, *o*-CH Tol), 7.29 (d, J = 8.1 Hz, 4H, *m*-CH Tol), 3.15 (m, 6H, PCy_3-CH), 2.73 (m, 4H, PCy_2-CH), 2.50 (s, 4H, PCy-CH_2), 2.41 (s, 6H, CH_3 Tol), 2.15 (s, 4H, PCy-CH_2), 1.95 (brd, J = 7.9 Hz, 6H, PCy-CH_2), 1.89 – 1.66 (m, 32H, PCy-CH_2), 1.58 – 1.39 (m, 26H, PCy-CH_2), 1.32 – 1.13 (m, 28H, PCy-CH_2), the exchangeable O-H proton signal could not be clearly detected. **^{13}C{^{1}H}-NMR** (101 MHz, CD_2Cl_2): δ (ppm) = ^{13}C NMR (101 MHz, CD_2Cl_2) δ 146.8 (s, C-S Tol), 142.9 (s, *C*-CH_3 Tol), 129.9 (s, *m*-*C*H Tol), 126.9 (s, *o*-*C*H Tol), 43.4 (d, J_{CP} = 40.1 Hz, PCy_2-*C*H), 36.3 (apparent d, J_{CP} = 43.7 Hz, P*C*P), 36.0 (brd, J_{CP} = 44.4 Hz, PCy_3-*C*H), 35.3 (d, J_{CP} = 17.3 Hz, PCy-*C*H_2), 34.3 (brs, PCy-*C*H_2), 28.6 (brs, PCy-*C*H_2), 27.9 (s, PCy-*C*H_2), 27.8 (s, PCy-*C*H_2), 27.6 (s, PCy-*C*H_2), 26.7 (s, PCy-*C*H_2), 26.4 (s, PCy-*C*H_2), 21.6 (s, *C*H_3 Tol). **^{31}P{^{1}H}-NMR** (162 MHz, CD_2Cl_2): δ (ppm) = 35.86 (d, J = 29.5 Hz), 28.84 (d, J = 29.6 Hz). **^{19}F{^{1}H}-NMR** (471 MHz, CD_2Cl_2): δ (ppm) = -152.75, -152.81. **CHN:** calc. for $C_{76}H_{125}Au_2BF_4O_5P_4S_2$: C: 51.07, H: 7.05, found C: 51.38, H: 6.70.

Synthesis of Cy-^{Cy}JohnPhos (P6)

(2-cyclohexylphenyl)dicyclohexylphosphine (**L6**) (100 mg, 0.280 mmol, 1.0 eq.) and [Au(tht)Cl] (90 mg, 0.280 mmol, 1.0 eq.) were placed into a schlenk tube and THF (5 mL) was added. The mixture was stirred for 10 min at room temperature. Subsequently, all volatiles were evaporated and the residue was dried for several hours in vacuo, giving the title compound as a pure colourless solid in quantitative yield (165 mg, 0.280 mmol). Colourless crystals suitable for X-ray structure determination were obtained by overlaying a saturated DCM solution of **P6** with *n*-pentane.

^{1}H-NMR (400 MHz, CD_2Cl_2) δ (ppm) = 7.57 – 7.42 (m, 3H, *CH*), 7.31 – 7.24 (m, 1H, *CH*), 4.02 – 3.81 (m, 1H, Cy-*CH*), 2.40 – 2.22 (m, 2H, PCy_2-*CH*), 2.22 – 2.08 (m, 2H, *CH$_2$*), 1.89 – 1.79 (m, 6H, *CH$_2$*), 1.80 – 1.72 (m, 2H, *CH$_2$*), 1.72 – 1.09 (m, 20H, *CH$_2$*).**^{13}C{^{1}H}-NMR** (101 MHz, CD_2Cl_2): δ (ppm) = 154.0 (d, J = 9.2 Hz, C_{ipso}), 134.5 (*CH*), 132.1 (d, J = 2.5 Hz, *CH*), 129.1 (d, J = 8.2 Hz, *CH*), 126.3 (d, J = 9.4 Hz, *CH*), 122.6 (d, J = 51.7 Hz, C_{ortho}), 43.3 (d, J = 9.7 Hz, Cy-*CH*), 36.3 (d, J = 34.1 Hz, PCy_2-*CH*), 35.4 (Cy-*CH$_2$*), 31.4 (d, J = 3.6 Hz, Cy-*CH$_2$*), 29.8 (Cy-*CH$_2$*), 27.3 – 26.9 (m, Cy-*CH$_2$*), 26.6 (Cy-*CH$_2$*), 26.3 (d, J = 1.8 Hz, Cy-*CH$_2$*).

$^{31}P\{^{1}H\}$-NMR (162 MHz, CD_2Cl_2): δ (ppm) = 47.9 – 24.8 (vbr). **CHN:** calc. for $C_{24}H_{37}AuClP$: C:48.95, H.6.33; found: C: 48.77, H: 6.25.

1.3 Procedure for the determination of TEP values

IR spectra were recorded on a Nicolet iS5 FT-IR in transmission mode with a Specac “Omni-cell” with KBr plates and a 0.1 mm spacer at 22 °C.

Procedure for $\tilde{\nu}_{(CO)}$Rh determination: 5.00 mg (19.4 µmol) $Rh(acac)(CO)_2$ were dissolved in 1 mL of DCM in a glovebox. 19.4 µmol of the phosphine were added to the solution and the solution was stirred for 15 min until gas evolution ceased. The solution was added into the IR cell using a syringe. The cell was closed, taken outside the glovebox and an IR spectrum was recorded. The TEP value was calculated *via* the linear correlation of TEP and $\tilde{\nu}_{(CO)}$Rh described by Carrow[4].

Table 1. Determined $\tilde{\nu}_{(CO)}$ frequencies of the novel ^{Cy}YPhos ligands and their corresponding $TEP_{calc.}$ values.

Compound	$\tilde{\nu}_{(CO)}$Rh	calcd. TEP
$^{Cy}Y_S$-PCy_2 (**L1**)	1956.8	2057.0
$^{Cy}Y_S$-P*i*Pr_2 (**L4**)	1959.7	2058.7
$^{Cy}Y_{SF}$-PCy_2 (**L5**)	1961.7	2059.8

1.4. Solution calorimetric measurement of the reaction enthalpy ΔH_{rxn}.

NMR monitoring: The kinetics of the reaction of interest were qualitatively examined via ^{31}P NMR spectroscopy by mixing the reagents in a *J*. Young NMR tube and rapidly recording spectra. If the reaction reached completion within 2 hours (most reactions examined reached full conversion within 5 minutes), then it was judged suitable for calorimetric investigation. All measurements were performed in the same manner with highly pure ligands in a manner previously reported using a Setaram C-80 Calvet calorimeter. All parts of the calorimetric sample cell were initially washed, dried in an oven maintained at 110°C overnight prior to bringing the dismantled cell inside the glovebox for loading. An accurately weighed amount of the ligand (ca 15-20 mg) was weighed in the tall bucket of the Setaram C-80 reverse mixing cell and 2mL of CH_2Cl_2 was added to dissolve it. In a seperate vial, 6 equivalents of [Au(DMS)Cl] was weighed and dissolved in 3mL of CH_2Cl_2. 2mL of this solution was subsequently injected in the calorimeter cell between the bucket and the outer wall. The cell was then sealed, removed from the glovebox and loaded into the calorimeter. After thermal equilibration at 30°C, the reaction was initiated by inverting the calorimeter and the reaction monitored until completion in an isothermal mode at 30°C. Following the return of the thermograph to the baseline, the cell was removed from the calorimeter, returned to the

glovebox, the cell opened and its content (where all species were in solution) was analysed by ^{31}P NMR spectroscopy and confirmed the quantitative conversion to the desired product.

1.5. Procedure for Au(I) catalyzed hydroamination reactions

The reaction was conducted in a similar fashion as previously described by our group.[5] A 2 mL glass vial with a rubber cap and a stir bar was charged in a glovebox with the indicated amount of LAuCl and NaBAr^{F}. The amine (5.25 mmol) and the alkyne (5.00 mmol) were added via syringe. The vial was heated on a hotplate to the indicated temperature while stirring. Small aliquots were removed via a syringe and added directly to an NMR tube to monitor the reaction progress. Yields were calculated by integration of the peak for the alkyne starting material with respect to the peak for the imine product in the ^{1}H-NMR spectrum.

Catalysis was also performed with AgOTf as abstraction reagent using the same protocol. These studies gave the following results:

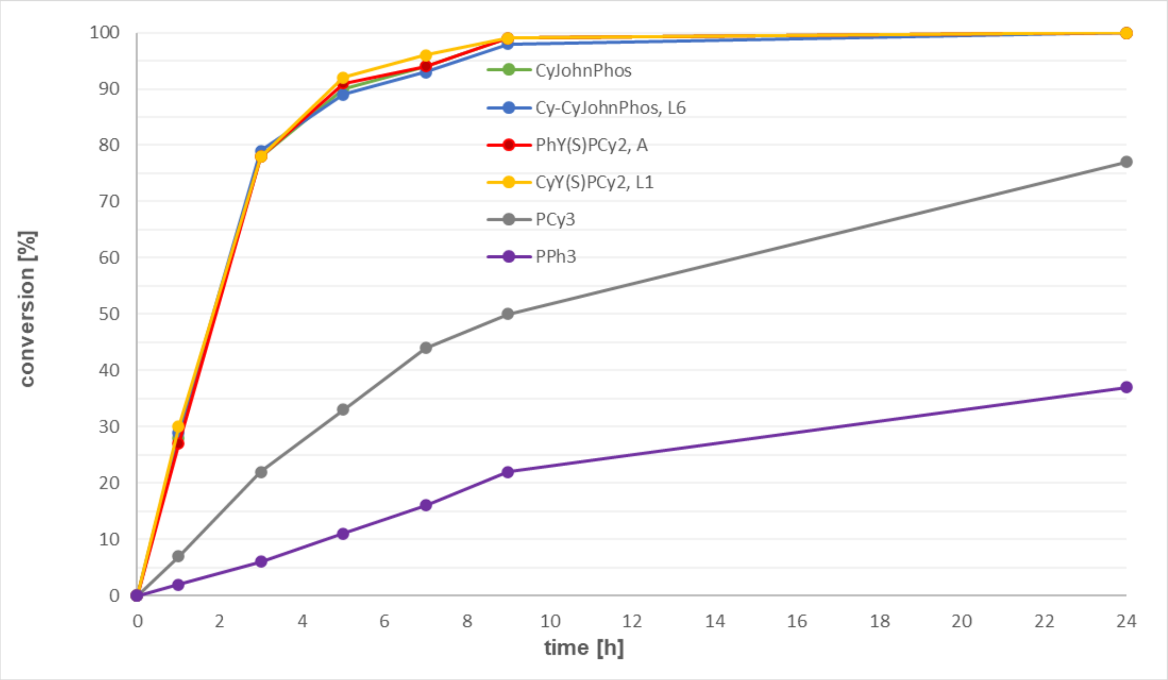

1.6. Procedure for the catalytic hydrophenoxylation of diphenylacetylene

To a 4 mL vial equipped with a septum screw-cap were added [Au] (1 mol%, or 0.5 mol% of digold, 0.0025 mmol), diphenylacetylene (89.1 mg, 0.5 mmol) and phenol (51.8 mg, 0.55 mmol). Toluene (1.0 mL) was added and the reaction was stirred at 80 ^{o}C for 1 hour in a preheated metal block-equipped stirring plate. The reaction was allowed to cool down and 1.00 mL of a 0.5 M solution of 1,3,5-trimethoxybenzene in dichloromethane was added. The yield was determined by integration of the 6.66 ppm signal of the desired Z-enol ether and comparison to the signals of the internal standard.

In case of NaBAr^{F} activation, the chloride abstractor (2 mol%, 8.86 mg, 0.010 mmol) was added after the addition of toluene, under air. 0% conversion was observed.

1.7. Procedure for the catalytic hydrocarboxylation of diphenylacetylene

To a 4 mL vial equipped with a septum screw-cap were added [Au] (1 mol%, or 0.5 mol% of digold, 0.0025 mmol), diphenylacetylene (89.1 mg, 0.5 mmol) and benzoic acid (67.2 mg, 0.55 mmol). Toluene (0.04 mL) was added, the mixture was heated at 80 ^{o}C without stirring until a melt was formed, and the reaction was stirred at 80 ^{o}C for 16 hours in a preheated metal block-equipped stirring plate. Alternatively, the procedure was followed excluding addition of toluene. The reaction was allowed to cool down and 1.00 mL of a 0.5 M solution of 1,3,5-trimethoxybenzene in dichloromethane was added. The yield was determined by integration of the 6.81 ppm signal of the desired Z-enol ester and comparison to the signals of the internal standard.

In case of $NaBAr^F$ activation, the chloride abstractor (2 mol%, 8.86 mg, 0.010 mmol) was added after the addition of [Au], under air or in the glovebox. An additional experiment was done under air, in which [Au] and the chloride abstractor were premixed in 0.05 mL of dichloromethane for 10 minutes, the solvent was removed under vacuum and the reactants were added as described in the above procedure. 0% conversion was observed in all cases.

Table 2. Results and reaction condition for the Au(I) catalyzed hydroamination of phenylacetylene with aniline.

**Catalyst**	**[Au($^{Cy}Y_{S}$-PCy$_2$)Cl] (P1)**	**[Au($^{Cy}Y_{oTol}$-PCy$_2$)Cl] (P2)**	**[Au($^{Cy}Y_{Mes}$-PCy$_2$)Cl] (P3)**	**[Au($^{Cy}Y_{S}$-P*i*Pr$_2$)Cl] (P4)**	**[Au($^{Cy}Y_{SF}$-PCy$_2$)Cl] (P5)**	**[Au($^{Cy}Y_{S}$-PCy$_2$)Cl] (P1)**	**[Au($^{Cy}Y_{SF}$-PCy$_2$)Cl] (P5)**	**P6**
Loading [mol%]	0.1	0.1	0.1	0.1	0.1	0.05	0.05	0.1
Temp. [°C]	50	50	50	50	50	50	50	50
0 h	0	0	0	0	0	0	0	0
1 h	86	74	77	79	50	58	25	86
3 h	96	84	94	90	71	77	47	96
5 h	99	94	97	95	85	85	67	98
24 h	>99	>99	>99	>99	95	>99	89	>99

2. NMR spectra

2.1 $^{Cy}Y_S$-PCy_2 (L1)

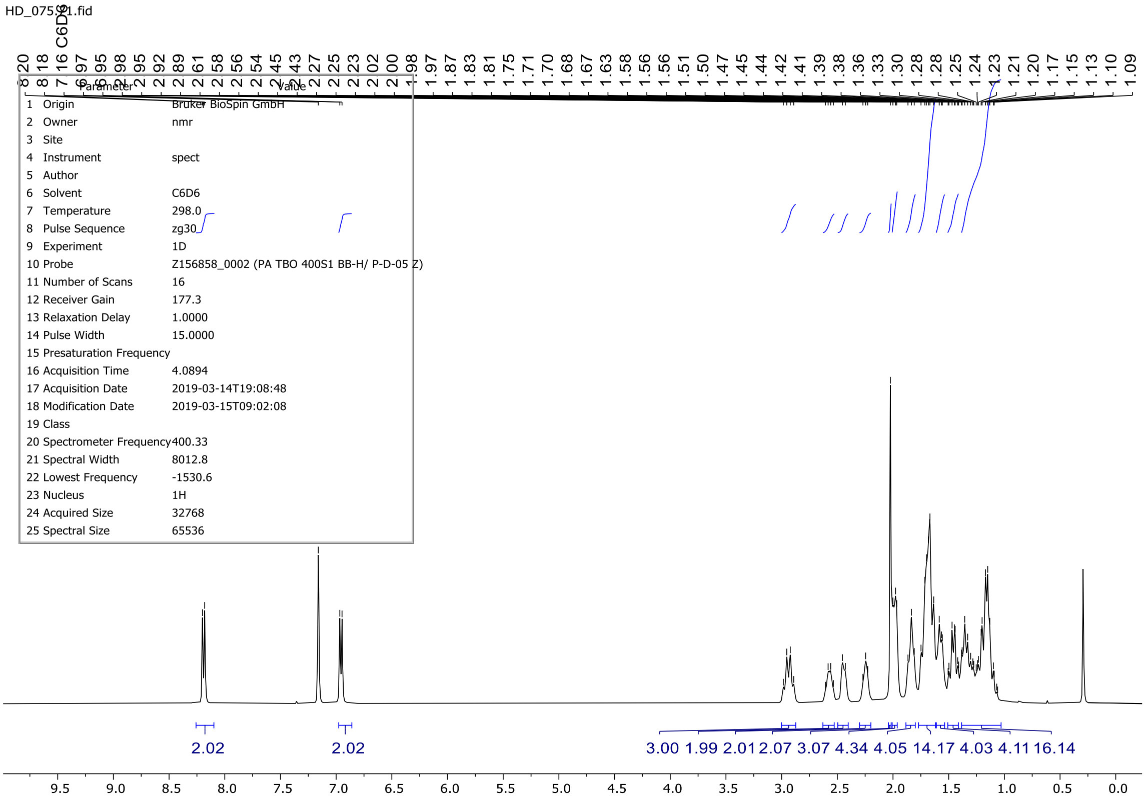

Figure 1. ^{1}H-NMR spectrum of **L1** in C_6D_6.

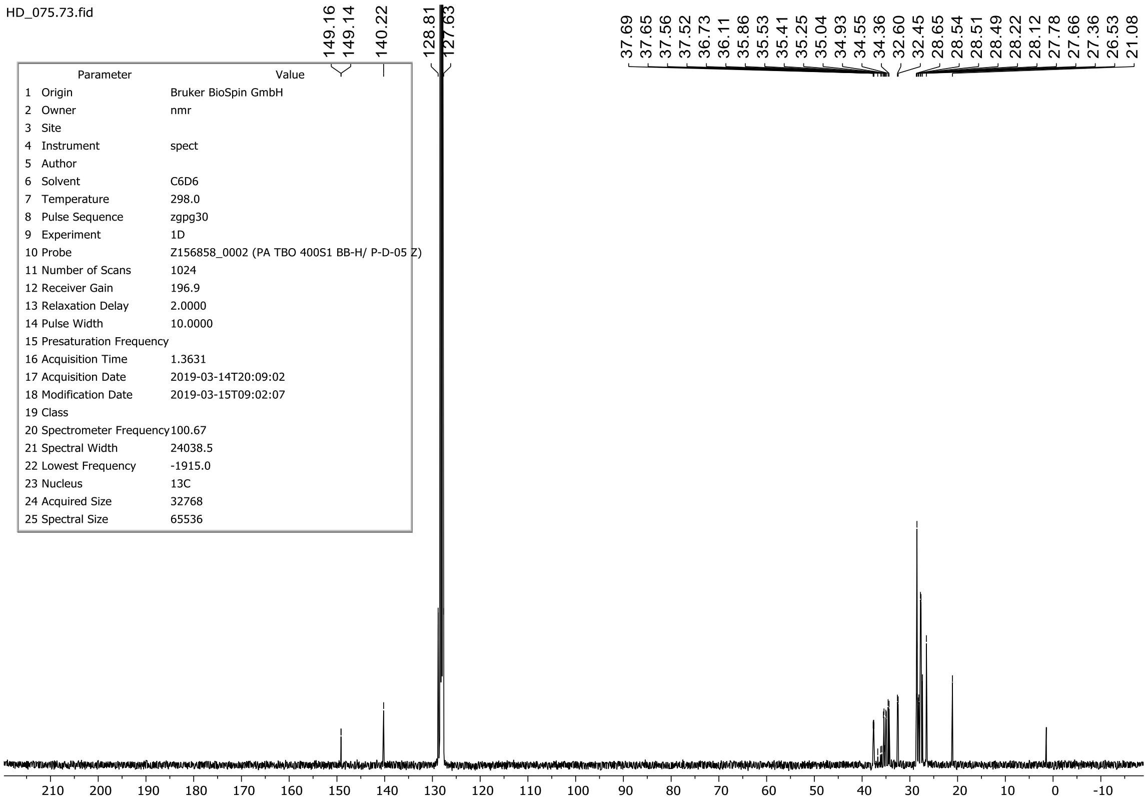

Figure 2. $^{13}C\{^{1}H\}$-NMR spectrum of **L1** in C_6D_6.

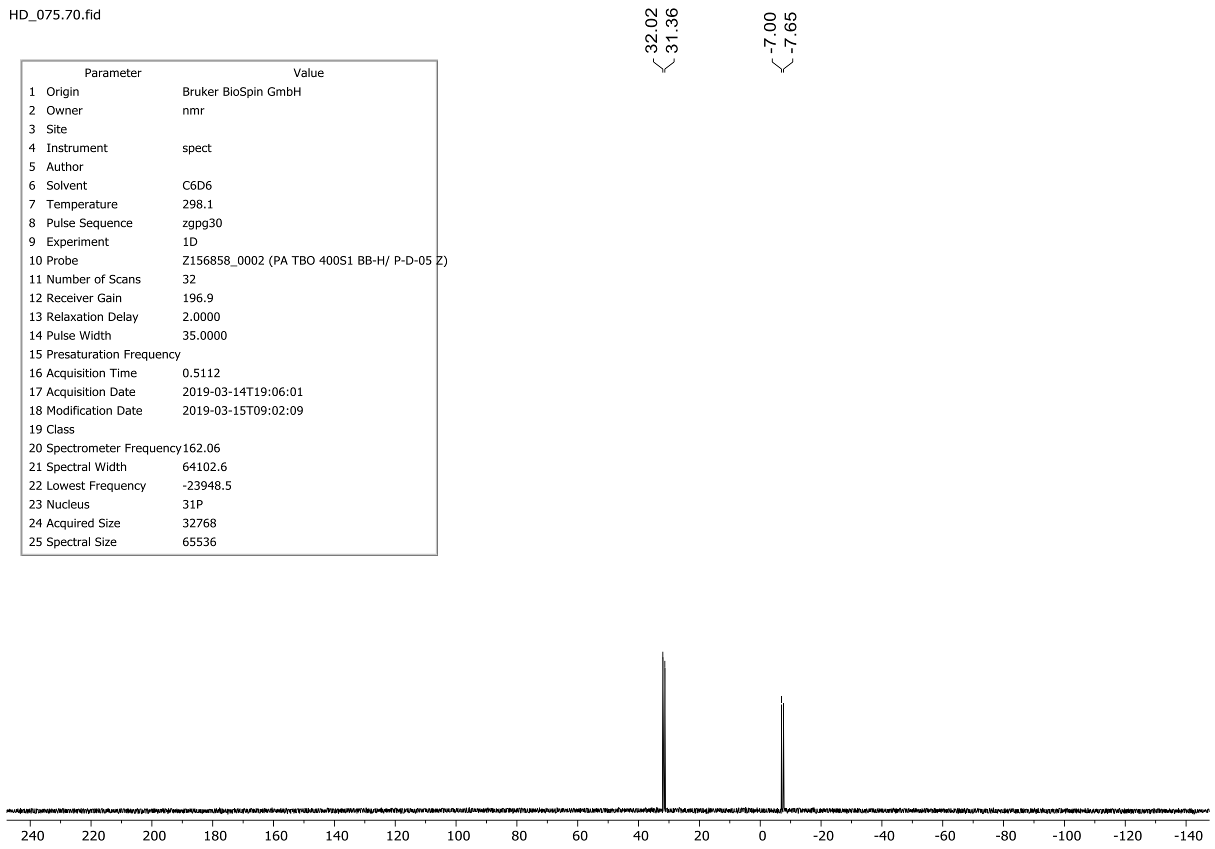

Figure 3. $^{31}P\{^{1}H\}$-NMR spectrum of **L1** in C_6D_6.

2.2 ^{Cy}Y$_S$-P*i*Pr$_2$ (L4)

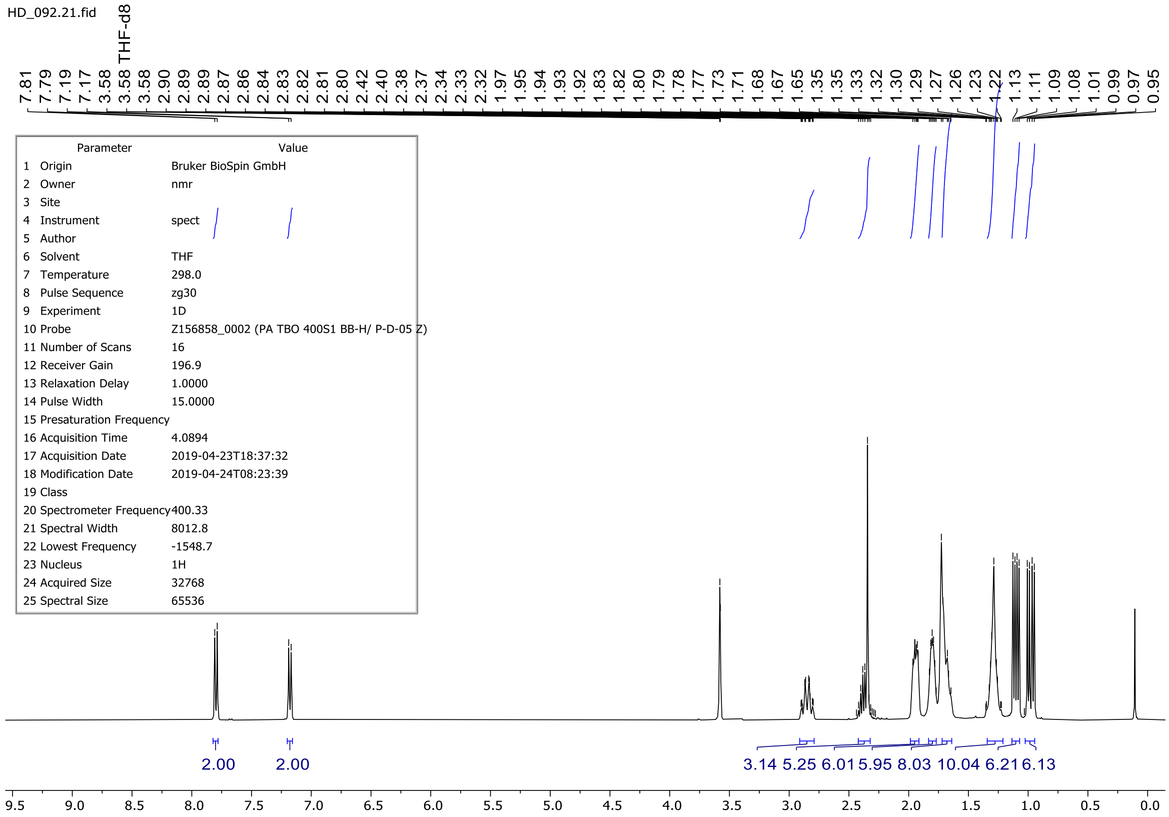

Figure 4. ^{1}H-NMR spectrum of **L4** in THF-d$_8$.

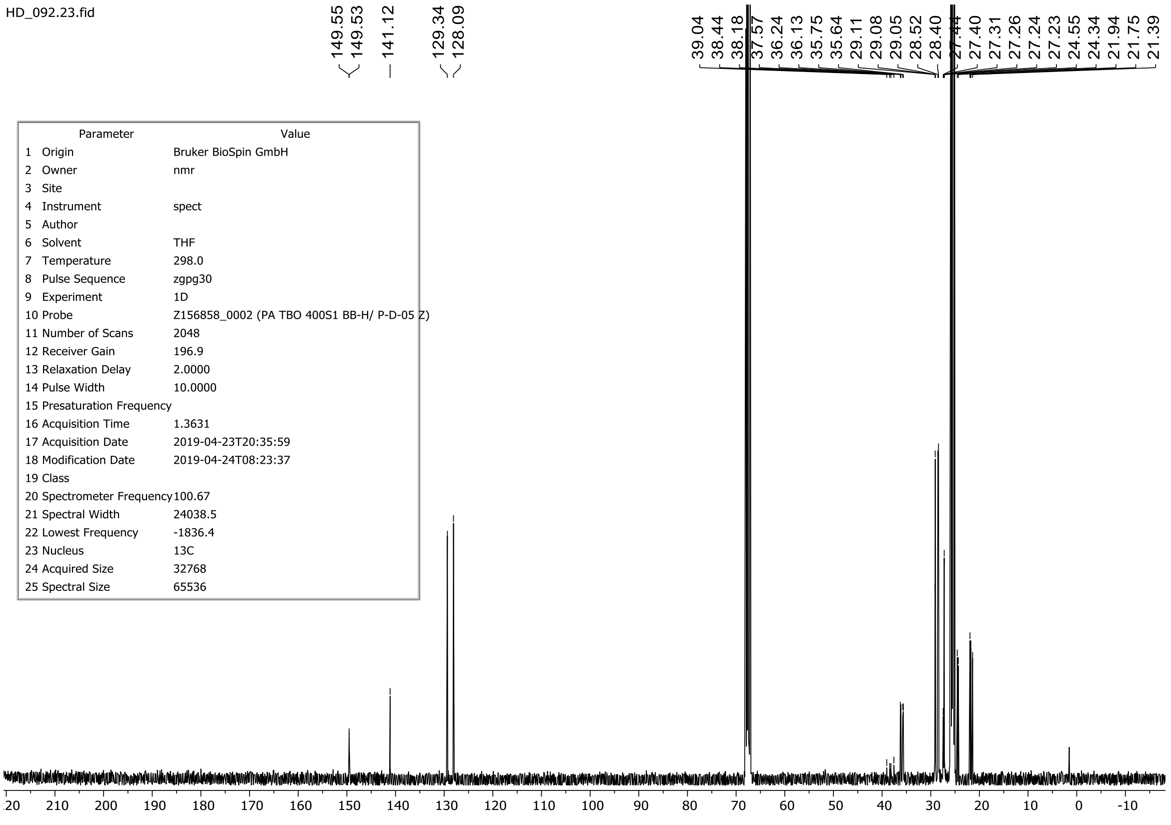

Figure 5. ^{13}C{^{1}H}-NMR spectrum of **L4** in THF-d$_8$.

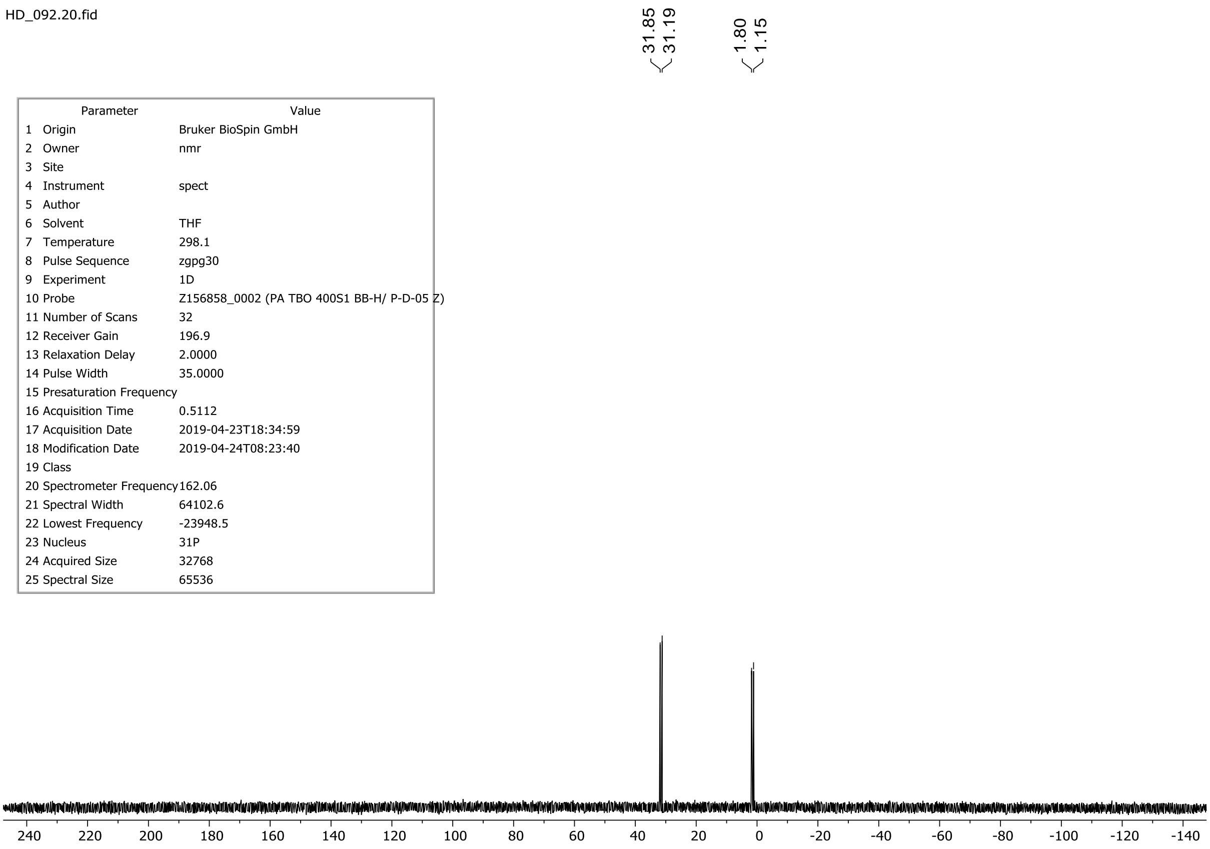

	Parameter	Value
1	Origin	Bruker BioSpin GmbH
2	Owner	nmr
3	Site	
4	Instrument	spect
5	Author	
6	Solvent	THF
7	Temperature	298.1
8	Pulse Sequence	zgpg30
9	Experiment	1D
10	Probe	Z156858_0002 (PA TBO 400S1 BB-H/ P-D-05 Z)
11	Number of Scans	32
12	Receiver Gain	196.9
13	Relaxation Delay	2.0000
14	Pulse Width	35.0000
15	Presaturation Frequency	
16	Acquisition Time	0.5112
17	Acquisition Date	2019-04-23T18:34:59
18	Modification Date	2019-04-24T08:23:40
19	Class	
20	Spectrometer Frequency	162.06
21	Spectral Width	64102.6
22	Lowest Frequency	-23948.5
23	Nucleus	31P
24	Acquired Size	32768
25	Spectral Size	65536

Figure 6. $^{31}P\{^{1}H\}$-NMR spectrum of **L4** in THF-d_8.

2.3 [Au(^{Cy}Y$_S$-PCy$_2$)Cl] (P1)

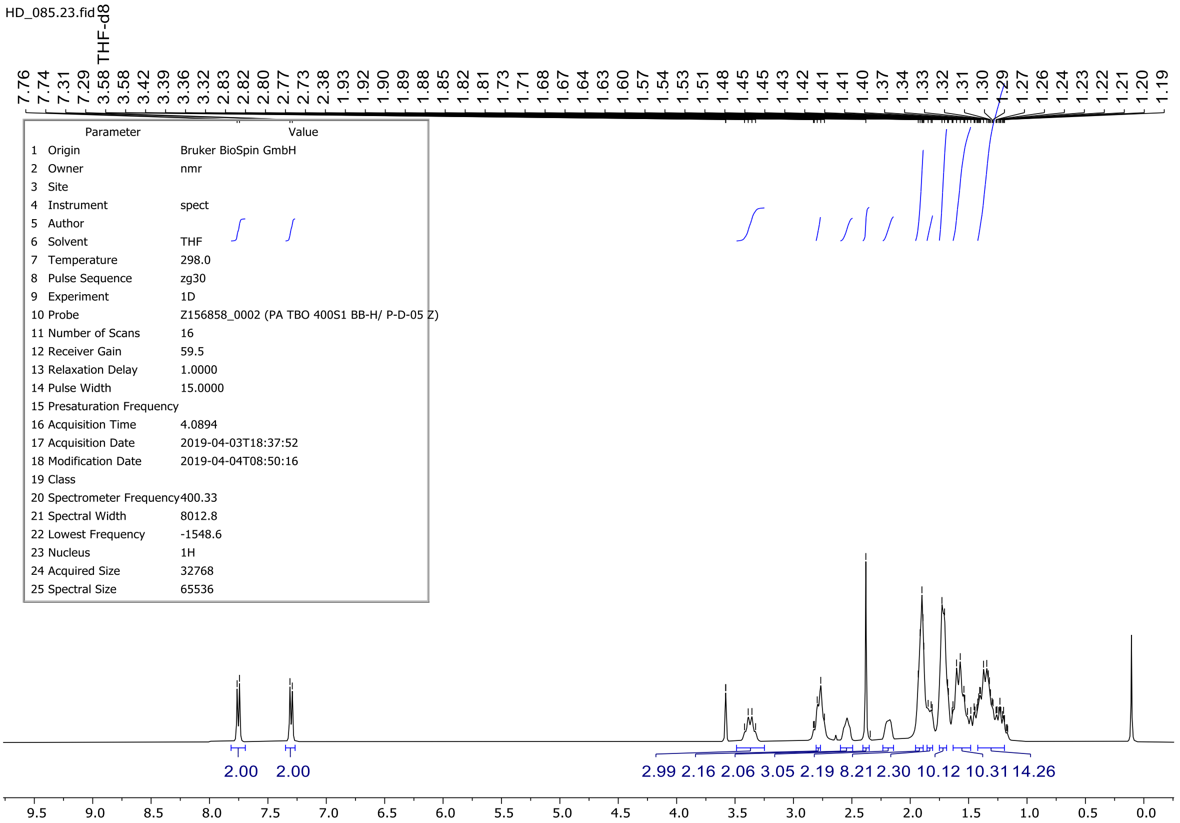

Figure 7. ^{1}H-NMR spectrum of **P1** in THF-d$_8$.

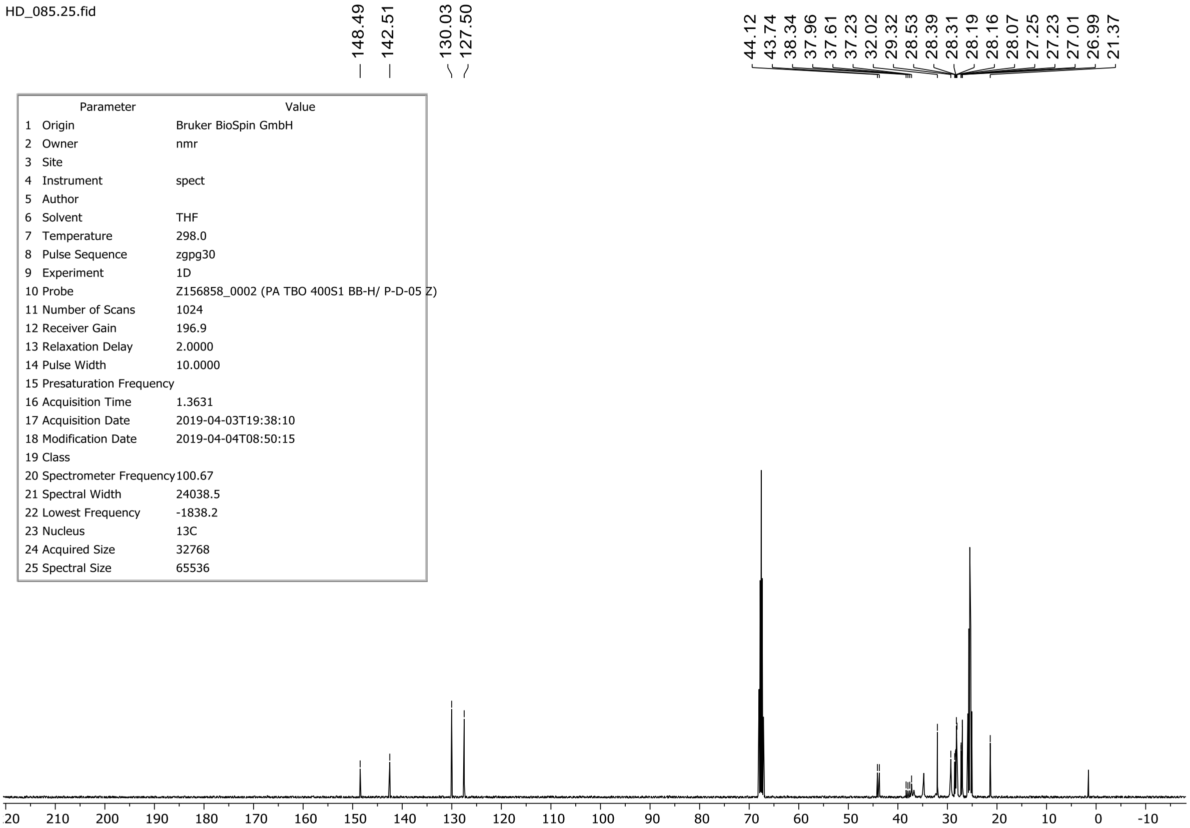

Figure 8. ^{13}C{^{1}H}-NMR spectrum of **P1** in THF-d$_8$.

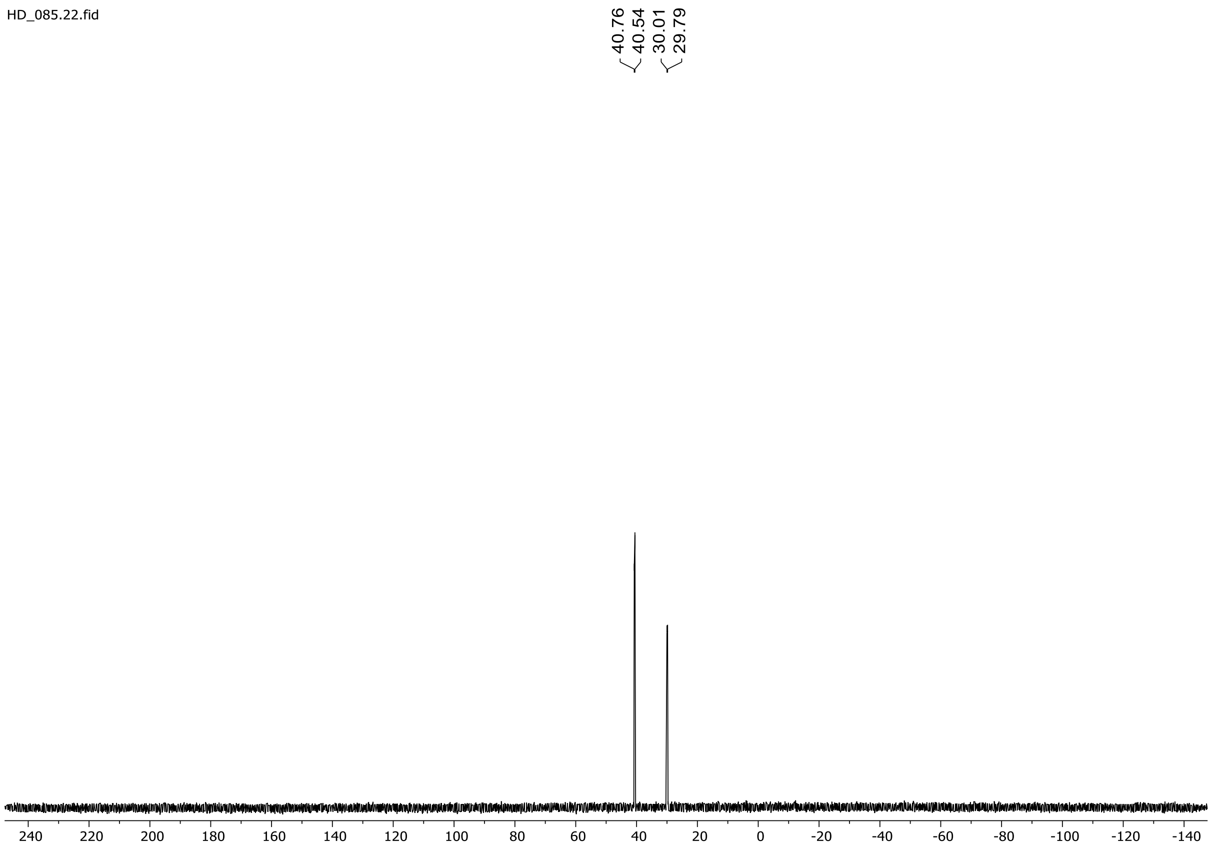

Figure 9. $^{31}P\{^{1}H\}$-NMR spectrum of **P1** in THF-d_8.

2.4 [Au($^{Cy}Y_S$-PiPr$_2$)Cl] (P4)

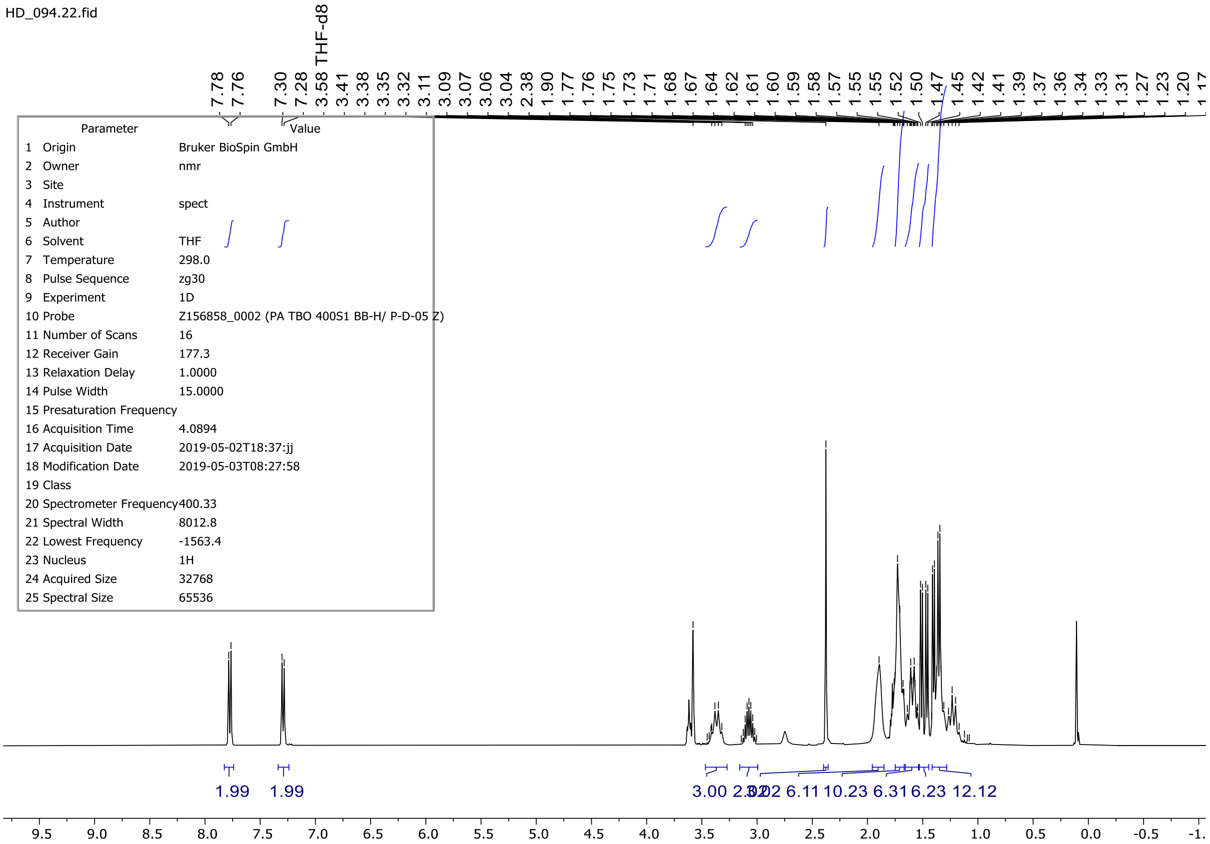

Figure 10. ^{1}H-NMR spectrum of **P4** in THF-d$_8$.

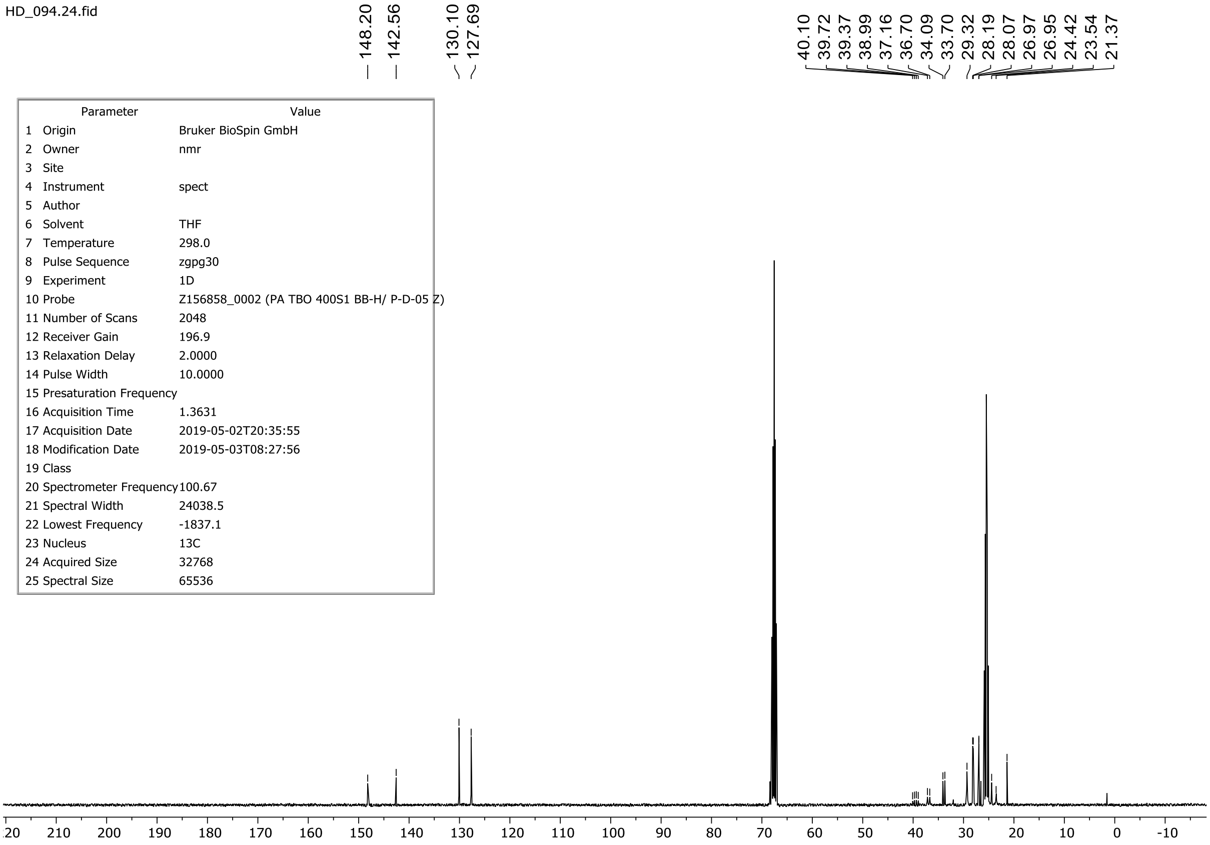

Figure 11. ^{13}C{^{1}H}-NMR spectrum of **P4** in THF-d$_8$.

HD_094.20.fid

	Parameter	Value
1	Origin	Bruker BioSpin GmbH
2	Owner	nmr
3	Site	
4	Instrument	spect
5	Author	
6	Solvent	THF
7	Temperature	298.1
8	Pulse Sequence	zgpg30
9	Experiment	1D
10	Probe	Z156858_0002 (PA TBO 400S1 BB-H/ P-D-05 Z)
11	Number of Scans	32
12	Receiver Gain	196.9
13	Relaxation Delay	2.0000
14	Pulse Width	35.0000
15	Presaturation Frequency	
16	Acquisition Time	0.5112
17	Acquisition Date	2019-05-02T18:34:56
18	Modification Date	2019-05-03T08:27:59
19	Class	
20	Spectrometer Frequency	162.06
21	Spectral Width	64102.6
22	Lowest Frequency	-23948.5
23	Nucleus	31P
24	Acquired Size	32768
25	Spectral Size	65536

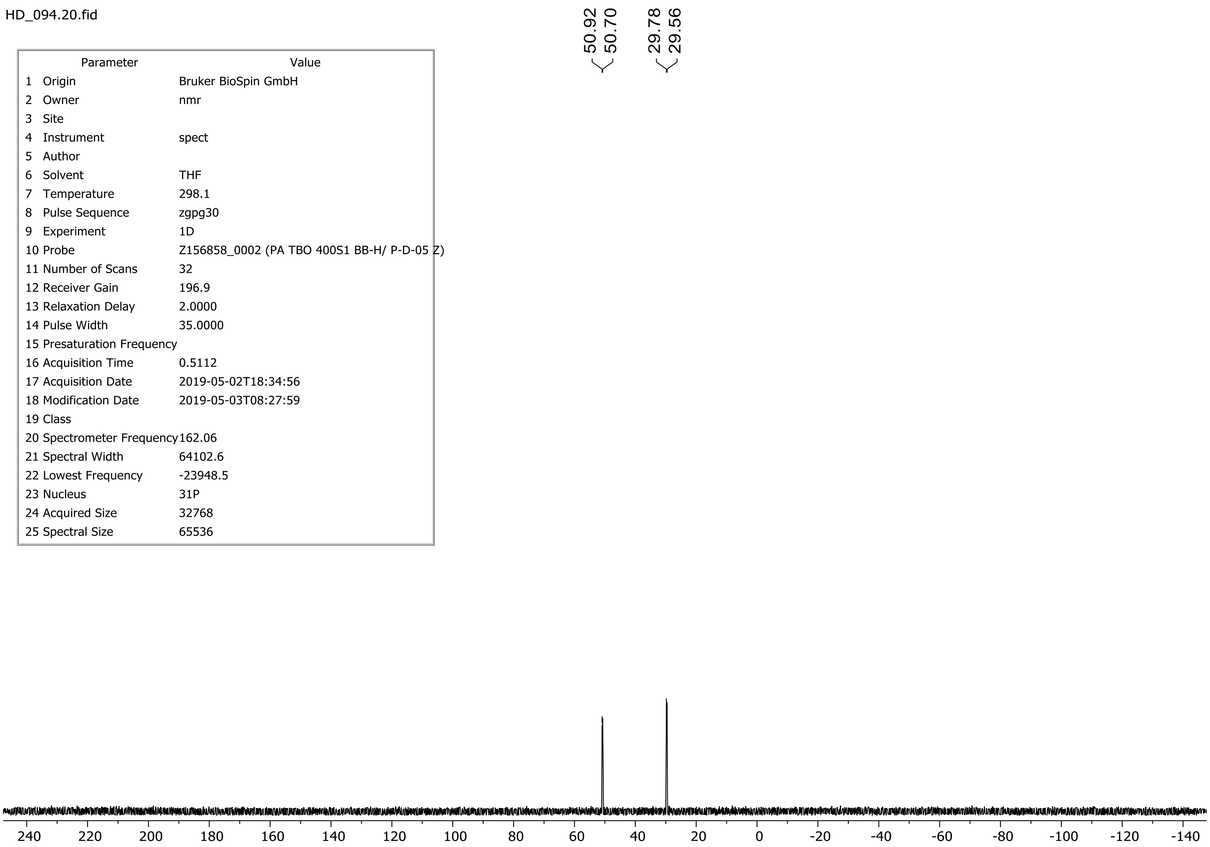

Figure 12. $^{31}P\{^1H\}$-NMR spectrum of **P4** in THF-d_8.

2.5 ^{Cy}**Y**$_{SF}$**-H**

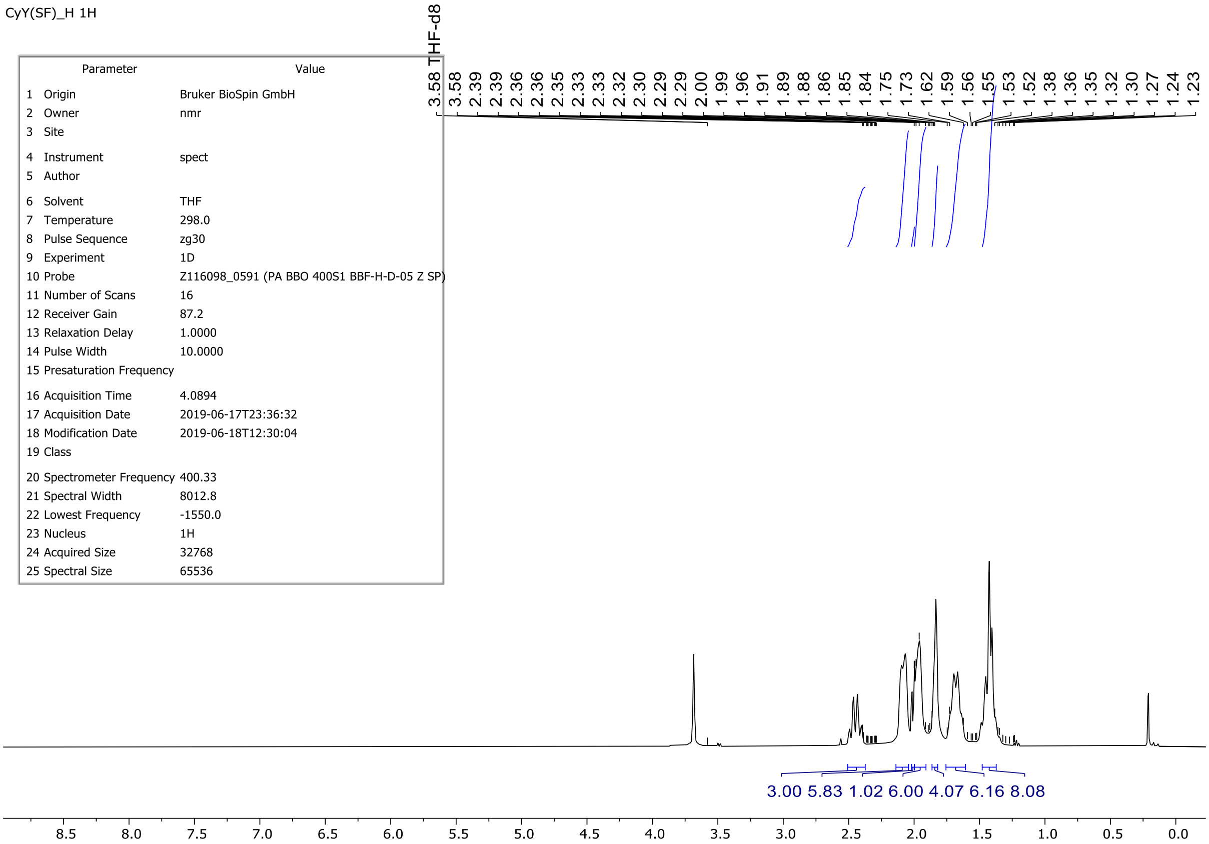

Figure 13. ^{1}H-NMR spectrum of ^{Cy}**Y**$_{SF}$**-H** in THF-d$_8$.

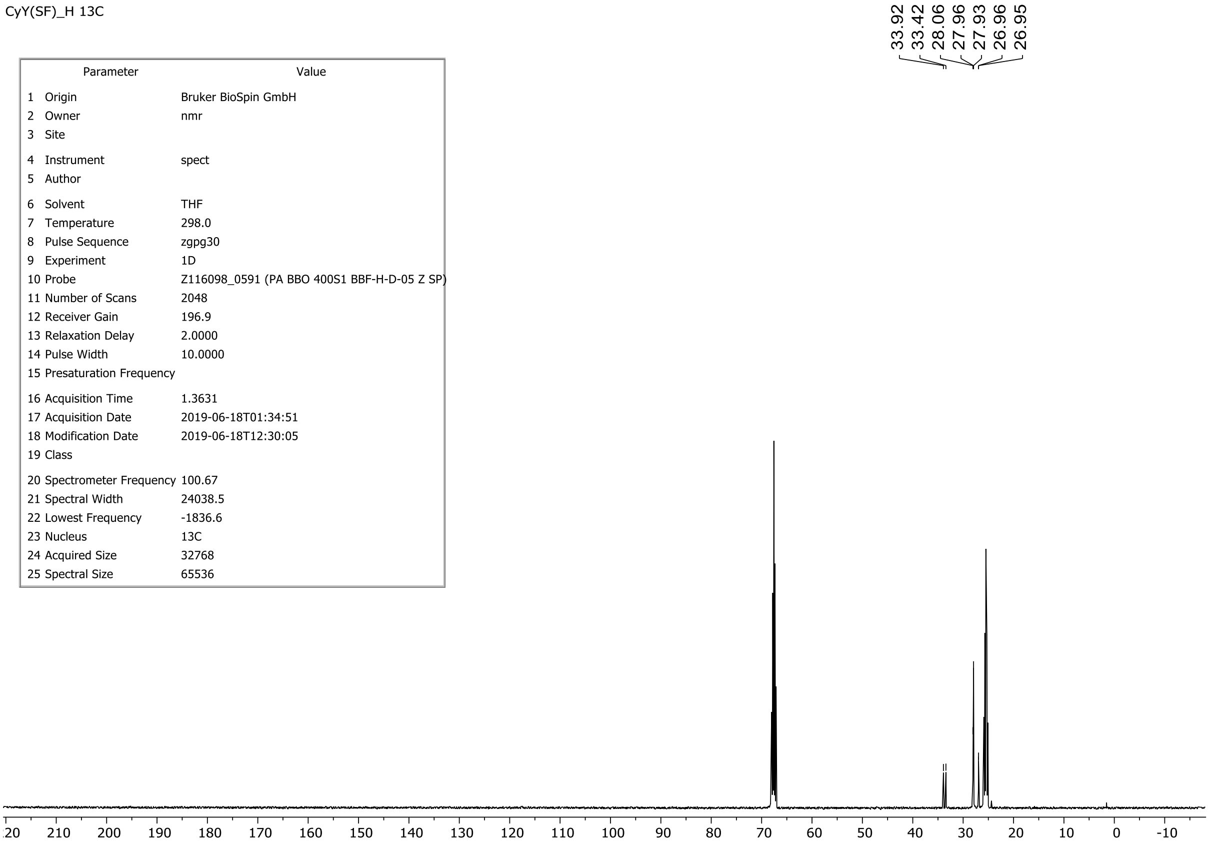

Figure 14. ^{13}C{^{1}H}-NMR spectrum of ^{Cy}**Y**$_{SF}$**-H** in THF-d$_8$.

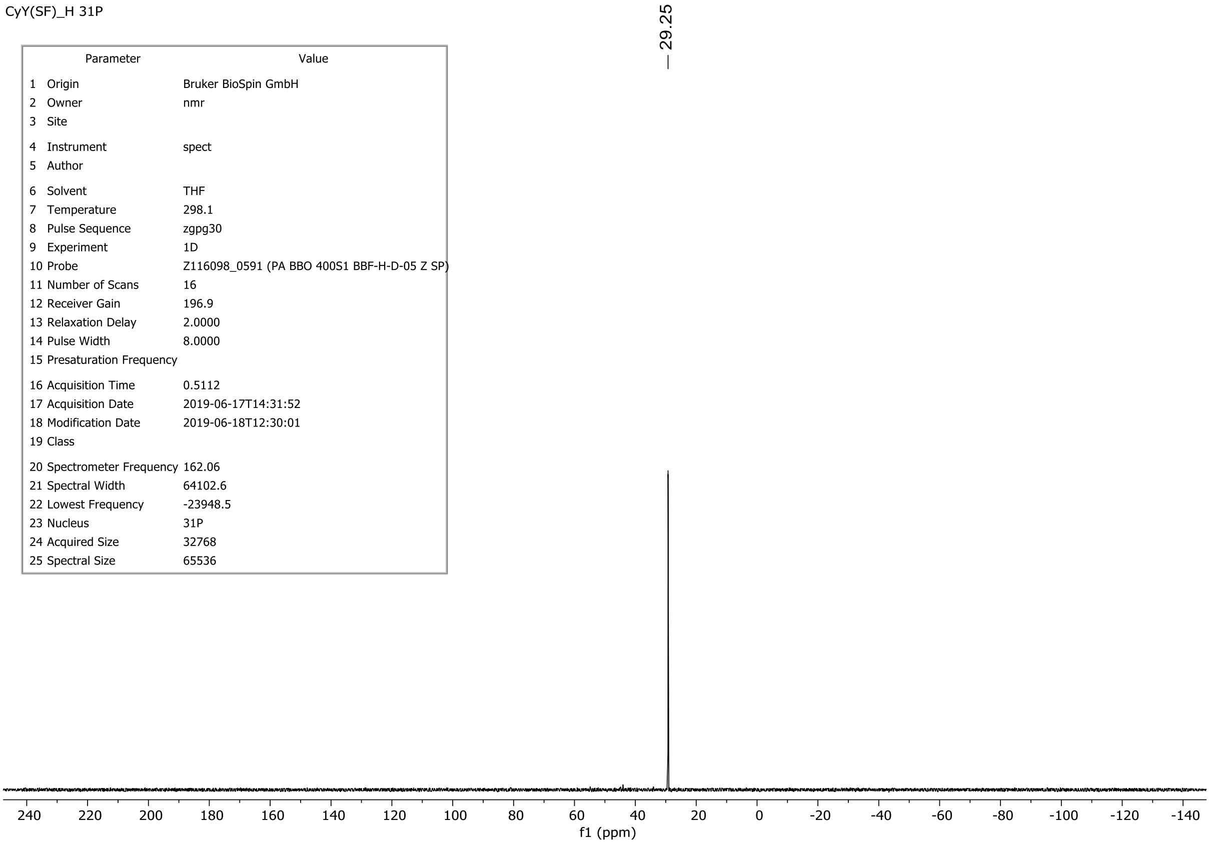

Figure 15. $^{31}P\{^{1}H\}$-NMR spectrum of **$^{Cy}Y_{SF}$-H** in THF-d_8.

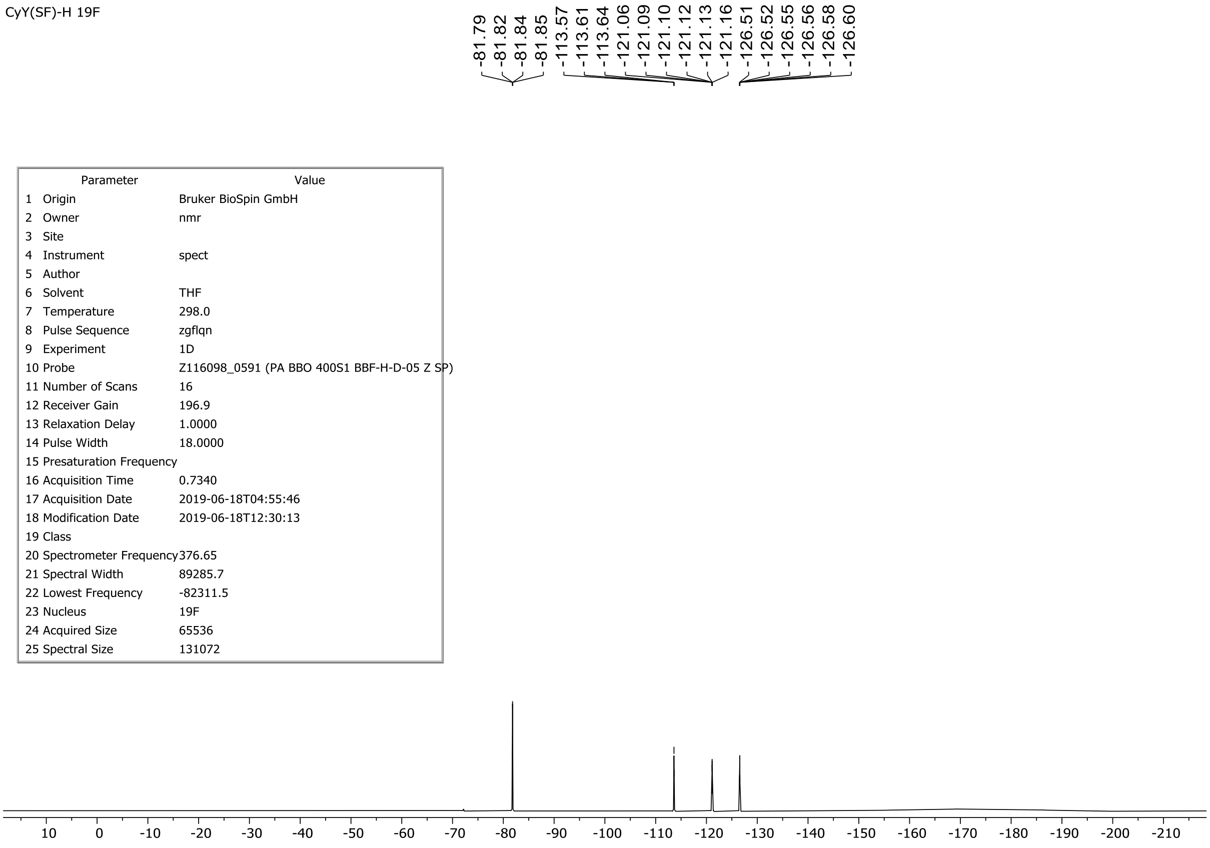

Figure 16. $^{19}F\{^{1}H\}$-NMR spectrum of **$^{Cy}Y_{SF}$-H** in THF-d_8.

2.6 $^{Cy}Y_{SF}$-PCy_2 (L5)

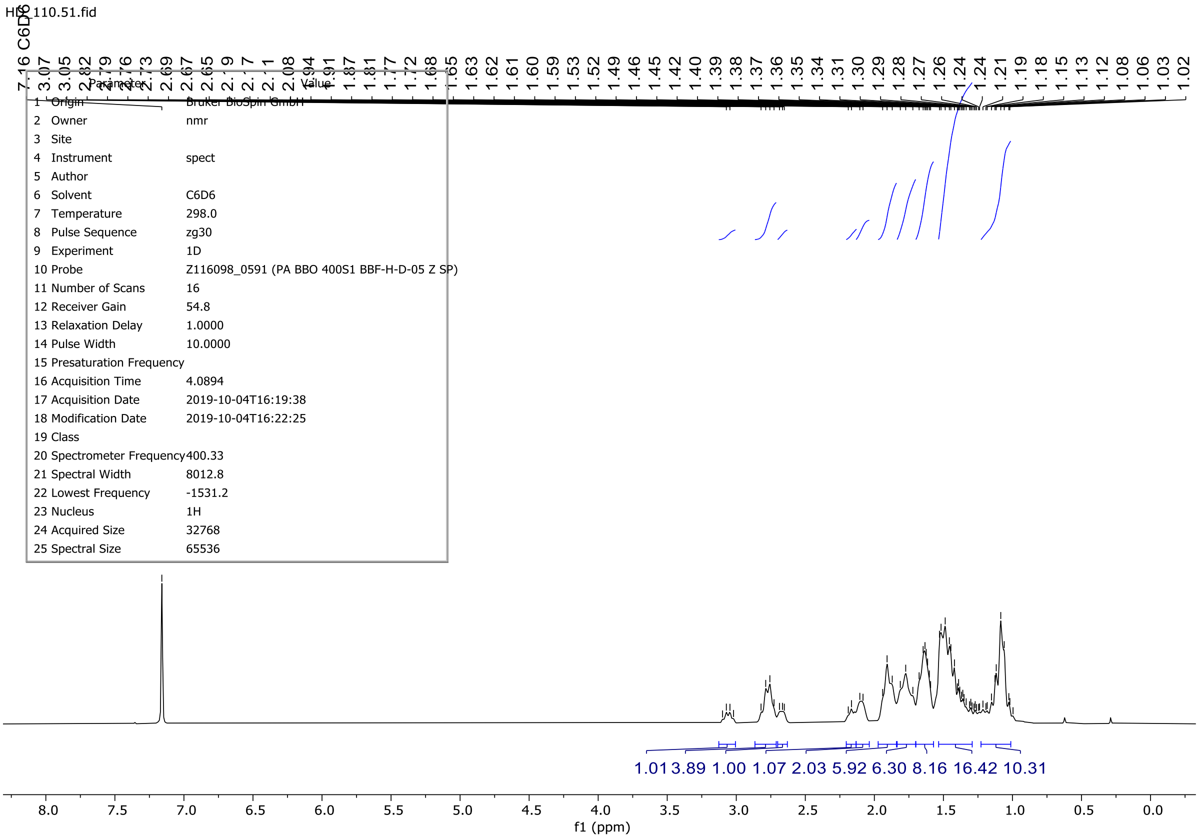

Figure 17. ^{1}H-NMR spectrum of **L5** in C_6D_6.

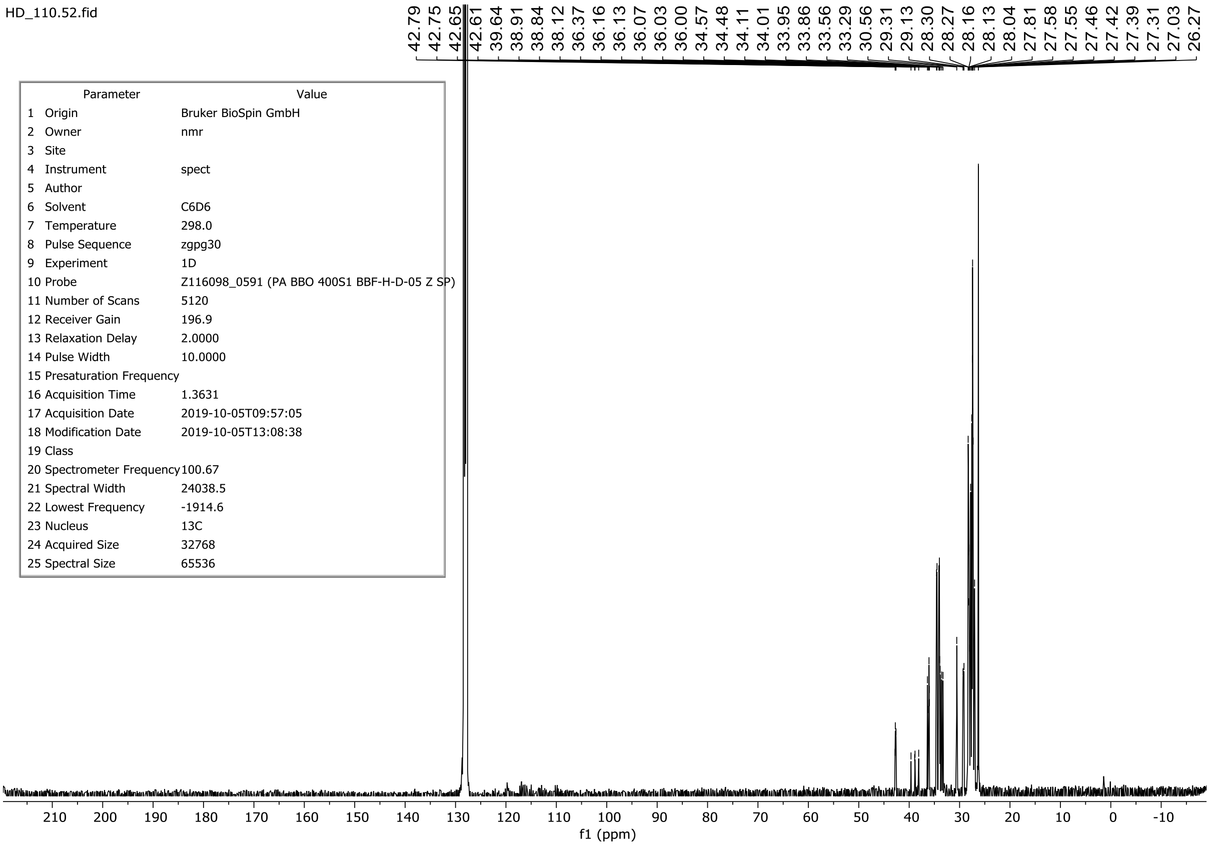

Figure 18. $^{13}C\{^1H\}$-NMR spectrum of **L5** in C_6D_6.

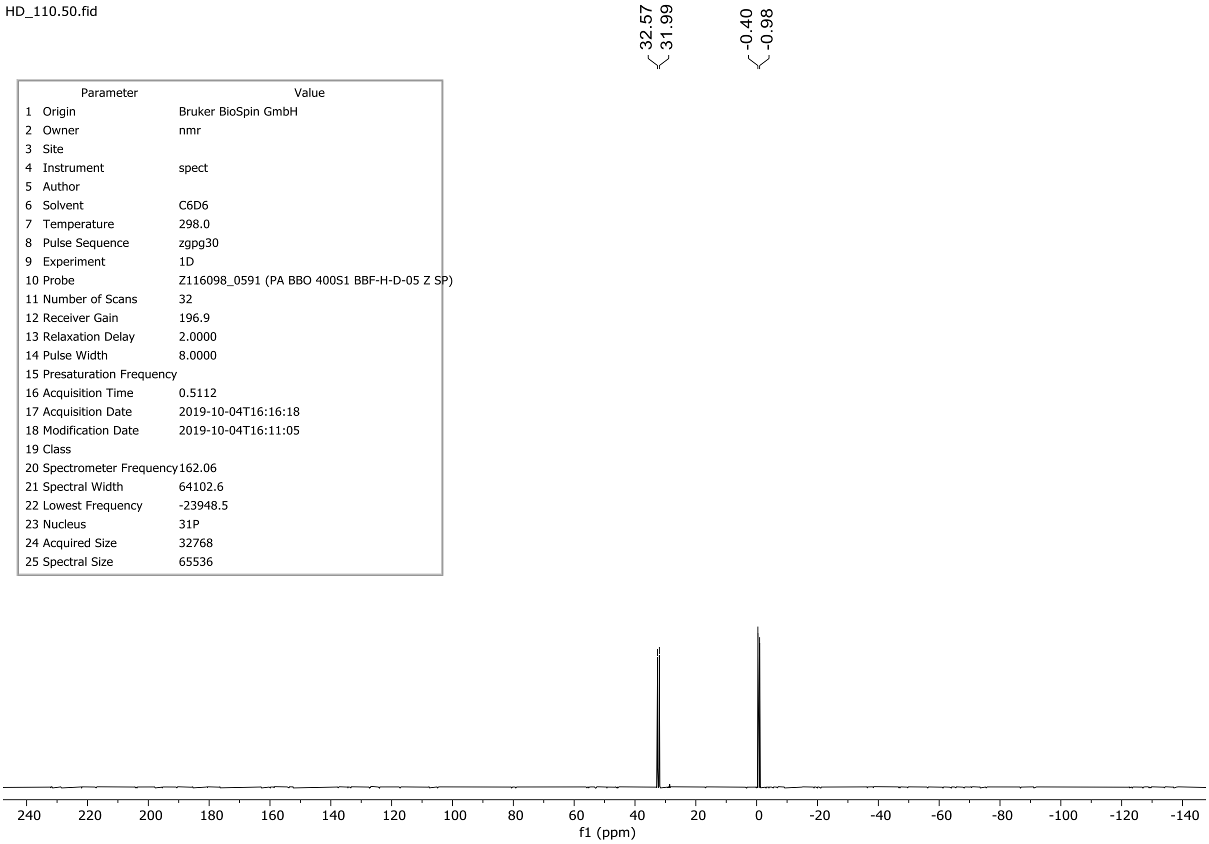

Figure 19. $^{31}P\{^{1}H\}$-NMR spectrum of **L5** in C_6D_6.

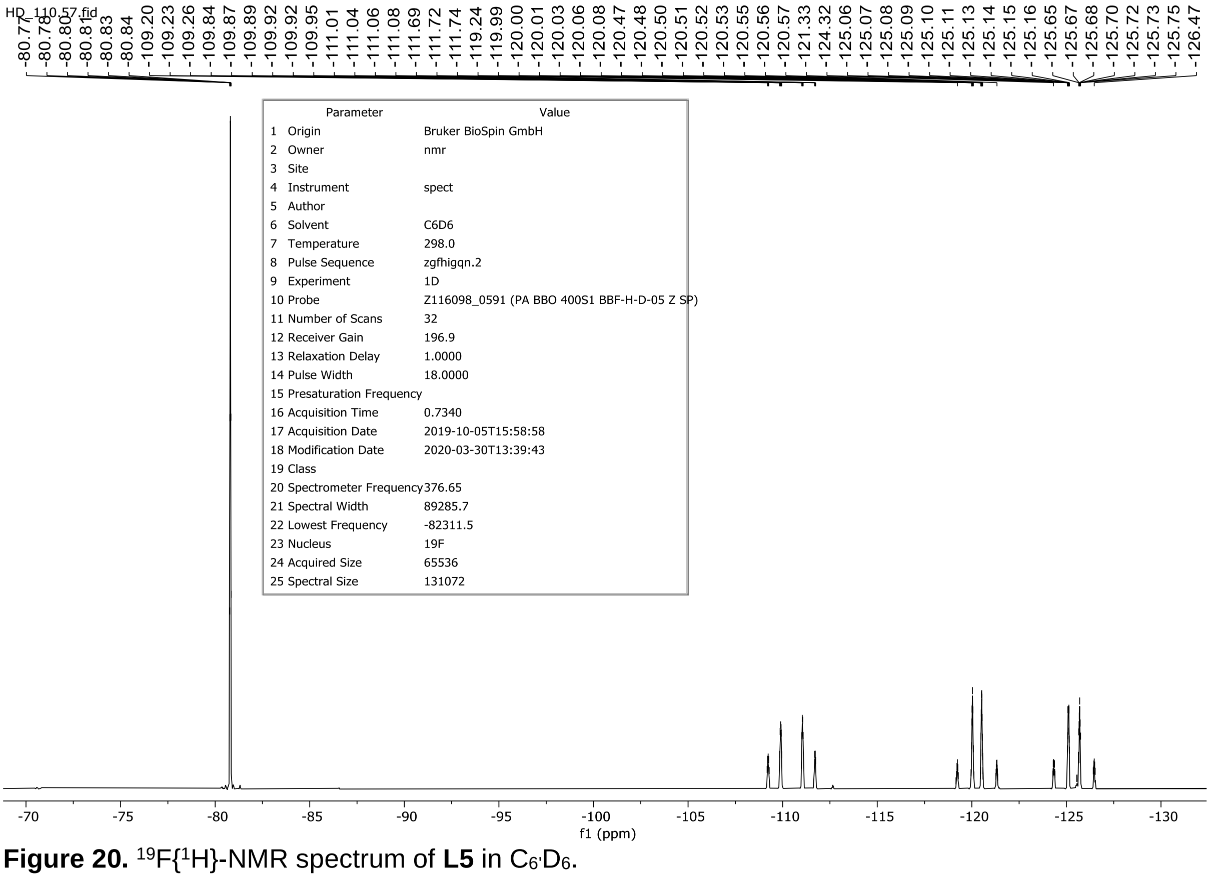

Figure 20. $^{19}F\{^{1}H\}$-NMR spectrum of **L5** in C_6D_6.

2.7 [Au(^{Cy}Y$_{SF}$-PCy$_2$)Cl] (P5)

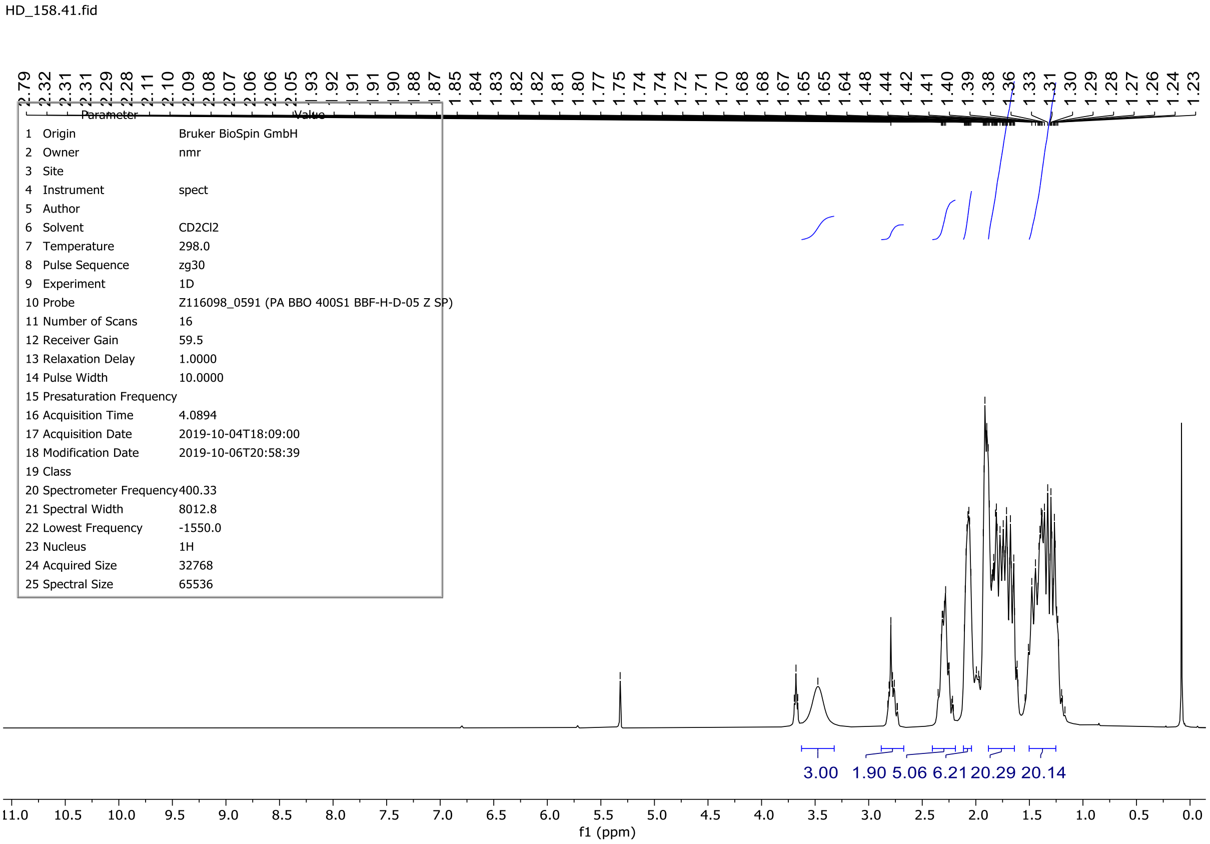

Figure 21. ^{1}H-NMR spectrum of **P5** in CD_2Cl_2.

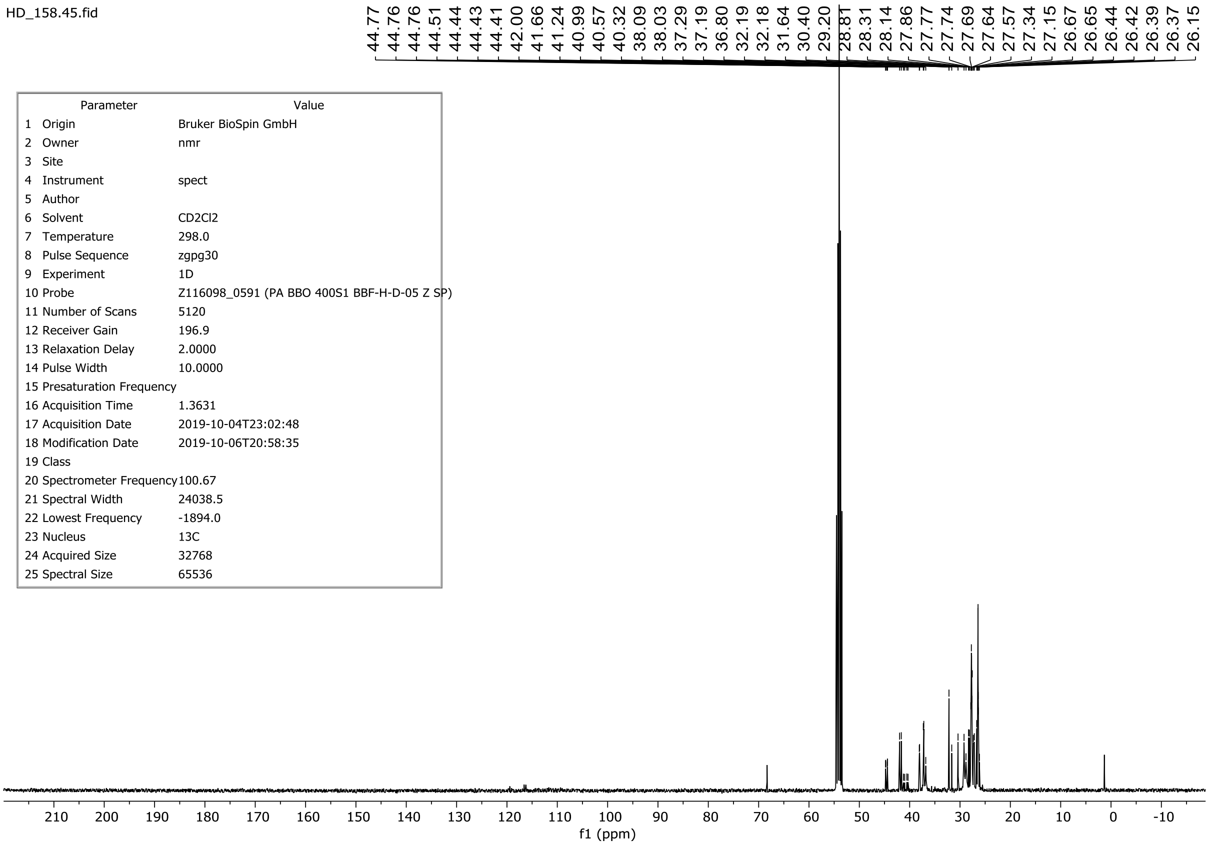

Figure 22. ^{13}C{^{1}H}-NMR spectrum of **P5** in CD_2Cl_2.

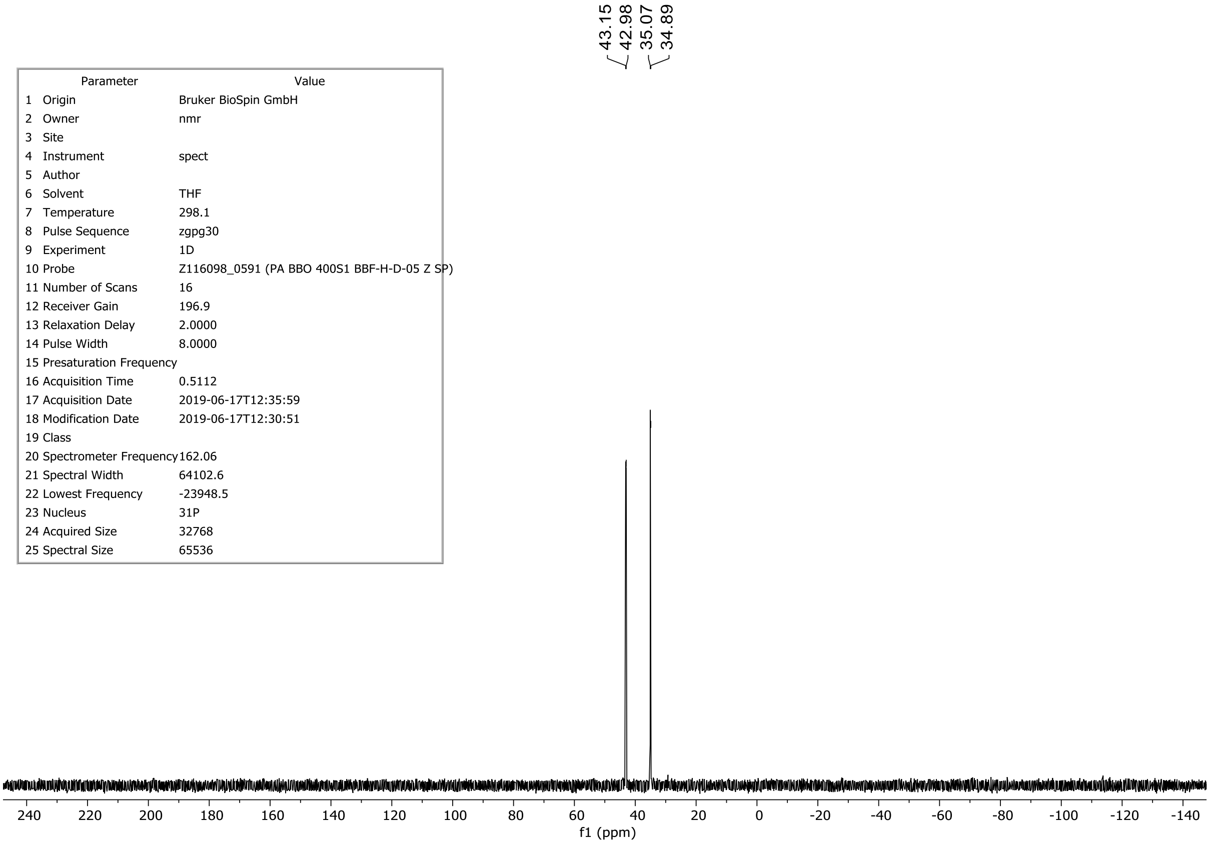

Figure 23. $^{31}P\{^1H\}$-NMR spectrum of **P5** in THF-d$_8$.

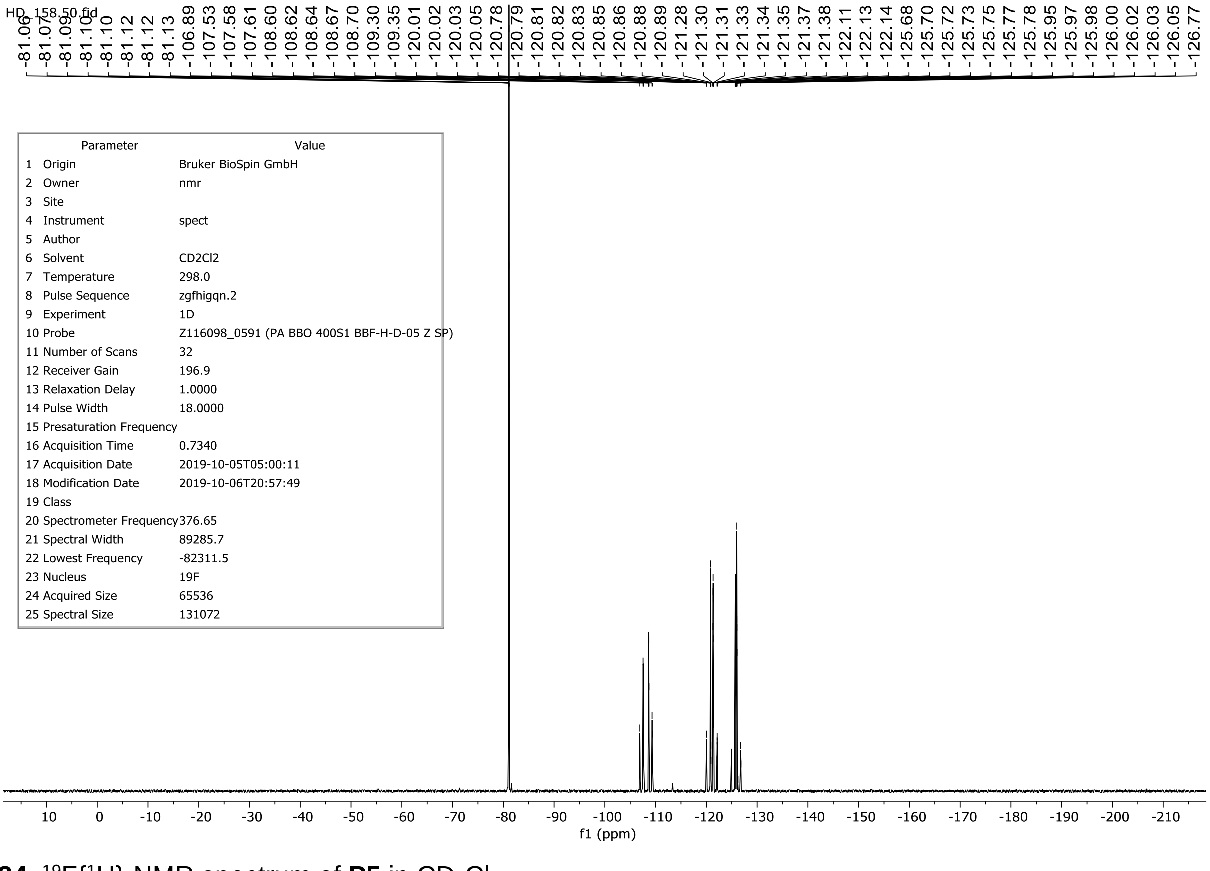

Figure 24. $^{19}F\{^1H\}$-NMR spectrum of **P5** in CD_2Cl_2.

2.8 VT-NMR of P1 and P5

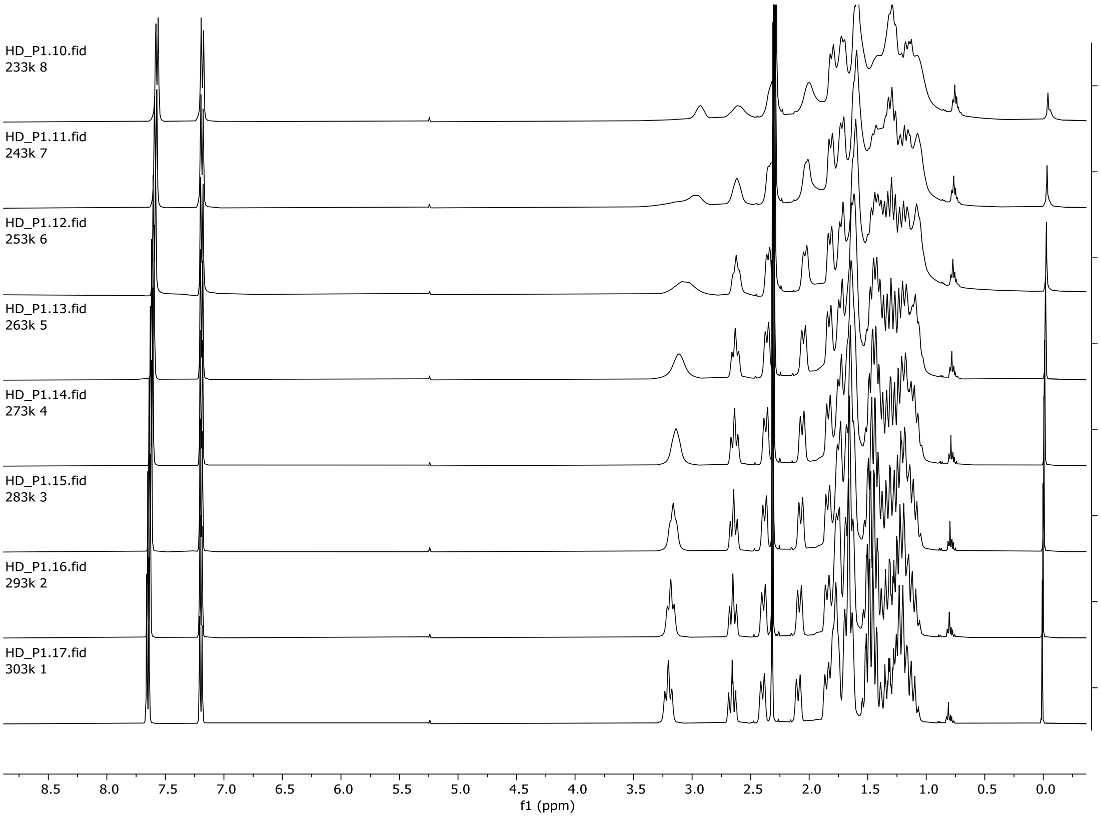

Figure 25. $^{1}H\{^{31}P\}$-NMR of **P1** at different temperatures. Starting from 303K (bottom) to 243K (top) in 10K steps.

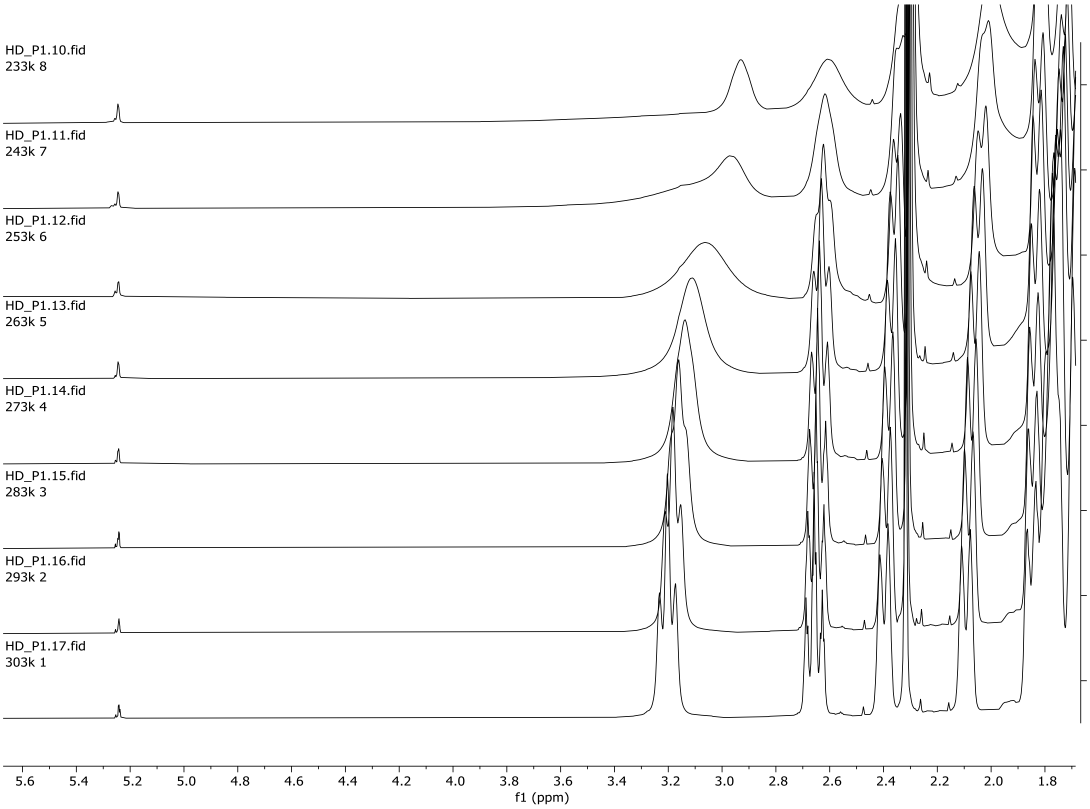

Figure 26. $^{1}H\{^{31}P\}$-NMR of **P1** at different temperatures. Starting from 303K (bottom) to 243K (top) in 10K steps. Zoomed into the region of the respective C-H signals.

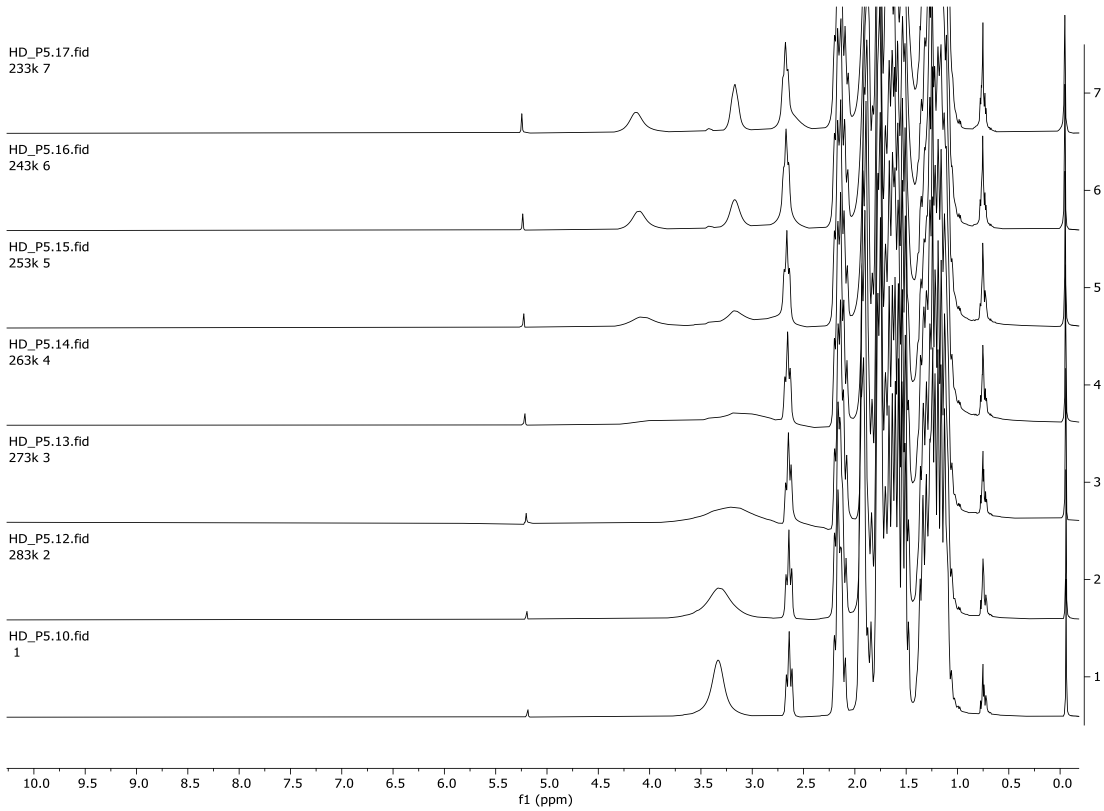

Figure 27. $^{1}H\{^{31}P\}$-NMR of **P5** at different temperatures. Starting from 303K (bottom) to 243K (top) in 10K steps.

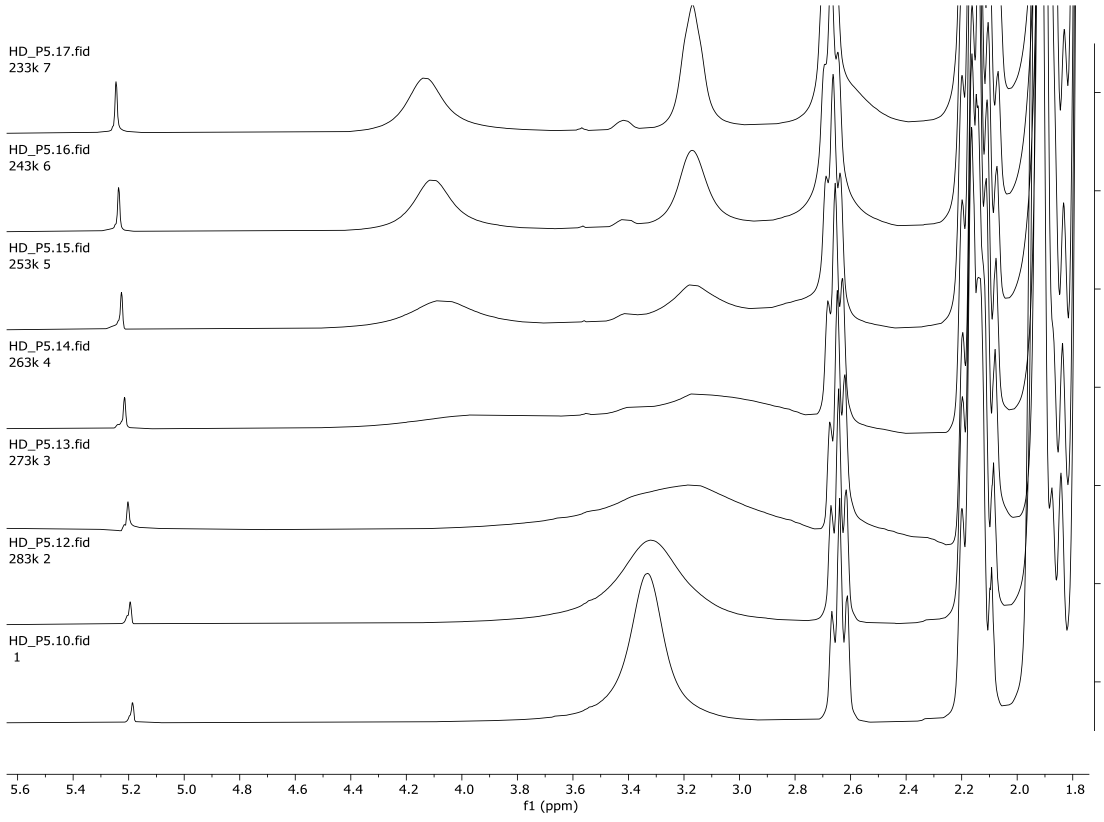

Figure 28a. $^{1}H\{^{31}P\}$-NMR of **P5** in DCM at different temperatures. Starting from 303K (bottom) to 243K (top) in 10K steps. Zoomed into the region of the respective C-H signals.

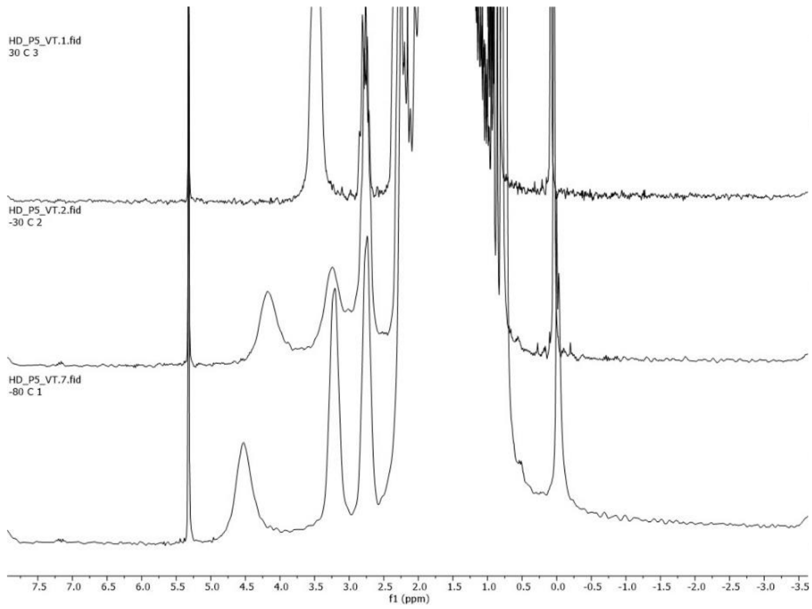

Figure 28b. Extract of the $^{1}H\{^{31}P\}$-NMR of **P5** in DCM at 30 °C (top), -30 °C (middle) and -80 °C (bottom).

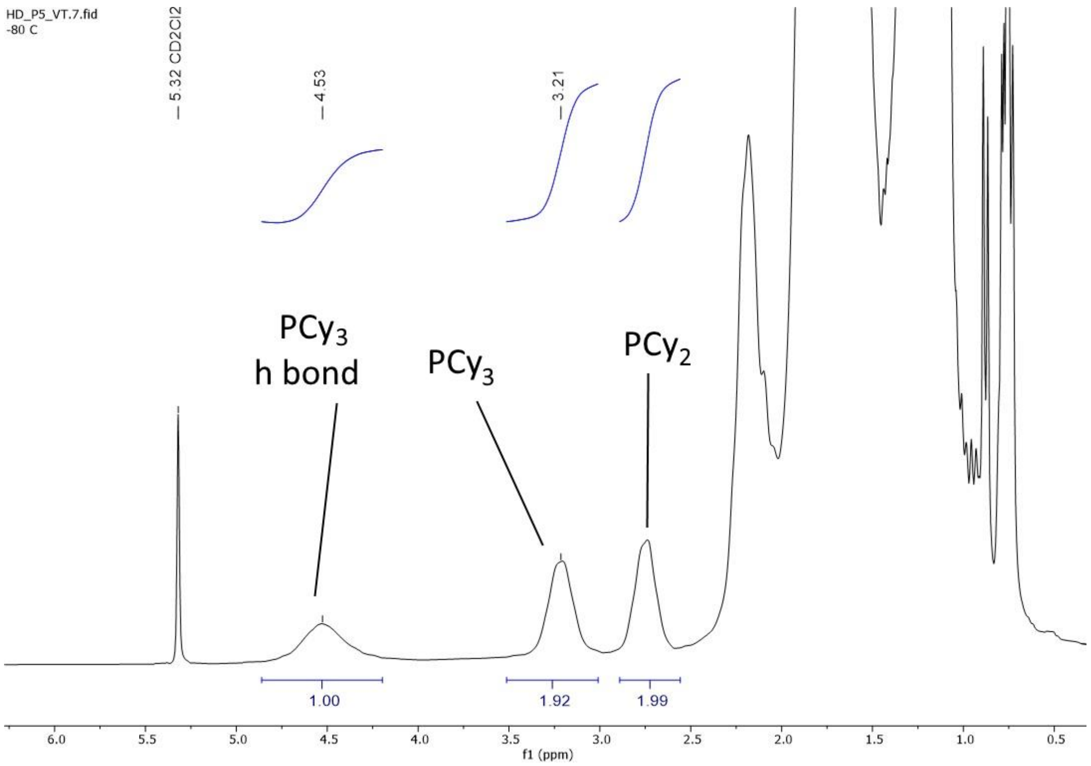

Figure 28c. Extract of the $^{1}H\{^{31}P\}$-NMR of **P5** in DCM at -80 °C showing the PC*H* signals.

2.9 [Au(^{Ph}Y$_S$-PCy$_2$)(4-CH$_3$O-C$_6$H$_4$)] (1a)

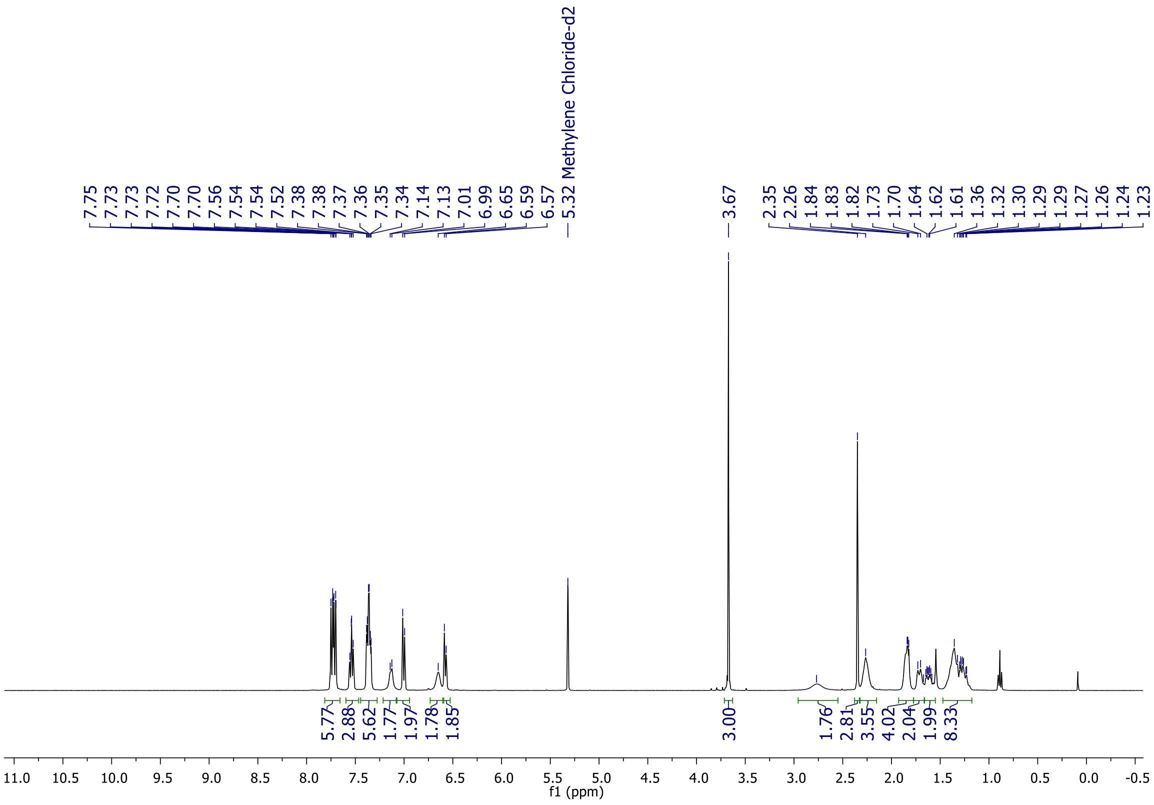

Figure 29. ^{1}H-NMR spectrum of **1a** in CD_2Cl_2.

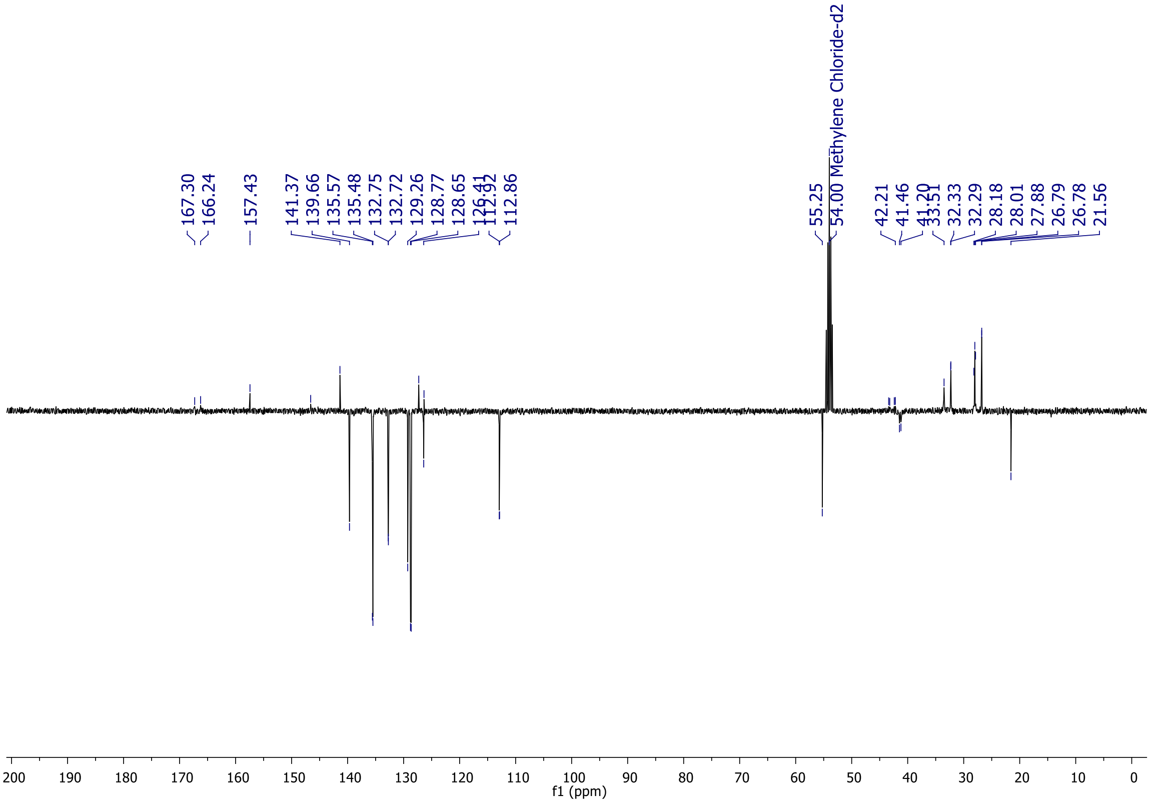

Figure 30. ^{13}C{^{1}H}-NMR spectrum of **1a** in CD_2Cl_2.

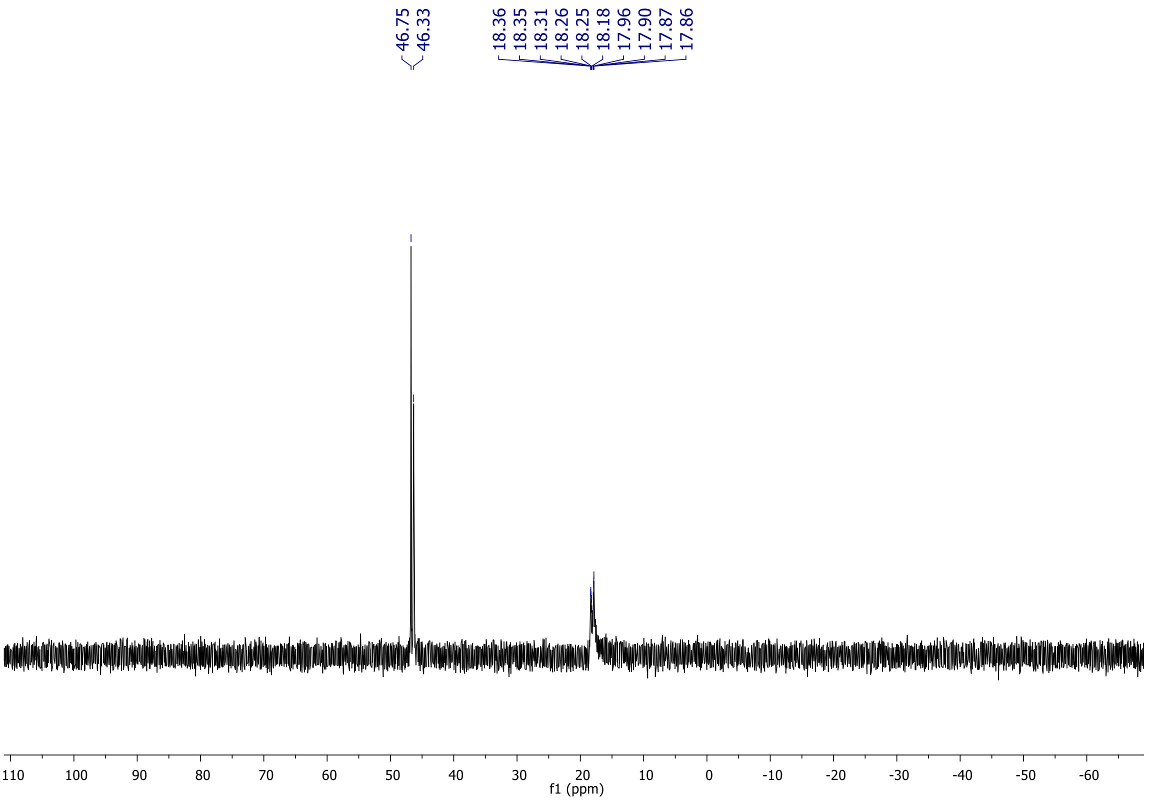

Figure 31. $^{31}P\{^{1}H\}$-NMR spectrum **1a** in CD_2Cl_2.

2.10 [Au($^{Cy}Y_S$-PCy_2)(4-CH_3O-C_6H_4)] (1b)

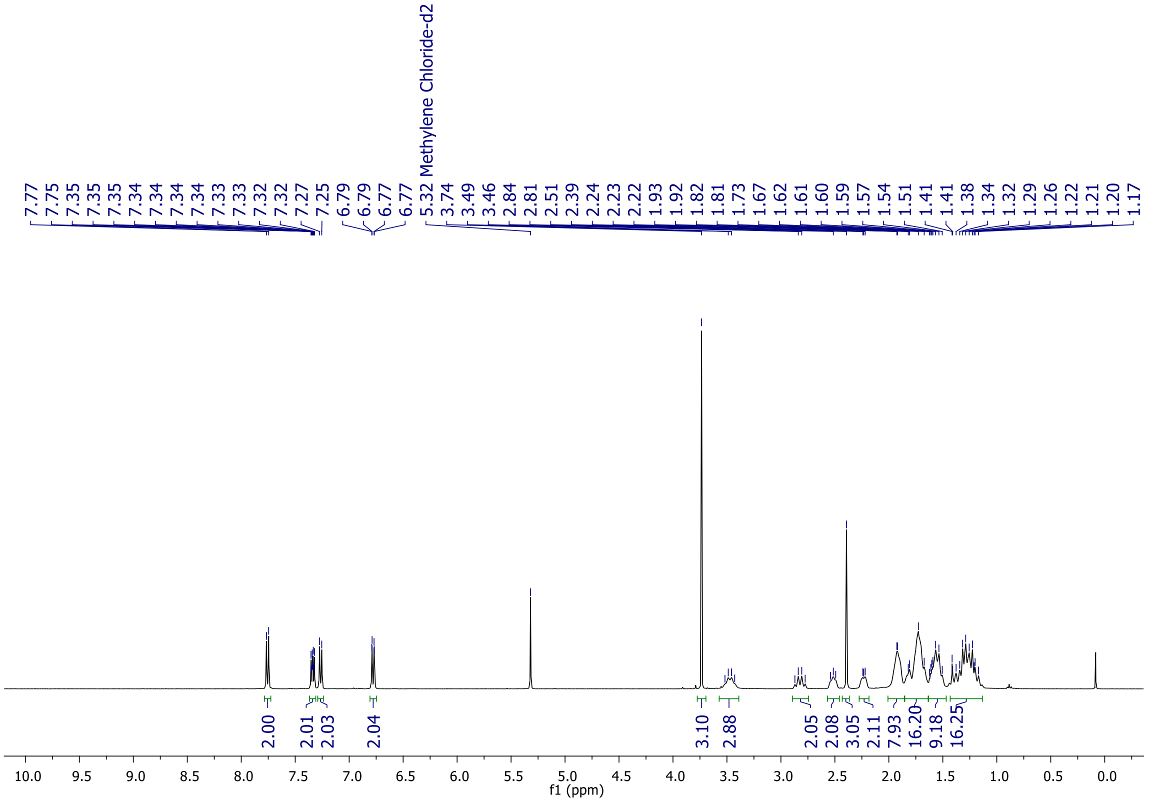

Figure 32. ^{1}H-NMR spectrum of **1b** in CD_2Cl_2.

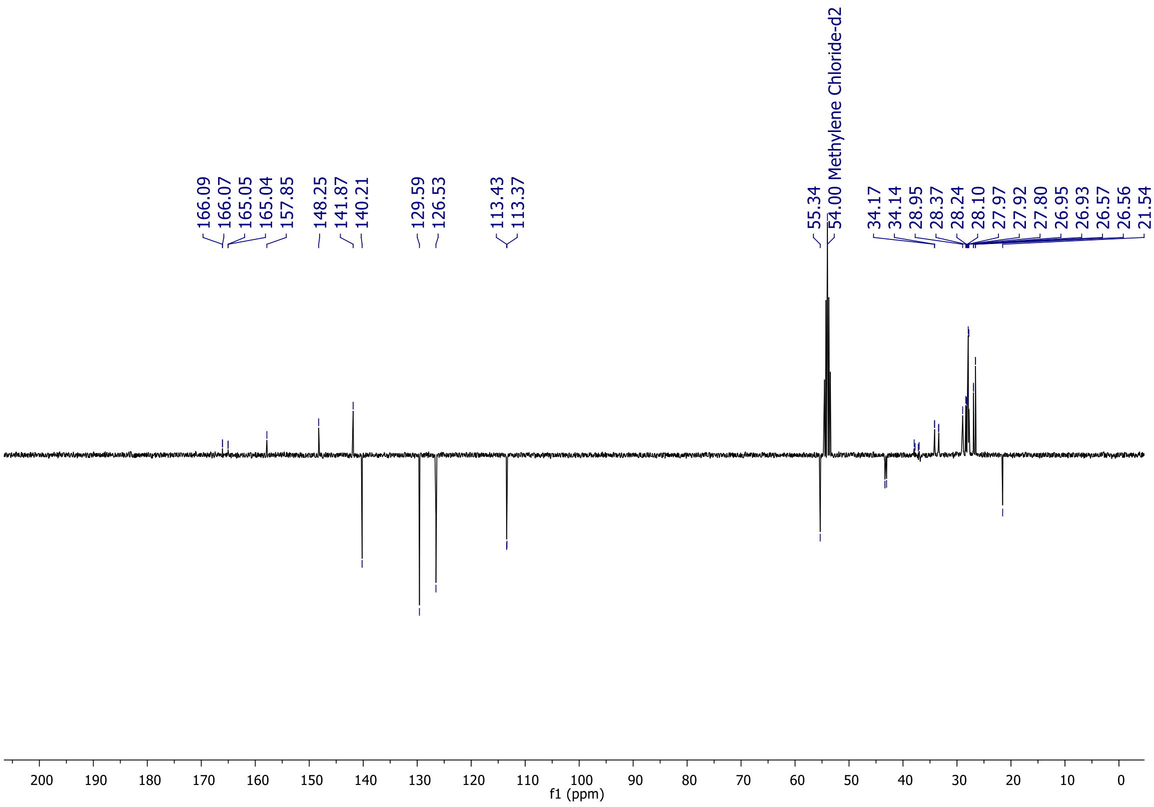

Figure 33. $^{13}C\{^{1}H\}$-NMR spectrum of **1b** in CD_2Cl_2.

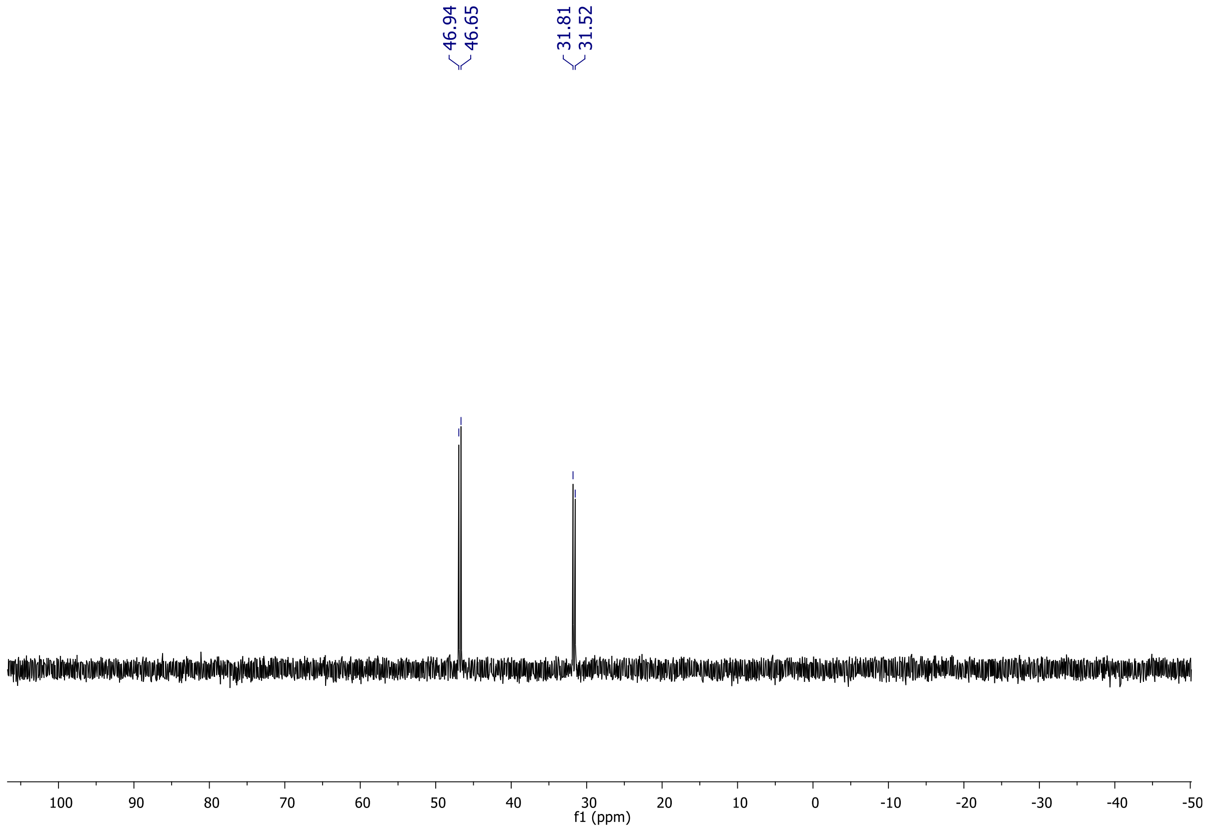

Figure 34. $^{31}P\{^{1}H\}$-NMR spectrum **1b** in CD_2Cl_2.

2.11 [{Au(^{Ph}Y$_S$PCy$_2$)}$_2$(μ-OH)][BF$_4$] (2a)

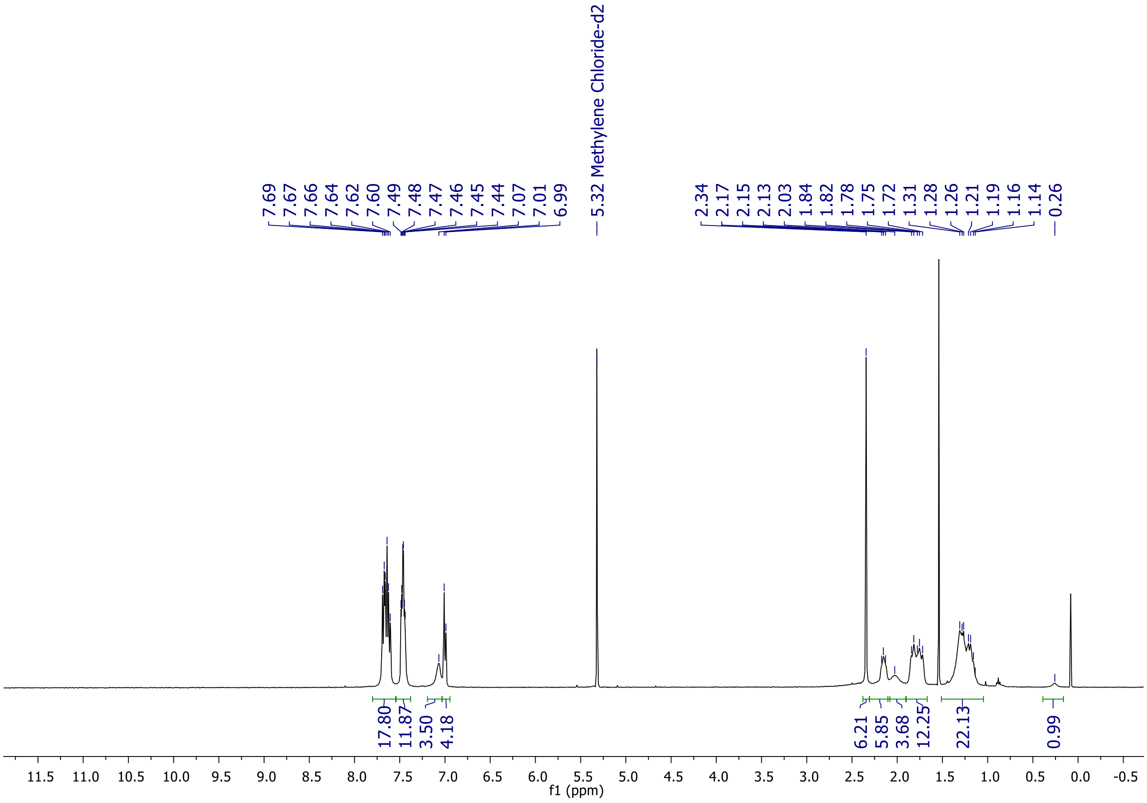

Figure 35. ^{1}H-NMR spectrum of **2a** in CD_2Cl_2.

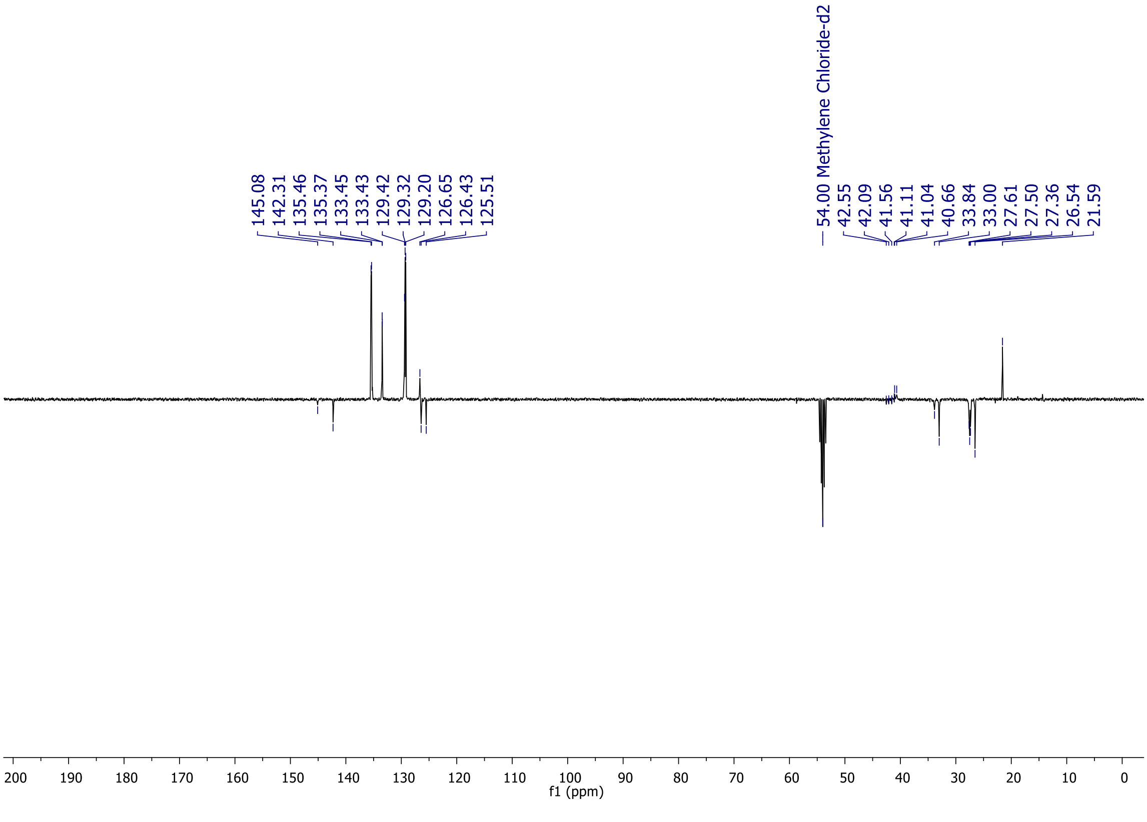

Figure 36. ^{13}C{^{1}H}-NMR spectrum of **2a** in CD_2Cl_2.

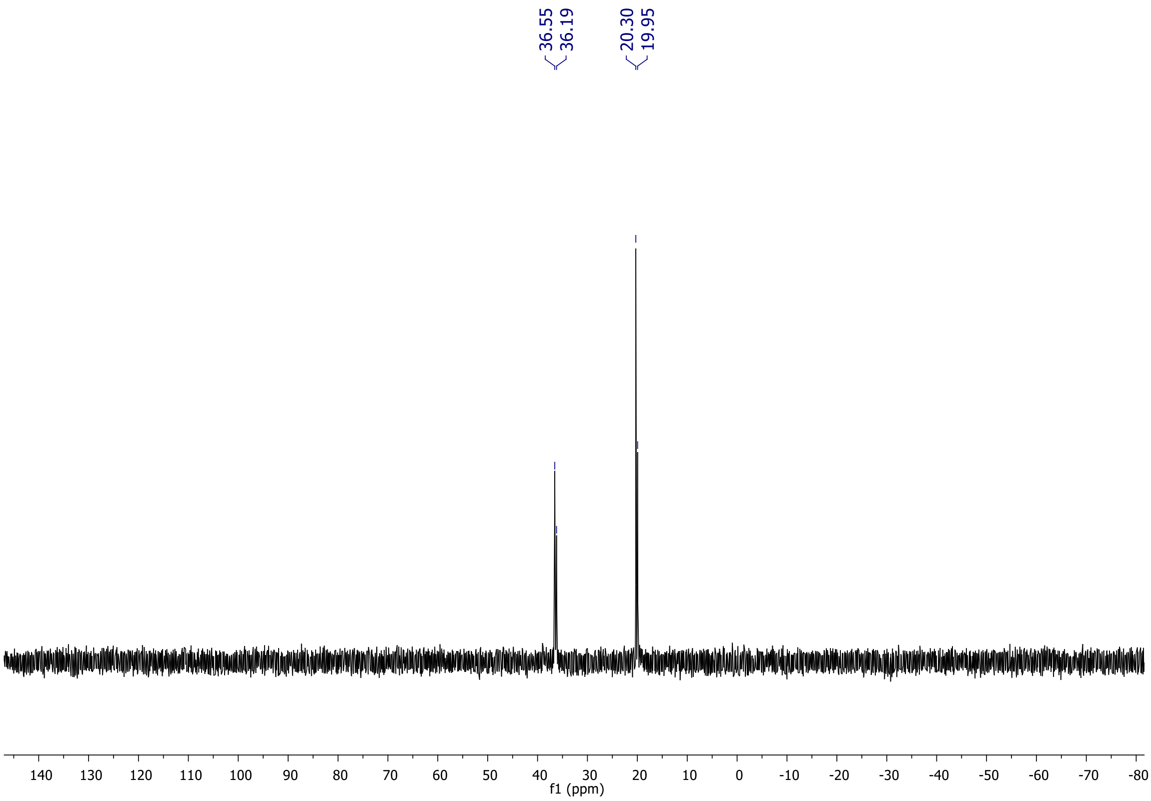

Figure 37. $^{31}P\{^1H\}$-NMR spectrum **2a** in CD_2Cl_2.

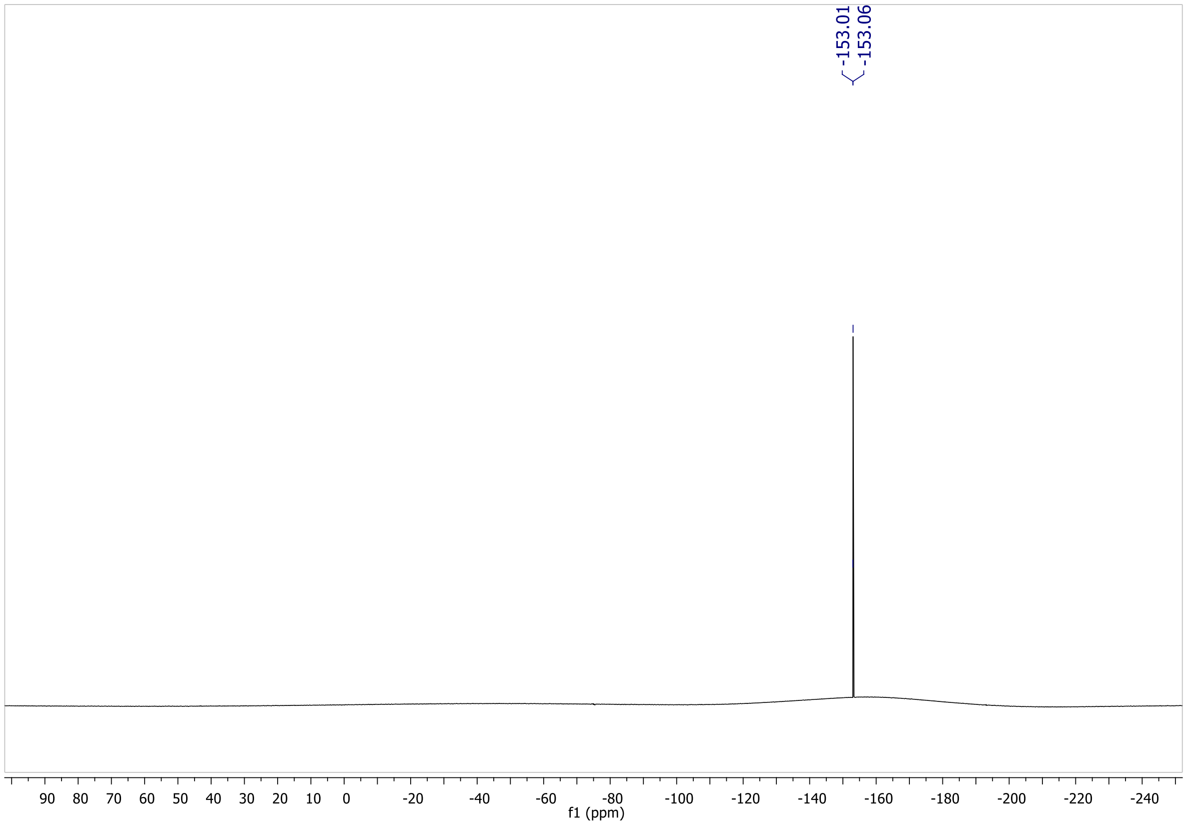

Figure 38. $^{19}F\{^1H\}$-NMR spectrum of **2a** in CD_2Cl_2.

2.12 [{Au($^{Cy}Y_S$PCy$_2$)}$_2$(μ-OH)][BF$_4$] (2b)

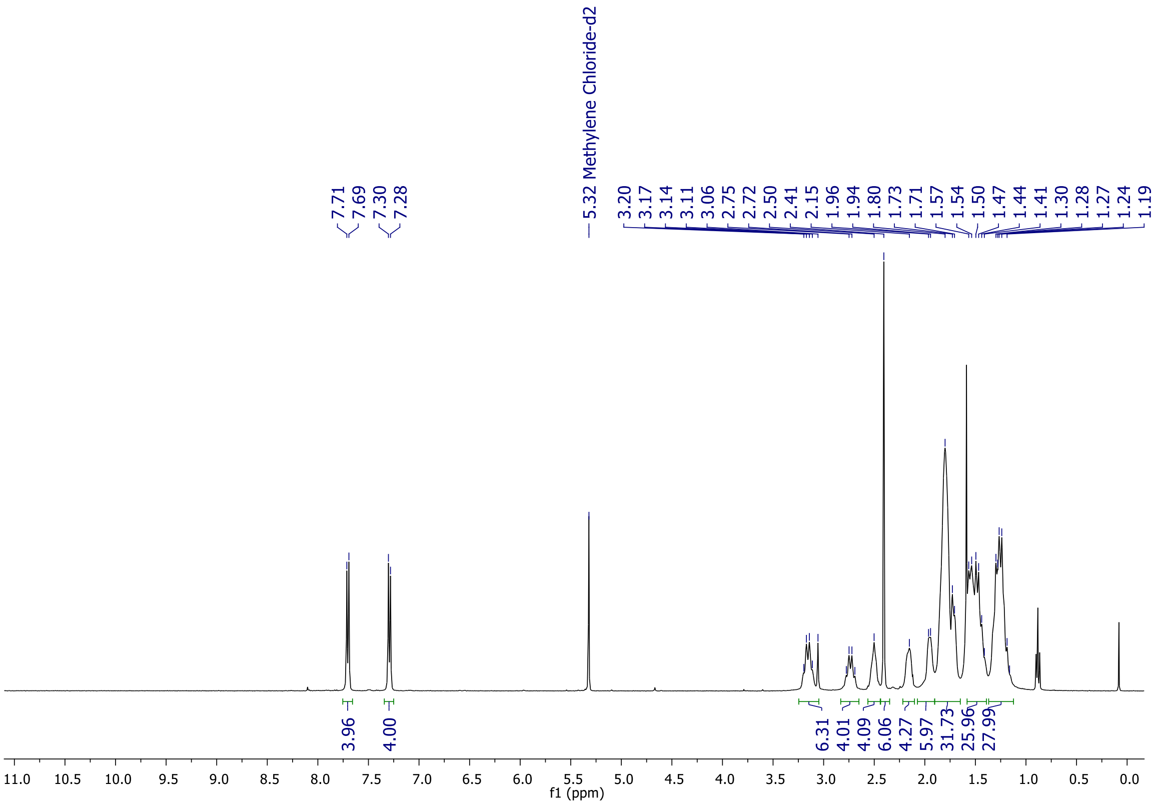

Figure 39. ^{1}H-NMR spectrum of **2b** in CD_2Cl_2.

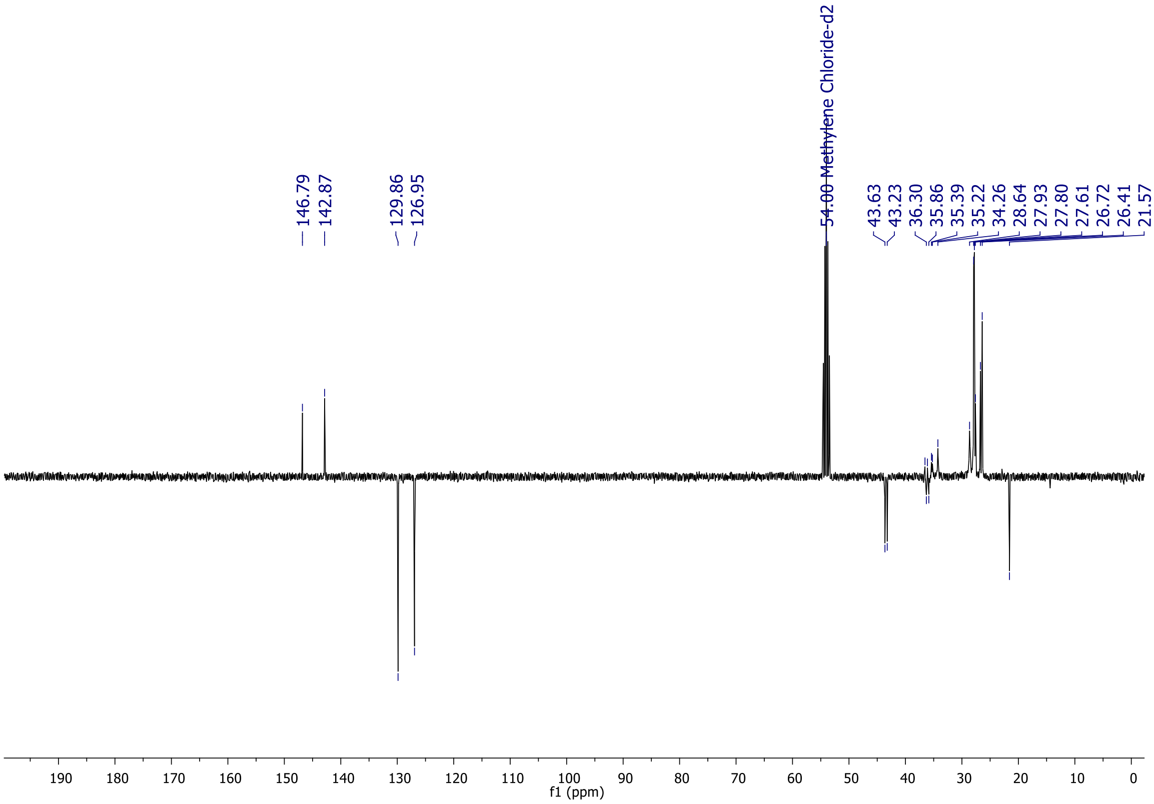

Figure 40. ^{13}C{^{1}H}-NMR spectrum of **2b** in CD_2Cl_2.

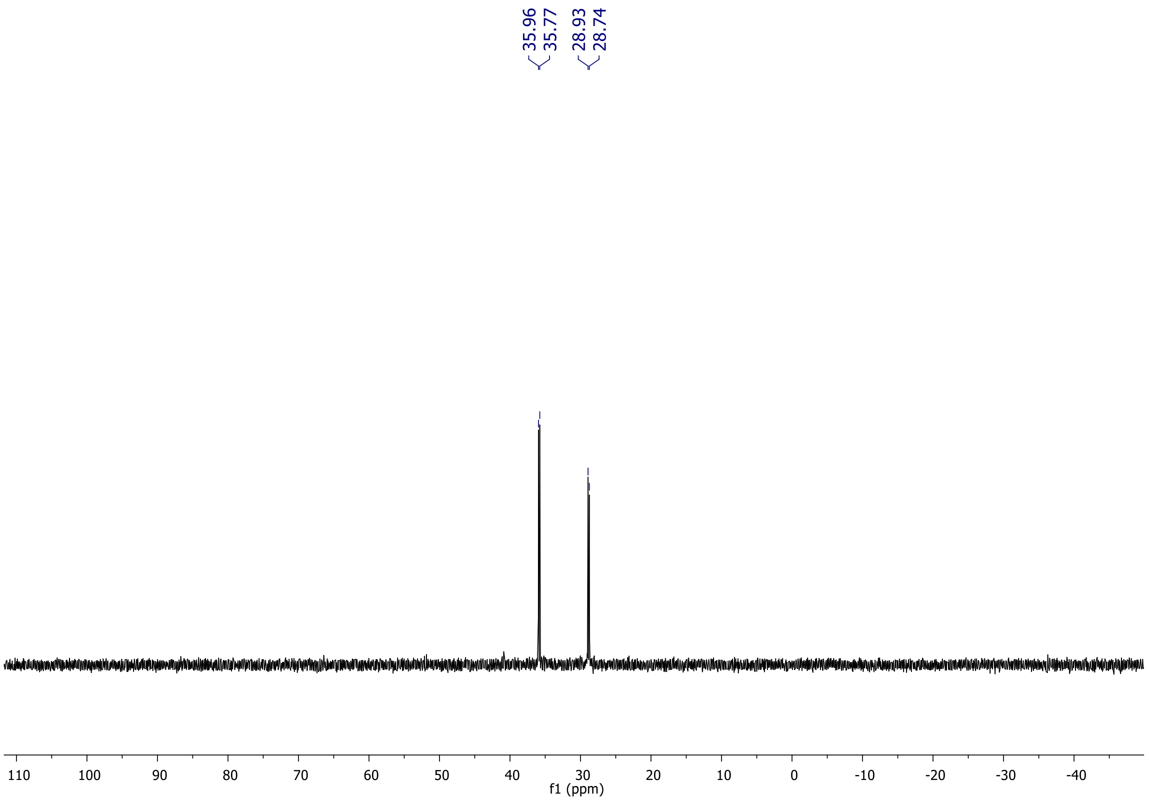

Figure 41. $^{31}P\{^1H\}$-NMR spectrum **2b** in CD_2Cl_2.

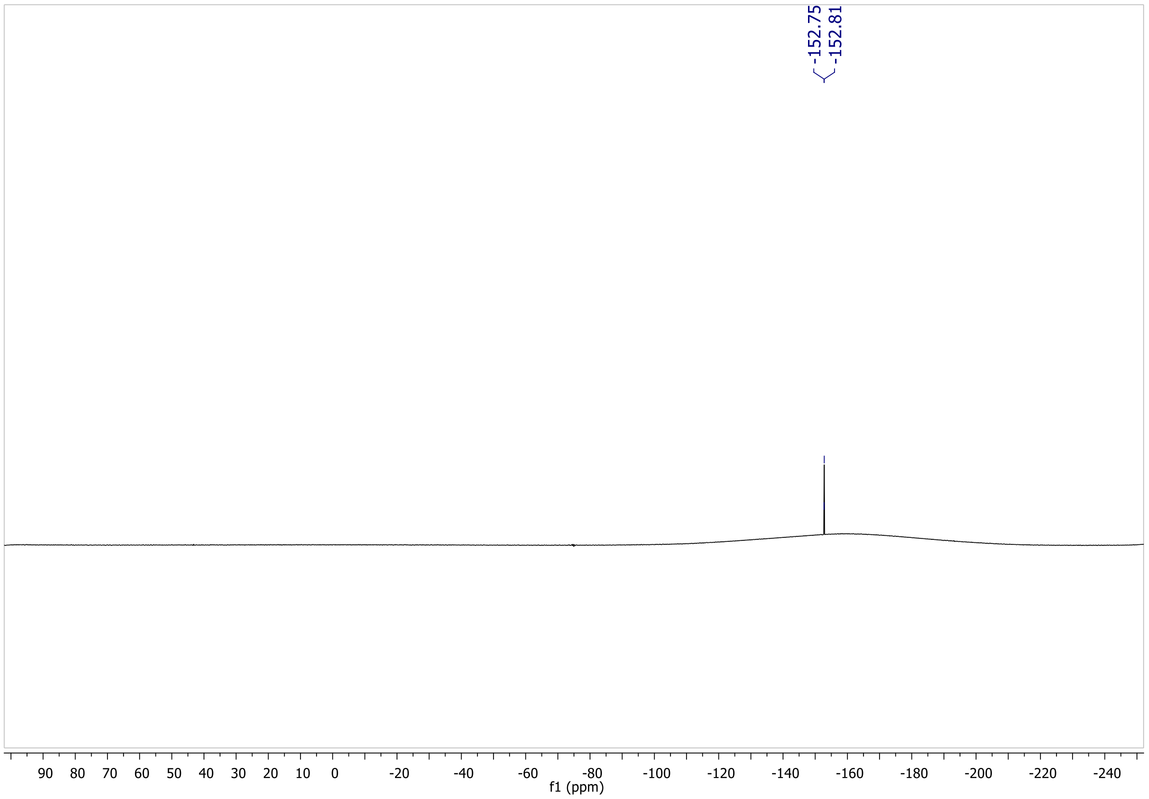

Figure 42. $^{19}F\{^1H\}$-NMR spectrum of **2b** in CD_2Cl_2.

2.13 [Au(Cy-^{Cy}JohnPhos)Cl] (P6)

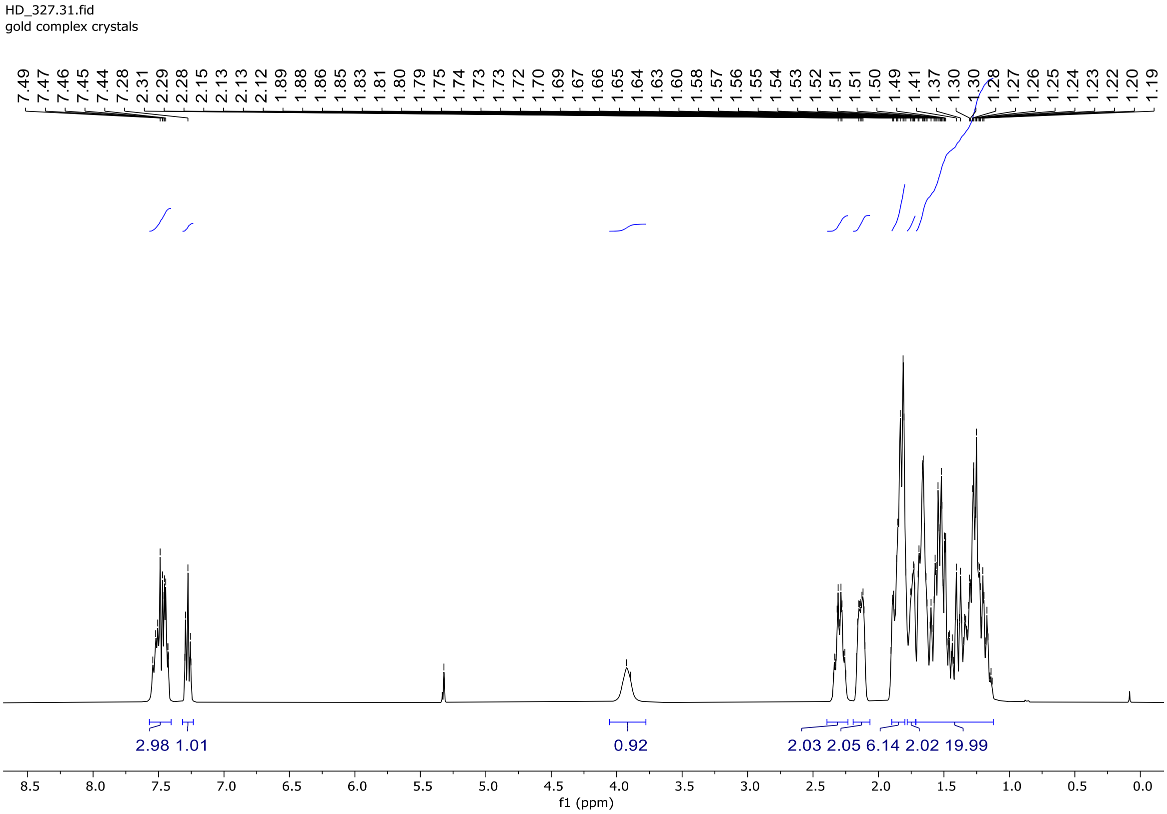

Figure 43. ^{1}H-NMR spectrum of **P6** in CD_2Cl_2.

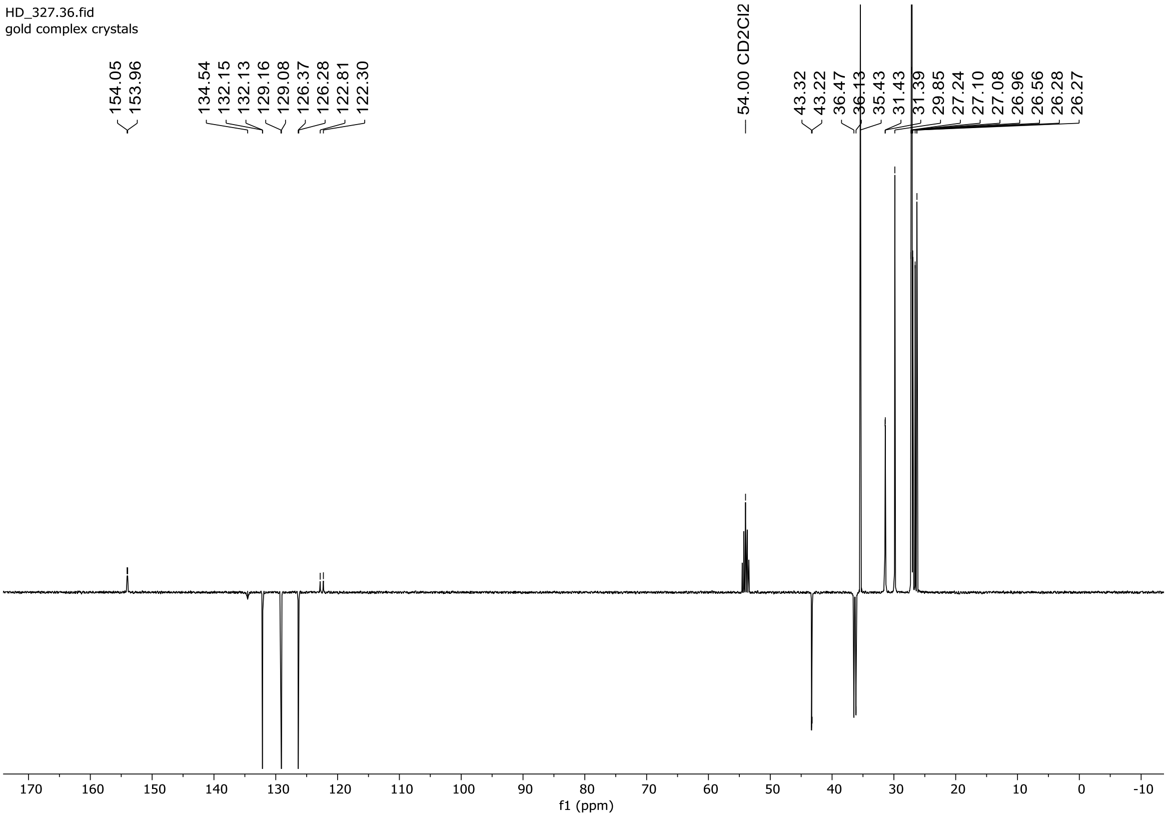

Figure 44. $^{13}C\{^{1}H\}$-NMR spectrum of **P6** in CD_2Cl_2.

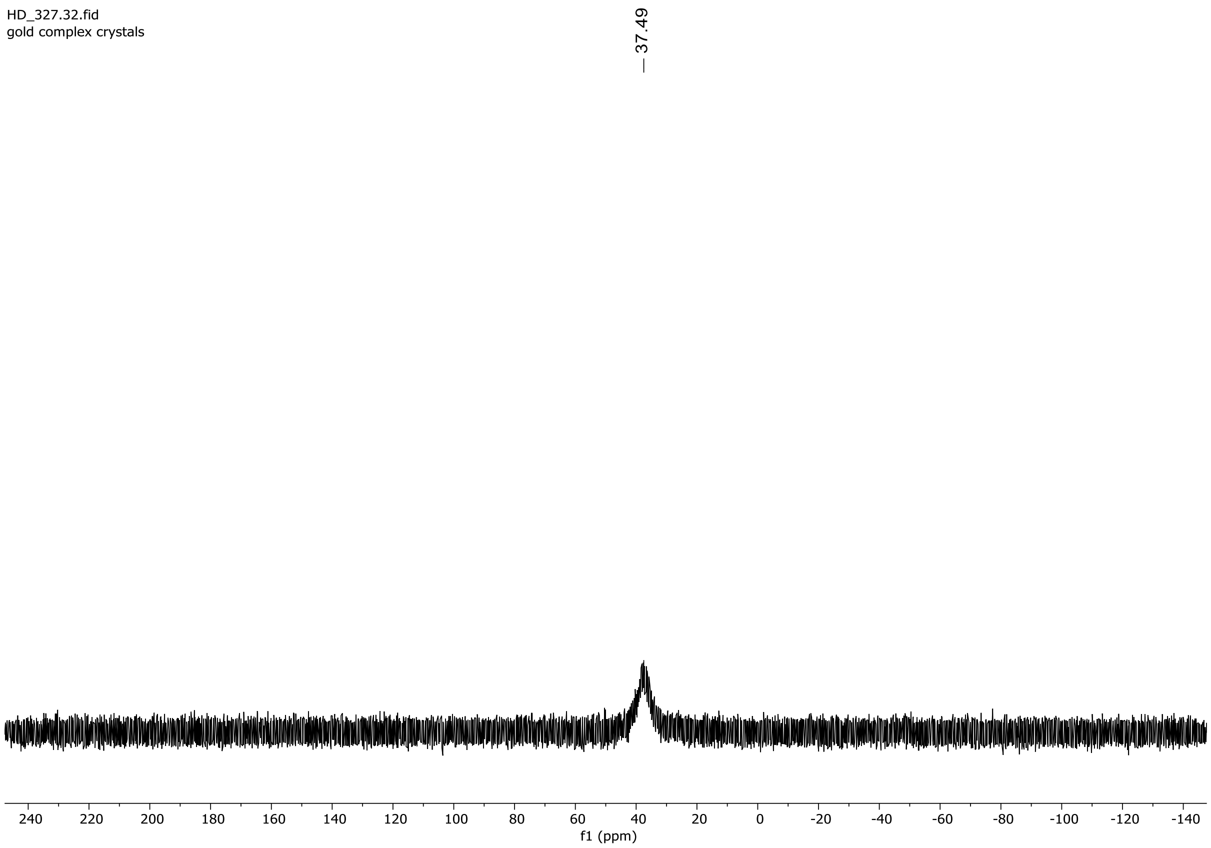

Figure 41. $^{31}P\{^{1}H\}$-NMR spectrum of **P6** in CD_2Cl_2.

3. Crystal Structure Determination

3.1 General information

Data collection of compounds (L1, L4, P1, P4, $^{Cy}Y_{SF}$-H, L5, P5 and P6) was conducted with Oxford Synergy and compounds (1a, 1b and 2a) were collected on a Rigaku Oxford Diffraction Supernova Dual source (Cu at zero) diffractometer equipped with an Atlas CCD detector using ω scans and Cu Kα (λ = 1.54184 Å). The structures were solved using direct methods, refined with the SHELX software package[2] and expanded using Fourier techniques. The crystals of all compounds were mounted in inert oils (perfluoropolyalkylether/Paratone® N). X-ray intensity data were collected at 100 K. Crystallographic data (including structure factors) have been deposited with the Cambridge Crystallographic Data Centre as supplementary publication no. CCDC 2071632-2071638 for **L4**, **P1**, **L1**, **P5**, **P4**, **L5**, **$^{Cy}Y_{SF}$-H** and CCDC 2071003-2071005 for **1a**, **1b** and **2a** as well as CCDC 2078561 for **P6**. These data can be obtained free of charge from the Cambridge Crystallographic Data Centre via www.ccdc.cam.ac.uk/structures.

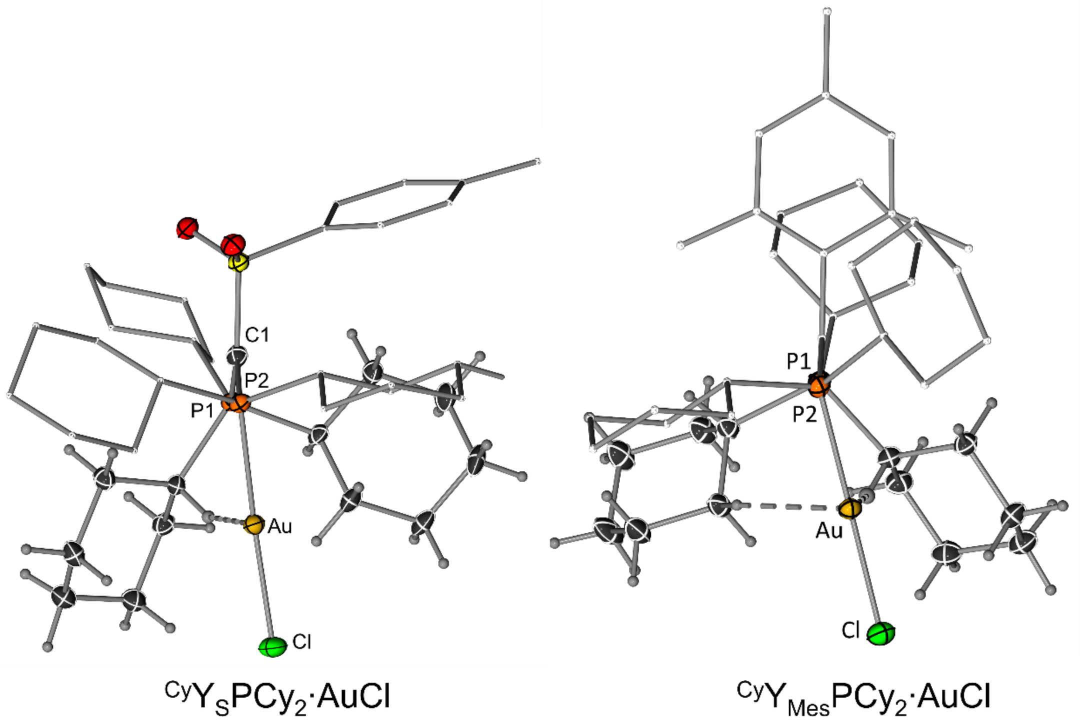

Figure 43. Comparison between the geometry of the complexes **P1** and **P3.**

Table 3. Data collection and structure refinement details for compounds **L1**, **L4** and **P1**.

Compound	L1	L4	P1
CCDC No.	2071634	2071632	2071633
Empirical formula	$C_{44} H_{68} O_2 P_2 S$	$C_{32} H_{54} O_2 P_2 S$	$C_{38} H_{62}$ Au Cl $O_2 P_2$ S
Formula weight	722.98	564.75	877.29
Temperature (K)	100.00(10)	100.00(10)	100.00(10)
Wavelength	1.54184 Å	1.54184 Å	1.54184 Å
Crystal system	Triclinic	Monoclinic	Triclinic
Space group	*P*-1	C2/c	P-1
a (Å)	10.57894(10)	38.4015(4)	10.19965(15)
b (Å)	11.86101(15)	9.60371(10)	10.66040(12)
c (Å)	17.34664(17	16.96876(19)	18.20122(17)
α (°)	71.7478(10)	90	91.7698(9)
β (°)	87.4897(8)	90.9335(10)	105.8271(11)
γ (°)	78.1942(9)	90	97.7483(11)
Volume ($Å^3$)	2022.89(4)	6257.19(12)	1881.89(4)
Z	2	8	2
Density (calculated)	1.187 Mg/m^3	1.199 Mg/m^3	1.548 Mg/m3
Absorption coefficient	1.714 mm^{-1}	2.078 mm^{-1}	9.558 mm^{-1}
F(000)	788	2464	896
Crystal dimensions (mm^3)	0.219 × 0.129 × 0.045	0.28 × 0.210 × 0.016	0.131 x 0.053 x 0.029
Theta range (°)	2.683 to 77.428	4.606 to 77.304	2.530 to 77.813
Index ranges	-13 ≤ h ≤ 12 , -15 ≤ k ≤14 , -21 ≤ l ≤ 19	-48 ≤ h ≤ 42, -12 ≤ k ≤ 6, -20 ≤ l ≤ 21	-12<=h<=12, -11<=k<=13, -19<=l<=22
Reflections collected	27462	19356	28727
Independent reflections	8299 [*R*(int) = 0.0215]	6307 [*R*(int)= 0.0248]	7858 [*R*(int)= 0.0305]
Data / restraints / parameters	8299/0/443	6307/0/339	7858 / 0 / 411
Goodness-of-fit on F^2	1.055	1.057	1.145
Final R indices [I>2sigma(I)]	R_1 = 0.0411, wR_2 = 0.1176	R_1 = 0.0348, wR_2 = 0.0910	R_1 = 0.0284, wR_2 = 0.0785
R indices (all data)	R_1 = 0.0424, wR_2 = 0.1187	R_1 = 0.0371, wR_2 = 0.0923	R_1 = 0.0290, wR_2 = 0.0788
Largest diff. peak and hole	0.626 and -0.521 e.$Å^{-3}$	0.629 and -0.461 e.$Å^{-3}$	1.066 and -1.792 e.Å-3

Table 4. Data collection and structure refinement details for compounds **P4**, **$^{Cy}Y_{SF}$-H** and **L5**.

Compound	P4	$^{Cy}Y_{SF}$-H	L5
CCDC No.	2071636	2071638	2071637
Empirical formula	$C_{39}H_{62}AuClO_2P_2S$	$C_{23}H_{34}F_9O_2PS$	$C_{35}H_{55}F_9O_2P_2S$
Formula weight (g mol^{-1})	889.30	576.53	772.79
Temperature (K)	100.00(10) K	100.00(10)	100.00(10)
Wavelength	1.54184 Å	1.54184 Å	1.54184 Å
Crystal system	Triclinic	Orthorhombic	Triclinic
Space group	P-1	Pbca	P-1
a (Å)	9.28850(14)	19.6559(2)	10.75436(10)
b (Å)	14.2022(3)	11.9311(2)	13.56365(11)
c (Å)	16.1477(2)	22.0689(3)	14.30270(9)
α (°)	70.1408(15)	90	110.3617
β (°)	89.6651(11)	90	100.4968
γ (°)	80.1610(14)	90	97.9783
Volume (Å^3)	1970.88(6)	5176.57(12)	1876.38(3)
Z	2	8	2
Density (calculated)	1.499	1.480	1.368 Mg/m^3
Absorption coefficient	9.135	2.477	2.239 mm^{-1}
F(000)	908	2400	816
Crystal dimensions (mm^3)	0.124 x 0.027 x 0.017	0.242 x 0.200 x 0.024	0.171 x 0.117 x 0.095
Theta range (°)	2.914 to 77.653°.	4.006 to 67.078	3.403 to 77.551
Index ranges	-11<=h<=11,<br>-17<=k<=17,<br>-20<=l<=20	-23 ≤ h ≤ 22,<br>-14 ≤ k ≤ 14,<br>-25 ≤ l ≤ 26	-13<=h<=13,<br>-15<=k<=17,<br>-18<=l<=16
Reflections collected	27759	64699	28860
Independent reflections	27759<br>[*R*(int)=?]	4624<br>[*R*(int)= 0.0487]	7812<br>[*R*(int) = 0.0228]
Data / restraints / parameters	27759 / 405 / 962	4624 / 72 / 374	7812 / 0 / 442
Goodness-of-fit on F^2	1.144	1.059	1.053
Final R indices [I>2sigma(I)]	R_1 = 0.0610,<br>wR_2 = 0.1750	R_1 = 0.0450,<br>wR_2 = 0.1263	R_1 = 0.0281,<br>wR_2 = 0.0736
R indices (all data)	R_1 = 0.0620,<br>wR_2 = 0.1774	R_1 = 0.0469,<br>wR_2 = 0.1283	R_1 = 0.0290,<br>wR_2 = 0.0742
Largest diff. peak and hole	2.533 and<br>-0.938 e.Å-3	0.531 and<br>-0.425 e.Å-3	0.368 and<br>-0.404 e.Å^{-3}

Table 5. Data collection and structure refinement details for compounds **P5** and **P6**.

**Compound**	**P5**	**P6**
CCDC No.	2071635	2078561
Empirical formula	$C_{35}H_{55}AuClF_9O_2P_2S$	$C_{24}H_{37}AuClP$
Formula weight	1005.20	588.92
Temperature (K)	100(2)	100(2)
Wavelength	1.54184 Å	1.54184 Å
Crystal system	Monoclinic	Triclinic
Space group	P 21/n	P-1
a (Å)	11.98170(10)	9.63402(18)
b (Å)	13.78420(10)	9.91609(15)
c (Å)	24.2263(3)	13.07001(14)
α (°)	90	105.1916(12)
β (°)	90.0340(10)	104.0487(13)(10)
γ (°)	90	99.8202(14)
Volume ($Å^3$)	4001.17(7)	1131.77(3)
Z	4	2
Density (calculated)	1.669 Mg/m3	1.728 Mg/m3
Absorption coefficient	9.411 mm^{-1}	13.996 mm^{-1}
F(000)	2016	584
Crystal dimensions (mm^3)	0.556 x 0.097 x 0.063	0.201 x 0.163 x 0.093
Theta range (°)	4.115 to 72.244	3.676 to 76.973
Index ranges	-14<=h<=14, -16<=k<=12, -29<=l<=29	-11<=h<=12, -12<=k<=12, -13<=l<=16
Reflections collected	55890	12713
Independent reflections	7867 [*R*(int) = 0.0668]	4570 [*R*(int) = 0.0302]
Data / restraints / parameters	7867 / 0 / 464	4570 / 0 / 248
Goodness-of-fit on F^2	1.040	1.040
Final R indices [I>2sigma(I)]	R_1 = 0.0308, wR_2 = 0.0803	R_1 = 0.0239, wR_2 = 0.0622
R indices (all data)	R_1 = 0.0333, wR_2 = 0.0819	R_1 = 0.0287, wR_2 = 0.0639
Largest diff. peak and hole	1.262 and -2.261 e.$Å^{-3}$	1.164 and -1.474 e.$Å^{-3}$

Table 6. Data collection and structure refinement details for compounds **1a**, **1b** and **2a**.

Compound	1a	1b	2a
CCDC No.	2071003	2071004	2071005
Empirical formula	$C_{45} H_{51} Au O_3 P_2 S$	$C_{46} H_{71} Au Cl_2 O_3 P_2 S$	$C_{79} H_{95} Au_2 B Cl_6 F_4 O_5 P_4 S_2$
Formula weight (g mol^{-1})	930.83	1033.90	2006.01
Temperature (K)	100.00(1)	100.00(10)	100.00(10)
Wavelength	1.54184 Å	1.54184 Å	1.54184 Å
Crystal system	Triclinic	Monoclinic	Triclinic
Space group	*P*-1	$P2_1$	P-1
a (Å)	10.8341(3)	12.27527(11)	15.8913(3)
b (Å)	13.6098(3)	29.4293(2)	17.0061(4)
c (Å)	15.2133(4)	13.22499(10)	18.2418(4)
α (°)	109.811(2)	90	108.226(2)
β (°)	104.848(2)	106.9116(8)	102.715(2)
γ (°)	91.308(2)	90	110.786(2)
Volume (Å^3)	2025.08(9)	4570.96(6)	4062.1(2)
Z	2	4	2
Density (calculated)	1.526 Mg/m^3	1.502 Mg/m^3	1.640 Mg/m3
Absorption coefficient	8.357 mm^{-1}	8.506 mm^{-1}	10.212 mm^{-1}
F(000)	940.0	2120.0	2004.0
Crystal dimensions (mm^3)	0.09 × 0.04 × 0.01	0.121 × 0.104 × 066	0.188 × 0.099 × 0.067
Theta range (°)	6.434 to 147.902	6.006 to 147.672	5.496 to 147.816
Index ranges	-13 ≤ h ≤ 11, -16 ≤ k ≤ 16, -18 ≤ l ≤ 18	-15 ≤ h ≤ 15, -36 ≤ k ≤ 31, -16 ≤ l ≤ 16	-19 ≤ h ≤ 19, -21 ≤ k ≤ 20, -22 ≤ l ≤ 22
Reflections collected	30282	44313	76522
Independent reflections	8025 [*R*(int) = 0.0234]	16923 [*R*(int)= 0.0424]	16193 [*R*(int)= 0.0706]
Data / restraints / parameters	8025/0/471	16923/1/996	16193/13/933
Goodness-of-fit on F^2	1.069	1.034	1.023
Final R indices [I>2sigma(I)]	R_1 = 0.0175, wR_2 = 0.0435	R_1 = 0.0301, wR_2 = 0.0635	R_1 = 0.0395, wR_2 = 0.0986
R indices (all data)	R_1 = 0.0178, wR_2 = 0.0437	R_1 = 0.0342, wR_2 = 0.0651	R_1 = 0.0519, wR_2 = 0.1066
Largest diff. peak and hole	0.91 and -0.86 e.Å^{-3}	1.04 and -0.49 e.Å^{-3}	1.56 and -1.42 e.Å-3

3.2 Crystal Structure of $^{Cy}Y_S$-PCy_2 (L1)

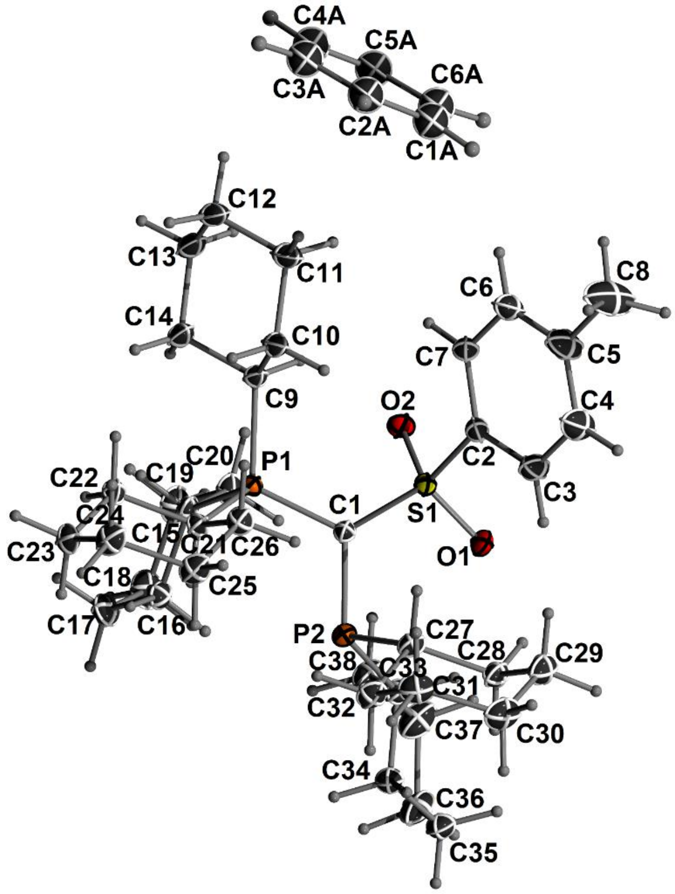

Figure 44. ORTEP plot of **L1** with one molecule of benzene**;** thermal displacement ellipsoids shown at the 50% probability level. Selected bond lengths [Å] and angles [°]: P(1)-C(1) 1.7570(15), P(2)-C(1) 1.8182(15), S(1)-C(1) 1.7097(15), S(1)-O(1) 1.4496(11); P(1)-C(1)-P(2) 114.66(8), S(1)-C(1)-P(1)121.49(9), S(1)-C(1)-P(2) 123.22(8).

Table 7. Atomic Coordinates (×10^4) and Equivalent Isotropic Displacement Parameters (Å^2×10^3) for **L1**. U_{eq} is defined as 1/3 of of the trace of the orthogonalised U_{ij} tensor.

Atom	*x*	*y*	*z*	U(eq)
S(1)	6801(1)	5451(1)	3433(1)	15(1)
P(1)	4626(1)	5511(1)	2336(1)	13(1)
P(2)	7311(1)	5372(1)	1673(1)	15(1)
O(1)	8044(1)	5808(1)	3308(1)	22(1)
O(2)	5789(1)	6164(1)	3779(1)	18(1)
C(1)	6274(1)	5372(1)	2537(1)	14(1)
C(2)	7145(2)	3997(2)	4188(1)	19(1)
C(3)	8360(2)	3263(2)	4224(1)	30(1)
C(4)	8660(2)	2166(2)	4833(1)	38(1)
C(5)	7749(2)	1764(2)	5409(1)	36(1)

C(6)	6548(2)	2524(2)	5377(1)	26(1)
C(7)	6252(2)	3638(2)	4780(1)	20(1)
C(8)	8054(3)	542(2)	6054(2)	59(1)
C(9)	3643(2)	5073(2)	3256(1)	18(1)
C(10)	3802(2)	3685(2)	3622(1)	21(1)
C(11)	3106(2)	3341(2)	4430(1)	29(1)
C(12)	1673(2)	3929(2)	4320(1)	33(1)
C(13)	1481(2)	5300(2)	3942(1)	31(1)
C(14)	2189(2)	5666(2)	3138(1)	24(1)
C(15)	3807(2)	7064(1)	1763(1)	17(1)
C(16)	4229(2)	7494(2)	878(1)	20(1)
C(17)	3496(2)	8776(2)	440(1)	25(1)
C(18)	3659(2)	9667(2)	883(1)	29(1)
C(19)	3238(2)	9243(2)	1758(1)	26(1)
C(20)	3967(2)	7963(2)	2210(1)	20(1)
C(21)	4484(2)	4623(1)	1654(1)	16(1)
C(22)	3085(2)	4708(2)	1393(1)	19(1)
C(23)	3079(2)	4136(2)	716(1)	23(1)
C(24)	3832(2)	2832(2)	962(1)	26(1)
C(25)	5208(2)	2746(2)	1243(1)	24(1)
C(26)	5200(2)	3293(1)	1936(1)	18(1)
C(27)	8503(2)	3880(1)	1991(1)	18(1)
C(28)	9870(2)	3845(2)	2273(1)	20(1)
C(29)	10620(2)	2530(2)	2589(1)	25(1)
C(30)	10671(2)	1879(2)	1955(1)	30(1)
C(31)	9326(2)	1950(2)	1638(1)	28(1)
C(32)	8603(2)	3268(2)	1320(1)	22(1)
C(33)	8360(2)	6505(1)	1556(1)	18(1)
C(34)	9136(2)	6623(2)	773(1)	22(1)
C(35)	10104(2)	7441(2)	732(1)	25(1)
C(36)	9416(2)	8694(2)	753(1)	32(1)
C(37)	8553(2)	8614(2)	1486(1)	31(1)
C(38)	7616(2)	7766(2)	1536(1)	24(1)
C1A1	4180(4)	-339(3)	6393(2)	83(1)
C2A1	3177(3)	-799(3)	6191(2)	74(1)
C3A1	1932(3)	-129(3)	6118(2)	71(1)
C4A1	1695(4)	953(3)	6289(2)	69(1)
C5A1	2647(4)	1388(3)	6490(2)	73(1)

C6A1	3923(4)	712(3)	6569(2)	76(1)

Table 8. Anisotropic displacement parameters (Å^2×10^3) for **L1.** The anisotropic displacement factor exponent takes the form: $-2\pi^2[h^2 a^{*2}U^{11} + ... + 2 h k a^* b^* U^{12}]$

Atom	U^{11}	U^{22}	U^{33}	U^{23}	U^{13}	U^{12}
S(1)	12(1)	20(1)	14(1)	-7(1)	0(1)	-3(1)
P(1)	11(1)	16(1)	13(1)	-6(1)	0(1)	-2(1)
P(2)	12(1)	17(1)	15(1)	-5(1)	1(1)	-3(1)
O(1)	15(1)	33(1)	20(1)	-10(1)	1(1)	-10(1)
O(2)	18(1)	21(1)	19(1)	-11(1)	2(1)	-3(1)
C(1)	11(1)	18(1)	13(1)	-6(1)	0(1)	-2(1)
C(2)	16(1)	25(1)	15(1)	-6(1)	-2(1)	0(1)
C(3)	21(1)	38(1)	22(1)	-3(1)	4(1)	4(1)
C(4)	29(1)	39(1)	29(1)	-1(1)	4(1)	12(1)
C(5)	36(1)	33(1)	25(1)	1(1)	4(1)	8(1)
C(6)	26(1)	28(1)	18(1)	-4(1)	4(1)	-1(1)
C(7)	17(1)	25(1)	17(1)	-8(1)	1(1)	-2(1)
C(8)	56(2)	43(1)	44(1)	14(1)	14(1)	19(1)
C(9)	14(1)	27(1)	16(1)	-10(1)	3(1)	-7(1)
C(10)	22(1)	28(1)	17(1)	-8(1)	3(1)	-12(1)
C(11)	31(1)	42(1)	18(1)	-9(1)	6(1)	-20(1)
C(12)	27(1)	58(1)	22(1)	-16(1)	10(1)	-24(1)
C(13)	18(1)	56(1)	27(1)	-22(1)	8(1)	-12(1)
C(14)	14(1)	37(1)	24(1)	-15(1)	5(1)	-6(1)
C(15)	13(1)	18(1)	19(1)	-8(1)	-1(1)	0(1)
C(16)	19(1)	20(1)	20(1)	-6(1)	-1(1)	-2(1)
C(17)	27(1)	21(1)	23(1)	-2(1)	-4(1)	-3(1)
C(18)	34(1)	17(1)	34(1)	-4(1)	-6(1)	-3(1)
C(19)	25(1)	20(1)	36(1)	-13(1)	-4(1)	2(1)
C(20)	18(1)	20(1)	24(1)	-10(1)	-2(1)	0(1)
C(21)	15(1)	19(1)	15(1)	-8(1)	1(1)	-4(1)
C(22)	15(1)	23(1)	20(1)	-10(1)	-1(1)	-5(1)
C(23)	22(1)	32(1)	22(1)	-14(1)	-1(1)	-10(1)
C(24)	25(1)	31(1)	31(1)	-20(1)	3(1)	-10(1)
C(25)	22(1)	26(1)	30(1)	-18(1)	4(1)	-6(1)
C(26)	18(1)	18(1)	21(1)	-9(1)	1(1)	-4(1)

C(27)	15(1)	17(1)	20(1)	-6(1)	3(1)	-3(1)
C(28)	15(1)	20(1)	22(1)	-5(1)	1(1)	-2(1)
C(29)	19(1)	23(1)	29(1)	-5(1)	0(1)	1(1)
C(30)	26(1)	23(1)	35(1)	-8(1)	5(1)	4(1)
C(31)	31(1)	23(1)	32(1)	-13(1)	6(1)	-4(1)
C(32)	20(1)	24(1)	26(1)	-12(1)	2(1)	-4(1)
C(33)	15(1)	18(1)	20(1)	-5(1)	1(1)	-4(1)
C(34)	20(1)	25(1)	20(1)	-4(1)	2(1)	-6(1)
C(35)	19(1)	29(1)	25(1)	-2(1)	3(1)	-8(1)
C(36)	24(1)	24(1)	43(1)	1(1)	1(1)	-10(1)
C(37)	26(1)	20(1)	50(1)	-11(1)	4(1)	-6(1)
C(38)	18(1)	18(1)	33(1)	-5(1)	3(1)	-4(1)
C1A1	100(3)	54(2)	89(3)	-8(2)	-34(2)	-15(2)
C2A1	79(2)	47(2)	100(3)	-32(2)	-31(2)	-1(2)
C3A1	71(2)	61(2)	92(2)	-40(2)	-12(2)	-6(2)
C4A1	100(3)	49(2)	61(2)	-20(1)	14(2)	-18(2)
C5A1	122(3)	60(2)	49(2)	-26(1)	24(2)	-38(2)
C6A1	108(3)	80(2)	56(2)	-21(2)	-4(2)	-54(2)

3.3 Crystal Structure of $^{Cy}Y_S$-P*i*Pr$_2$ (L4)

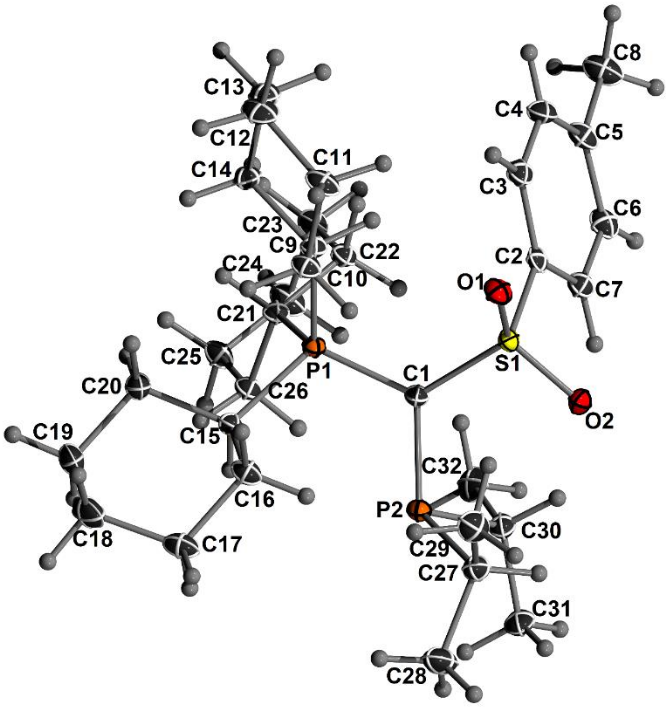

Figure 45. ORTEP plot of **L4;** thermal displacement ellipsoids shown at the 50% probability level. Selected bond lengths [Å] and angles [°]: P(1)-C(1) 1.7614(14), P(2)-C(1) 1.8079(14), S(1)-C(1) 1.7220(14), S(1)-O(1) 1.4517(10); P(1)-C(1)-P(2) 115.83(7), S(1)-C(1)-P(1) 122.33(8), S(1)-C(1)-P(2) 123.22(8).

Table 9. Atomic Coordinates (×10^4) and Equivalent Isotropic Displacement Parameters (Å^2×10^3) for **L4**. U_{eq} is defined as 1/3 of of the trace of the orthogonalised U_{ij} tensor.

Atom	*x*	*y*	*z*	U(eq)
S(1)	3677(1)	5130(1)	3234(1)	13(1)
P(1)	3839(1)	4495(1)	4964(1)	11(1)
P(2)	3922(1)	2210(1)	3756(1)	14(1)
O(1)	3870(1)	6429(1)	3267(1)	17(1)
O(2)	3685(1)	4362(1)	2497(1)	17(1)
C(1)	3797(1)	3983(1)	3970(1)	13(1)
C(2)	3231(1)	5651(2)	3323(1)	14(1)
C(3)	3146(1)	6950(2)	3628(1)	17(1)
C(4)	2798(1)	7326(2)	3695(1)	19(1)
C(5)	2532(1)	6439(2)	3448(1)	19(1)
C(6)	2623(1)	5166(2)	3105(1)	19(1)
C(7)	2969(1)	4770(2)	3049(1)	17(1)
C(8)	2154(1)	6822(2)	3558(1)	26(1)
C(9)	3880(1)	6399(1)	5044(1)	13(1)
C(10)	4250(1)	6913(2)	4863(1)	16(1)

C(11)	4252(1)	8501(2)	4791(1)	20(1)
C(12)	4117(1)	9195(2)	5537(1)	23(1)
C(13)	3756(1)	8646(2)	5736(1)	20(1)
C(14)	3754(1)	7059(2)	5816(1)	16(1)
C(15)	4202(1)	3574(2)	5451(1)	14(1)
C(16)	4552(1)	3610(2)	5030(1)	18(1)
C(17)	4803(1)	2572(2)	5416(1)	22(1)
C(18)	4858(1)	2883(2)	6292(1)	28(1)
C(19)	4510(1)	2929(2)	6718(1)	25(1)
C(20)	4254(1)	3952(2)	6326(1)	19(1)
C(21)	3465(1)	4041(1)	5587(1)	13(1)
C(22)	3122(1)	4579(2)	5216(1)	15(1)
C(23)	2810(1)	4256(2)	5734(1)	18(1)
C(24)	2786(1)	2702(2)	5903(1)	22(1)
C(25)	3122(1)	2184(2)	6292(1)	18(1)
C(26)	3440(1)	2478(2)	5784(1)	15(1)
C(27)	4258(1)	2226(2)	2951(1)	19(1)
C(28)	4495(1)	941(2)	3055(1)	27(1)
C(29)	4487(1)	3522(2)	2881(1)	23(1)
C(30)	3549(1)	1411(2)	3188(1)	17(1)
C(31)	3648(1)	-26(2)	2865(1)	23(1)
C(32)	3228(1)	1263(2)	3704(1)	23(1)

Table 10. Anisotropic displacement parameters (Å^2×10^3) for **L4.** The anisotropic displacement factor exponent takes the form: $-2\pi^2[h^2 a^{*2}U^{11} + ... + 2 h k a^* b^* U^{12}]$

Atom	U^{11}	U^{22}	U^{33}	U^{23}	U^{13}	U^{12}
S(1)	15(1)	12(1)	12(1)	1(1)	1(1)	1(1)
P(1)	12(1)	10(1)	12(1)	0(1)	0(1)	0(1)
P(2)	17(1)	11(1)	13(1)	0(1)	0(1)	1(1)
O(1)	20(1)	13(1)	18(1)	3(1)	1(1)	-1(1)
O(2)	22(1)	16(1)	12(1)	0(1)	2(1)	3(1)
C(1)	16(1)	10(1)	12(1)	0(1)	-1(1)	1(1)
C(2)	16(1)	14(1)	11(1)	4(1)	0(1)	2(1)
C(3)	21(1)	13(1)	18(1)	2(1)	-3(1)	0(1)
C(4)	22(1)	13(1)	22(1)	1(1)	-1(1)	4(1)
C(5)	19(1)	16(1)	21(1)	6(1)	0(1)	1(1)
C(6)	20(1)	15(1)	21(1)	4(1)	-2(1)	-3(1)

C(7)	21(1)	14(1)	16(1)	2(1)	-1(1)	0(1)
C(8)	19(1)	21(1)	38(1)	4(1)	1(1)	2(1)
C(9)	15(1)	10(1)	15(1)	0(1)	0(1)	0(1)
C(10)	15(1)	12(1)	20(1)	2(1)	0(1)	-1(1)
C(11)	18(1)	14(1)	28(1)	5(1)	0(1)	-1(1)
C(12)	26(1)	10(1)	33(1)	-1(1)	-2(1)	-2(1)
C(13)	26(1)	13(1)	22(1)	-2(1)	2(1)	1(1)
C(14)	21(1)	12(1)	16(1)	-2(1)	2(1)	0(1)
C(15)	15(1)	11(1)	16(1)	0(1)	-2(1)	2(1)
C(16)	14(1)	18(1)	23(1)	2(1)	0(1)	1(1)
C(17)	16(1)	19(1)	32(1)	1(1)	-1(1)	4(1)
C(18)	23(1)	27(1)	35(1)	-1(1)	-12(1)	7(1)
C(19)	31(1)	24(1)	21(1)	1(1)	-9(1)	8(1)
C(20)	23(1)	17(1)	17(1)	-2(1)	-5(1)	4(1)
C(21)	14(1)	12(1)	14(1)	0(1)	1(1)	0(1)
C(22)	16(1)	13(1)	15(1)	2(1)	0(1)	1(1)
C(23)	16(1)	17(1)	20(1)	3(1)	2(1)	1(1)
C(24)	16(1)	19(1)	31(1)	7(1)	1(1)	-2(1)
C(25)	19(1)	15(1)	20(1)	5(1)	1(1)	-1(1)
C(26)	16(1)	12(1)	16(1)	2(1)	0(1)	0(1)
C(27)	21(1)	19(1)	17(1)	0(1)	2(1)	5(1)
C(28)	29(1)	24(1)	29(1)	2(1)	6(1)	11(1)
C(29)	21(1)	24(1)	23(1)	4(1)	6(1)	2(1)
C(30)	23(1)	13(1)	16(1)	0(1)	-3(1)	-1(1)
C(31)	33(1)	13(1)	21(1)	-4(1)	-5(1)	-1(1)
C(32)	24(1)	22(1)	22(1)	-1(1)	-2(1)	-6(1)

3.4 Crystal Structure of [Au($^{Cy}Y_S$-PCy_2)Cl] (P1)

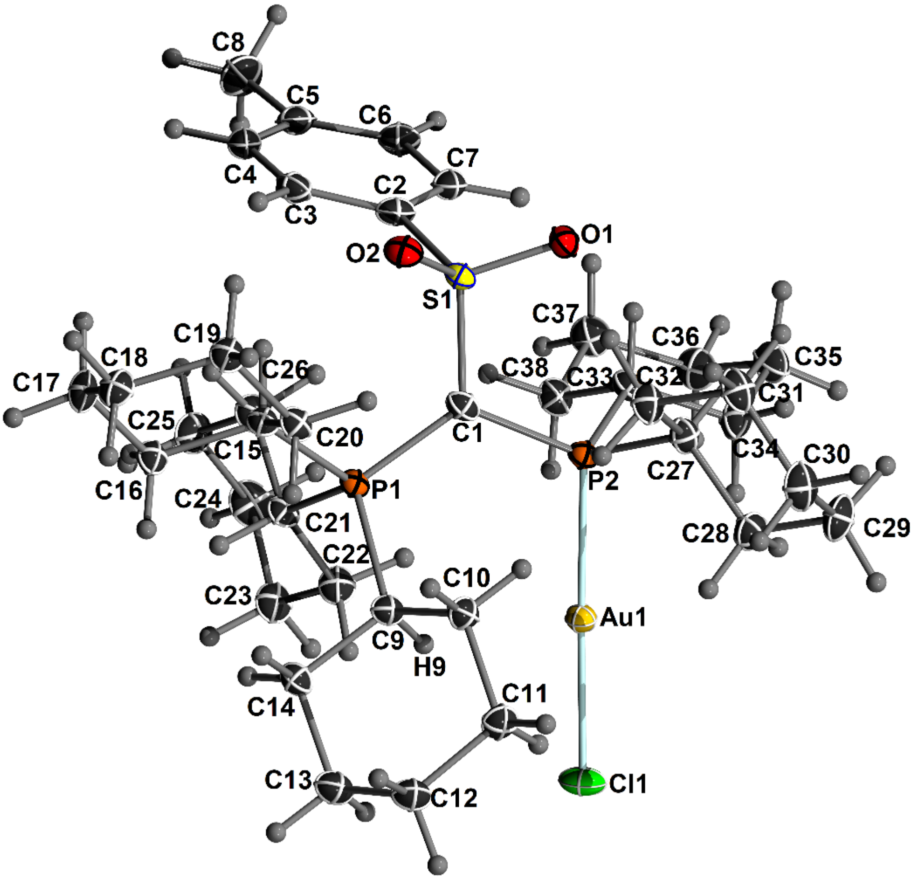

Figure 46. ORTEP plot of **P1;** thermal displacement ellipsoids shown at the 50% probability level. Selected bond lengths [Å] and angles [°]: P(1)-C(1) 1.772(3), P(2)-C(1) 1.796(3), S(1)-C(1) 1.743(3), S(1)-O(1) 1.4517(10), Au(1)-P(2) 2.2639(8), Au(1)-Cl(1) 2.2919(8), Au(1)-C(9) 3.264(3), Au(1)-H(9) 2.39(4); P(1)-C(1)-P(2) 122.74(18), S(1)-C(1)-P(1) 122.23(19), S(1)-C(1)-P(2) 115.02(17), P(2)-Au(1)-Cl(1) 177.17(3)**,** P(2)-Au(1)-C(9) 78.03(6), Cl(1)-Au(1)-C(9) 104.57(6), P(2)-Au(1)-H(9) 88.4(10), Cl(1)-Au(1)-H(9) 94.1(10), C(9)-Au(1)-H(9) 11.4(10)**.**

Table 11. Atomic Coordinates (×10^4) and Equivalent Isotropic Displacement Parameters (Å^2×10^3) for **P1**. U_{eq} is defined as 1/3 of of the trace of the orthogonalised U_{ij} tensor.

Atom	*x*	*y*	*z*	U(eq)
Au(1)	4950(1)	1185(1)	7347(1)	16(1)
Cl(1)	5909(1)	-180(1)	6723(1)	27(1)
S(1)	3433(1)	5141(1)	8199(1)	15(1)
P(1)	4075(1)	4355(1)	6707(1)	13(1)
P(2)	4080(1)	2519(1)	8018(1)	15(1)
O(1)	3169(2)	4477(2)	8839(1)	19(1)
O(2)	2374(2)	5818(2)	7766(1)	19(1)
C(1)	3874(3)	4026(3)	7622(2)	16(1)
C(2)	4870(3)	6342(3)	8608(2)	17(1)
C(3)	5035(4)	7476(3)	8270(2)	20(1)

C(4)	6204(4)	8353(3)	8573(2)	21(1)
C(5)	7212(4)	8126(3)	9228(2)	23(1)
C(6)	6991(4)	7014(3)	9587(2)	22(1)
C(7)	5833(4)	6126(3)	9282(2)	19(1)
C(8)	8504(4)	9057(4)	9540(2)	31(1)
C(9)	3472(3)	2945(3)	6024(2)	15(1)
C(10)	2085(3)	2174(3)	6017(2)	19(1)
C(11)	1880(4)	906(3)	5554(2)	21(1)
C(12)	1979(4)	1088(3)	4738(2)	22(1)
C(13)	3327(4)	1911(3)	4747(2)	22(1)
C(14)	3513(3)	3192(3)	5199(2)	18(1)
C(15)	3158(3)	5675(3)	6337(2)	15(1)
C(16)	3647(3)	6399(3)	5715(2)	18(1)
C(17)	2921(4)	7559(3)	5537(2)	21(1)
C(18)	1355(3)	7213(3)	5304(2)	21(1)
C(19)	877(3)	6469(3)	5916(2)	19(1)
C(20)	1580(3)	5288(3)	6073(2)	17(1)
C(21)	5876(3)	4838(3)	6677(2)	16(1)
C(22)	6761(3)	3759(3)	6779(2)	20(1)
C(23)	8227(3)	4249(3)	6757(2)	22(1)
C(24)	8900(4)	5328(4)	7359(2)	26(1)
C(25)	8061(4)	6421(4)	7235(2)	24(1)
C(26)	6572(3)	5981(3)	7247(2)	20(1)
C(27)	2453(3)	1702(3)	8198(2)	18(1)
C(28)	2270(4)	270(3)	7961(2)	22(1)
C(29)	1007(4)	-469(4)	8140(2)	27(1)
C(30)	-301(4)	87(4)	7774(2)	27(1)
C(31)	-125(4)	1487(4)	8028(2)	24(1)
C(32)	1115(3)	2244(3)	7832(2)	22(1)
C(33)	5366(3)	2751(3)	8971(2)	18(1)
C(34)	5524(4)	1498(3)	9361(2)	21(1)
C(35)	6602(4)	1710(4)	10140(2)	25(1)
C(36)	7989(4)	2395(4)	10097(2)	26(1)
C(37)	7827(4)	3627(4)	9700(2)	24(1)
C(38)	6772(3)	3379(3)	8912(2)	20(1)

Table 12. Anisotropic displacement parameters ($Å^2 \times 10^3$) for **P1**. The anisotropic displacement factor exponent takes the form: $-2\pi^2[h^2 a^{*2}U^{11} + ... + 2 h k a^* b^* U^{12}]$

Atom	U^{11}	U^{22}	U^{33}	U^{23}	U^{13}	U^{12}
Au(1)	16(1)	17(1)	16(1)	1(1)	5(1)	4(1)
Cl(1)	28(1)	31(1)	26(1)	-6(1)	8(1)	11(1)
S(1)	14(1)	18(1)	14(1)	0(1)	5(1)	3(1)
P(1)	11(1)	14(1)	14(1)	1(1)	4(1)	2(1)
P(2)	14(1)	15(1)	16(1)	2(1)	6(1)	3(1)
O(1)	20(1)	20(1)	17(1)	2(1)	8(1)	2(1)
O(2)	16(1)	23(1)	20(1)	0(1)	5(1)	6(1)
C(1)	15(1)	20(2)	15(1)	1(1)	5(1)	1(1)
C(2)	14(1)	19(2)	17(1)	-5(1)	4(1)	2(1)
C(3)	24(2)	23(2)	14(1)	1(1)	4(1)	7(1)
C(4)	31(2)	16(2)	19(2)	1(1)	11(1)	5(1)
C(5)	27(2)	19(2)	24(2)	-6(1)	11(1)	1(1)
C(6)	20(2)	24(2)	19(2)	-3(1)	2(1)	3(1)
C(7)	23(2)	18(2)	18(2)	0(1)	7(1)	5(1)
C(8)	28(2)	28(2)	33(2)	-2(2)	6(2)	-2(2)
C(9)	12(1)	17(1)	16(1)	-2(1)	4(1)	1(1)
C(10)	18(2)	19(2)	18(2)	0(1)	6(1)	0(1)
C(11)	20(2)	19(2)	20(2)	-1(1)	2(1)	-1(1)
C(12)	23(2)	23(2)	21(2)	-3(1)	5(1)	2(1)
C(13)	21(2)	25(2)	20(2)	-4(1)	7(1)	2(1)
C(14)	17(2)	21(2)	16(1)	2(1)	6(1)	4(1)
C(15)	14(1)	16(1)	15(1)	1(1)	4(1)	3(1)
C(16)	16(2)	18(2)	21(2)	4(1)	8(1)	4(1)
C(17)	19(2)	18(2)	26(2)	8(1)	6(1)	4(1)
C(18)	18(2)	20(2)	25(2)	4(1)	5(1)	5(1)
C(19)	15(2)	20(2)	23(2)	2(1)	6(1)	4(1)
C(20)	12(1)	21(2)	17(1)	1(1)	5(1)	0(1)
C(21)	12(1)	19(2)	20(1)	2(1)	8(1)	3(1)
C(22)	13(2)	23(2)	24(2)	5(1)	6(1)	6(1)
C(23)	14(2)	25(2)	31(2)	6(1)	11(1)	6(1)
C(24)	10(2)	39(2)	29(2)	5(2)	4(1)	2(1)
C(25)	16(2)	25(2)	31(2)	0(1)	8(1)	-2(1)
C(26)	13(2)	24(2)	22(2)	0(1)	5(1)	1(1)
C(27)	16(2)	19(2)	18(2)	3(1)	5(1)	0(1)
C(28)	21(2)	21(2)	24(2)	2(1)	7(1)	-1(1)

C(29)	25(2)	21(2)	35(2)	7(1)	9(2)	-2(1)
C(30)	18(2)	30(2)	30(2)	7(2)	4(1)	-4(1)
C(31)	17(2)	31(2)	26(2)	7(1)	9(1)	2(1)
C(32)	16(2)	24(2)	25(2)	6(1)	8(1)	4(1)
C(33)	16(2)	22(2)	16(1)	1(1)	4(1)	2(1)
C(34)	20(2)	20(2)	24(2)	7(1)	7(1)	6(1)
C(35)	29(2)	29(2)	22(2)	7(1)	8(1)	13(2)
C(36)	25(2)	30(2)	22(2)	5(1)	2(1)	10(2)
C(37)	21(2)	28(2)	23(2)	2(1)	3(1)	2(1)
C(38)	16(2)	24(2)	18(2)	2(1)	4(1)	2(1)

3.5 Crystal Structure of [Au($^{Cy}Y_S$-P*i*Pr$_2$)Cl] (P4)

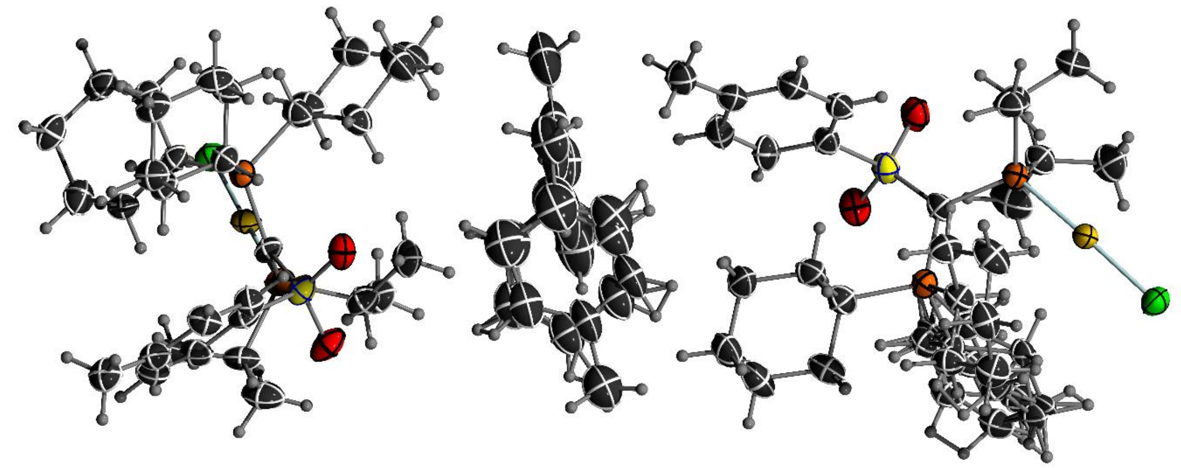

Figure 47. ORTEP plot of **P4** ^{Cy}**Y$_S$-P*i*Pr$_2$-AuCl** crystallizes as a twin with two molecules of toluene**;** thermal displacement ellipsoids shown at the 50% probability level. Selected bond lengths [Å] and angles [°] (taken only from one molecule of the asymmetric unit): P(1)-C(1) 1.785(13), P(2)-C(1) 1.788(13), S(1)-C(1) 1.746(13), S(1)-O(1) 1.455(13), Au(1)-P(2) 2.262(4), Au(1)-Cl(1) 2.310(4), Au(1)-C(9) 3.318(14); P(1)-C(1)-P(2) 124.8(7), S(1)-C(1)-P(1) 118.3(7), S(1)-C(1)-P(2) 115.1(7), P(2)-Au(1)-Cl(1) 178.43(14), P(2)-Au(1)-C(9) 80.0(3), Cl(1)-Au(1)-C(9) 99.2(3).

Table 13. Atomic Coordinates (×10^4) and Equivalent Isotropic Displacement Parameters (Å^2×10^3) for **P4**. U_{eq} is defined as 1/3 of of the trace of the orthogonalised U_{ij} tensor.

Atom	*x*	*y*	*z*	U(eq)
Au(1)	5439(1)	255(1)	1841(1)	36(1)
Au(2)	-250(1)	10122(1)	6871(1)	38(1)
Cl(1)	7579(4)	-839(3)	2495(3)	46(1)
Cl(2)	-2397(4)	11202(3)	6883(3)	51(1)

S(1)	2175(4)	3217(3)	-339(2)	40(1)
S(2)	2991(4)	7164(3)	6450(2)	41(1)
P(1)	5418(4)	2392(3)	-298(2)	35(1)
P(2)	3377(4)	1362(3)	1192(2)	37(1)
P(3)	-251(4)	8017(3)	6019(2)	35(1)
P(4)	1811(4)	9016(3)	6878(2)	37(1)
O(1)	2552(13)	4223(9)	-554(7)	46(2)
O(2)	976(12)	2998(10)	232(7)	50(3)
O(3)	4184(13)	7367(10)	6896(8)	51(3)
O(4)	2597(14)	6171(9)	6823(7)	47(2)
C(1)	3669(14)	2251(10)	143(8)	32(2)
C(2)	1581(15)	3153(12)	-1364(9)	38(3)
C(3)	1185(16)	2259(13)	-1400(10)	43(3)
C(4)	731(17)	2228(12)	-2197(11)	44(3)
C(5)	655(16)	3065(12)	-2983(10)	42(3)
C(6)	1021(17)	3962(12)	-2915(11)	44(3)
C(7)	1482(17)	4008(12)	-2111(10)	41(3)
C(8)	240(20)	2997(15)	-3846(12)	50(4)
C(9)	6456(15)	1140(12)	-213(9)	38(3)
C(10)	5670(17)	482(13)	-573(13)	48(4)
C(11)	6480(20)	-620(13)	-226(12)	54(4)
C(12)	8074(19)	-674(14)	-519(11)	50(4)
C(13)	8841(18)	18(15)	-225(11)	51(4)
C(14)	8022(15)	1120(12)	-538(10)	39(3)
C(15)	5299(16)	3261(11)	-1467(9)	38(3)
C(16)	4937(16)	2752(11)	-2114(9)	38(3)
C(17)	4780(18)	3507(15)	-3069(10)	46(4)
C(18)	6167(18)	3939(13)	-3329(10)	47(3)
C(19)	6558(18)	4451(13)	-2684(11)	48(3)
C(20)	6722(17)	3707(12)	-1734(10)	42(3)
C(21)	6590(17)	2927(13)	292(10)	44(3)
C(22)	7150(20)	2226(15)	1213(12)	49(4)
C(23)	8190(20)	2726(16)	1612(11)	55(4)
C(24)	7470(30)	3775(18)	1597(13)	65(5)
C(25)	6950(30)	4460(17)	661(13)	62(5)
C(26)	5860(20)	3986(13)	258(12)	48(4)
C(27)	2576(17)	2025(13)	1970(10)	45(3)
C(28)	3030(20)	3040(17)	1817(12)	58(4)
C(29)	3040(20)	1349(17)	2935(10)	55(4)
C(30)	1937(15)	632(13)	1079(10)	44(3)
C(31)	2524(17)	-134(14)	640(12)	52(4)
C(32)	1370(20)	54(18)	1968(13)	62(5)
C(33)	1532(16)	8125(11)	6356(9)	37(3)
C(34)	3617(15)	7216(12)	5389(10)	39(3)
C(35)	3966(16)	8111(12)	4803(10)	40(3)
C(36)	4468(18)	8124(13)	3982(12)	48(3)

C(37)	4634(17)	7244(13)	3757(12)	46(3)
C(38)	4310(20)	6355(13)	4372(12)	51(4)
C(39)	3806(18)	6338(12)	5175(10)	44(3)
C(40)	5110(20)	7259(18)	2862(13)	58(5)
C(41)	-157(17)	7125(12)	5403(10)	41(3)
C(42)	-1610(20)	6722(14)	5386(12)	50(4)
C(43)	-1420(20)	5931(14)	4913(14)	62(5)
C(44)	-960(20)	6356(17)	3996(14)	60(5)
C(45)	460(20)	6760(20)	4009(17)	68(6)
C(46)	263(18)	7566(14)	4447(11)	48(3)
C(47A)	-1480(20)	7502(13)	6909(13)	35(4)
C(48A)	-2020(20)	8224(16)	7410(13)	37(5)
C(49A)	-3010(30)	7770(17)	8129(15)	38(6)
C(50A)	-2290(30)	6729(15)	8752(15)	49(5)
C(51A)	-1770(30)	6002(17)	8261(15)	48(5)
C(52A)	-800(40)	6430(20)	7520(20)	40(8)
C(47B)	-1060(40)	7610(30)	7112(18)	35(8)
C(48B)	-2700(40)	7980(30)	7110(20)	44(11)
C(49B)	-3240(60)	7660(40)	8040(30)	41(12)
C(50B)	-2790(40)	6520(30)	8530(30)	50(8)
C(51B)	-1150(50)	6160(30)	8530(30)	45(8)
C(52B)	-590(80)	6470(40)	7610(40)	35(10)
C(53)	-1257(16)	9270(11)	5333(9)	39(3)
C(54)	-435(19)	9876(12)	4580(11)	46(3)
C(55)	-1220(20)	10996(14)	4239(13)	60(5)
C(56)	-2790(20)	11065(13)	3907(12)	51(4)
C(57)	-3620(20)	10392(14)	4617(11)	53(4)
C(58)	-2803(17)	9285(13)	4984(10)	44(3)
C(59)	3267(17)	9730(13)	6335(11)	45(3)
C(60)	2725(18)	10510(14)	5433(11)	50(4)
C(61)	3830(20)	10295(16)	6882(15)	54(5)
C(62)	2565(18)	8333(14)	8057(11)	48(4)
C(63)	2130(30)	7331(19)	8486(12)	69(6)
C(64)	2110(20)	9028(19)	8604(13)	64(5)
C11	5830(40)	3840(20)	3964(19)	87(8)
C21	4830(50)	4466(18)	3250(16)	92(10)
C31	3320(70)	4500(30)	3280(20)	127(18)
C41	2860(50)	3910(30)	3980(20)	114(13)
C51	3740(40)	3290(20)	4680(20)	98(9)
C61	5260(40)	3240(20)	4686(17)	85(8)
C71	7400(50)	3850(30)	3940(30)	120(13)
C1A2	-400(60)	6600(40)	1270(40)	68(8)
C2A2	320(50)	7260(40)	1490(40)	71(9)
C3A2	1810(50)	7170(50)	1390(60)	80(8)
C4A2	2590(60)	6450(50)	1120(60)	80(13)
C5A2	1850(70)	5860(100)	850(110)	81(12)

C6A2	360(60)	5930(50)	900(60)	69(9)
C7A2	-2020(50)	6670(40)	1350(30)	80(11)
C1B2	2330(60)	6610(60)	980(60)	77(13)
C2B2	1870(80)	5860(100)	730(110)	78(11)
C3B2	480(60)	5670(50)	920(60)	72(10)
C4B2	-510(70)	6260(40)	1240(40)	74(10)
C5B2	-20(60)	6950(40)	1510(40)	74(9)
C6B2	1400(60)	7120(60)	1430(60)	78(9)
C7B2	3880(50)	6760(40)	870(30)	79(12)

Table 14. Anisotropic displacement parameters (Å^2×10^3) for **P4**. The anisotropic displacement factor exponent takes the form: $-2\pi^2[h^2 a^{*2}U^{11} + ... + 2 h k a^* b^* U^{12}]$

Atom	U^{11}	U^{22}	U^{33}	U^{23}	U^{13}	U^{12}
Au(1)	33(1)	45(1)	31(1)	-10(1)	2(1)	-12(1)
Au(2)	32(1)	50(1)	42(1)	-25(1)	6(1)	-14(1)
Cl(1)	38(2)	55(2)	42(2)	-13(2)	-3(1)	-5(1)
Cl(2)	36(2)	67(2)	57(2)	-34(2)	4(1)	-3(2)
S(1)	40(2)	46(2)	32(2)	-12(1)	3(1)	-3(1)
S(2)	44(2)	42(2)	36(2)	-15(1)	-5(1)	-3(1)
P(1)	35(2)	42(2)	28(2)	-12(1)	3(1)	-10(1)
P(2)	34(2)	46(2)	30(2)	-10(1)	2(1)	-10(1)
P(3)	38(2)	36(2)	30(2)	-11(1)	2(1)	-8(1)
P(4)	33(2)	47(2)	38(2)	-22(2)	6(1)	-10(1)
O(1)	56(6)	46(6)	41(5)	-21(5)	3(5)	-5(5)
O(2)	43(5)	66(7)	32(5)	-11(5)	6(4)	3(5)
O(3)	49(6)	63(7)	44(6)	-28(5)	-5(5)	-2(5)
O(4)	60(6)	39(5)	38(5)	-11(4)	-4(5)	-4(5)
C(1)	30(6)	38(7)	26(6)	-9(5)	3(4)	-5(5)
C(2)	32(6)	50(8)	28(6)	-9(5)	7(5)	-8(5)
C(3)	40(7)	54(9)	37(7)	-13(6)	5(6)	-19(6)
C(4)	40(7)	41(8)	48(8)	-10(6)	3(6)	-13(6)
C(5)	33(6)	50(8)	44(8)	-15(6)	3(6)	-9(6)
C(6)	43(7)	37(8)	43(8)	-4(6)	-3(6)	-8(6)
C(7)	40(7)	40(8)	40(7)	-13(6)	-2(6)	-4(6)
C(8)	53(9)	55(10)	40(8)	-13(7)	4(7)	-11(7)
C(9)	37(7)	46(8)	32(6)	-15(5)	9(5)	-5(5)
C(10)	35(7)	54(9)	64(10)	-29(8)	13(7)	-15(6)
C(11)	65(10)	42(9)	53(9)	-15(7)	14(8)	-11(7)
C(12)	50(9)	47(9)	45(8)	-13(7)	4(7)	6(7)
C(13)	42(8)	71(11)	41(8)	-25(7)	4(6)	-2(7)
C(14)	29(6)	51(8)	38(7)	-16(6)	3(5)	-5(5)
C(15)	42(7)	42(7)	32(7)	-11(6)	5(5)	-12(6)
C(16)	40(7)	44(8)	30(7)	-12(6)	5(5)	-8(6)
C(17)	46(8)	62(10)	29(7)	-11(7)	0(6)	-14(7)

C(18)	49(8)	56(9)	30(7)	-6(6)	9(6)	-14(7)
C(19)	43(8)	50(9)	44(8)	-6(7)	9(6)	-15(7)
C(20)	42(7)	49(8)	36(7)	-11(6)	3(6)	-18(6)
C(21)	44(7)	54(9)	39(7)	-19(6)	-2(6)	-16(6)
C(22)	44(9)	56(10)	45(9)	-12(7)	3(7)	-17(7)
C(23)	50(9)	84(13)	34(7)	-17(8)	5(6)	-29(9)
C(24)	73(12)	85(14)	51(10)	-28(9)	4(9)	-42(11)
C(25)	87(14)	64(12)	46(9)	-20(8)	2(9)	-39(11)
C(26)	56(9)	48(9)	45(8)	-16(7)	6(7)	-23(7)
C(27)	42(7)	53(9)	39(8)	-14(6)	5(6)	-6(6)
C(28)	64(10)	80(13)	43(9)	-35(9)	8(8)	-16(9)
C(29)	48(8)	84(13)	30(7)	-18(7)	2(6)	-10(8)
C(30)	30(6)	56(9)	44(8)	-9(6)	0(6)	-17(6)
C(31)	36(7)	66(10)	56(9)	-17(8)	-5(6)	-24(7)
C(32)	54(12)	77(14)	53(11)	-11(9)	9(8)	-33(10)
C(33)	39(7)	45(8)	30(6)	-16(6)	-6(5)	-8(6)
C(34)	36(7)	46(8)	38(7)	-20(6)	-1(5)	-5(6)
C(35)	38(7)	43(8)	44(7)	-19(6)	-3(6)	-9(6)
C(36)	45(8)	51(9)	51(9)	-21(7)	4(7)	-14(7)
C(37)	36(7)	58(9)	53(9)	-28(7)	4(6)	-13(6)
C(38)	57(9)	45(9)	56(9)	-26(7)	0(7)	-6(7)
C(39)	51(8)	37(7)	40(8)	-14(6)	-1(6)	-1(6)
C(40)	61(11)	72(12)	52(10)	-33(9)	7(8)	-21(9)
C(41)	43(7)	40(8)	40(7)	-13(6)	-2(6)	-7(6)
C(42)	54(9)	52(9)	47(9)	-11(7)	-7(7)	-24(7)
C(43)	62(11)	42(9)	80(13)	-22(9)	-30(9)	-4(8)
C(44)	59(10)	70(12)	66(12)	-41(10)	-10(9)	-11(9)
C(45)	46(9)	102(17)	82(14)	-66(13)	4(9)	-12(10)
C(46)	42(7)	65(10)	47(8)	-27(7)	3(6)	-18(7)
C(47A)	40(10)	40(8)	30(8)	-15(6)	7(8)	-16(7)
C(48A)	39(11)	47(10)	32(9)	-20(7)	4(7)	-14(8)
C(49A)	34(9)	55(9)	38(9)	-26(7)	3(7)	-24(7)
C(50A)	50(12)	60(10)	41(10)	-17(7)	9(8)	-23(8)
C(51A)	56(12)	47(10)	40(10)	-9(7)	6(9)	-20(9)
C(52A)	46(13)	44(8)	33(10)	-13(7)	0(10)	-12(8)
C(47B)	41(15)	42(12)	25(14)	-11(10)	8(12)	-17(11)
C(48B)	41(16)	60(20)	35(15)	-16(14)	6(13)	-9(14)
C(49B)	41(17)	59(14)	32(13)	-22(12)	3(13)	-18(13)
C(50B)	50(15)	61(14)	43(17)	-14(12)	3(13)	-24(11)
C(51B)	52(14)	45(16)	38(13)	-8(11)	4(12)	-21(12)
C(52B)	43(18)	40(11)	30(13)	-17(10)	-7(13)	-18(11)
C(53)	41(7)	40(7)	32(7)	-8(5)	-6(5)	-6(6)
C(54)	48(8)	40(8)	44(8)	-7(6)	6(6)	-12(6)
C(55)	81(13)	43(9)	51(9)	-5(7)	-11(9)	-21(8)
C(56)	67(11)	40(8)	46(9)	-14(6)	-9(8)	-4(7)
C(57)	50(9)	58(10)	42(8)	-14(7)	-12(7)	3(7)

C(58)	41(8)	50(9)	40(8)	-14(6)	-3(6)	-10(6)
C(59)	39(7)	55(9)	53(9)	-28(7)	7(6)	-18(6)
C(60)	45(8)	64(10)	48(8)	-22(7)	13(7)	-26(7)
C(61)	44(10)	59(11)	69(12)	-28(9)	8(8)	-22(8)
C(62)	44(8)	59(10)	43(8)	-22(7)	-4(6)	-7(7)
C(63)	80(13)	96(16)	34(8)	-15(9)	-7(8)	-36(12)
C(64)	63(11)	94(15)	42(9)	-34(10)	-5(8)	-13(10)
C11	130(20)	74(15)	85(18)	-52(14)	25(16)	-27(15)
C21	180(40)	48(12)	48(11)	-24(9)	5(16)	-15(16)
C31	220(50)	80(20)	66(18)	-37(16)	-50(30)	20(30)
C41	160(30)	100(20)	80(20)	-46(18)	-40(20)	0(20)
C51	130(30)	100(20)	70(15)	-29(14)	-2(16)	-34(19)
C61	140(30)	63(14)	53(12)	-24(10)	0(14)	-7(15)
C71	160(40)	100(20)	130(30)	-80(20)	10(30)	-30(20)
C1A2	97(14)	60(20)	56(13)	-29(15)	-7(12)	-21(12)
C2A2	101(17)	60(20)	58(16)	-28(16)	-12(15)	-23(14)
C3A2	101(17)	63(15)	69(16)	-12(13)	-12(17)	-21(15)
C4A2	108(18)	60(20)	50(30)	0(20)	-15(19)	-7(16)
C5A2	109(13)	70(15)	50(30)	-10(17)	3(12)	-15(12)
C6A2	110(13)	50(30)	48(14)	-10(20)	2(12)	-18(13)
C7A2	98(15)	70(30)	70(30)	-20(20)	-1(17)	-18(17)
C1B2	105(17)	60(20)	50(30)	0(20)	-15(19)	-8(16)
C2B2	109(14)	69(15)	50(30)	-12(17)	4(13)	-18(12)
C3B2	109(14)	50(30)	48(14)	-10(20)	0(12)	-15(14)
C4B2	97(16)	70(20)	60(16)	-24(17)	-6(14)	-19(14)
C5B2	102(16)	70(20)	57(14)	-23(15)	-7(14)	-21(14)
C6B2	101(18)	61(16)	67(15)	-15(13)	-8(16)	-19(15)
C7B2	105(18)	80(30)	50(30)	-20(20)	-18(18)	-11(18)

3.6 Crystal Structure of $^{Cy}Y_{SF}$-H

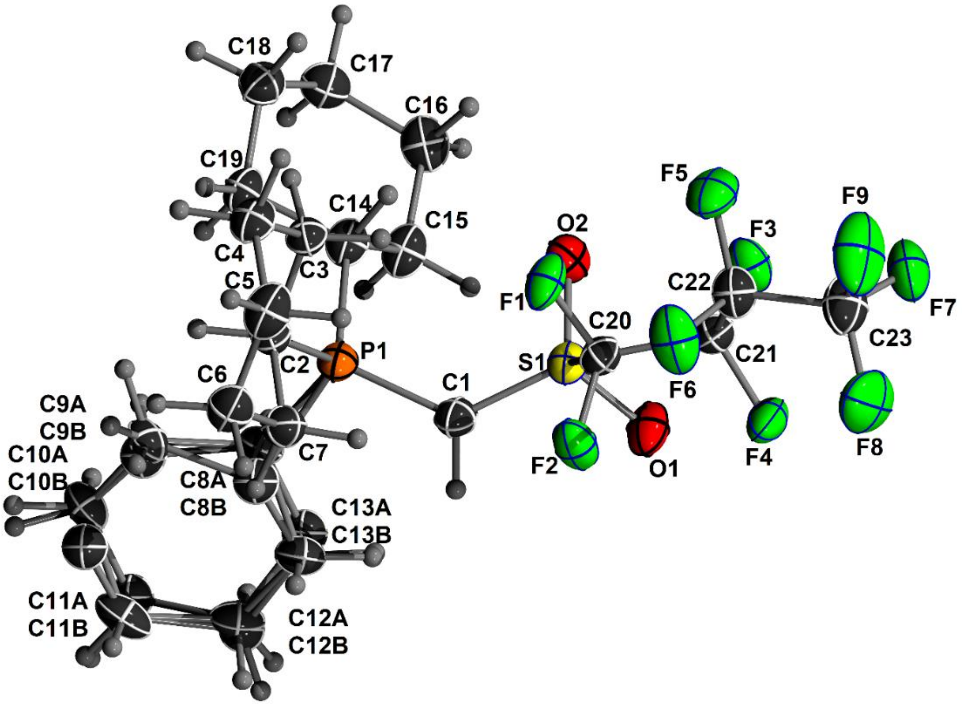

Figure 48. ORTEP plot of **$^{Cy}Y_{SF}$-H;** thermal displacement ellipsoids shown at the 50% probability level. Selected bond lengths [Å] and angles [°]: P(1)-C(1) 1.733(2), S(1)-C(1) 1.639(2), S(1)-O(1) 1.4400(16), S(1)-C(20) 1.864(2), F(1)-C(20) 1.355(2); S(1)-C(1)-P(1)121.49(9), F(1)-C(20)-S(1) 109.77(14).

Table 15. Atomic Coordinates (×10^4) and Equivalent Isotropic Displacement Parameters (Å^2×10^3) for **$^{Cy}Y_{SF}$-H**. U_{eq} is defined as 1/3 of of the trace of the orthogonalised U_{ij} tensor.

Atom	*x*	*y*	*z*	U(eq)
S(1)	3693(1)	3486(1)	6401(1)	29(1)
F(1)	4928(1)	3371(1)	5924(1)	39(1)
O(1)	3369(1)	2670(2)	6788(1)	40(1)
P(1)	3771(1)	6037(1)	6406(1)	28(1)
C(1)	3747(1)	4713(2)	6730(1)	31(1)
C(2)	4644(1)	6522(2)	6262(1)	28(1)
O(2)	3499(1)	3553(2)	5776(1)	35(1)
F(2)	4881(1)	3026(1)	6893(1)	42(1)
F(3)	4101(1)	1333(1)	5806(1)	39(1)
C(3)	4940(1)	6052(2)	5669(1)	28(1)
C(4)	5679(1)	6426(2)	5581(1)	33(1)
F(4)	4426(1)	1018(1)	6736(1)	43(1)
C(5)	6119(2)	6115(3)	6125(2)	40(1)
F(5)	5312(1)	1395(2)	5389(1)	48(1)
C(6)	5834(2)	6610(3)	6704(1)	38(1)
F(6)	5777(1)	1661(2)	6267(1)	52(1)
C(7)	5098(2)	6231(3)	6806(1)	33(1)
F(7)	4832(1)	-666(2)	5837(1)	53(1)

F(8)	5453(1)	-399(2)	6625(1)	68(1)
F(9)	5910(1)	-435(2)	5747(1)	75(1)
C(14)	3309(1)	6081(2)	5690(1)	30(1)
C(15)	2571(1)	5659(3)	5733(1)	35(1)
C(16)	2289(1)	5533(3)	5092(1)	39(1)
C(17)	2313(2)	6642(3)	4747(1)	39(1)
C(18)	3027(2)	7145(3)	4747(1)	37(1)
C(19)	3313(1)	7248(2)	5389(1)	32(1)
C(20)	4559(1)	2852(2)	6361(1)	29(1)
C(21)	4570(1)	1582(2)	6226(1)	29(1)
C(22)	5259(1)	1151(2)	5980(1)	35(1)
C(23)	5361(2)	-120(3)	6052(2)	45(1)
C(8)	3340(2)	7014(3)	6925(2)	46(1)
C(9)	3610(2)	8207(3)	6902(1)	37(1)
C(10)	3218(2)	9007(3)	7302(2)	53(1)
C(11)	3126(2)	8592(3)	7921(2)	61(1)
C(12)	2834(2)	7406(3)	7953(1)	39(1)
C(13)	3234(2)	6594(3)	7549(2)	54(1)

Table 16. Anisotropic displacement parameters ($Å^2 \times 10^3$) for **$^{Cy}Y_{SF}$-H**. The anisotropic displacement factor exponent takes the form: $-2\pi^2[h^2 a^{*2}U^{11} + ... + 2 h k a^* b^* U^{12}]$

Atom	U^{11}	U^{22}	U^{33}	U^{23}	U^{13}	U^{12}
S(1)	25(1)	27(1)	33(1)	1(1)	4(1)	1(1)
F(1)	31(1)	31(1)	57(1)	11(1)	13(1)	0(1)
O(1)	36(1)	32(1)	52(1)	3(1)	16(1)	-2(1)
P(1)	28(1)	27(1)	29(1)	0(1)	7(1)	1(1)
C(1)	35(2)	30(1)	29(1)	1(1)	7(1)	2(1)
C(2)	29(1)	26(1)	27(1)	0(1)	3(1)	-1(1)
O(2)	33(1)	34(1)	38(1)	-3(1)	-4(1)	6(1)
F(2)	45(1)	35(1)	46(1)	-6(1)	-18(1)	5(1)
F(3)	29(1)	38(1)	51(1)	-10(1)	-9(1)	0(1)
C(3)	28(1)	28(1)	28(1)	0(1)	5(1)	-1(1)
C(4)	29(1)	34(2)	37(2)	2(1)	7(1)	0(1)
F(4)	58(1)	31(1)	40(1)	8(1)	15(1)	3(1)
C(5)	26(1)	40(2)	53(2)	9(1)	0(1)	0(1)
F(5)	50(1)	49(1)	46(1)	6(1)	19(1)	2(1)
C(6)	33(2)	40(2)	41(2)	6(1)	-11(1)	-5(1)
F(6)	28(1)	43(1)	84(1)	-9(1)	-14(1)	2(1)
C(7)	35(2)	36(2)	29(1)	1(1)	-1(1)	-2(1)
F(7)	56(1)	33(1)	71(1)	-11(1)	10(1)	-6(1)
F(8)	86(2)	46(1)	72(1)	12(1)	-10(1)	24(1)
F(9)	55(1)	44(1)	125(2)	-4(1)	38(1)	14(1)
C(14)	24(1)	29(1)	38(1)	3(1)	5(1)	2(1)
C(15)	23(1)	35(2)	48(2)	7(1)	4(1)	2(1)
C(16)	26(1)	38(2)	51(2)	-4(1)	0(1)	1(1)
C(17)	35(2)	42(2)	39(2)	-1(1)	-3(1)	3(1)
C(18)	39(2)	35(2)	36(2)	3(1)	1(1)	2(1)

C(19)	30(1)	29(1)	38(2)	4(1)	2(1)	1(1)
C(20)	28(1)	29(1)	30(1)	3(1)	0(1)	-2(1)
C(21)	28(1)	30(1)	28(1)	4(1)	1(1)	-1(1)
C(22)	28(1)	33(2)	44(2)	-1(1)	0(1)	0(1)
C(23)	42(2)	34(2)	60(2)	-2(2)	8(2)	6(1)
C(8)	51(2)	36(2)	51(2)	-7(1)	23(2)	1(1)
C(9)	39(2)	34(2)	39(2)	-6(1)	4(1)	-1(1)
C(10)	49(2)	39(2)	69(2)	-15(2)	18(2)	-1(2)
C(11)	76(3)	57(2)	49(2)	-22(2)	10(2)	4(2)
C(12)	37(2)	51(2)	29(1)	-5(1)	3(1)	6(1)
C(13)	77(3)	45(2)	39(2)	-5(2)	20(2)	-1(2)

3.7 Crystal Structure of $^{Cy}Y_{SF}$-PCy_2 (L5)

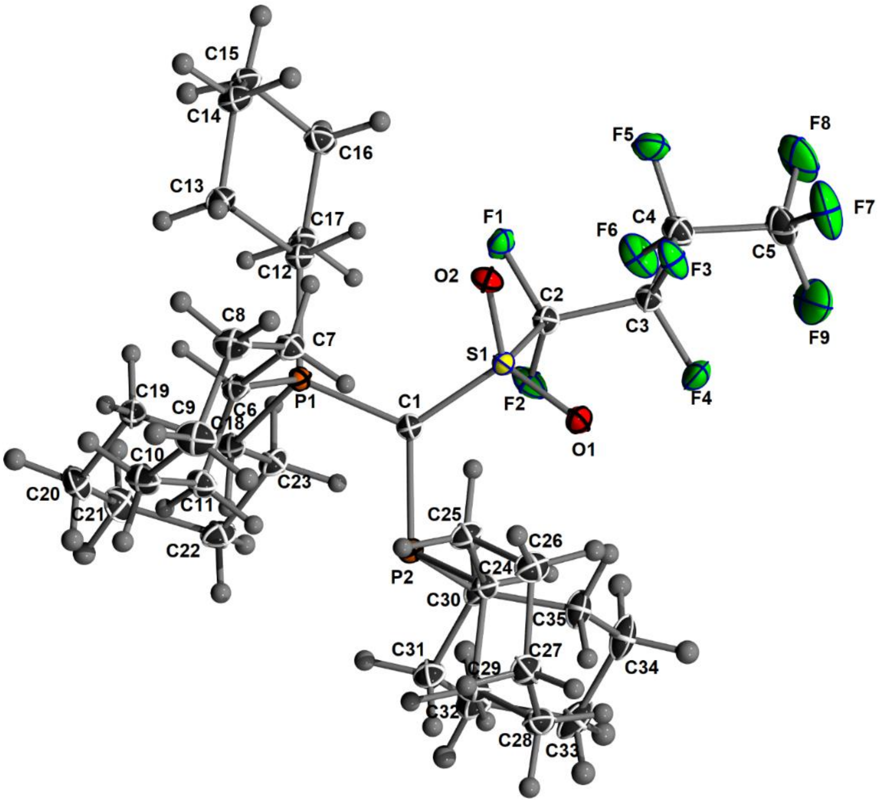

Figure 49. ORTEP plot of **L5;** thermal displacement ellipsoids shown at the 50% probability level. Selected bond lengths [Å] and angles [°]: P(1)-C(1) 1.7688(11), P(2)-C(1) 1.8248(11), S(1)-C(1) 1.6809(11), S(1)-O(1) 1.4402(8), S(1)-C(2) 1.8862(11), F(1)-C(2) 1.3513(13); P(1)-C(1)-P(2) 113.97(6), S(1)-C(1)-P(1) 122.73(6), S(1)-C(1)-P(2) 122.55(6), F(1)-C(2)-S(1) 109.58(7).

Table 17. Atomic Coordinates (×10^4) and Equivalent Isotropic Displacement Parameters (Å^2×10^3) for **L5**. U_{eq} is defined as 1/3 of of the trace of the orthogonalised U_{ij} tensor.

Atom	*x*	*y*	*z*	U(eq)
S(1)	6664(1)	8451(1)	3317(1)	13(1)
P(1)	4365(1)	7094(1)	3573(1)	11(1)
P(2)	6823(1)	6272(1)	3488(1)	13(1)
F(1)	5041(1)	8646(1)	1778(1)	20(1)
F(2)	6047(1)	7340(1)	1286(1)	23(1)
F(3)	7653(1)	10047(1)	2487(1)	25(1)
F(4)	8167(1)	8619(1)	1474(1)	29(1)
F(5)	5872(1)	10084(1)	1014(1)	28(1)
F(6)	5886(1)	8467(1)	-30(1)	30(1)
F(7)	8475(1)	10708(1)	1039(1)	46(1)
F(8)	7046(1)	10193(1)	-397(1)	45(1)
F(9)	8290(1)	9160(1)	-146(1)	52(1)
O(1)	8041(1)	8536(1)	3459(1)	19(1)
O(2)	6198(1)	9383(1)	3860(1)	18(1)
C(1)	5946(1)	7304(1)	3386(1)	13(1)
C(2)	6194(1)	8366(1)	1950(1)	15(1)
C(3)	7184(1)	9104(1)	1680(1)	17(1)
C(4)	6618(1)	9372(1)	741(1)	20(1)
C(5)	7640(2)	9868(1)	302(1)	30(1)
C(6)	4334(1)	7327(1)	4914(1)	14(1)
C(7)	5127(1)	8456(1)	5648(1)	16(1)

C(8)	4962(1)	8693(1)	6740(1)	21(1)
C(9)	5327(1)	7830(1)	7132(1)	24(1)
C(10)	4554(1)	6711(1)	6390(1)	21(1)
C(11)	4733(1)	6461(1)	5299(1)	16(1)
C(12)	3374(1)	7968(1)	3196(1)	14(1)
C(13)	2343(1)	8274(1)	3807(1)	18(1)
C(14)	1705(1)	9099(1)	3504(1)	22(1)
C(15)	1111(1)	8696(1)	2349(1)	22(1)
C(16)	2099(1)	8333(1)	1733(1)	20(1)
C(17)	2710(1)	7498(1)	2036(1)	16(1)
C(18)	3589(1)	5657(1)	2863(1)	14(1)
C(19)	2208(1)	5352(1)	2984(1)	17(1)
C(20)	1746(1)	4124(1)	2548(1)	22(1)
C(21)	1829(1)	3605(1)	1434(1)	24(1)
C(22)	3192(1)	3937(1)	1320(1)	19(1)
C(23)	3620(1)	5168(1)	1726(1)	16(1)
C(24)	8448(1)	6960(1)	4398(1)	15(1)
C(25)	8414(1)	7832(1)	5412(1)	17(1)
C(26)	9794(1)	8366(1)	6091(1)	20(1)
C(27)	10508(1)	7538(1)	6302(1)	20(1)
C(28)	10485(1)	6620(1)	5306(1)	20(1)
C(29)	9100(1)	6105(1)	4634(1)	18(1)
C(30)	7240(1)	5653(1)	2217(1)	15(1)
C(31)	7024(1)	4421(1)	1888(1)	19(1)
C(32)	7182(1)	3882(1)	795(1)	24(1)
C(33)	8509(1)	4352(1)	710(1)	26(1)
C(34)	8730(1)	5571(1)	1031(1)	25(1)
C(35)	8573(1)	6122(1)	2122(1)	20(1)

Table 18. Anisotropic displacement parameters (Å^2×10^3) for **L5**. The anisotropic displacement factor exponent takes the form: $-2\pi^2[h^2 a^{*2}U^{11} + ... + 2 h k a^* b^* U^{12}]$

Atom	U^{11}	U^{22}	U^{33}	U^{23}	U^{13}	U^{12}
S(1)	13(1)	12(1)	15(1)	6(1)	3(1)	2(1)
P(1)	12(1)	11(1)	12(1)	4(1)	3(1)	3(1)
P(2)	13(1)	12(1)	14(1)	5(1)	5(1)	4(1)
F(1)	13(1)	30(1)	23(1)	15(1)	4(1)	5(1)
F(2)	35(1)	15(1)	17(1)	5(1)	6(1)	3(1)
F(3)	26(1)	21(1)	22(1)	10(1)	-1(1)	-6(1)
F(4)	20(1)	42(1)	42(1)	28(1)	17(1)	17(1)
F(5)	31(1)	32(1)	31(1)	20(1)	11(1)	17(1)
F(6)	40(1)	27(1)	18(1)	9(1)	1(1)	-2(1)
F(7)	47(1)	46(1)	42(1)	22(1)	11(1)	-14(1)
F(8)	55(1)	57(1)	41(1)	39(1)	15(1)	10(1)
F(9)	69(1)	58(1)	62(1)	37(1)	52(1)	31(1)
O(1)	13(1)	21(1)	25(1)	13(1)	2(1)	2(1)
O(2)	21(1)	13(1)	18(1)	5(1)	4(1)	4(1)
C(1)	13(1)	13(1)	15(1)	6(1)	4(1)	3(1)
C(2)	14(1)	15(1)	17(1)	7(1)	4(1)	3(1)
C(3)	14(1)	19(1)	20(1)	10(1)	5(1)	4(1)
C(4)	23(1)	20(1)	20(1)	10(1)	6(1)	5(1)
C(5)	36(1)	32(1)	29(1)	19(1)	14(1)	7(1)
C(6)	15(1)	15(1)	13(1)	5(1)	5(1)	5(1)
C(7)	18(1)	14(1)	15(1)	4(1)	5(1)	4(1)
C(8)	28(1)	19(1)	15(1)	3(1)	5(1)	7(1)
C(9)	33(1)	25(1)	14(1)	6(1)	4(1)	9(1)
C(10)	28(1)	22(1)	16(1)	10(1)	8(1)	9(1)

C(11)	20(1)	16(1)	14(1)	6(1)	5(1)	6(1)
C(12)	14(1)	13(1)	17(1)	6(1)	4(1)	5(1)
C(13)	17(1)	19(1)	21(1)	8(1)	8(1)	8(1)
C(14)	20(1)	20(1)	31(1)	10(1)	9(1)	10(1)
C(15)	15(1)	20(1)	34(1)	14(1)	4(1)	6(1)
C(16)	18(1)	20(1)	23(1)	12(1)	1(1)	4(1)
C(17)	15(1)	16(1)	17(1)	7(1)	3(1)	4(1)
C(18)	14(1)	13(1)	15(1)	5(1)	4(1)	3(1)
C(19)	15(1)	16(1)	19(1)	7(1)	6(1)	2(1)
C(20)	22(1)	18(1)	27(1)	9(1)	6(1)	-1(1)
C(21)	22(1)	16(1)	26(1)	3(1)	4(1)	-1(1)
C(22)	20(1)	16(1)	19(1)	2(1)	3(1)	5(1)
C(23)	15(1)	16(1)	14(1)	4(1)	4(1)	3(1)
C(24)	15(1)	15(1)	15(1)	6(1)	4(1)	5(1)
C(25)	16(1)	17(1)	16(1)	4(1)	3(1)	6(1)
C(26)	18(1)	18(1)	20(1)	3(1)	2(1)	4(1)
C(27)	17(1)	22(1)	21(1)	9(1)	0(1)	2(1)
C(28)	18(1)	19(1)	24(1)	10(1)	3(1)	7(1)
C(29)	19(1)	16(1)	18(1)	6(1)	3(1)	6(1)
C(30)	14(1)	16(1)	15(1)	5(1)	5(1)	4(1)
C(31)	21(1)	17(1)	20(1)	4(1)	8(1)	5(1)
C(32)	24(1)	22(1)	21(1)	1(1)	8(1)	5(1)
C(33)	21(1)	35(1)	18(1)	2(1)	8(1)	10(1)
C(34)	18(1)	36(1)	17(1)	6(1)	8(1)	1(1)
C(35)	16(1)	24(1)	17(1)	6(1)	7(1)	1(1)

3.8 Crystal Structure of [Au($^{Cy}Y_{SF}$-PCy_2)Cl] (P5)

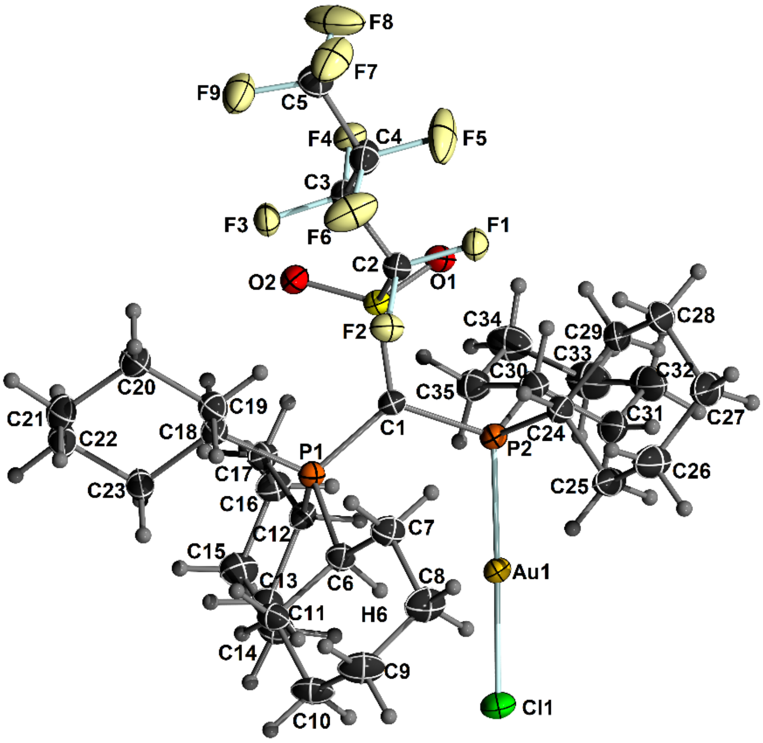

Figure 50. ORTEP plot of **P5;** thermal displacement ellipsoids shown at the 50% probability level. Selected bond lengths [Å] and angles [°]: P(1)-C(1) 1.794(4), P(2)-C(1) 1.816(4), S(1)-C(1) 1.686(4), S(1)-O(1) 1.440(3), S(1)-C(2) 1.913(4), F(1)-C(2) 1.354(4), Au(1)-P(2) 2.2632(9), Au(1)-Cl(1) 2.2863(9), Au(1)-H(6) 2.38(5), Au(1)-C(6) 3.301(4); P(1)-C(1)-P(2) 119.6(2), S(1)-C(1)-P(1) 123.0(2), S(1)-C(1)-P(2) 117.2(2) , F(1)-C(2)-S(1) 110.7(2), Au(1)-P(2) 2.2632(9), Au(1)-Cl(1) 2.2863(9), Au(1)-H(6) 2.38(5), Au(1)-C(6) 3.301(4).

Table 19. Atomic Coordinates (×10^4) and Equivalent Isotropic Displacement Parameters (Å^2×10^3) for **P5**. U_{eq} is defined as 1/3 of of the trace of the orthogonalised U_{ij} tensor.

Atom	*x*	*y*	*z*	U(eq)
Au(1)	3027(1)	2046(1)	7015(1)	24(1)
Cl(1)	2765(1)	2390(1)	7929(1)	40(1)
O(1)	4015(2)	3663(2)	4801(1)	27(1)
S(1)	3650(1)	2754(1)	5030(1)	22(1)
F(1)	2339(2)	1462(1)	4534(1)	30(1)
P(1)	2961(1)	3978(1)	6027(1)	21(1)
C(1)	3274(3)	2843(2)	5699(1)	22(1)
F(2)	1430(2)	2724(1)	4824(1)	29(1)
P(2)	3312(1)	1739(1)	6108(1)	23(1)
O(2)	4323(2)	1905(2)	4940(1)	28(1)
C(2)	2395(3)	2433(2)	4581(1)	26(1)
C(3)	2444(3)	2871(2)	3992(1)	26(1)
F(3)	2352(2)	3845(1)	4030(1)	31(1)
C(4)	1508(3)	2539(2)	3589(1)	33(1)
F(4)	3433(2)	2648(1)	3769(1)	31(1)
F(5)	524(2)	2852(2)	3780(1)	52(1)
C(5)	1638(3)	2912(2)	2990(2)	33(1)
C(6)	1667(3)	3857(2)	6428(1)	25(1)
F(6)	1484(3)	1571(2)	3557(1)	64(1)
C(7)	753(3)	3240(2)	6148(1)	29(1)
F(7)	1755(2)	3852(2)	2979(1)	46(1)
C(8)	-164(3)	3001(3)	6566(2)	38(1)
F(8)	2528(3)	2525(2)	2751(1)	67(1)
F(9)	744(2)	2677(2)	2701(1)	53(1)
C(9)	-656(3)	3912(3)	6825(2)	42(1)
C(10)	256(3)	4542(3)	7078(1)	35(1)
C(12)	4105(2)	4181(2)	6514(1)	22(1)
C(11)	1137(3)	4803(2)	6650(1)	29(1)
C(14)	4868(3)	4781(3)	7417(1)	32(1)
C(13)	3873(3)	4814(2)	7027(1)	27(1)
C(15)	5954(3)	5059(3)	7133(1)	35(1)
C(16)	6165(3)	4435(2)	6625(1)	31(1)
C(23)	2956(3)	6019(2)	5826(1)	29(1)
C(22)	2983(3)	6814(2)	5386(2)	35(1)
C(21)	1975(3)	6767(3)	5007(2)	37(1)
C(20)	1875(3)	5764(2)	4743(1)	33(1)
C(19)	1822(3)	4971(2)	5183(1)	27(1)
C(18)	2872(3)	5006(2)	5547(1)	24(1)
C(17)	5192(3)	4490(2)	6221(1)	26(1)
C(30)	4674(3)	1124(2)	6053(1)	28(1)
C(31)	4741(3)	309(2)	6489(1)	36(1)
C(32)	5852(4)	-234(3)	6439(2)	47(1)
C(33)	6832(4)	449(3)	6500(2)	49(1)
C(34)	6760(3)	1287(3)	6094(2)	43(1)
C(35)	5652(3)	1826(3)	6134(1)	32(1)
C(24)	2241(3)	887(2)	5825(1)	28(1)
C(25)	1457(3)	513(3)	6279(1)	34(1)
C(26)	469(3)	-37(3)	6023(2)	42(1)
C(27)	876(4)	-877(3)	5666(2)	44(1)
C(28)	1693(4)	-519(2)	5229(2)	39(1)
C(29)	2682(3)	30(2)	5478(1)	32(1)

Table 20. Anisotropic displacement parameters (Å^2×10^3) for **P5**. The anisotropic displacement factor exponent takes the form: $-2\pi^2[h^2 a^{*2}U^{11} + ... + 2 h k a^* b^* U^{12}]$

Atom	U^{11}	U^{22}	U^{33}	U^{23}	U^{13}	U^{12}
Au(1)	32(1)	22(1)	18(1)	1(1)	3(1)	-1(1)
Cl(1)	63(1)	35(1)	21(1)	-1(1)	7(1)	0(1)
O(1)	32(1)	25(1)	23(1)	2(1)	3(1)	-3(1)
S(1)	26(1)	22(1)	18(1)	0(1)	2(1)	-1(1)
F(1)	44(1)	20(1)	27(1)	2(1)	-3(1)	-5(1)
P(1)	22(1)	20(1)	19(1)	0(1)	0(1)	1(1)
C(1)	27(2)	23(1)	17(1)	1(1)	2(1)	0(1)
F(2)	28(1)	34(1)	24(1)	-3(1)	2(1)	-2(1)
P(2)	30(1)	20(1)	18(1)	1(1)	3(1)	-1(1)
O(2)	33(1)	29(1)	22(1)	-2(1)	4(1)	4(1)
C(2)	31(2)	23(1)	23(1)	-1(1)	3(1)	-1(1)
C(3)	31(2)	20(1)	25(2)	0(1)	0(1)	-2(1)
F(3)	44(1)	20(1)	30(1)	1(1)	-4(1)	-2(1)
C(4)	43(2)	26(2)	31(2)	4(1)	-7(1)	-7(1)
F(4)	38(1)	32(1)	24(1)	2(1)	5(1)	0(1)
F(5)	33(1)	89(2)	35(1)	10(1)	-4(1)	-11(1)
C(5)	37(2)	35(2)	28(2)	-3(1)	-5(1)	-1(1)
C(6)	24(1)	26(1)	25(1)	-1(1)	1(1)	0(1)
F(6)	111(2)	29(1)	53(1)	8(1)	-42(2)	-26(1)
C(7)	24(2)	33(2)	29(2)	-4(1)	2(1)	-3(1)
F(7)	68(2)	36(1)	35(1)	9(1)	-9(1)	-11(1)
C(8)	28(2)	47(2)	38(2)	-3(1)	5(2)	-9(1)
F(8)	77(2)	98(2)	27(1)	-9(1)	-1(1)	37(2)
F(9)	66(2)	59(1)	35(1)	10(1)	-22(1)	-25(1)
C(9)	26(2)	62(2)	38(2)	-7(2)	10(1)	-1(2)
C(10)	30(2)	48(2)	28(2)	-7(1)	5(1)	8(1)
C(12)	23(1)	24(1)	20(1)	2(1)	1(1)	-2(1)
C(11)	29(2)	30(2)	27(2)	-5(1)	2(1)	7(1)
C(14)	34(2)	36(2)	25(2)	-3(1)	-3(1)	1(1)
C(13)	32(2)	26(1)	24(1)	-3(1)	-1(1)	-1(1)
C(15)	33(2)	42(2)	30(2)	-4(1)	-6(1)	-6(1)
C(16)	28(2)	36(2)	29(2)	0(1)	0(1)	0(1)
C(23)	38(2)	21(1)	28(2)	0(1)	-3(1)	-1(1)
C(22)	42(2)	22(1)	41(2)	2(1)	-1(2)	0(1)
C(21)	48(2)	29(2)	34(2)	6(1)	-5(2)	9(1)
C(20)	43(2)	31(2)	26(2)	1(1)	-2(1)	7(1)
C(19)	30(2)	27(2)	26(1)	-2(1)	-2(1)	3(1)
C(18)	30(2)	22(1)	21(1)	1(1)	-1(1)	1(1)
C(17)	27(2)	27(2)	24(1)	0(1)	1(1)	-6(1)
C(30)	34(2)	26(2)	24(1)	2(1)	1(1)	5(1)
C(31)	50(2)	28(2)	29(2)	7(1)	4(1)	10(1)
C(32)	64(3)	40(2)	35(2)	3(2)	3(2)	24(2)
C(33)	51(2)	57(2)	40(2)	0(2)	-4(2)	26(2)
C(34)	36(2)	55(2)	38(2)	-3(2)	0(2)	12(2)
C(35)	33(2)	35(2)	28(2)	0(1)	-1(1)	3(1)
C(24)	36(2)	24(1)	24(1)	0(1)	3(1)	-6(1)
C(25)	41(2)	35(2)	26(2)	-3(1)	7(1)	-12(1)
C(26)	48(2)	44(2)	34(2)	-8(2)	8(2)	-20(2)
C(27)	63(3)	36(2)	34(2)	-5(1)	7(2)	-22(2)
C(28)	61(2)	26(2)	31(2)	-6(1)	8(2)	-13(2)
C(29)	47(2)	22(1)	28(2)	-1(1)	5(1)	-4(1)

3.9 Crystal structure of 1a

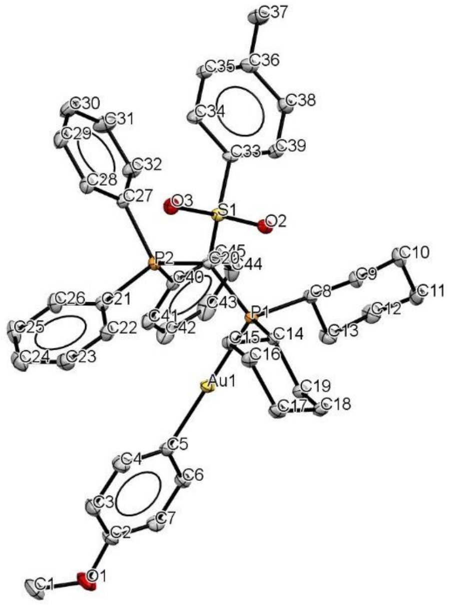

Figure 51. Molecular structure of **1a**, showing thermal displacement ellipsoids, drawn at the 50% probability level and atom labeling scheme**.**

Table 21. Fractional Atomic Coordinates (×104) and Equivalent Isotropic Displacement Parameters (Å^2×10^3) for 1a**. Ueq is defined as 1/3 of of the trace of the orthogonalised U_{IJ} tensor.**

Atom	x	y	z	U(eq)
Au1	4647.4(2)	2713.2(2)	8390.2(2)	13.35(3)
S1	713.4(4)	2776.8(3)	5859.2(3)	11.17(9)
P1	3038.7(4)	3532.9(4)	7689.2(3)	10.59(9)
P2	3091.5(4)	1704.2(3)	5907.3(3)	10.98(9)
O1	8587.7(17)	373.2(13)	10509.1(13)	32.8(4)
O2	252.4(13)	3640.5(11)	6513.1(10)	14.6(3)
O3	-48.0(13)	1756.7(11)	5449.6(10)	17.5(3)
C1	8322(3)	-727(2)	10289(2)	41.1(7)
C2	7676(2)	830.3(17)	10008.5(16)	21.1(4)
C3	6429(2)	374.8(17)	9500.0(17)	24.6(5)
C4	5588(2)	928.3(17)	9036.9(16)	23.2(4)
C5	5951(2)	1931.6(15)	9056.2(14)	15.5(4)
C6	7225(2)	2353.2(16)	9568.5(15)	17.3(4)
C7	8076(2)	1822.4(16)	10039.0(15)	19.7(4)
C8	3559.9(19)	4838.1(15)	7672.6(14)	13.5(4)
C9	2627(2)	5670.5(15)	7851.3(15)	16.1(4)
C10	3108(2)	6653.2(16)	7704.3(16)	20.8(4)
C11	4470(2)	7104.8(16)	8352.3(16)	23.8(5)

C12	5393(2)	6272.2(17)	8196.8(17)	24.1(5)
C13	4915(2)	5289.6(17)	8347.9(16)	21.5(4)
C14	1892.1(18)	3786.0(14)	8435.1(13)	12.3(4)
C15	1078.2(19)	2780.1(15)	8292.7(14)	14.6(4)
C16	131(2)	3028.9(15)	8905.7(14)	16.5(4)
C17	841(2)	3577.6(16)	9981.5(14)	18.3(4)
C18	1706(2)	4564.9(16)	10149.8(14)	17.1(4)
C19	2635(2)	4318.5(16)	9515.3(14)	16.1(4)
C20	2254.1(18)	2690.3(14)	6461.5(13)	11.2(3)
C21	3100.9(19)	560.2(14)	6258.4(14)	14.0(4)
C22	2473(2)	517.9(16)	6934.5(15)	17.1(4)
C23	2421(2)	-383.0(17)	7167.5(16)	21.7(4)
C24	2990(2)	-1239.8(17)	6716.8(18)	25.1(5)
C25	3605(2)	-1208.6(17)	6031.9(18)	25.5(5)
C26	3665(2)	-310.5(16)	5796.7(16)	20.9(4)
C27	2488.4(19)	1130.2(15)	4586.0(14)	14.5(4)
C28	1484(2)	303.7(16)	4164.1(15)	18.9(4)
C29	1066(2)	-175.3(17)	3155.7(16)	24.6(5)
C30	1655(2)	131.7(19)	2569.8(16)	29.3(5)
C31	2652(2)	943(2)	2982.2(16)	28.7(5)
C32	3064(2)	1445.9(17)	3985.9(15)	20.7(4)
C33	755.9(18)	3157.0(15)	4856.9(14)	12.8(4)
C34	220(2)	2456.7(16)	3914.6(15)	17.8(4)
C35	278(2)	2755.0(17)	3137.7(15)	21.0(4)
C36	845(2)	3741.7(17)	3284.0(15)	19.3(4)
C37	922(2)	4044.4(19)	2435.2(16)	27.2(5)
C38	1344(2)	4450.3(16)	4241.3(15)	19.1(4)
C39	1306(2)	4160.5(16)	5024.9(14)	16.6(4)
C40	4735.7(18)	2262.1(15)	6164.1(14)	13.9(4)
C41	5801(2)	1817.7(16)	6535.6(15)	18.3(4)
C42	7030(2)	2294.9(18)	6707.7(17)	26.4(5)
C43	7207(2)	3204.6(18)	6517.8(19)	29.4(5)
C44	6154(2)	3656.7(17)	6158.2(17)	24.7(5)
C45	4918(2)	3188.5(16)	5980.2(15)	17.6(4)

Table 22. Anisotropic displacement parameters (Å^2×10^3) for **1a**. The anisotropic displacement factor exponent takes the form: $-2\pi^2[h^2a^{*2}U_{11}+2hka^*b^*U_{12}+\ldots]$.

Atom	U11	U22	U33	U23	U13	U12
Au1	12.79(5)	15.21(4)	13.20(5)	6.68(3)	3.13(3)	4.72(3)
S1	9.2(2)	12.7(2)	12.5(2)	5.62(16)	3.11(16)	1.03(16)
P1	10.4(2)	11.5(2)	10.9(2)	5.12(17)	3.02(17)	2.42(17)
P2	10.5(2)	10.8(2)	12.6(2)	4.23(17)	4.54(18)	1.64(16)
O1	30.4(9)	26.2(8)	40.0(10)	19.2(7)	-3.9(8)	9.0(7)
O2	12.3(7)	18.8(7)	15.0(6)	7.2(5)	5.7(5)	5.6(5)
O3	13.7(7)	17.2(7)	20.3(7)	7.1(6)	2.5(6)	-3.8(5)

C1	48.8(17)	26.3(12)	47.5(16)	21.4(12)	0.0(13)	14.4(12)
C2	23.3(11)	21.2(10)	20.5(10)	11.1(8)	3.2(9)	9.5(8)
C3	26.5(12)	16.5(10)	29.7(12)	11.8(9)	1.7(9)	2.3(9)
C4	19.0(11)	21.1(10)	26.5(11)	10.3(9)	-1.0(9)	1.0(8)
C5	18.1(10)	16.6(9)	13.1(9)	6.2(7)	4.8(8)	5.6(8)
C6	17.4(10)	17.9(9)	18.3(10)	7.8(8)	5.9(8)	3.9(8)
C7	13.8(10)	22.0(10)	21.6(10)	7.9(8)	1.7(8)	2.4(8)
C8	13.4(9)	13.9(9)	13.8(9)	5.9(7)	3.8(7)	0.9(7)
C9	17.7(10)	12.9(9)	19.1(10)	6.8(7)	6.2(8)	2.9(7)
C10	24.2(11)	14.8(9)	27.4(11)	10.1(8)	10.3(9)	4.1(8)
C11	31.2(13)	15.8(9)	22.3(11)	5.6(8)	6.6(9)	-4.6(9)
C12	18.2(11)	23.9(11)	28.6(11)	11.7(9)	1.6(9)	-6.1(8)
C13	15.6(10)	21.9(10)	25.8(11)	11.8(9)	-0.2(9)	-2.5(8)
C14	13.0(9)	12.5(8)	12.7(9)	5.6(7)	4.3(7)	3.2(7)
C15	17.1(10)	14.6(9)	14.4(9)	6.5(7)	6.5(8)	2.5(7)
C16	17.7(10)	17.7(9)	16.6(9)	6.8(8)	8.3(8)	2.0(8)
C17	20.8(11)	23.3(10)	15.1(9)	9.5(8)	8.4(8)	5.8(8)
C18	19.0(10)	19.5(9)	11.7(9)	3.6(7)	4.9(8)	4.6(8)
C19	16.1(10)	18.5(9)	13.6(9)	5.9(7)	3.7(8)	2.6(7)
C20	10.2(9)	12.4(8)	11.2(8)	4.5(7)	2.8(7)	2.1(7)
C21	13.0(9)	10.9(8)	17.6(9)	5.1(7)	3.6(7)	1.5(7)
C22	17.1(10)	15.8(9)	18.2(10)	4.8(8)	6.1(8)	1.0(8)
C23	23.5(11)	22.2(10)	21.6(10)	10.9(8)	5.9(9)	-1.7(8)
C24	24.2(12)	17.6(10)	35.8(12)	15.2(9)	4.5(10)	1.7(8)
C25	22.2(11)	15.1(10)	40.7(13)	10.2(9)	10.7(10)	7.0(8)
C26	20.7(11)	17.4(10)	26.7(11)	7.2(8)	10.8(9)	4.7(8)
C27	13.9(9)	15.1(9)	13.8(9)	3.3(7)	4.7(8)	5.3(7)
C28	18.3(10)	15.5(9)	20.4(10)	3.9(8)	4.9(8)	1.5(8)
C29	22.8(11)	18.1(10)	23.1(11)	-1.2(8)	1.6(9)	1.2(8)
C30	34.1(13)	30.6(12)	14.5(10)	-0.8(9)	4.1(9)	4.8(10)
C31	28.8(13)	40.4(13)	18.0(11)	9.1(10)	10.5(9)	2.9(10)
C32	18.1(11)	25.7(10)	18.1(10)	6.9(8)	6.1(8)	1.4(8)
C33	9.8(9)	17.3(9)	12.7(9)	7.5(7)	2.5(7)	3.3(7)
C34	16.1(10)	17.4(9)	17.1(10)	6.2(8)	0.1(8)	1.5(8)
C35	25.1(11)	21.4(10)	12.5(9)	4.4(8)	0.9(8)	5.5(8)
C36	19.3(10)	26.1(10)	15.9(10)	10.7(8)	6.0(8)	7.4(8)
C37	36.6(14)	32.1(12)	17.4(10)	12.7(9)	9.7(10)	6.0(10)
C38	21.0(11)	19.0(10)	19.2(10)	8.6(8)	6.5(8)	0.2(8)
C39	17.1(10)	18.1(9)	12.7(9)	4.3(7)	2.9(8)	0.9(8)
C40	11.0(9)	14.9(9)	14.3(9)	1.9(7)	5.7(7)	1.3(7)
C41	16.2(10)	18.0(9)	18.4(10)	3.2(8)	5.3(8)	4.0(8)
C42	13.3(10)	26.1(11)	31.1(12)	0.9(9)	4.0(9)	4.7(9)
C43	13.6(11)	26.7(11)	41.1(14)	0.5(10)	12.4(10)	-3.9(9)
C44	23.4(12)	16.8(10)	35.0(12)	4.8(9)	16.6(10)	-1.3(8)
C45	17.2(10)	17.0(9)	20.0(10)	4.5(8)	10.0(8)	2.7(8)

3.10 Crystal structure of 1b

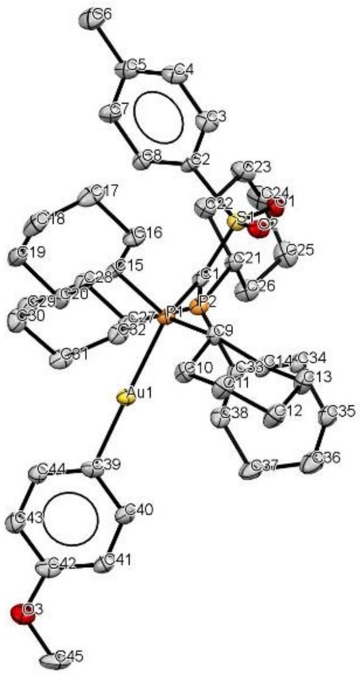

Figure 52. Molecular structure of **1b**, showing thermal displacement ellipsoids at the 50% probability level and atom labeling scheme.

Table 23. Fractional Atomic Coordinates (×10^{4}) and Equivalent Isotropic Displacement Parameters (Å^{2}×10^{3}) for 1a. Ueq is defined as 1/3 of of the trace of the orthogonalised U_{IJ} tensor.

Atom	x	y	z	U(eq)
Au1	1888.5(2)	5384.2(2)	4522.8(2)	19.08(7)
S1	2994.1(14)	6850.5(6)	3072.5(12)	18.9(3)
P1	1893.3(15)	5961.0(6)	3327.3(13)	16.8(4)
P2	3904.5(16)	6308.4(7)	5136.7(13)	18.6(4)
O1	3634(4)	7219.5(18)	3685(4)	24.6(11)
O2	1836(4)	6955.9(19)	2453(4)	23.4(12)
O3	1992(5)	3869(2)	7870(4)	35.2(14)
C1	2980(6)	6385(2)	3834(5)	18.3(15)
C2	3707(6)	6719(3)	2096(5)	18.3(15)
C3	3423(6)	6978(3)	1172(6)	25.9(17)
C4	4011(7)	6908(3)	427(6)	28.4(18)
C5	4856(7)	6588(3)	579(6)	25.4(17)
C6	5501(8)	6518(3)	-219(6)	37(2)
C7	5114(6)	6322(3)	1498(6)	23.2(16)
C8	4544(6)	6390(3)	2252(6)	22.4(16)
C9	454(6)	6214(2)	2943(5)	18.5(15)
C10	-463(6)	5841(3)	2617(5)	22.6(16)
C11	-1655(6)	6047(3)	2281(6)	27.8(19)
C12	-1863(7)	6335(3)	3164(7)	32.3(19)
C13	-967(7)	6696(3)	3529(7)	29(2)
C14	237(6)	6486(3)	3855(6)	24.1(17)
C15	2094(6)	5735(3)	2080(5)	19.0(16)
C16	1436(7)	5972(3)	1067(6)	26.1(18)
C17	1800(7)	5796(3)	132(6)	33(2)

C18	1659(7)	5283(3)	32(6)	38(2)
C19	2311(7)	5046(3)	1048(6)	29.5(19)
C20	1944(8)	5219(3)	1982(6)	28.5(19)
C21	4991(7)	6754(3)	5535(6)	20.9(16)
C22	5895(6)	6748(3)	4943(6)	24.1(16)
C23	6597(7)	7182(3)	5185(6)	30.5(18)
C24	7173(7)	7247(3)	6368(7)	37(2)
C25	6296(7)	7216(3)	6978(6)	33(2)
C26	5621(7)	6778(3)	6742(6)	28.4(18)
C27	4538(6)	5733(3)	5296(5)	19.8(16)
C28	4839(7)	5546(3)	4325(5)	23.3(17)
C29	5075(7)	5039(3)	4452(6)	25.9(18)
C30	6053(7)	4942(3)	5453(6)	30.9(19)
C31	5804(7)	5140(3)	6430(6)	26.3(18)
C32	5563(7)	5652(3)	6293(6)	24.1(17)
C33	2991(6)	6336(3)	6013(5)	18.5(16)
C34	2653(6)	6831(3)	6164(5)	22.5(16)
C35	1701(7)	6844(3)	6695(6)	25.2(18)
C36	2043(8)	6585(3)	7745(6)	38(2)
C37	2395(7)	6102(3)	7600(6)	32(2)
C38	3354(6)	6076(3)	7071(5)	25.1(17)
C39	1901(6)	4884(3)	5620(6)	22.9(17)
C40	1443(6)	4965(3)	6446(6)	24.1(17)
C41	1449(5)	4639(3)	7217(5)	24.0(15)
C42	1929(7)	4217(3)	7157(6)	27.8(18)
C43	2412(7)	4133(3)	6348(6)	28.3(18)
C44	2385(7)	4465(3)	5585(6)	23.6(17)
C45	1493(8)	3937(3)	8689(7)	40(2)
Au2	8483.7(2)	4623.4(2)	413.8(2)	18.39(7)
S2	7295.7(15)	3152.1(6)	1790.5(12)	18.7(3)
P3	6428.3(16)	3705.0(7)	-254.3(13)	18.1(4)
P4	8465.7(15)	4031.3(6)	1576.7(12)	16.8(4)
O4	8438(4)	3029.1(18)	2399(4)	22.3(12)
O5	6623(4)	2791.4(18)	1163(4)	24.4(11)
O6	8442(5)	6173(2)	-2850(4)	33.3(13)
C46	7344(6)	3622(2)	1047(5)	17.8(14)
C47	6583(6)	3282(3)	2769(5)	20.0(15)
C48	6834(6)	3008(3)	3664(5)	23.2(16)
C49	6256(7)	3067(3)	4411(6)	26.4(18)
C50	5443(6)	3405(3)	4302(6)	26.8(18)
C51	5222(6)	3687(3)	3417(6)	24.3(17)
C52	5782(6)	3620(3)	2652(5)	21.9(16)
C53	4798(7)	3468(3)	5102(6)	33(2)
C54	8273(7)	4241(3)	2843(5)	20.9(16)
C55	8493(7)	4747(3)	3003(6)	30(2)
C56	8141(8)	4909(3)	3963(7)	33(2)
C57	8755(6)	4641(4)	4951(5)	34.7(19)
C58	8581(7)	4136(3)	4796(6)	29.8(19)
C59	8942(6)	3973(3)	3843(6)	25.3(18)
C60	9887(6)	3766(3)	1931(5)	20.2(16)
C61	10081(6)	3502(3)	1002(6)	23.1(16)
C62	11269(7)	3286(3)	1308(7)	31(2)
C63	12214(7)	3639(3)	1712(6)	30.0(18)
C64	12007(7)	3920(3)	2614(6)	28.1(19)
C65	10808(6)	4133(3)	2292(5)	20.7(15)
C66	5298(6)	3273(3)	-665(5)	22.7(17)
C67	4693(6)	3253(3)	-1870(5)	25.2(16)
C68	3978(6)	2821(3)	-2115(6)	31.1(19)
C69	3121(7)	2791(3)	-1500(7)	31.4(19)
C70	3683(6)	2853(3)	-309(6)	26.3(17)

C71	4402(6)	3282(3)	-65(6)	26.1(17)
C72	5830(6)	4283(3)	-406(5)	20.1(16)
C73	5558(6)	4474(2)	570(5)	22.7(17)
C74	5344(7)	4988(3)	457(6)	27.8(19)
C75	4362(7)	5089(3)	-540(6)	29.1(18)
C76	4603(7)	4888(3)	-1504(6)	28.7(19)
C77	4816(6)	4376(3)	-1399(5)	20.5(16)
C78	7341(6)	3657(3)	-1132(5)	20.6(16)
C79	7660(7)	3163(3)	-1263(6)	23.6(17)
C80	8603(7)	3130(3)	-1794(6)	28.5(18)
C81	8280(8)	3382(3)	-2840(6)	33(2)
C82	7962(7)	3874(3)	-2717(6)	31.7(19)
C83	7016(6)	3915(3)	-2184(6)	26.2(17)
C84	8486(6)	5132(3)	-652(5)	18.6(15)
C85	8092(7)	5569(3)	-573(6)	26.6(18)
C86	8085(7)	5909(3)	-1306(6)	27.7(18)
C87	8493(6)	5813(3)	-2163(6)	24.7(17)
C88	8884(5)	5388(3)	-2274(5)	23.0(14)
C89	8868(6)	5049(3)	-1533(6)	23.3(17)
C90	8834(8)	6078(3)	-3748(7)	39(2)
Cl1	10416(2)	7277.3(9)	9127(2)	51.9(6)
Cl2	8584(2)	7117.4(9)	10081(2)	54.6(6)
C91	9923(8)	6935(3)	9993(8)	50(3)
Cl3	1462(2)	3165.0(9)	4919(2)	59.4(7)
Cl4	-417(2)	2724.6(9)	5454.4(19)	48.9(6)
C92	380(7)	2759(3)	4533(6)	41(2)

Table 24. Anisotropic displacement parameters (Å^2×10^3) for **1a**. The anisotropic displacement factor exponent takes the form: $-2\pi^2[h^2a^{*2}U_{11}+2hka^*b^*U_{12}+\ldots]$.

**Atom**	**U_{11}**	**U_{22}**	**U_{33}**	**U_{23}**	**U_{13}**	**U_{12}**
Au1	18.73(14)	22.06(16)	16.21(12)	2.60(11)	4.70(9)	0.25(12)
S1	18.9(9)	20.0(9)	16.2(7)	1.3(6)	2.6(6)	0.1(7)
P1	16.1(9)	19.9(10)	13.6(8)	0.3(6)	3.2(6)	-0.8(7)
P2	16.7(9)	24.5(10)	12.9(8)	0.8(7)	1.7(7)	0.9(7)
O1	30(3)	21(3)	21(2)	-3(2)	4(2)	0(2)
O2	21(3)	27(3)	20(2)	6(2)	3(2)	1(2)
O3	44(4)	35(3)	26(3)	11(2)	10(2)	5(3)
C1	22(4)	21(4)	12(3)	-2(3)	5(3)	1(3)
C2	12(3)	27(4)	16(3)	-3(3)	5(3)	-3(3)
C3	24(4)	30(5)	19(3)	3(3)	-1(3)	-1(3)
C4	29(4)	34(5)	19(3)	4(3)	2(3)	-8(4)
C5	27(4)	32(5)	16(3)	-3(3)	4(3)	-13(3)
C6	42(5)	46(6)	23(4)	-3(4)	11(4)	-5(4)
C7	17(4)	29(4)	23(4)	1(3)	4(3)	-3(3)
C8	22(4)	26(4)	19(3)	-2(3)	5(3)	-1(3)
C9	18(4)	15(4)	22(3)	1(3)	4(3)	5(3)
C10	20(4)	27(4)	19(3)	-6(3)	2(3)	-5(3)
C11	13(4)	39(5)	29(4)	-5(4)	1(3)	2(3)
C12	21(4)	44(6)	33(4)	-10(4)	8(3)	0(4)
C13	23(4)	31(5)	32(4)	-10(4)	6(3)	3(3)
C14	18(4)	32(5)	21(4)	-5(3)	4(3)	6(3)
C15	17(4)	28(5)	15(3)	-2(3)	8(3)	1(3)
C16	27(4)	35(5)	15(3)	0(3)	5(3)	-1(4)
C17	28(4)	56(6)	13(3)	1(3)	3(3)	5(4)

C18	28(4)	61(7)	27(4)	-26(4)	10(3)	-12(4)
C19	33(5)	29(5)	33(4)	-9(3)	19(4)	-6(4)
C20	42(5)	21(4)	27(4)	-10(3)	17(3)	-9(4)
C21	21(4)	22(4)	21(4)	0(3)	7(3)	-1(3)
C22	19(4)	26(4)	23(4)	-1(3)	1(3)	-4(3)
C23	25(4)	31(5)	33(4)	1(3)	4(3)	-7(3)
C24	31(5)	39(5)	34(4)	-5(4)	-1(4)	-17(4)
C25	29(5)	35(5)	26(4)	-5(3)	-4(3)	-4(4)
C26	23(4)	37(5)	22(4)	-2(3)	2(3)	-4(3)
C27	22(4)	22(4)	16(3)	5(3)	7(3)	4(3)
C28	27(4)	28(4)	15(3)	4(3)	6(3)	9(3)
C29	26(4)	29(5)	25(4)	1(3)	10(3)	5(3)
C30	33(5)	33(5)	25(4)	5(3)	6(3)	14(4)
C31	20(4)	39(5)	17(3)	10(3)	2(3)	10(3)
C32	22(4)	31(5)	17(3)	3(3)	2(3)	7(3)
C33	20(4)	20(4)	15(3)	1(3)	6(3)	1(3)
C34	24(4)	26(4)	17(3)	-3(3)	5(3)	1(3)
C35	26(4)	27(5)	22(4)	-1(3)	6(3)	3(3)
C36	42(5)	51(6)	20(4)	1(4)	11(4)	8(4)
C37	36(5)	41(5)	20(4)	6(4)	13(3)	5(4)
C38	29(4)	26(4)	18(3)	3(3)	4(3)	6(3)
C39	19(4)	28(5)	21(4)	5(3)	4(3)	-4(3)
C40	22(4)	30(5)	21(4)	3(3)	8(3)	4(3)
C41	22(3)	33(4)	17(3)	-2(4)	6(2)	-1(4)
C42	26(4)	38(5)	17(4)	8(3)	1(3)	-1(3)
C43	36(5)	27(5)	23(4)	1(3)	10(3)	5(4)
C44	30(4)	21(4)	21(3)	1(3)	9(3)	1(3)
C45	37(5)	51(6)	35(5)	23(4)	16(4)	0(4)
Au2	17.72(14)	21.67(15)	15.88(12)	2.52(11)	5.06(9)	-0.31(12)
S2	18.8(9)	20.7(9)	15.9(7)	1.6(6)	4.2(6)	-1.0(7)
P3	16.4(9)	23.3(10)	13.5(8)	0.3(7)	2.9(7)	-1.3(7)
P4	17.5(9)	20.2(10)	13.0(7)	0.6(6)	4.9(6)	-1.5(7)
O4	21(3)	24(3)	20(2)	5(2)	4(2)	1(2)
O5	26(3)	23(3)	22(2)	-4(2)	4(2)	-6(2)
O6	35(3)	37(4)	30(3)	15(3)	12(2)	2(3)
C46	15(3)	22(4)	16(3)	1(3)	4(3)	-2(3)
C47	22(4)	25(4)	13(3)	0(3)	5(3)	-4(3)
C48	29(4)	20(4)	19(3)	2(3)	3(3)	0(3)
C49	35(5)	26(4)	17(3)	6(3)	6(3)	-6(3)
C50	21(4)	44(5)	17(3)	-4(3)	7(3)	-8(3)
C51	21(4)	30(5)	20(4)	-2(3)	4(3)	-4(3)
C52	20(4)	29(4)	16(3)	6(3)	4(3)	-4(3)
C53	29(5)	53(6)	21(4)	-1(4)	12(3)	-9(4)
C54	22(4)	26(4)	15(3)	-1(3)	6(3)	-3(3)
C55	36(5)	38(6)	26(4)	-4(3)	22(3)	1(4)
C56	41(5)	32(5)	34(4)	-16(4)	21(4)	-11(4)
C57	19(4)	61(6)	25(3)	-16(4)	10(3)	-4(5)
C58	24(4)	49(5)	16(3)	-1(3)	6(3)	-3(4)
C59	18(4)	36(5)	20(4)	3(3)	2(3)	-3(3)
C60	17(4)	28(4)	15(3)	3(3)	4(3)	-4(3)
C61	19(4)	24(4)	24(4)	-6(3)	3(3)	2(3)
C62	28(5)	34(5)	30(4)	-2(4)	6(3)	-1(4)
C63	18(4)	34(5)	36(4)	-3(4)	4(3)	6(3)

C64	19(4)	36(5)	27(4)	-2(3)	3(3)	-6(3)
C65	15(4)	28(4)	21(3)	-4(3)	8(3)	-3(3)
C66	21(4)	30(5)	15(3)	0(3)	1(3)	-6(3)
C67	26(4)	29(4)	17(3)	-2(3)	0(3)	-4(3)
C68	24(4)	39(5)	25(4)	-4(3)	-2(3)	-2(3)
C69	22(4)	29(5)	38(5)	0(4)	1(3)	-4(3)
C70	16(4)	35(5)	29(4)	1(3)	7(3)	-1(3)
C71	21(4)	36(5)	23(4)	3(3)	8(3)	-2(3)
C72	21(4)	25(4)	16(3)	2(3)	7(3)	-2(3)
C73	27(4)	23(4)	19(3)	1(3)	7(3)	4(3)
C74	38(5)	27(5)	20(4)	-1(3)	10(3)	4(4)
C75	28(4)	32(5)	29(4)	6(3)	10(3)	12(3)
C76	25(4)	37(5)	24(4)	7(3)	8(3)	5(4)
C77	14(4)	37(5)	12(3)	3(3)	6(3)	4(3)
C78	22(4)	25(4)	16(3)	-1(3)	6(3)	-3(3)
C79	27(4)	24(4)	20(4)	-2(3)	8(3)	-7(3)
C80	30(4)	26(5)	34(4)	-7(3)	17(3)	1(4)
C81	42(5)	41(5)	22(4)	0(3)	17(4)	1(4)
C82	36(5)	37(5)	26(4)	6(4)	15(3)	4(4)
C83	25(4)	34(5)	21(4)	0(3)	10(3)	6(3)
C84	17(4)	19(4)	20(3)	2(3)	6(3)	-3(3)
C85	29(4)	34(5)	18(3)	-2(3)	10(3)	-3(3)
C86	34(5)	20(4)	28(4)	2(3)	6(3)	1(4)
C87	22(4)	29(5)	20(4)	5(3)	2(3)	-9(3)
C88	24(4)	29(4)	16(3)	3(3)	4(2)	0(4)
C89	22(4)	21(4)	26(4)	4(3)	5(3)	0(3)
C90	36(5)	51(6)	36(5)	19(4)	19(4)	3(4)
Cl1	48.9(14)	48.3(14)	62.1(15)	-6.6(11)	21.8(11)	10.1(11)
Cl2	51.3(15)	48.3(15)	68.2(15)	11.0(12)	23.5(12)	9.2(11)
C91	35(5)	38(5)	67(6)	8(5)	-2(4)	3(4)
Cl3	48.6(14)	54.3(16)	63.0(15)	3.0(12)	-3.1(11)	-10.0(12)
Cl4	46.4(13)	48.7(14)	50.4(13)	-2.4(10)	12.2(10)	8.5(11)
C92	42(5)	35(5)	36(4)	4(4)	-3(4)	4(4)

3.11 Crystal structure of 2a.

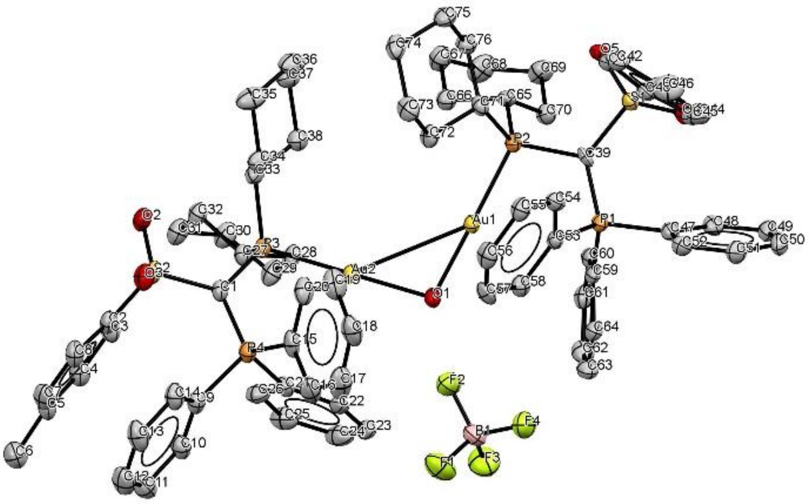

Figure 53. Molecular structure of **2a**, showing thermal displacement ellipsoids at the 50% probability level and atom labeling scheme.

Table 25. Fractional Atomic Coordinates (×10^4) and Equivalent Isotropic Displacement Parameters (Å^2×10^3) for 2a**. Ueq is defined as 1/3 of of the trace of the orthogonalised U_{IJ} tensor.**

Atom	*x*	*y*	*z*	U(eq)
Au1	2227.7(2)	7268.5(2)	1303.5(2)	19.34(6)
Au2	1203.3(2)	7393.4(2)	2648.0(2)	19.94(6)
S1	3815.1(8)	7882.8(8)	-630.6(7)	22.0(2)
S2	1300.1(8)	9294.9(9)	5339.3(7)	25.1(2)
P1	3580.7(8)	6648.6(8)	294.4(7)	19.0(2)
P2	2907.6(8)	8111.1(8)	677.2(7)	20.1(2)
P3	772.0(8)	8340.9(8)	3462.9(7)	21.4(2)
P4	1500.0(9)	7565.9(9)	4606.4(8)	23.4(3)
O1	1541(2)	6486(2)	1854(2)	23.9(7)
O2	1011(3)	9897(3)	5059(2)	30.1(8)
O3	2250(3)	9720(3)	5989(2)	32.6(9)
O4	3502(3)	7127(3)	-1431(2)	27.6(8)
O5	3572(3)	8630(3)	-615(2)	27.2(7)
C1	1185(3)	8409(3)	4483(3)	23.0(10)
C2	455(4)	8778(4)	5756(3)	26.6(11)
C3	-525(4)	8257(4)	5259(3)	28.8(11)
C4	-1176(4)	7771(4)	5552(3)	31.5(12)
C5	-851(4)	7813(4)	6347(4)	33.2(12)
C6	-1519(5)	7272(5)	6667(4)	42.3(14)
C7	130(4)	8375(5)	6845(3)	35.0(13)
C8	788(4)	8862(4)	6557(3)	32.6(12)
C9	1553(4)	7482(4)	5580(3)	27.3(11)
C10	811(4)	6790(4)	5633(3)	31.3(12)
C11	902(5)	6707(5)	6375(4)	39.2(13)
C12	1744(5)	7321(5)	7071(4)	43.3(15)

C13	2490(4)	8021(5)	7031(4)	39.9(14)
C14	2402(4)	8103(4)	6289(3)	31.9(12)
C15	2700(4)	7752(4)	4599(3)	30.8(12)
C16	3101(5)	7209(5)	4801(4)	42.0(15)
C17	4038(5)	7394(6)	4857(4)	47.9(18)
C18	4549(4)	8111(5)	4697(4)	40.7(15)
C19	4186(4)	8663(5)	4491(4)	41.5(15)
C20	3246(4)	8478(4)	4444(4)	34.2(13)
C21	585(4)	6426(4)	3799(3)	27.6(11)
C22	815(5)	5800(4)	3295(3)	32.8(12)
C23	70(6)	4952(4)	2668(4)	42.1(15)
C24	-882(5)	4735(4)	2553(4)	46.0(16)
C25	-1122(4)	5350(4)	3075(4)	38.1(13)
C26	-386(4)	6194(4)	3686(3)	31.4(12)
C27	-565(3)	7887(3)	3067(3)	22.8(10)
C28	-1079(4)	7195(4)	2146(3)	28.7(11)
C29	-2176(4)	6735(4)	1921(3)	31.7(12)
C30	-2570(4)	7444(4)	2135(4)	33.4(12)
C31	-2024(4)	8151(4)	3043(4)	33.6(13)
C32	-928(4)	8634(4)	3263(3)	27.7(11)
C33	1268(4)	9485(3)	3441(3)	26.6(11)
C34	2380(4)	10053(4)	3891(4)	32.4(12)
C35	2712(4)	10998(4)	3850(4)	41.3(15)
C36	2396(4)	10897(4)	2959(4)	38.0(13)
C37	1304(4)	10305(4)	2506(4)	32.8(12)
C38	970(4)	9353(4)	2532(3)	29.5(11)
C39	3362(3)	7462(3)	25(3)	19.4(9)
C40	5104(4)	8395(4)	-207(3)	24.8(10)
C41	5643(4)	9304(4)	420(3)	27.1(10)
C42	6632(4)	9663(4)	817(3)	31.1(12)
C43	7108(4)	9126(4)	603(4)	34.0(12)
C44	6571(4)	8240(4)	-42(4)	36.2(13)
C45	5579(4)	7869(4)	-461(4)	29.1(11)
C46	8178(4)	9499(5)	1085(5)	46.3(16)
C47	4202(3)	6148(3)	-279(3)	23.2(10)
C48	3736(4)	5623(4)	-1133(3)	27.1(11)
C49	4203(4)	5248(4)	-1588(3)	29.9(11)
C50	5126(4)	5370(4)	-1189(4)	32.1(12)
C51	5573(4)	5861(4)	-333(4)	31.0(12)
C52	5114(4)	6253(4)	121(3)	25.9(10)
C53	4350(3)	7188(3)	1386(3)	20.1(9)
C54	5209(3)	7991(4)	1637(3)	23.6(10)
C55	5831(4)	8485(4)	2470(3)	28.3(11)
C56	5600(4)	8164(4)	3039(3)	31.9(12)
C57	4752(4)	7375(4)	2804(3)	30.9(12)
C58	4115(4)	6876(4)	1969(3)	26.4(11)
C59	2474(3)	5652(3)	74(3)	20.9(9)
C60	1586(3)	5510(3)	-433(3)	22.6(10)
C61	732(4)	4745(4)	-612(3)	29.4(11)
C62	760(4)	4120(4)	-285(4)	34.7(13)
C63	1640(4)	4241(4)	209(4)	32.6(12)
C64	2500(4)	4990(3)	374(3)	25.8(10)
C65	1995(3)	8312(3)	54(3)	21.7(9)

C66	1412(4)	8620(4)	556(3)	27.3(11)
C67	733(4)	8879(4)	64(4)	36.3(13)
C68	56(4)	8090(5)	-794(4)	41.9(15)
C69	617(4)	7731(4)	-1276(4)	36.3(13)
C70	1287(4)	7465(4)	-773(3)	27.7(11)
C71	3907(3)	9277(3)	1453(3)	23.4(10)
C72	4008(4)	9431(4)	2345(3)	27.9(11)
C73	4924(4)	10332(4)	2969(3)	32.7(12)
C74	4945(4)	11178(4)	2822(3)	31.4(12)
C75	4798(4)	11012(4)	1925(4)	31.8(12)
C76	3892(4)	10119(4)	1306(3)	27.1(11)
Cl1	1598.9(15)	4938.4(18)	4892.7(17)	76.4(6)
Cl2	3500(2)	5887(2)	6194.9(18)	93.4(8)
C77	2768(7)	5027(7)	5197(7)	79(3)
Cl3	6188.9(14)	6430.3(16)	3783.5(13)	66.5(5)
Cl4	5665.4(13)	6243.3(14)	5177.4(12)	56.6(4)
C78	5311(6)	5759(6)	4086(5)	68(2)
Cl5	2182.4(13)	1634.7(13)	1320.1(13)	55.5(4)
Cl6	4052.5(17)	3112.7(17)	2587.6(16)	77.6(6)
C79	2919(6)	2865(6)	1891(6)	57.1(19)
F1	2995(3)	5046(3)	3412(3)	53.7(10)
F2	2578(3)	5759(3)	2640(2)	42.5(8)
F3	1767(3)	4182(3)	2150(3)	57.0(11)
F4	3324(3)	4886(3)	2239(3)	51.1(10)
B1	2650(5)	4951(5)	2601(4)	33.6(14)

Table 26. Anisotropic displacement parameters ($Å^2×10^3$) for **2a**. The anisotropic displacement factor exponent takes the form: $-2\pi^2[h^2a^{*2}U_{11}+2hka^*b^*U_{12}+…]$.

Atom	U_{11}	U_{22}	U_{33}	U_{23}	U_{13}	U_{12}
Au1	22.10(9)	19.40(10)	15.98(10)	6.88(7)	8.24(7)	8.86(7)
Au2	23.08(9)	19.09(10)	16.62(10)	5.93(8)	8.73(7)	9.29(7)
S1	28.4(5)	21.8(6)	16.6(5)	9.2(4)	10.3(4)	10.2(4)
S2	26.1(5)	23.3(6)	16.4(5)	0.8(4)	4.9(4)	10.0(4)
P1	21.2(5)	16.9(5)	15.4(5)	5.5(4)	6.4(4)	6.5(4)
P2	23.2(5)	19.5(6)	15.7(5)	6.3(4)	7.1(4)	9.1(4)
P3	23.3(5)	19.6(6)	17.1(5)	3.7(5)	7.5(4)	9.3(4)
P4	26.1(5)	25.7(6)	17.1(6)	6.8(5)	7.9(4)	12.6(5)
O1	28.0(16)	20.4(17)	21.3(17)	6.3(14)	13.5(14)	8.6(14)
O2	37.4(19)	29.1(19)	23.3(18)	6.0(15)	11.2(15)	19.5(16)
O3	29.3(17)	31(2)	20.9(18)	1.5(15)	2.6(14)	9.6(15)
O4	38.4(18)	28.8(19)	14.3(16)	8.2(15)	12.7(14)	13.4(16)
O5	36.9(18)	25.8(18)	27.9(19)	18.1(16)	17.2(15)	15.1(15)
C1	24.3(17)	23(2)	18.3(18)	4.2(15)	9.7(14)	10.3(15)
C2	31(2)	30(3)	21(2)	7(2)	11.3(19)	18(2)
C3	28(2)	32(3)	21(2)	8(2)	7.8(19)	12(2)
C4	29(2)	36(3)	27(3)	9(2)	10(2)	16(2)
C5	43(3)	36(3)	26(3)	12(2)	20(2)	21(3)
C6	49(3)	45(4)	38(3)	18(3)	22(3)	22(3)
C7	44(3)	48(3)	21(3)	16(2)	11(2)	29(3)
C8	33(3)	42(3)	23(3)	8(2)	11(2)	23(2)
C9	31(2)	32(3)	20(2)	8(2)	9.7(19)	18(2)
C10	35(3)	37(3)	22(3)	11(2)	11(2)	18(2)
C11	43(3)	48(4)	37(3)	24(3)	20(3)	23(3)
C12	55(4)	61(4)	26(3)	21(3)	15(3)	37(3)

C13	36(3)	58(4)	22(3)	12(3)	8(2)	23(3)
C14	30(2)	39(3)	22(3)	7(2)	7(2)	17(2)
C15	32(2)	43(3)	15(2)	7(2)	9.4(19)	20(2)
C16	52(3)	60(4)	36(3)	28(3)	26(3)	38(3)
C17	54(4)	74(5)	33(3)	21(3)	16(3)	48(4)
C18	39(3)	62(4)	21(3)	15(3)	10(2)	27(3)
C19	31(3)	50(4)	33(3)	10(3)	11(2)	14(3)
C20	28(2)	34(3)	28(3)	3(2)	10(2)	10(2)
C21	38(3)	27(3)	17(2)	10(2)	11(2)	14(2)
C22	59(3)	25(3)	25(3)	16(2)	22(2)	22(3)
C23	80(5)	21(3)	24(3)	9(2)	24(3)	20(3)
C24	66(4)	25(3)	27(3)	10(2)	5(3)	9(3)
C25	40(3)	30(3)	27(3)	11(2)	-2(2)	7(2)
C26	38(3)	20(3)	28(3)	9(2)	6(2)	8(2)
C27	23(2)	23(2)	18(2)	4.6(19)	6.9(17)	9.1(18)
C28	30(2)	20(2)	23(3)	0(2)	6(2)	8(2)
C29	28(2)	27(3)	24(3)	0(2)	0.4(19)	10(2)
C30	28(2)	29(3)	29(3)	4(2)	1(2)	12(2)
C31	32(3)	31(3)	30(3)	5(2)	8(2)	17(2)
C32	28(2)	24(3)	22(2)	3(2)	5.0(19)	11(2)
C33	29(2)	20(2)	27(3)	6(2)	11(2)	10(2)
C34	29(2)	29(3)	40(3)	17(2)	13(2)	13(2)
C35	29(3)	28(3)	55(4)	16(3)	6(3)	8(2)
C36	34(3)	29(3)	53(4)	21(3)	20(3)	12(2)
C37	39(3)	26(3)	36(3)	16(2)	14(2)	16(2)
C38	35(3)	27(3)	28(3)	12(2)	14(2)	14(2)
C39	19.6(16)	27(2)	12.8(17)	10.3(15)	9.9(13)	8.6(15)
C40	29(2)	26(3)	21(2)	13(2)	12.5(19)	10(2)
C41	39(3)	21(2)	25(3)	13(2)	16(2)	12(2)
C42	35(3)	25(3)	27(3)	12(2)	12(2)	5(2)
C43	33(3)	34(3)	38(3)	23(3)	14(2)	11(2)
C44	35(3)	29(3)	47(4)	19(3)	19(3)	14(2)
C45	34(3)	22(3)	34(3)	14(2)	17(2)	13(2)
C46	32(3)	38(3)	51(4)	16(3)	7(3)	5(3)
C47	26(2)	20(2)	21(2)	7.7(19)	10.1(18)	7.4(18)
C48	27(2)	23(2)	24(3)	8(2)	9.3(19)	5.5(19)
C49	37(3)	22(3)	27(3)	7(2)	16(2)	11(2)
C50	39(3)	29(3)	36(3)	12(2)	26(2)	17(2)
C51	30(2)	33(3)	36(3)	16(2)	17(2)	18(2)
C52	27(2)	26(3)	26(3)	13(2)	11.1(19)	12(2)
C53	24(2)	18(2)	15(2)	4.7(18)	4.9(17)	9.1(18)
C54	25(2)	25(2)	25(2)	13(2)	10.7(19)	11.6(19)
C55	26(2)	27(3)	22(3)	4(2)	4.0(19)	11(2)
C56	32(2)	36(3)	16(2)	3(2)	0.2(19)	15(2)
C57	40(3)	39(3)	16(2)	15(2)	11(2)	18(2)
C58	27(2)	28(3)	23(3)	10(2)	9.3(19)	13(2)
C59	26(2)	17(2)	16(2)	3.2(18)	8.8(17)	8.0(18)
C60	29(2)	19(2)	14(2)	3.1(18)	8.3(18)	7.6(19)
C61	26(2)	26(3)	24(3)	3(2)	5.0(19)	8(2)
C62	33(3)	26(3)	31(3)	7(2)	14(2)	2(2)
C63	43(3)	22(3)	30(3)	12(2)	18(2)	9(2)
C64	33(2)	21(2)	23(2)	7(2)	12.3(19)	12(2)
C65	30(2)	20(2)	18(2)	11.2(19)	7.8(18)	12.0(19)
C66	29(2)	27(3)	24(2)	10(2)	9.8(19)	12(2)
C67	39(3)	37(3)	33(3)	12(3)	11(2)	23(3)
C68	35(3)	42(3)	41(3)	11(3)	2(2)	22(3)
C69	42(3)	34(3)	26(3)	10(2)	1(2)	21(2)
C70	32(2)	25(3)	20(2)	8(2)	5.6(19)	12(2)
C71	25(2)	23(2)	16(2)	5.3(19)	7.5(17)	7.7(19)
C72	32(2)	24(3)	16(2)	2(2)	4.2(19)	9(2)

C73	31(2)	28(3)	21(3)	4(2)	3(2)	4(2)
C74	29(2)	26(3)	28(3)	3(2)	12(2)	6(2)
C75	34(3)	21(3)	32(3)	9(2)	11(2)	7(2)
C76	31(2)	22(3)	22(2)	9(2)	9(2)	7(2)
Cl1	55.2(10)	74.2(14)	93.0(17)	36.7(13)	24.9(11)	23.3(10)
Cl2	87.1(16)	77.5(16)	78.1(16)	17.4(13)	1.7(13)	30.0(13)
C77	61(5)	67(6)	94(7)	25(5)	14(5)	33(4)
Cl3	57.2(10)	74.8(13)	51.9(11)	19.5(10)	11.5(8)	25.9(9)
Cl4	52.6(9)	56.8(10)	58.0(10)	22.1(8)	14.0(8)	28.7(8)
C78	47(4)	60(5)	62(5)	4(4)	11(3)	13(4)
Cl5	56.1(9)	52.1(10)	61.1(11)	25.7(8)	30.1(8)	21.5(8)
Cl6	73.6(13)	62.0(13)	72.5(14)	16.5(11)	10.7(11)	26.1(10)
C79	67(5)	52(4)	67(5)	32(4)	34(4)	32(4)
F1	54(2)	69(3)	46(2)	37(2)	17.5(18)	29(2)
F2	49.5(19)	44(2)	55(2)	28.9(18)	29.1(17)	30.2(17)
F3	48(2)	44(2)	57(3)	16(2)	16.4(19)	5.8(18)
F4	65(2)	54(2)	67(3)	35(2)	47(2)	40(2)
B1	35(3)	38(4)	34(3)	21(3)	18(3)	16(3)

3.12 Crystal structure of [Au(Cy-^{Cy}JohnPhos)Cl] (P6)

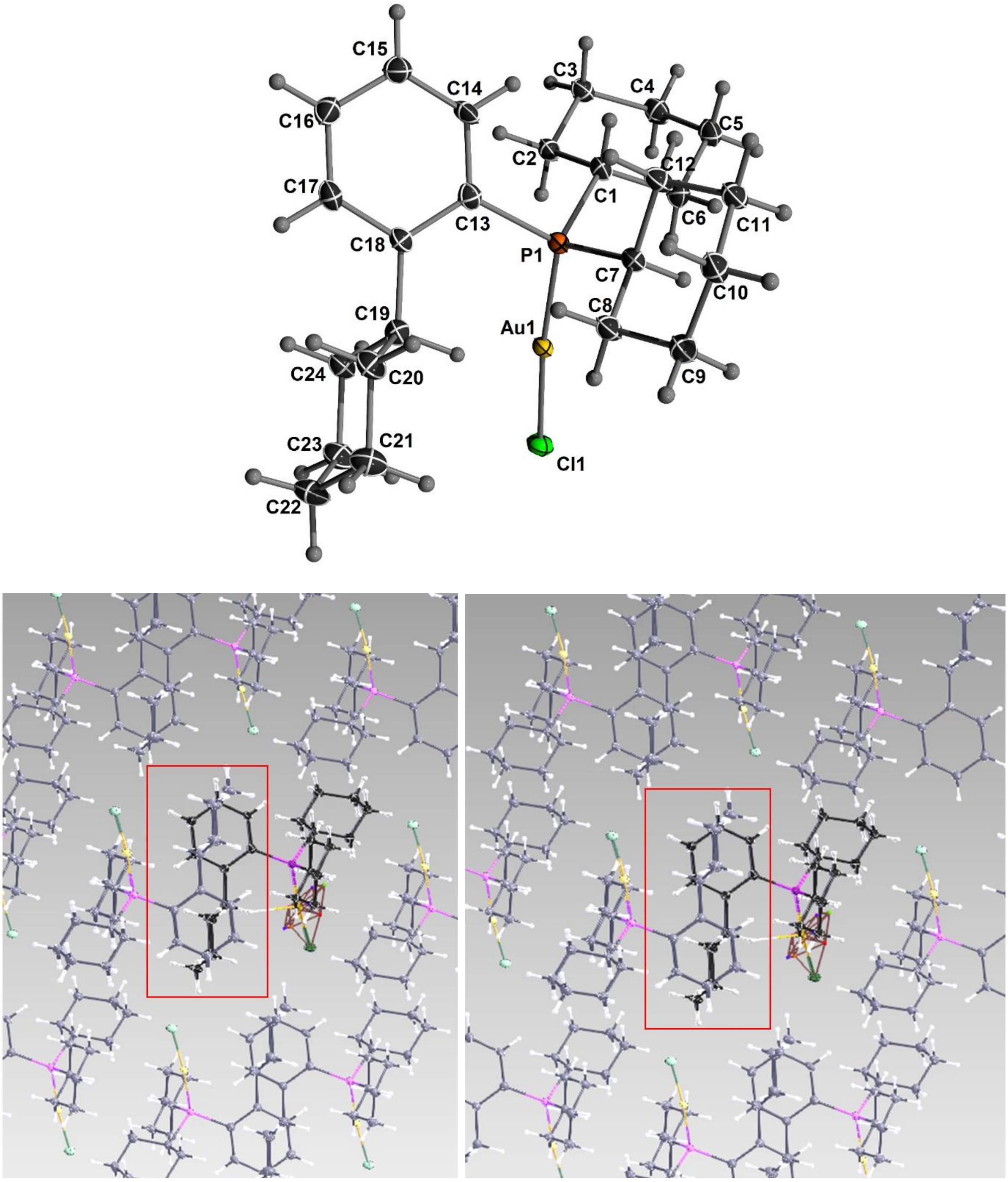

Figure 54. (Top) ORTEP plot of **P6**; ellipsoids at 50% probability. (Bottom) Representation of the packing of **P6** in the crystal. Red boxes highlight the interaction between the phenylene and cyclohexyl group as explanation for the deviation of the calculated structure and the structure determined by XRD analysis. Selected bond lengths [Å] and angles [°]: Au(1)-P(1) 2.2439(8), Au(1)-Cl(1) 2.2977(8), Au(1)-H(19) 2.77(4), C(1)-C(2) 1.528(4), C(1)-C(6) 1.539(4), C(1)-P(1) 1.843(3), P(1)-C(13) 1.834(3), P(1)-C(7) 1.846(3), P(1)-Au(1)-Cl(1) 174.01(3), P(1)-Au(1)-H(19) 65.3(8), Cl(1)-Au(1)-H(19) 119.8(8), C(13)-P(1)-C(1) 105.19(15), C(13)-P(1)-C(7) 108.05(14), C(1)-P(1)-C(7) 104.95(14), C(13)-P(1)-Au(1) 118.45(11), C(1)-P(1)-Au(1) 107.51(11), C(7)-P(1)-Au(1) 111.67(10).

Table 27. Atomic Coordinates (×10[4]) and Equivalent Isotropic Displacement Parameters (Å^2×10^3) for **P6**. U_{eq} is defined as 1/3 of of the trace of the orthogonalised U_{ij} tensor.

Atom	*x*	*y*	*z*	U(eq)
Au(1)	6055(1)	4981(1)	2314(1)	16(1)
Cl(1)	7968(1)	3886(1)	2274(1)	22(1)
C(1)	2512(4)	4599(3)	1103(3)	18(1)
P(1)	4114(1)	5967(1)	2159(1)	15(1)
C(2)	2110(4)	3263(4)	1449(3)	19(1)
C(3)	730(4)	2187(4)	589(3)	20(1)
C(5)	1396(4)	3096(4)	-905(3)	23(1)
C(4)	914(4)	1765(4)	-570(3)	24(1)
C(6)	2770(4)	4160(4)	-42(3)	23(1)
C(8)	5699(4)	8744(4)	2357(3)	21(1)
C(7)	4380(3)	7487(3)	1599(3)	17(1)
C(13)	3496(4)	6554(3)	3387(3)	17(1)
C(12)	2978(4)	8039(4)	1311(3)	21(1)
C(11)	3227(4)	9181(4)	735(3)	22(1)
C(10)	4571(4)	10429(4)	1449(3)	22(1)
C(9)	5953(4)	9889(4)	1782(3)	23(1)
C(14)	1973(4)	6306(4)	3225(3)	20(1)
C(15)	1394(4)	6579(4)	4103(3)	23(1)
C(16)	2334(4)	7131(4)	5183(3)	22(1)
C(17)	3844(4)	7423(4)	5352(3)	21(1)
C(18)	4468(3)	7176(3)	4481(3)	16(1)
C(19)	6152(4)	7585(4)	4777(3)	19(1)
C(20)	6806(4)	9186(4)	5469(3)	22(1)
C(21)	8490(4)	9593(4)	5751(3)	28(1)
C(22)	9180(4)	8630(4)	6356(3)	29(1)
C(23)	8529(4)	7038(4)	5681(3)	26(1)
C(24)	6851(4)	6624(4)	5389(3)	20(1)

Table 28. Anisotropic displacement parameters (Å^2×10^3) for **P6.** The anisotropic displacement factor exponent takes the form: $-2\pi^2[h^2 a^{*2}U^{11} + ... + 2 h k a^* b^* U^{12}]$

Atom	U^{11}	U^{22}	U^{33}	U^{23}	U^{13}	U^{12}
Au(1)	14(1)	20(1)	13(1)	5(1)	2(1)	5(1)
Cl(1)	17(1)	27(1)	23(1)	8(1)	4(1)	10(1)
C(1)	16(2)	21(2)	13(1)	5(1)	2(1)	3(1)
P(1)	13(1)	19(1)	13(1)	5(1)	2(1)	5(1)
C(2)	17(2)	23(2)	15(2)	6(1)	1(1)	4(1)
C(3)	17(2)	21(2)	19(2)	7(1)	2(1)	3(1)
C(5)	23(2)	26(2)	15(2)	5(1)	3(1)	6(1)
C(4)	23(2)	23(2)	19(2)	2(1)	2(1)	4(1)
C(6)	23(2)	28(2)	16(2)	6(1)	6(1)	5(1)
C(8)	17(2)	24(2)	20(2)	9(1)	0(1)	4(1)
C(7)	14(1)	22(2)	15(1)	7(1)	2(1)	4(1)
C(13)	19(2)	16(1)	16(2)	5(1)	4(1)	3(1)
C(12)	17(2)	23(2)	22(2)	9(1)	1(1)	6(1)
C(11)	18(2)	25(2)	20(2)	10(1)	1(1)	5(1)

C(10)	23(2)	23(2)	19(2)	8(1)	3(1)	5(1)
C(9)	21(2)	26(2)	23(2)	10(1)	3(1)	5(1)
C(14)	15(2)	24(2)	14(2)	1(1)	-2(1)	6(1)
C(15)	19(2)	27(2)	21(2)	4(1)	6(1)	6(1)
C(16)	24(2)	24(2)	20(2)	7(1)	10(1)	7(1)
C(17)	22(2)	23(2)	14(2)	4(1)	2(1)	4(1)
C(18)	16(2)	16(1)	15(1)	6(1)	3(1)	5(1)
C(19)	18(2)	22(2)	17(2)	7(1)	4(1)	4(1)
C(20)	21(2)	21(2)	22(2)	7(1)	3(1)	7(1)
C(21)	20(2)	25(2)	33(2)	7(2)	3(2)	2(1)
C(22)	16(2)	32(2)	30(2)	6(2)	-1(1)	4(1)
C(23)	21(2)	29(2)	28(2)	11(2)	4(1)	11(1)
C(24)	18(2)	21(2)	19(2)	6(1)	1(1)	3(1)

4. Computational Studies

4.1 General remarks

All computational studies were carried out without symmetry restrictions. If it was not possible to obtain starting coordinates from crystal structures GaussView 6.0[6] was used. Calculations were performed with the Gaussian16 Revision B.01[7] or the Gaussian16 Revision C.01[8] program packages using Density-Functional Theory (DFT).[9] Energy optimizations were carried out with the PW6B95D3 functional[10] and def2svp basis set[11] as well as the MWB60 ECP[12] as implemented in Gaussian for Au together with GRIMMES D3 dispersion correction with Becke-Johnson damping.[13] To determine the nature of the structure harmonic vibrational frequency analyses were performed on the same level of theory.[14] No imaginary frequencies were observed for the optimized structures. Single point energies were calculated on PW6B95D3[10]/def2tzvp[11] level of theory with the MWB60 ECP[12] as implemented in Gaussian for Au. Solvent corrections were included by using the polarizable continuum model (PCM)[14] for aniline, which is also the solvent used in experiments. In order to probe the explicit coordination of aniline to the complex, we also attempted the optimization of the YPhos gold complexes in which coordination of aniline displaces the C-H-Au interaction. However, these energy optimizations always resulted in the displacement of aniline. This is in line with gold preferentially acting as donor (H bonding) rather than as acceptor (aniline coordination).
NBO Analysis was performed with NBO Version 7.0.[15] The optimized structures were used for the quantum theory of atoms in molecule (QTAIM)[16] and noncovalent interaction (NCI)[17] analyses using Multiwfn[18] to depict the topological properties of the complexes. The results were visualized using the VMD 1.9.4a51 software.[19]

4.2 Pictures of the conformers of the gold complexes

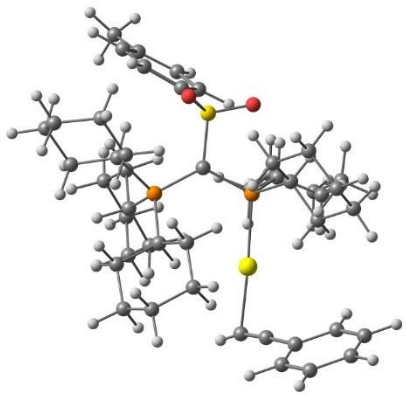

L1-Au(pa)$^{+}$

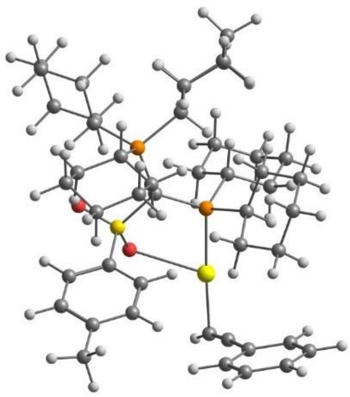

L1-Au(pa)$^{+}$ C2

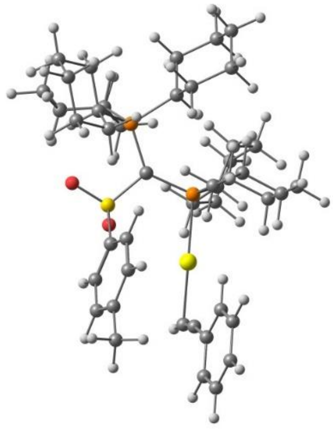

L1-Au(pa)$^{+}$ C3

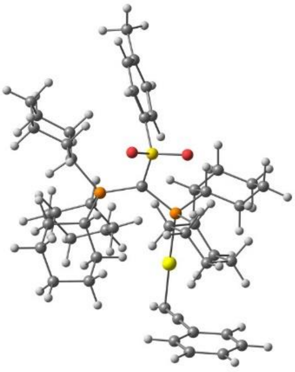

L1-Au(pa)$^+$ C4

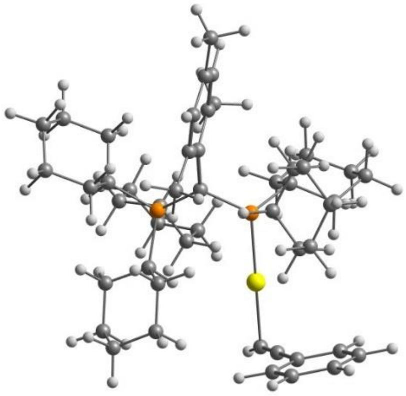

L3-Au(pa)$^+$

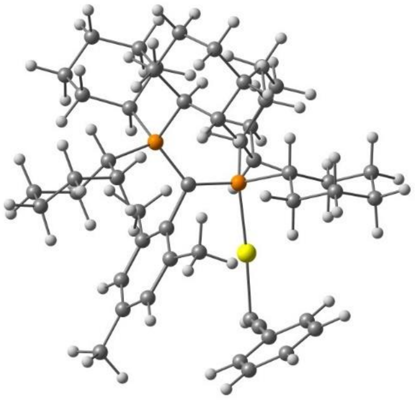

L3-Au(pa)$^+$ C2

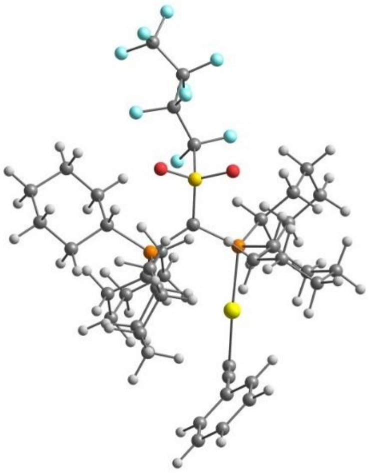

L5-Au(pa)$^+$
C3

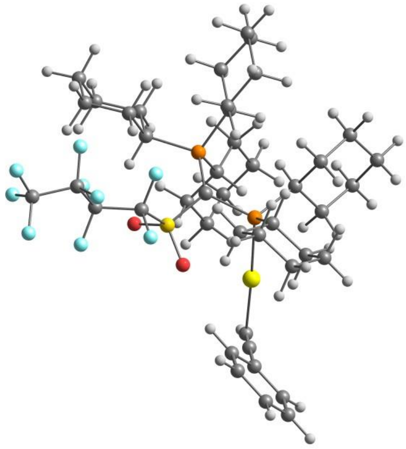

L5-Au(pa)$^+$ C2

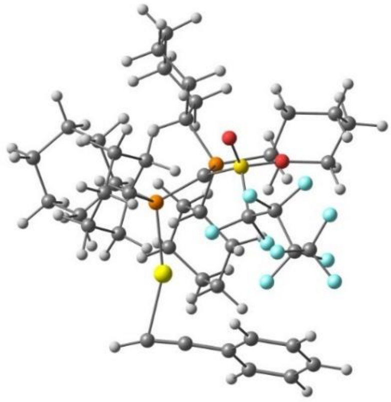

L5-Au(pa)$^+$

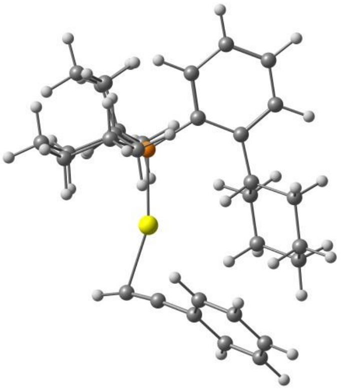

L6-Au(pa)$^+$

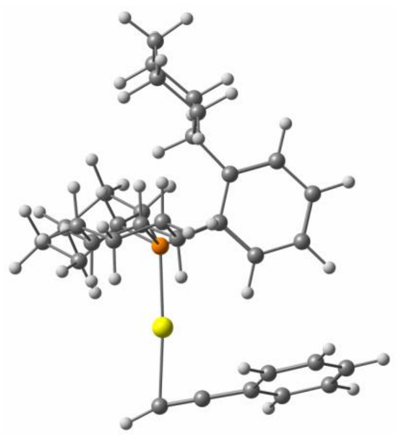

L6-Au(pa)$^+$ C2

4.3 Results of the AIM and NBO analyses

Table 29: Results of AIM analyses for the LAuCl and LAu+ cations without solvent effects: BCP's and local electron density ρ(r) and laplacian $\nabla^2\rho(r)$,

LAuCl complex						LAu+ complex		
	**BCP**	**Posi-tion**	**C-H-AuCl [Å] calc.**	**ρ(r) [e.bohr^{-3}]**	**$\nabla^2\rho(r)$ [e.bohr^{-5}]**	**C_{PCy}-H-Au+ [Å] calc.**	**ρ(r) [e.bohr^{-3}]**	**$\nabla^2\rho(r)$ [e.bohr^{-5}]**
$^{Cy}Y_S$-PCy_2 (**P1**)	1 (H9)	PCy_3 Pos. 1	2.406	0.0233	0.0646	2.473	0.0211	0.0575
	2 (H22A)	PCy_3 Pos. 2	2.842	0.0113	0.0319	2.911	0.0101	0.0275
	3 (H18B)	PCy_3 Pos. 2	3.092	0.0067	0.0204	2.942	0.0107	0.0307
	4 (H11A)	PCy_3 Pos. 3	2.959	0.0053	0.0297	3.062	0.0071	0.0205
	5 (H38A)	PCy_2 Pos. 2	n.o.	n.o.	n.o.	2.899	0.0109	0.0344
$^{Ph}Y_S$-PCy_2 (**A**)	1 (C9)	PPh_3 Pos. 1	3.152	0.0154	0.0471	n.o.	n.o.	n.o.
	2 (CH16)	PPh_3 Pos. 2	3.286 2.900	0.0109	0.0385	3.029	0.0206	0.0646
	3 (H28B)	PCy_2 Pos. 2	2.837	0.0122	0.0384	2.760	0.0113	0.0344
$^{Cy}Y_{oTol}$ -PCy_2 (**P2**)	1 (H21)	PCy_3 Pos. 1	2.529	0.0186	0.052	2.586	0.0170	0.04246
	2 (H20B)	PCy_3 Pos. 2	2.855	0.0119	0.0367	2.996	0.0967	0.0285
	3 (H20A)	PCy_3 Pos. 2	2.877	0.0110	0.0382	2.936	0.0099	0.03
	4 (H26B)	PCy_3 Pos. 2	3.028	0.0088	0.0257	n.o.	n.o.	n.o.
	5 (H34B)	PCy_2 Pos. 2	n.o.	n.o.	n.o.	2.866	0.0111	0.0314
	6 (H38A)	PCy_2 Pos. 2	n.o.	n.o.	n.o.	2.933	0.0101e	0.0292
	7 (H25A)	PCy_3 Pos. 3	n.o.	n.o.	n.o.	3.298	0.0051e	0.0136
$^{Cy}Y_{Mes}$ -PCy_2 (**P3**)	1 (H18B)	PCy_3 Pos. 2	2.562	0.0174	0.0431	2.809	0.0116	0.0276
	2 (H23)	PCy_3 Pos. 1	2.594	0.0167	0.0472	2.669	0.0149	0.0418
	3 (H28A)	PCy_3 Pos. 2	2.868	0.0112	0.0325	2.805	0.0121	0.0361
	4 (H40B)	PCy_2 Pos. 2	2.858	0.0115	0.0359	2.829	0.0121	0.0373
	5 (H30B)	PCy_2 Pos. 2	n.o.	n.o.	n.o.	2.909	0.0106	0.0327
	6 (H27B)	PCy_3 Pos. 2	n.o.	n.o.	n.o.	3.094	0.0099	0.0195
$^{Cy}Y_{SF}$-PCy_2 (**P5**)	1 (H6)	PCy_3 Pos. 1	2.373	0.0248	0.0677	2.439	0.0224	0.0600
	2 (H12)	PCy_3 Pos. 1	2.599	0.0175	0.0456	2.750	0.0144	0.0368
	3 (H31B)	PCy_2 Pos. 2	2.815	0.0123	0.0379	2.713	0.0139	0.0425
	4 (H25A)	PCy_2 Pos. 2	n.o.	n.o.	n.o.	2.813	0.0132	0.0448

Table 30: Results of NBO analysis for the LAuCl and LAu^+ cations without solvent effects: Occupancy of the C-H antibonding σ orbital.

**LAuCl complex**				**LAu^+ complex**		
Complex	**BCP**	**C_{PCy}-H [Å] calc.**	**occ(σ*)**	Complex	**C_{PCy}-H [Å] calc.**	**occ(σ*)**
$^{Cy}Y_S$-PCy_2 (**P1**)	1 (H9)	1.101	0.04185	$^{Cy}Y_S$-PCy_2 Au^+	2.473	0.0211
	2 (H22A)	1.097	0.02186		2.911	0.0101
	3 (H18B)	1.101	0.0223		2.942	0.0107
	4 (H11A)	1.094	0.01898		3.062	0.0071
	5 (H38A)	n.o.	n.o.		2.899	0.0109
$^{Cy}Y_{oTol}$-PCy_2 (**P2**)	1 (H21)	1.1	0.03656	$^{Cy}Y_{oTol}$-PCy_2 Au^+	1.103	0.02145
	2 (H20B)	1.101	0.02144		1.100	0.01059
	3 (H20A)	1.1	0.01591		1.096	0.00917
	4 (H26B)	1.094	0.01841		n.o.	n.o.
	5 (H34B)	n.o.	n.o.		1.102	0.0117
	6 (H38A)	n.o.	n.o.		1.102	0.01097
	7 (H25A)	n.o.	n.o.		1.101	0.01139
$^{Cy}Y_{Mes}$-PCy_2 (**P3**)	1 (H18B)	1.096	0.02939	$^{Cy}Y_{Mes}$-PCy_2 Au^+	1.098	0.02503
	2 (H23)	1.1	0.03269		1.102	0.03136
	3 (H28A)	1.097	0.01845		1.097	0.01834
	4 (H40B)	1.098	0.0154		1.099	0.01528
	5 (H30B)	n.o.	n.o.		1.103	0.02236
	6 (H27B)	n.o.	n.o.		1.105	0.01999
$^{Cy}Y_{SF}$-PCy_2 (**P5**)	1 (H6)	1.101	0.04411	$^{Cy}Y_{SF}$-PCy_2 Au^+	1.106	0.0383
	2 (H12)	1.101	0.03558		1.103	0.0331
	3 (H31B)	1.101	0.02156		1.102	0.02152
	4 (H25A)	n.o.	n.o.		1.100	0.01535

Table 31: results of AIM and NBO analysis of the structures optimized with the PCM model (aniline as solvent): BCP's and local electrondensity ρ(r) and laplacian $\nabla^2\rho(r)$, natural charges on Au and H and occupation of the σ*(C-H) orbital. The first entries correspond to the interactions between Au and the PCy_3 CH moiety and the following to other PCy_3/PCy_2 CH_2 moieties.

	**BCP**	**CH-Au [Å] calc.**	**C-H [Å] calc.**	**ρ(r) [E-1 e.bohr^{-3}]**	**$\nabla^2\rho(r)$[E-1 e.bohr^{-5}]**	**occ(σ*)**	**Nat. Charge Au**	**Nat. Charge H**
$^{Cy}Y_S$-PCy_2-AuCl (**P1**)	H9	2.43813	1.10039	0.21925	0.60347	0.03795	0.07534	0.26871
	H10A	2.99441	1.09414	0.10001	0.27869	0.01809	0.07534	0.23047
	H11B	3.10412	1.1009	0.06521	0.19427	0.02050	0.07534	0.22415

	H22A	2.89443	1.09762	0.10330	0.28557	0.02137	0.07534	0.22846
$^{Cy}Y_{S}$-PCy_2-Au^+pa	H9	2.47456	1.10073	0.20449	0.56283	0.03572	0.27889	0.26067
	H11B	3.07839	1.10087	0.06853	0.20144	0.01938	0.27889	0.20749
	H22A	2.89883	1.0988	0.09925	0.27657	0.02177	0.27889	0.22256
$^{Cy}Y_{S}$-PCy_2-Au^+an	H9	2.483	1.1023	0.20162	0.55154	0.03668	0.23118	0.26172
	H10A	3.04879	1.09374	0.09415	0.25928	0.01789	0.23118	0.22932
	H11B	3.01278	1.10154	0.07608	0.21925	0.01988	0.23118	0.20786
	H22A	2.94246	1.09927	0.09377	0.25463	0.02192	0.23118	0.22583
$^{Cy}Y_{oTol}$-PCy_2-AuCl (**P2**)	H21	2.57429	1.10057	0.17090	0.47469	0.03292	0.06565	0.26427
	H26B	3.06196	1.09367	0.08353	0.24182	0.01792	0.06565	0.24141
	H20A	2.92609	1.09568	0.10166	0.35023	0.01475	0.06565	0.23741
	H20B	2.89135	1.10037	0.11178	0.33658	0.02103	0.06565	0.21936
	H34B	2.87608	1.10200	0.11212	0.34546	0.02097	0.06565	0.22921
	H32B	2.88396	1.09859	0.11027	0.34009	0.01578	0.06565	0.23378
$^{Cy}Y_{oTol}$-PCy_2-Au^+pa	H21	2.54485	1.10165	0.17858	0.48525	0.03387	0.27456	0.25518
	H25A	3.35388	1.10039	0.04256	0.13198	0.01871	0.27456	0.20885
	H20B	2.84157	1.10135	0.11671	0.34804	0.02181	0.27456	0.21516
	H32B	2.80777	1.09941	0.12136	0.37020	0.01600	0.27456	0.22591
$^{Cy}Y_{Mes}$-PCy_2-AuCl (**P3**)	H23	2.64229	1.10051	0.15286	0.43247	0.02994	0.06956	0.26913
	H28A	2.89317	1.09669	0.10744	0.30870	0.01750	0.06956	0.23908
	H27B	3.20599	1.10089	0.05545	0.16857	0.02087	0.06956	0.22466
	H18B	2.59572	1.09682	0.16411	0.40543	0.02761	0.06956	0.23133
	H40B	2.83557	1.09774	0.11922	0.36873	0.01496	0.06956	0.23506
$^{Cy}Y_{Mes}$-PCy_2-Au^+pa	H23	2.67951	1.10075	0.14218	0.40697	0.02872	0.27149	0.26088
	H28A	2.91042	1.09704	0.10226	0.29657	0.01709	0.27149	0.23725
	H27B	3.26624	1.10161	0.05033	0.15416	0.01886	0.27149	0.21116
	H18B	2.6038	1.09831	0.15885	0.39994	0.02679	0.27149	0.22165
	H40B	2.83757	1.0977	0.11759	0.37258	0.01511	0.27149	0.22971
$^{Cy}Y_{Mes}$-PCy_2-Au^+dpa	H23	2.6607	1.10095	0.14685	0.41782	0.02894	0.25593	0.2625
	H28A	2.9404	1.09697	0.09839	0.28295	0.01688	0.25593	0.23503
	H27B	3.25256	1.09987	0.05165	0.15575	0.0188	0.25593	0.2096
	H18B	2.58485	1.0966	0.16617	0.42216	0.02739	0.25593	0.22345
	H30B	2.904	1.10333	0.10574	0.32510	0.0213	0.25593	0.21928
	H40B	2.8519	1.09779	0.11539	0.36673	0.01509	0.25593	0.22892
$^{Cy}Y_{Mes}$-PCy_2-Au^+an	H23	2.80732	1.1025	0.11539	0.33890	0.02711	0.21832	0.26129
	H28A	2.86284	1.09657	0.10997	0.31194	0.01807	0.21832	0.23695
	H18B	2.58022	1.09516	0.17058	0.43203	0.02766	0.21832	0.22666

	H40B	2.83893	1.09827	0.11847	0.36860	0.01509	0.21832	0.22926
$^{Cy}Y_{SF}$-PCy_2-AuCl (**P5**)	H6	2.65485	1.10113	0.15842	0.41276	0.03262	0.09625	0.27206
	H12	2.39394	1.10096	0.23774	0.64578	0.03969	0.09625	0.26566
	H25B	2.79333	1.10118	0.12654	0.38961	0.02069	0.09625	0.22750
$^{Cy}Y_{SF}$-PCy_2-Au^+pa	H12	2.38659	1.10146	0.24048	0.65818	0.03723	0.30621	0.25548
	H25B	2.82817	1.09929	0.13041	0.39416	0.01470	0.30621	0.22854
	H6	2.78595	1.10182	0.12551	0.32879	0.02981	0.30621	0.26644
	H31A	2.76766	1.10113	0.12270	0.41761	0.02196	0.30621	0.21460
$^{Cy}Y_{SF}$-PCy_2-Au^+an	H6	2.45051	1.103	0.21494	0.58184	0.03679	0.24631	0.25743
	H12	2.61589	1.10151	0.16657	0.43479	0.03358	0.24631	0.26578
	H31A	2.7427	1.10203	0.13705	0.42007	0.02144	0.24631	0.21951
^{Cy}JohnPhos-AuCl **(L6)**	H2A	3.16398	1.09641	0.07422	0.23737	0.01481	0.12239	0.22402
	H19	2.48237	1.10017	0.20435	0.54488	0.03521	0.12239	0.23718
	H8B	3.00008	1.10059	0.07523	0.21969	0.02176	0.12239	0.22003
^{Cy}JohnPhos-Au^+pa	H2A	3.15097	1.09686	0.07329	0.23486	0.01462	0.35621	0.22384
	H19	2.52254	1.10246	0.18747	0.50699	0.03365	0.35621	0.22700
	H8B	2.99089	1.10126	0.07735	0.22569	0.01964	0.35621	0.20356
^{Cy}JohnPhos-Au^+dpa	H2A	3.15181	1.09706	0.07432	0.24432	0.01463	0.34949	0.22331
	H19	2.48719	1.1023	0.20072	0.54018	0.03473	0.34949	0.22658
	H8B	2.94393	1.10163	0.08434	0.24465	0.01988	0.34949	0.20466
$^{Ph}Y_S$-PCy_2-AuCl (**A**)	C9	3.2066	–	0.14167	0.41657	–	0.10783	-0.44183
	C16	3.30796	–	0.10212	0.32256	–	0.10783	-0.29472
	H28B	2.8436	1.09855	0.12078	0.38592	0.01492	0.10783	0.23474
$^{Ph}Y_S$-PCy_2-Au^+pa	C9	3.25181	–	0.13298	0.38878	–	0.31307	-0.38800
	H34B	2.83164	1.10225	0.11766	0.35908	0.02102	0.31307	0.22162
$^{Ph}Y_S$-PCy_2-Au^+dpa	H28B	2.3095	1.10179	0.11892	0.35681	0.02173	0.29832	0.21643
$^{Ph}Y_S$-PCy_2-Au^+an	C9	3.2944	–	0.12062	0.33738	–	0.25181	-0.38662
	H34B	2.80094	1.10241	0.12410	0.37931	0.02124	0.25181	0.22347

Comparison of interacting and non-interacting C-H bonds

Table 32: Comparison of NBO analysis results of interacting and non-interacting PCy_3 C-H bonds of the structures optimized with the PCM model (aniline as solvent):

	**Interacting**			**non-interacting**		
		**C-H [Å] calc.**	**occ(σ*)**		**C-H [Å] calc.**	**occ(σ*)**
$^{Cy}Y_S$-PCy_2-AuCl (**P1**)	H9	1.10039	0.03795	H21	1.09987	0.02473

				H15	1.09499	0.02785
$^{Cy}Y_{S}$-PCy_2-Au^{+}pa	H9	1.10073	0.03572	H21<br>H15	1.09967<br>1.09536	0.02430<br>0.02788
$^{Cy}Y_{Mes}$-PCy_2-AuCl (**P3**)	H23	1.10051	0.02994	H17<br>H11	1.09992<br>1.10183	0.02453<br>0.02381
$^{Cy}Y_{Mes}$-PCy_2-Au^{+}pa	H23	1.10075	0.02872	H17<br>H11	1.09968<br>1.10184	0.02444<br>0.02386
$^{Cy}Y_{SF}$-PCy_2-AuCl (**P5**)	H6<br>H12	1.10113<br>1.10096	0.03262<br>0.03969	H18	1.09656	0.02785
$^{Cy}Y_{SF}$-PCy_2-Au^{+}pa	H6<br>H12	1.10182<br>1.10146	0.02981<br>0.03723	H18	1.09656	0.02464

4.4 NBOs involved in the donor-acceptor bonding with the associated second order pertubation energies

Representation of all involved NBOs on the example of **P5** with the associated second order pertubation energies.

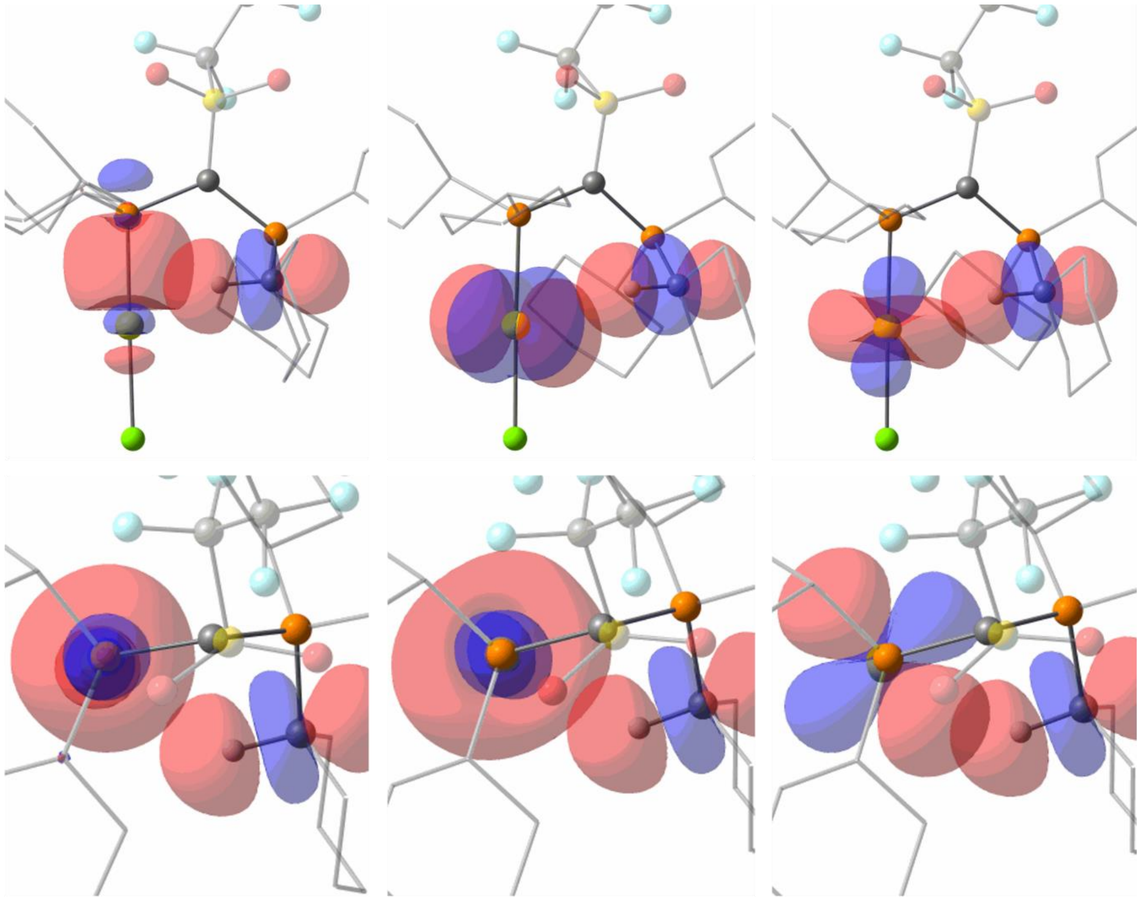

$n_{Au} \rightarrow \sigma^*_{C\text{-}H}$ = 4.2 kJ/mol $n_{Au} \rightarrow \sigma^*_{C\text{-}H}$ = 2.6 kJ/mol $n_{Au} \rightarrow \sigma^*_{C\text{-}H}$ = 12.0 kJ/mol

For the other structures the strongest orbital interaction is depicted with the associated second order pertubation energies, aswell as the sum of all order pertubation energies from the interaction of the σ*(CH) orbital with all n_{Au} orbitals.

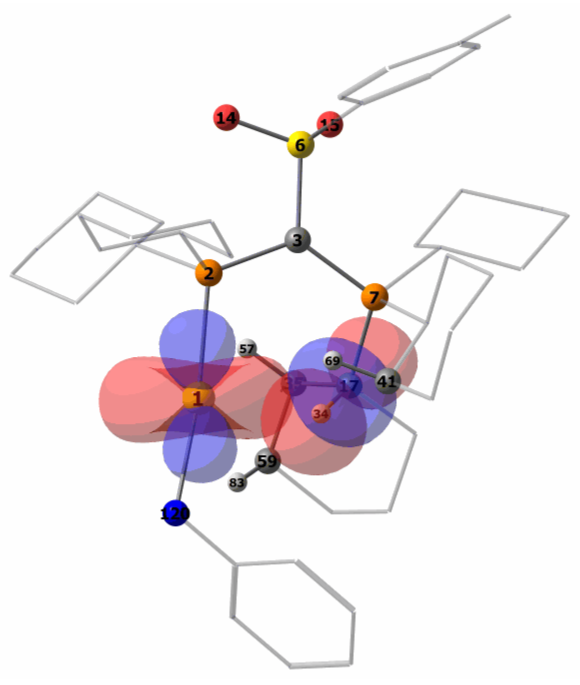

L1-Au(an)$^{+}$

$n_{Au} \rightarrow \sigma^*_{C\text{-}H}$ = 6.4 kJ/mol

$\sum n_{Au} \rightarrow \sigma^*_{C\text{-}H}$ = 15.3 kJ/mol

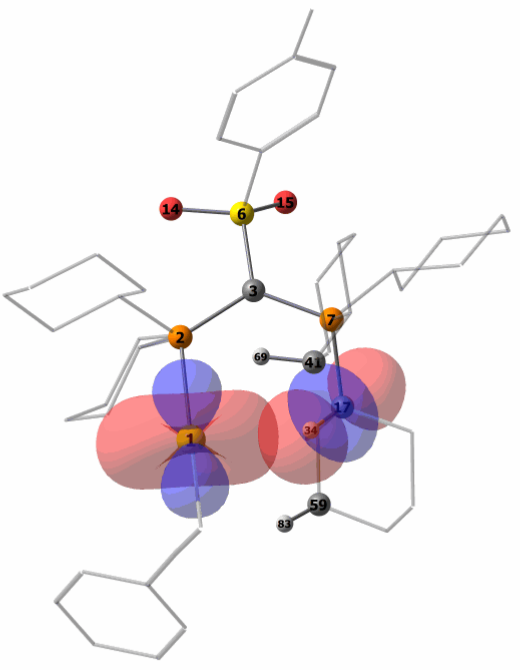

L1-Au(pa)$^{+}$

$n_{Au} \rightarrow \sigma^*_{C\text{-}H}$ = 7.6 kJ/mol

$\sum n_{Au} \rightarrow \sigma^*_{C\text{-}H}$ = 12.1 kJ/mol

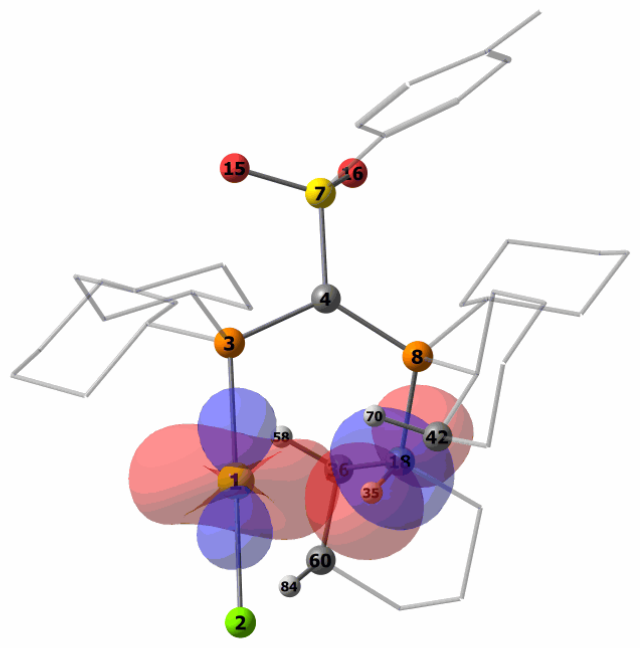

L1-AuCl

$n_{Au} \rightarrow \sigma^*_{C\text{-}H}$ = 10.6 kJ/mol

$\sum n_{Au} \rightarrow \sigma^*_{C\text{-}H}$ = 14.4 kJ/mol

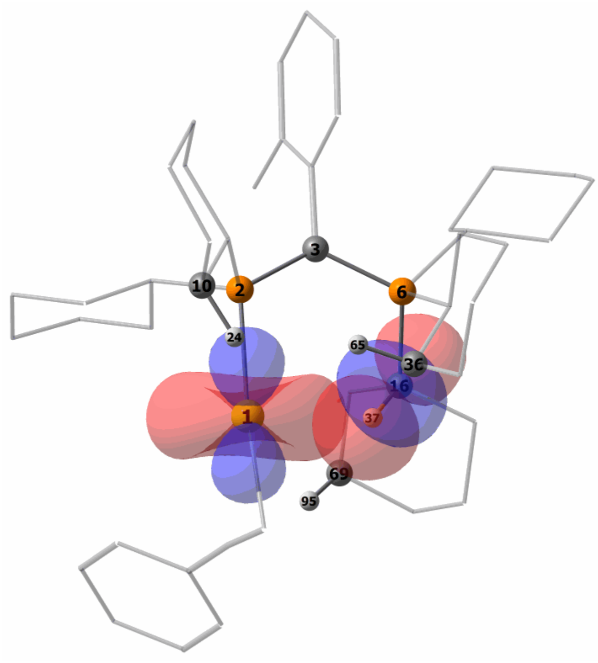

L2-Au(pa)$^{+}$

$n_{Au} \rightarrow \sigma^*_{C\text{-}H}$ = 6.6 kJ/mol

$\sum n_{Au} \rightarrow \sigma^*_{C\text{-}H}$ = 10.3 kJ/mol

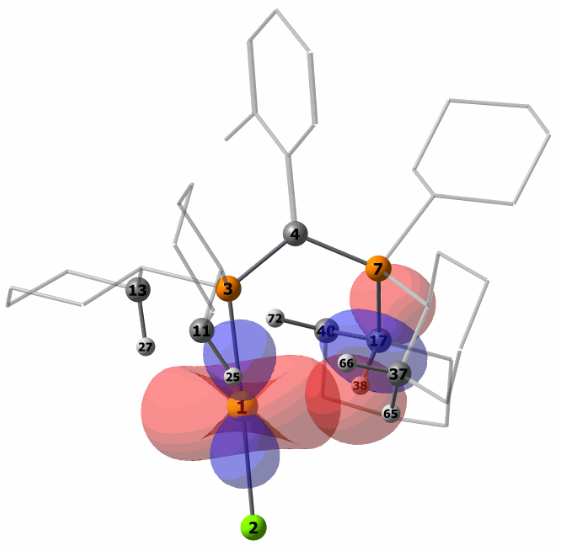

L2-AuCl

$n_{Au} \rightarrow \sigma^*_{C\text{-}H}$ = 6.5 kJ/mol

$\sum n_{Au} \rightarrow \sigma^*_{C\text{-}H}$ = 8.7 kJ/mol

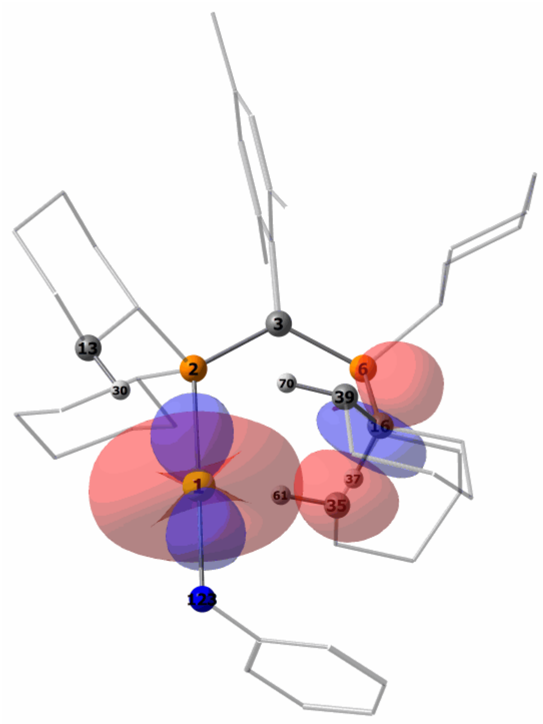

L3-Au(an)$^{+}$

$n_{Au} \rightarrow \sigma^*_{C\text{-}H}$ = 7.4 kJ/mol

$\sum n_{Au} \rightarrow \sigma^*_{C\text{-}H}$ = 14.0 kJ/mol

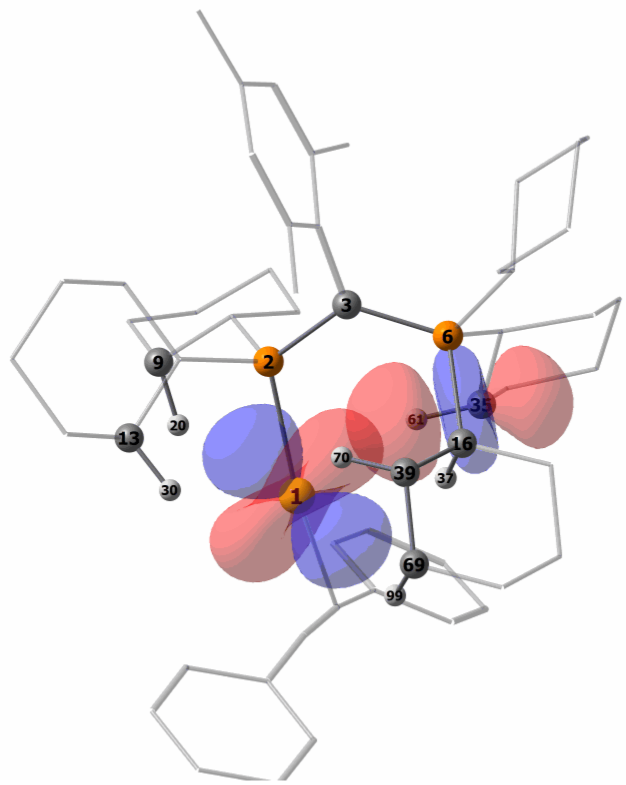

L3-Au(dpa)$^{+}$

$n_{Au} \rightarrow \sigma^*_{C\text{-}H}$ = 7.6 kJ/mol

$\sum n_{Au} \rightarrow \sigma^*_{C\text{-}H}$ = 13.1 kJ/mol

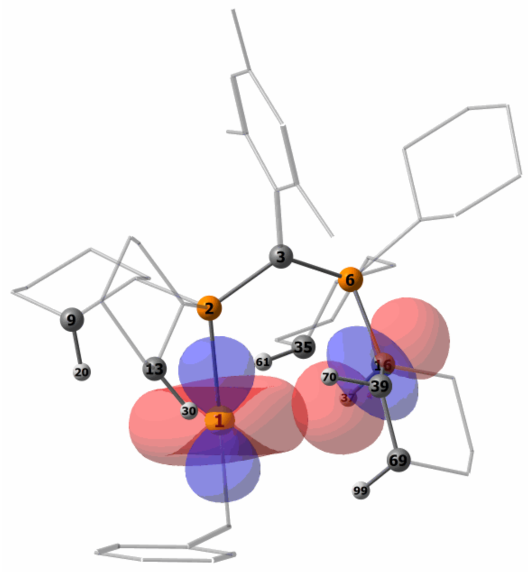

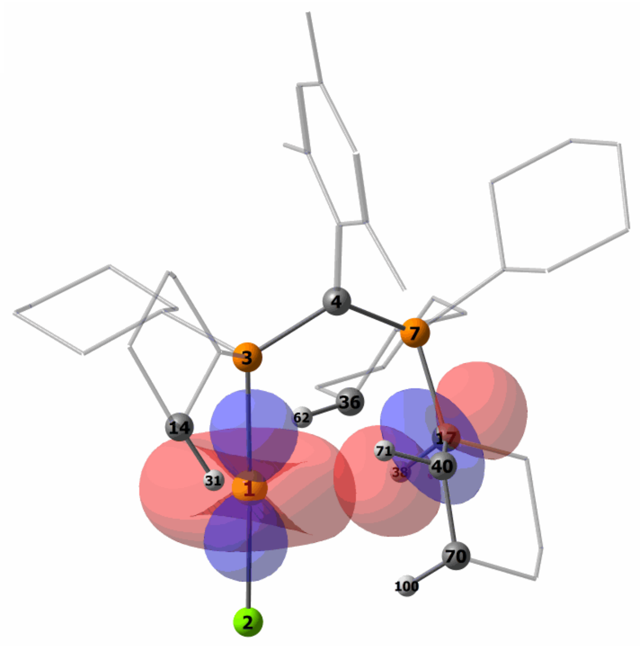

L3-Au(pa)$^{+}$

$n_{Au} \rightarrow \sigma^*_{C\text{-}H}$ = 7.9 kJ/mol

$\sum n_{Au} \rightarrow \sigma^*_{C\text{-}H}$ = 14.9 kJ/mol

L3-AuCl

$n_{Au} \rightarrow \sigma^*_{C\text{-}H}$ = 6.7 kJ/mol

$\sum n_{Au} \rightarrow \sigma^*_{C\text{-}H}$ = 15.31 kJ/mol

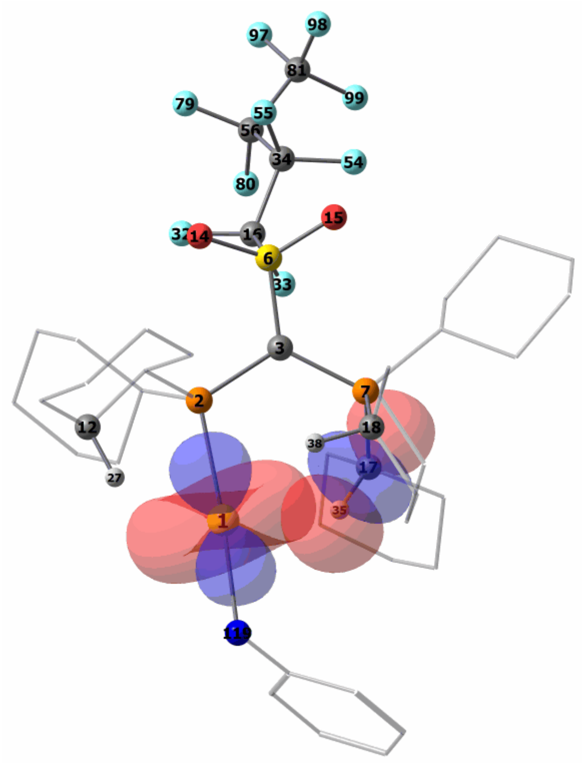

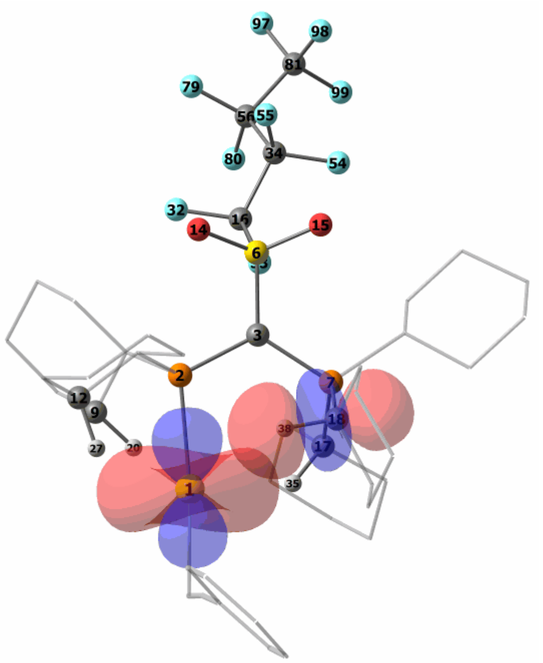

L5-Au(an)$^{+}$

$n_{Au} \rightarrow \sigma^*_{C\text{-}H}$ = 5.2 kJ/mol

$\sum n_{Au} \rightarrow \sigma^*_{C\text{-}H}$ = 15.5 kJ/mol

L5-Au(pa)$^{+}$

$n_{Au} \rightarrow \sigma^*_{C\text{-}H}$ = 10.8 kJ/mol

$\sum n_{Au} \rightarrow \sigma^*_{C\text{-}H}$ = 15.7 kJ/mol

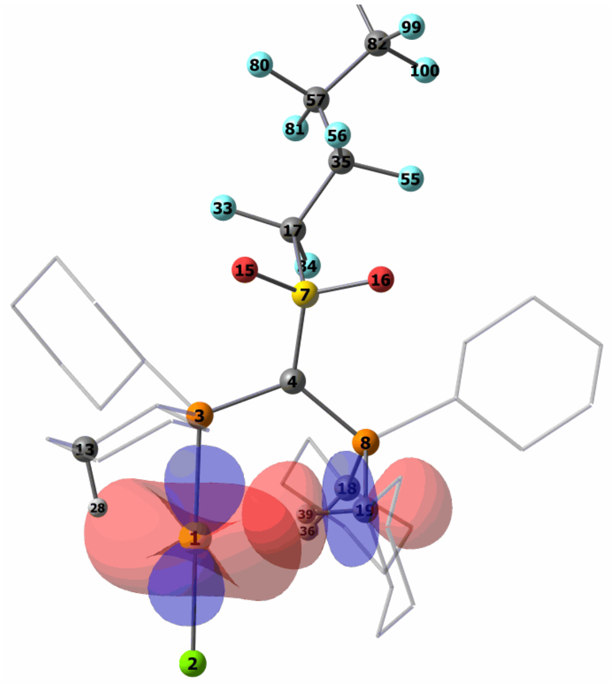

L5-AuCl

$n_{Au} \rightarrow \sigma^*_{C\text{-}H}$ = 12.0 kJ/mol

$\sum n_{Au} \rightarrow \sigma^*_{C\text{-}H}$ = 18.8 kJ/mol

4.5 NCI plots

NCI plots for the studied complexes. Coloured in a blue-green-red scheme over the range of −0.035 < sign(λ2)ρ < 0.02 and isosurface of RDG = 0.5. Blue indicates strong attraction, green indicates weak interaction, and red indicates repulsion.

4.5.1 LAuCl complexes

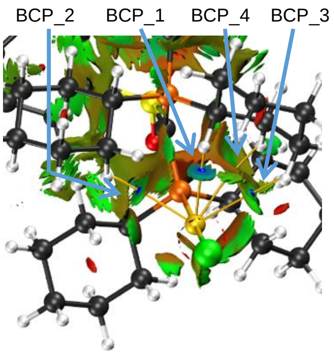

(P1) $^{Cy}Y_S$-PCy$_2$

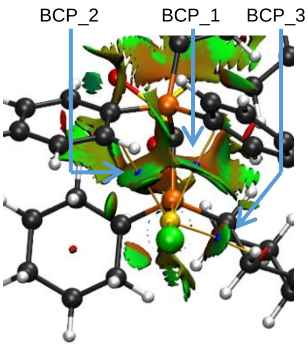

(A) $^{Ph}Y_S$-PCy$_2$

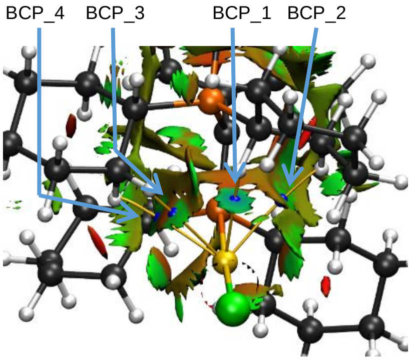

(P2) $^{Cy}Y_{oTol}$-PCy$_2$

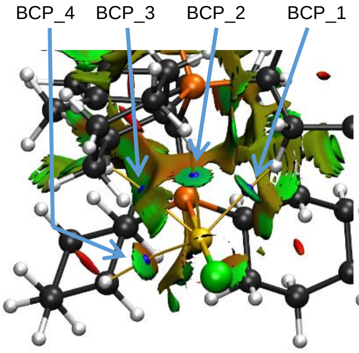

(P3) $^{Cy}Y_{Mes}$-PCy$_2$

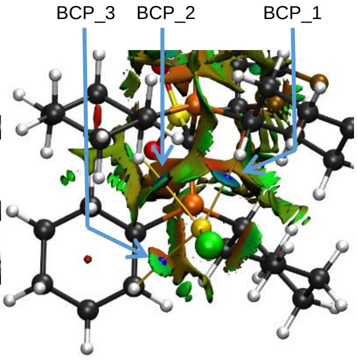

(P5) $^{Cy}Y_{SF}$-PCy$_2$

4.5.2 LAu$^+$ complexes

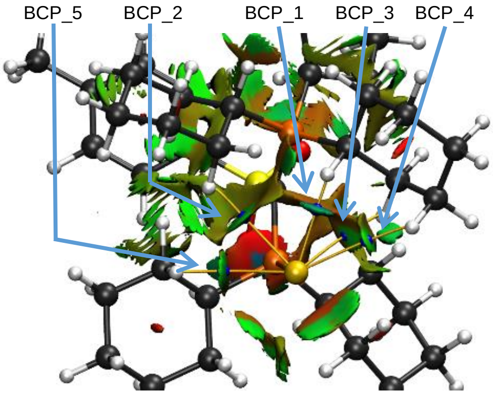

(P1) $^{Cy}Y_S$-PCy$_2$

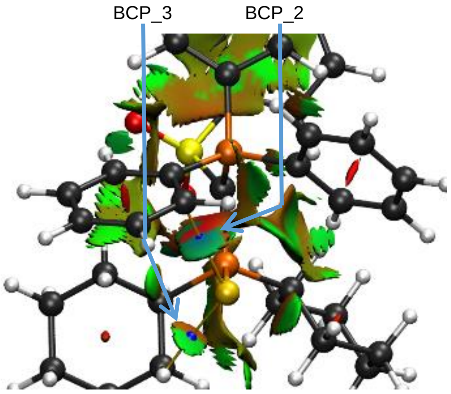

(A) $^{Ph}Y_S$-PCy$_2$

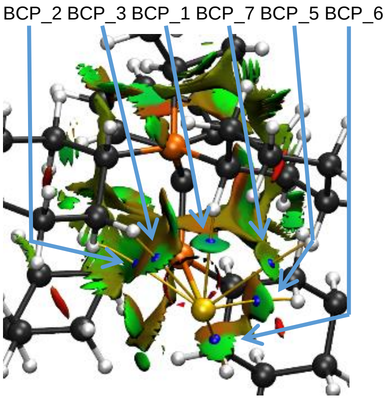

(P2) $^{Cy}Y_{oTol}$-PCy$_2$

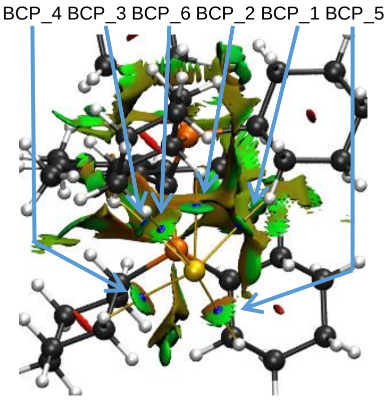

(P3) $^{Cy}Y_{Mes}$-PCy$_2$

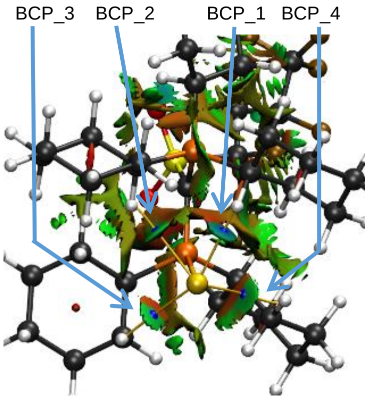

(P5) $^{Cy}Y_{SF}$-PCy$_2$

4.5.3 NCI scatter graphs

The scatter graphs show the reduced density gradient (RDG) against sign(λ_2)ρ over the range of −0.035 < sign(λ_2)ρ < 0.02. Every point corresponds to a grid point in 3D space.

P1·AuCl

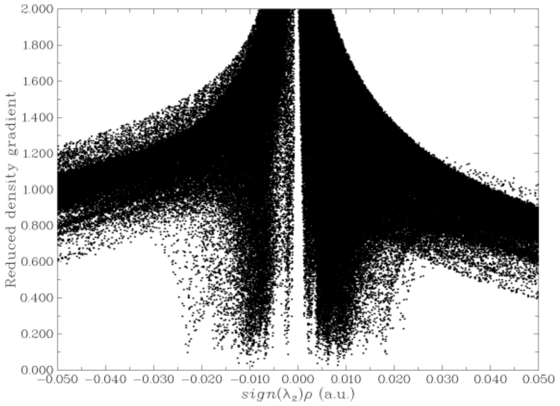

P1·Au⁺

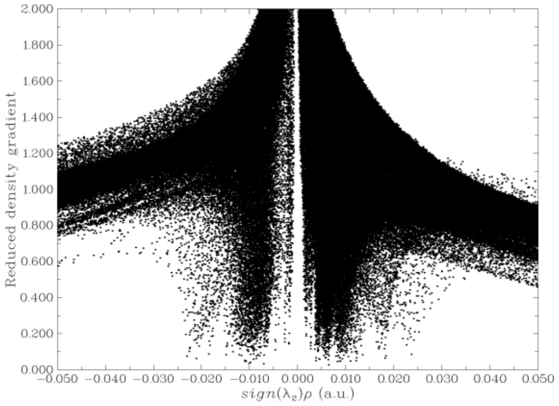

A·AuCl

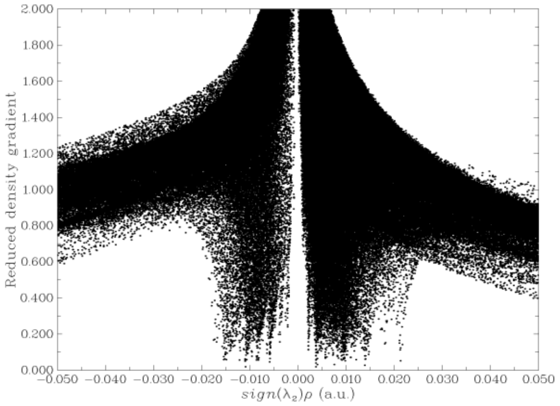

A·Au⁺

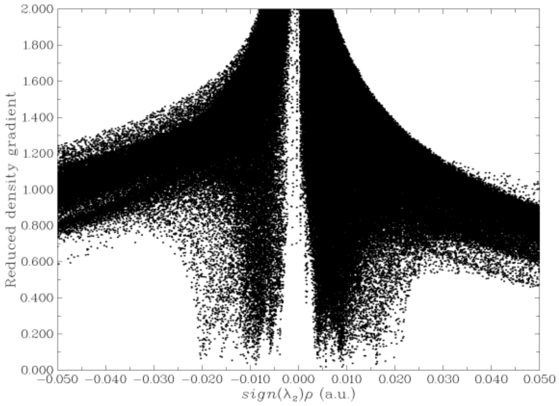

P2·AuCl

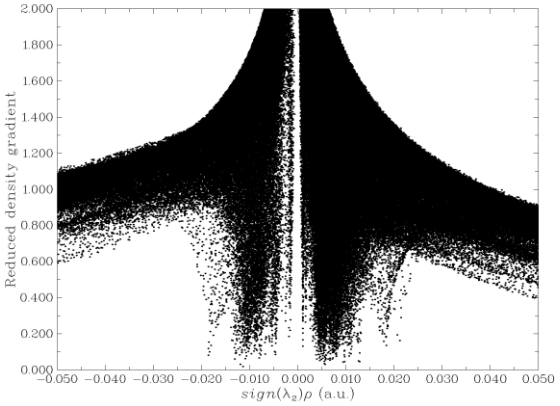

P2·Au⁺

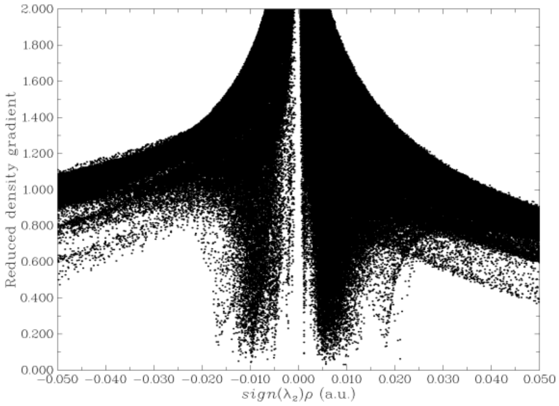

P3·AuCl

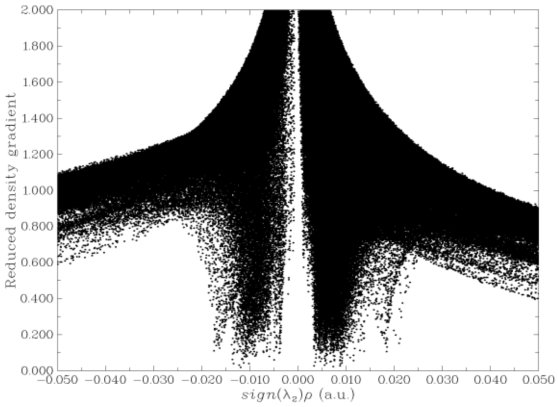

P3·Au⁺

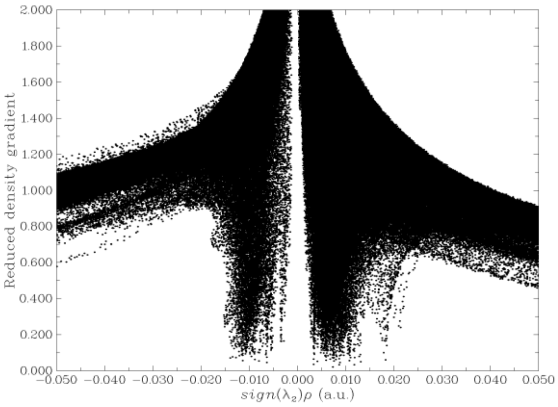

P5·AuCl

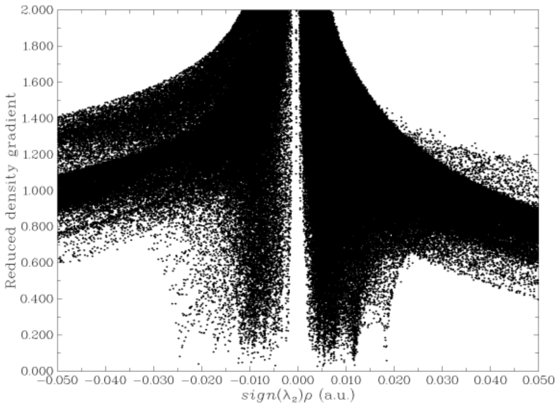

P5·Au⁺

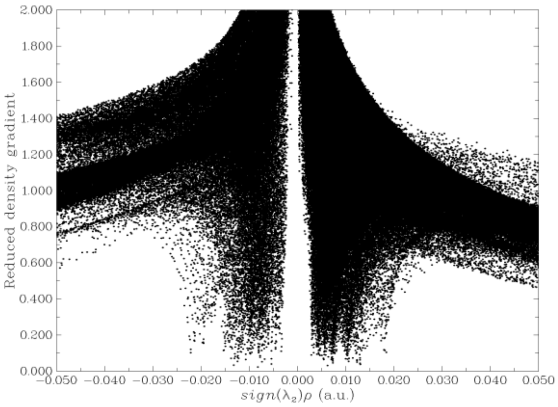

4.6 Energies and coordinates of the structures

4.6.1 Energies and coordinates of LAuCl complexes:

	**E(SCF)**	**Corr(H)**	**Corr(G)**	**ΔG [kJ/mol]**
**$^{Cy}Y_{S}PCy_{2}$ (P1)**	-3316.97627174	1.002261	0.874006	–
**$^{Ph}Y_{S}PCy_{2}$ (A)**	-3306.09630934	0.788503	0.664006	–
**$^{Cy}Y_{oTol}PCy_{2}$ (P2)**	-2767.76119760	0.986673	0.867730	–
**$^{Cy}Y_{Mes}PCy_{2}$ (P3)**	-2846.51986514	1.046765	0.919248	–
**$^{Cy}Y_{SF}PCy_{2}$ (P5)**	-4098.13743602	0.942299	0.803451	–

4.7 Computational details to the calculations of the NMR chemical shifts and C-H vibration in P5

Geometries of **P5** complex and free ligand **L5** were optimized at PBE0 (*Phys. Rev. Lett.* **1996**, *77* (18), 3865–3868) functional with Becke-Johson damped D3 dispersion correction (J. Comput. Chem., 32: 1456-1465.) as implemented in Gaussian 09 (Gaussian 09, Revision A.02,. Gaussian, Inc., Wallingford CT, 2016.) The TZVP (J. Chem. Phys. 1992, 97 (4), 2571–2577.) basis set was used for nonmetal atoms whereas the SDD (Theor. Chim. Acta 1990, 77 (2), 123–141.) pseudopotential and its associated double-ζ basis set was employed for Au. Solvent effects were included using SMD (J. Phys. Chem. B, 2009, **113**, 6378-6396.) continuum solvation model in geometry optimizations and dichloromethane (DCM) solvent parameters (ε = 8.9) were adjusted to mimic the experimental NMR analysis. The natural population analysis (NPA) was carried out based on the natural bond orbital (NBO) method followed by Wiberg bond index (WBI) analysis as implemented in Gaussian 09. Structural characterization of the **P5** complex and free ligand **L5** was done based on ^{1}H NMR calculations in Amsterdam Density Functional (ADF) software suite (J. Comput. Chem. 2001, 22 (9), 931–967) using GGA BP86 (*Phys. Rev. A* **1988**, *38* (6), 3098–3100.) functional and all-electron triple-ζ basis set with two polarization functions. Spin-orbit coupling (SOC) (*Chem. Phys. Lett.* **1999**, *306* (5), 357–365.) through zero-order regular approximation (ZORA) treatment (J. Chem. Phys. 1994, 101 (11), 9783–9792.) was also included to account for relativistic effects which in particular, are critical for Au. Chemical shifts were calculated relative to TMS based on the following equation :

$\Delta\delta = \sigma_{TMS} - \sigma_{H}$ where σ_{T} = DFT magnetic shielding

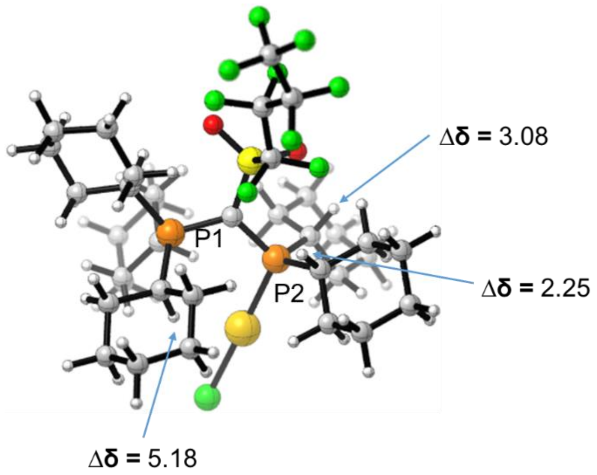

Table 33. Predicted ^{1}H NMR chemical shifts for complex **P5**. For comparison, Δδ values of H bound to the two PCy groups bound to P2 are also reported.

Atom	d(Au-H) (Å)	Δδ (ppm)
P1-H1	5.88	3.99
P1-H2	2.66	3.89
P1-H3	2.44	5.18
P2-H1		2.25
P2-H2		3.08

The chemical shifts, Δδ, for all three H atoms are deshielded moving from free ligand **L5** to the **P5** complex (Table 34). Deshielding upon complexation is more pronounced for the H3 atom, 4.00 ppm, followed by H2 and H1 and the Δ_{shift} of the three H atoms increases with decreasing Au-H distances, which is Au-H1 > Au-H2 > Au-H3. Variation in J values follows variation in the C-H bond lengths such that the former is reduced with the elongated C-H3 bond, and it increases with the shortened C-H1 bond. The positive charge on H3 decreases significantly from **L5** to **P5** pointing out electrons flow from Au to H that can further support a favorable interaction between Au and H3. The C-H3 bond is associated with a strong reduction in the wavelength of the v(C-H) vibration upon complexation. The softer C-H3 vibrational mode in **P5** is more reasonably explained with attractive AuH-bonding interaction, rather than steric compression since the latter should result in a stiffer C-H vibration, with an increased in wavelength. Changes in the Wiberg bond index, WBI, for the three C-H bonds are almost the same thus WBI doesn't provide any additional insight.

Table 34. ^{1}H NMR, NBO and Structural Analysis. Δδ = chemical shift, J(C-H) = Coupling constant, q = NPA charge, d(C-H) = bond distance, v(C-H) = vibrational frequency, WBI = Wiberg Bond Index. P5-L5 = difference between P5 and L5 values.

P5	Δδ (ppm)	J(C-H) (Hz)	q_H	d(C-H) (Å)	v(C-H) (cm^{-1})	WBI (C-H)	WBI (Au-H)	d(Au-H) (Å)
P1-H1	3.99	132	0.284	1.092	3102	0.857	0.0001	5.876
P1-H2	3.89	118	0.270	1.097	3028	0.853	0.0124	2.656
P1-H3	5.18	120	0.262	1.099	3011	0.851	0.0209	2.437
**L5**								

H1	2.98	124	0.261	1.097	3036	0.864
H2	2.84	125	0.259	1.097	3041	0.871
H3	4.00	131	0.283	1.093	3093	0.857
**P5-L5**						
H1	1.01	8	0.023	-0.005	65	-0.007
H2	1.05	-7	0.011	0.000	-13	-0.018
H3	1.18	-11	-0.021	0.006	-82	-0.006

Cartesian coordinates of all the systems considered in this work.

$^{Cy}Y_SPCy_2$ (P1)

Au -2.422917 -0.216145 -1.011903
Cl -3.632837 0.527609 -2.867380
P -1.099759 -0.863669 0.740814
C 0.474936 -0.009895 0.773411
C -1.984844 -0.719522 2.375438
C -0.832471 -2.684324 0.513335
S 1.526791 -0.508378 2.076176
P 0.892400 1.297659 -0.335651
H -1.726030 -1.656352 2.890737
C -3.498235 -0.652207 2.155256
C -1.546200 0.425817 3.286073
H -0.155236 -2.987688 1.321707
C -2.130428 -3.480069 0.645836
C -0.190774 -2.957078 -0.843469
O 0.812434 -1.589345 2.772320
O 2.020101 0.618351 2.879980
C 3.003539 -1.265254 1.411135
C -0.516472 2.399619 -0.774215
C 2.252744 2.278200 0.417693
C 1.547989 0.739153 -1.970574
H -3.732229 0.296651 1.646119
H -3.836050 -1.442327 1.473314
C -4.247180 -0.725760 3.479131
C -2.280992 0.343939 4.619168
H -0.467949 0.408187 3.455989
H -1.778744 1.389058 2.813178
H -2.578886 -3.329347 1.634804
H -2.857066 -3.110237 -0.096119
C -1.878448 -4.968754 0.427575
C 0.080551 -4.440427 -1.046573
H -0.877500 -2.607503 -1.630681
H 0.729651 -2.371056 -0.950984
C 4.218462 -0.596400 1.461311
C 2.921620 -2.539992 0.860438
H -1.115542 1.758932 -1.438953
C -1.444871 2.799745 0.368512
C -0.109335 3.614703 -1.611596
H 2.868512 1.509671 0.896490

C 3.177260 3.042790 -0.532076
C 1.739859 3.199751 1.527379
H 1.924610 1.659668 -2.441510
C 0.491048 0.134486 -2.892908
C 2.716955 -0.221134 -1.764999
H -5.328316 -0.659536 3.301332
H -4.065360 -1.709338 3.939211
C -3.791590 0.369134 4.433304
H -1.988144 -0.586323 5.128816
H -1.959430 1.167593 5.269551
H -1.237566 -5.342896 1.240354
H -2.825450 -5.518749 0.500159
C -1.203627 -5.245062 -0.908575
H 0.539029 -4.606633 -2.030530
H 0.810400 -4.787459 -0.297811
H 4.280537 0.362982 1.970333
C 5.338899 -1.174374 0.873207
C 4.044958 -3.105950 0.280538
H 1.980243 -3.081454 0.893522
H -1.735890 1.907661 0.930841
H -0.931035 3.466949 1.071054
C -2.685100 3.484062 -0.192842
C -1.361264 4.286039 -2.168042
H 0.442650 4.332109 -0.987910
H 0.556216 3.326734 -2.435238
H 3.566859 2.393157 -1.323019
H 2.632288 3.857529 -1.026907
C 4.348465 3.613641 0.264256
C 2.912886 3.746665 2.325696
H 1.191558 4.035580 1.070048
H 1.049876 2.656483 2.180394
H 0.015945 -0.713635 -2.384348
H -0.312775 0.848848 -3.105380
C 1.105201 -0.341700 -4.203514
C 3.316264 -0.685828 -3.083843
H 2.348888 -1.094346 -1.215677
H 3.489958 0.229082 -1.130092
H -4.086876 1.346923 4.022097
H -4.301375 0.272425 5.400758
H -1.002499 -6.318146 -1.021242
H -1.888050 -4.967136 -1.724240
H 6.291175 -0.646804 0.903867
C 5.267492 -2.422966 0.253895
H 3.976448 -4.100742 -0.157908
H -3.344919 3.785221 0.630824
H -3.244424 2.756748 -0.800426
C -2.319281 4.684254 -1.054161
H -1.076296 5.161029 -2.766205
H -1.868382 3.584499 -2.846754
H 5.011415 4.176635 -0.405186
H 4.943043 2.774245 0.657645
C 3.888642 4.487450 1.422789
H 3.412292 2.902340 2.822784
H 2.548098 4.407048 3.122351
H 0.323682 -0.800240 -4.821550

H 1.476595 0.528777 -4.766270
C 2.252954 -1.312982 -3.972204
H 3.770900 0.170520 -3.605558
H 4.129077 -1.396705 -2.882999
C 6.461763 -3.024984 -0.421580
H -3.223624 5.137781 -1.478540
H -1.845772 5.456309 -0.427136
H 3.396454 5.386113 1.019862
H 4.755188 4.840941 1.996159
H 2.686211 -1.631950 -4.928698
H 1.866056 -2.221605 -3.484653
H 6.358809 -2.969628 -1.514019
H 6.576102 -4.084042 -0.162959
H 7.384480 -2.503054 -0.148911

$^{Ph}Y_SPCy_2$ (A)
Au 2.329648 -0.327729 0.869620
Cl 3.634396 -1.833831 2.093889
P 0.945893 1.106617 -0.262298
C -0.618211 0.281730 -0.509295
C 1.729171 1.570549 -1.864977
C 0.639834 2.668909 0.707312
S -1.717382 0.856450 -1.707265
P -0.777342 -1.379927 -0.009740
C 1.684885 0.379569 -2.817482
H 1.133518 2.387010 -2.290888
C 3.168231 2.032032 -1.632398
C 1.685729 2.881545 1.803746
H -0.320348 2.455769 1.202840
C 0.454720 3.930500 -0.136273
O -1.212005 2.154146 -2.171925
C -3.290485 1.161449 -0.922587
O -1.997488 -0.178408 -2.719210
C -0.417471 -1.595957 1.753170
C -2.522829 -1.886768 -0.111944
C 0.264429 -2.513744 -0.971256
C 2.396295 0.689193 -4.126790
H 2.161289 -0.487354 -2.328878
H 0.643340 0.102356 -3.013020
C 3.857446 2.356015 -2.951967
H 3.197654 2.901722 -0.962923
H 3.718681 1.228022 -1.119109
C 1.342463 4.090099 2.665127
H 2.674488 3.021463 1.341166
H 1.770260 1.978980 2.424172
C 0.105971 5.124036 0.744176
H 1.390040 4.144642 -0.673577
H -0.315770 3.763154 -0.894077
C -4.444472 0.692712 -1.530451
C -3.349052 1.887684 0.261911
C -0.320044 -2.883052 2.284839
C -0.311593 -0.487086 2.595436
C -3.360225 -1.456492 0.923963
C -3.049279 -2.651354 -1.151194
C 0.057418 -2.555238 -2.356689

C 1.353179 -3.190377 -0.413452
C 3.824445 1.161303 -3.894594
H 2.382491 -0.198901 -4.772188
H 1.835637 1.471669 -4.659770
H 4.891888 2.670583 -2.762730
H 3.351055 3.210023 -3.427008
C 1.150658 5.346393 1.827719
H 0.412360 3.880601 3.217118
H 2.123548 4.242064 3.421066
H -0.006338 6.022148 0.123399
H -0.874847 4.949658 1.214335
H -4.363362 0.113401 -2.446938
C -5.673607 0.947961 -0.932404
C -4.579847 2.123816 0.851756
H -2.430304 2.242578 0.724657
H -0.446539 -3.752872 1.643153
C -0.044236 -3.053831 3.634978
C -0.048488 -0.664460 3.946932
H -0.424359 0.512180 2.181628
H -2.956347 -0.858679 1.738188
C -4.707133 -1.786230 0.916765
H -2.413095 -2.994601 -1.960271
C -4.401263 -2.977068 -1.153941
H -0.733302 -1.955415 -2.804513
C 0.905180 -3.303913 -3.161508
C 2.208138 -3.920335 -1.232226
H 1.580872 -3.120558 0.646799
H 4.410178 0.340435 -3.453283
H 4.306526 1.411880 -4.848472
H 2.108141 5.614489 1.355260
H 0.871649 6.194261 2.466727
H -6.582907 0.572777 -1.399926
C -5.763311 1.657481 0.264725
H -4.628635 2.681711 1.786114
H 0.059351 -4.057600 4.040769
C 0.106230 -1.946366 4.462423
H 0.052981 0.203631 4.594593
C -5.229644 -2.547643 -0.124062
H -5.349748 -1.443424 1.724778
H -4.805615 -3.574393 -1.968337
H 0.737306 -3.331419 -4.236274
C 1.980661 -3.987679 -2.601207
H 3.066104 -4.418929 -0.786978
C -7.087979 1.922577 0.914179
H 0.338259 -2.083958 5.516337
H -6.286451 -2.807722 -0.131571
H 2.654157 -4.558535 -3.237749
H -7.099199 1.571491 1.953726
H -7.903798 1.426225 0.379153
H -7.307406 2.997925 0.937588

$^{Cy}Y_{oTol}PCy_2$ (P2)
Au -1.760369 -1.572826 0.285429

Cl -2.172327 -3.835627 0.696305
P -1.199490 0.596542 -0.168121
C 0.529494 0.855180 -0.475114
C -1.643869 1.750154 1.231447
C -2.250230 1.109309 -1.605556
P 1.674031 -0.297245 0.148744
C 0.997604 2.247453 -0.782757
H -0.692109 1.736361 1.785877
C -1.904911 3.207742 0.843310
C -2.709953 1.241823 2.200055
H -1.927979 2.121433 -1.902027
C -2.086538 0.142002 -2.778861
C -3.731768 1.135901 -1.217578
C 3.345457 0.360319 -0.287501
C 1.797874 -0.589270 1.985909
C 1.448900 -2.002996 -0.525525
C 1.394016 3.111625 0.254758
C 1.065806 2.753544 -2.101342
H -1.155091 3.569363 0.132154
H -2.877264 3.291707 0.340230
C -1.922235 4.082092 2.091008
C -2.748759 2.121974 3.444201
H -3.695546 1.240232 1.713198
H -2.506020 0.197564 2.473263
H -1.039076 0.049726 -3.068685
H -2.400934 -0.857687 -2.439039
C -2.933397 0.562194 -3.972390
C -4.607099 1.555199 -2.393038
H -4.014596 0.123881 -0.885343
H -3.915780 1.800275 -0.369362
H 3.248226 1.423051 -0.021205
C 3.607712 0.301807 -1.792978
C 4.553646 -0.180633 0.480903
H 2.672444 -1.251956 2.080392
C 2.085785 0.698853 2.755958
C 0.610418 -1.302605 2.629761
H 0.619658 -2.401337 0.077499
C 2.630001 -2.949685 -0.309251
C 0.971707 -2.036483 -1.974585
H 1.344352 2.742755 1.274451
C 1.826041 4.410370 0.034181
C 1.494165 4.070392 -2.310294
C 0.727495 1.928447 -3.305420
H -0.921969 4.063306 2.552211
H -2.117682 5.127272 1.818040
C -2.957508 3.590166 3.094529
H -1.793902 2.010890 3.982916
H -3.532899 1.775130 4.129549
H -2.588112 1.544061 -4.334334
H -2.787010 -0.143099 -4.800707
C -4.405370 0.648715 -3.597882
H -5.661270 1.558603 -2.086893
H -4.359938 2.591672 -2.670345
H 3.768473 -0.744548 -2.090101
H 2.733141 0.656776 -2.343740

C 4.839963 1.119678 -2.151922
C 5.798220 0.625161 0.116971
H 4.398603 -0.128499 1.563726
H 4.718488 -1.237601 0.236046
H 2.905777 1.269923 2.302844
H 1.192043 1.335593 2.695784
C 2.378228 0.414132 4.223626
C 0.881188 -1.593859 4.101237
H 0.360838 -2.235475 2.112372
H -0.278866 -0.659501 2.548215
H 3.478186 -2.635818 -0.934879
H 2.975545 -2.925732 0.732315
C 2.225612 -4.369557 -0.693058
C 0.587415 -3.457988 -2.363560
H 1.758260 -1.656085 -2.642433
H 0.112596 -1.368540 -2.085605
H 2.118975 5.036898 0.874699
C 1.869659 4.900757 -1.265957
H 1.545280 4.442567 -3.333305
H 1.402266 2.164130 -4.136629
H -0.293659 2.125965 -3.657280
H 0.796341 0.860737 -3.078995
H -3.961412 3.715136 2.660211
H -2.935539 4.206069 4.003047
H -4.770568 -0.359976 -3.353264
H -5.003027 1.000047 -4.448962
H 5.021263 1.070697 -3.233269
H 4.645725 2.176820 -1.913803
C 6.059201 0.632605 -1.382625
H 5.662101 1.660483 0.465269
H 6.667196 0.227329 0.656650
H 3.294062 -0.192262 4.299051
H 2.582380 1.353989 4.752646
C 1.220189 -0.328570 4.873909
H 0.009165 -2.094583 4.539733
H 1.718991 -2.303915 4.177626
H 3.072984 -5.051147 -0.543086
H 1.422696 -4.705558 -0.020351
C 1.732863 -4.433425 -2.131957
H -0.279198 -3.761495 -1.757045
H 0.265262 -3.483071 -3.412660
H 2.200915 5.918031 -1.465919
H 6.935762 1.251364 -1.613390
H 6.304962 -0.389338 -1.710024
H 0.337524 0.329912 4.890431
H 1.451602 -0.564175 5.920503
H 1.419687 -5.455531 -2.379569
H 2.566111 -4.188511 -2.809811

$^{Cy}Y_{Mes}PCy_2$ (P3)
Au 2.359683 -0.807570 -0.540651
Cl 3.702095 -2.613097 -1.165581
P 0.971777 0.898047 0.096574
C -0.770755 0.532681 0.093299
C 1.564802 1.464025 1.763340

C 1.186237 2.389138 -0.996856
P -1.368783 -1.090601 0.003326
C -1.681759 1.729975 0.163254
H 0.927275 2.317227 2.044649
C 3.024099 1.920066 1.738714
C 1.421427 0.350494 2.794330
H 0.395495 2.216625 -1.734167
C 0.912464 3.744630 -0.336767
C 2.498822 2.419259 -1.782613
C -3.197261 -1.155380 -0.185319
C -1.110718 -2.140382 1.548452
C -0.625125 -2.118202 -1.349060
C -2.093796 2.421743 -1.003776
C -2.058907 2.290180 1.411581
H 3.182667 2.718151 1.007088
H 3.646630 1.072028 1.411021
C 3.473701 2.391873 3.117631
C 1.832143 0.816990 4.182971
H 0.399949 -0.042074 2.794485
H 2.076442 -0.480276 2.488284
H -0.025226 3.732038 0.229893
H 1.711091 3.973015 0.382790
C 0.873396 4.845936 -1.389322
C 2.468104 3.533466 -2.822740
H 3.349160 2.570840 -1.102763
H 2.668479 1.449192 -2.267071
H -3.365410 -2.244187 -0.160812
C -3.774494 -0.648234 -1.502873
C -3.977650 -0.540740 0.980567
H -1.172445 -1.372155 2.333599
C 0.289910 -2.747274 1.611151
C -2.133395 -3.222639 1.919674
H 0.350817 -2.402602 -0.927818
C -1.381095 -3.417029 -1.629822
C -0.319927 -1.377965 -2.648994
C -2.768178 3.640761 -0.898705
C -1.846139 1.924957 -2.400561
C -2.728441 3.510146 1.468633
C -1.800465 1.593862 2.715292
H 4.527366 2.697776 3.081604
H 2.898353 3.289653 3.392063
C 3.269123 1.319901 4.178812
H 1.712240 -0.000561 4.906522
H 1.159011 1.625383 4.510346
H 0.020407 4.663926 -2.062058
H 0.685360 5.815773 -0.910185
C 2.163593 4.887378 -2.196066
H 1.692010 3.299801 -3.568580
H 3.420864 3.565787 -3.366854
H -3.700712 0.443445 -1.521432
H -3.207551 -1.027108 -2.360304
C -5.240726 -1.050724 -1.614515
C -5.444365 -0.945063 0.893033
H -3.555762 -0.831869 1.949376
H -3.898530 0.551021 0.923139

H 0.354250 -3.579481 0.894050
H 1.048167 -2.021222 1.295324
C 0.601762 -3.268833 3.005473
C -1.842363 -3.750771 3.322875
H -3.162317 -2.852466 1.884059
H -2.075656 -4.057571 1.208458
H -1.595170 -3.954484 -0.699249
H -2.353027 -3.188926 -2.094021
C -0.566731 -4.305263 -2.564905
C 0.497827 -2.268469 -3.575788
H 0.230109 -0.454727 -2.429602
H -1.253945 -1.085958 -3.149251
H -3.057518 4.153937 -1.816654
C -3.074190 4.223202 0.323682
H -2.789732 1.837691 -2.953489
H -1.355405 0.952542 -2.397377
H -1.221345 2.627373 -2.967307
H -2.996724 3.912853 2.445997
H -0.871193 1.930408 3.190831
H -1.718757 0.514718 2.579000
H -2.613026 1.795013 3.422593
H 3.549672 1.701146 5.169232
H 3.940300 0.473823 3.967577
H 2.109974 5.664383 -2.969878
H 2.993147 5.169982 -1.529494
H -5.657726 -0.684416 -2.561342
H -5.312163 -2.149072 -1.646941
C -6.051093 -0.522545 -0.438337
H -5.526587 -2.037961 1.005543
H -6.004946 -0.503172 1.726643
H 1.611849 -3.696744 3.023226
H 0.606050 -2.424101 3.712104
C -0.427989 -4.298996 3.445120
H -1.979606 -2.930387 4.044301
H -2.577976 -4.521594 3.587179
H -1.119626 -5.230186 -2.774706
H 0.361785 -4.598973 -2.053453
C -0.217656 -3.582015 -3.857601
H 0.707676 -1.735535 -4.512139
H 1.470212 -2.470020 -3.102060
C -3.745793 5.560157 0.411608
H -7.093875 -0.857507 -0.509110
H -6.071612 0.576748 -0.485280
H -0.233355 -4.628350 4.473852
H -0.333515 -5.192391 2.809410
H 0.401717 -4.225118 -4.495376
H -1.143596 -3.382040 -4.420093
H -3.010769 6.355881 0.595523
H -4.468863 5.593900 1.234758
H -4.270735 5.808400 -0.517065

$^{Cy}Y_{SF}PCy_2$ (P5)
Au -3.025194 -0.702524 0.760643
Cl -4.966891 0.156842 1.720793
P -1.052100 -1.348126 -0.216006

C -0.001992 0.090991 -0.518792
C -0.104523 -2.560034 0.838681
C -1.483731 -2.226070 -1.782679
S 1.421515 -0.174594 -1.382862
P -0.588060 1.708292 -0.049799
H 0.732174 -1.962557 1.227212
C -0.931764 -3.025491 2.039271
C 0.482217 -3.753262 0.080611
H -0.580379 -2.749408 -2.113600
C -2.604520 -3.235120 -1.521214
C -1.904936 -1.254630 -2.875903
O 1.324719 -1.400763 -2.173141
O 1.945114 1.047356 -1.996638
C 2.811109 -0.661432 -0.164328
C -1.073431 1.719017 1.725667
C -2.072040 2.003739 -1.093006
C 0.617054 3.042502 -0.404305
H -1.316142 -2.157137 2.591327
H -1.818244 -3.570185 1.678474
C -0.108022 -3.916012 2.959507
C 1.316211 -4.619116 1.016909
H -0.335190 -4.361930 -0.329167
H 1.080458 -3.407796 -0.767387
H -2.340909 -3.927580 -0.711790
H -3.497316 -2.690226 -1.176595
C -2.938484 -4.001280 -2.795088
C -2.255804 -2.000179 -4.156673
H -2.787694 -0.695585 -2.526387
H -1.102761 -0.531499 -3.060100
F 2.853810 -1.993055 -0.058259
F 2.547363 -0.156451 1.049308
C 4.198582 -0.188495 -0.639013
H -2.072505 1.257881 1.698960
C -0.196211 0.823103 2.598189
C -1.266872 3.089374 2.376120
H -2.488356 0.985534 -1.141665
C -3.187066 2.883883 -0.533798
C -1.670750 2.380046 -2.521232
H 0.898383 2.828655 -1.442645
C 1.885098 2.961815 0.441193
C 0.062383 4.471100 -0.362204
H 0.700110 -3.314000 3.403256
H -0.730338 -4.264547 3.793564
C 0.500088 -5.093231 2.210578
H 1.724149 -5.474240 0.463051
H 2.179725 -4.036533 1.369905
H -2.062166 -4.591117 -3.103547
H -3.745417 -4.718348 -2.597496
C -3.332463 -3.050215 -3.916663
H -2.578555 -1.286595 -4.926108
H -1.346999 -2.485048 -4.542357
F 4.317251 1.128665 -0.425007
F 4.332256 -0.448833 -1.937303
C 5.356623 -0.905541 0.103524
H -0.058025 -0.152476 2.121854

H 0.807878 1.260985 2.697449
C -0.846379 0.647812 3.963962
C -1.935411 2.901169 3.734541
H -0.292880 3.580115 2.511959
H -1.875201 3.747181 1.746080
H -3.487710 2.532236 0.460907
H -2.852346 3.925965 -0.433681
C -4.389235 2.818767 -1.468638
C -2.889058 2.338364 -3.435457
H -1.247325 3.393130 -2.541311
H -0.879579 1.706956 -2.879761
H 1.651863 3.249962 1.476974
H 2.265368 1.942923 0.473708
C 2.940077 3.896745 -0.131602
C 1.106704 5.416697 -0.950008
H -0.876928 4.561789 -0.915131
H -0.145952 4.770925 0.670401
H 1.120353 -5.696202 2.886036
H -0.307609 -5.752903 1.857718
H -4.272536 -2.546849 -3.643221
H -3.535631 -3.608169 -4.839633
F 5.542813 -2.105937 -0.442498
F 5.046243 -1.053554 1.396928
C 6.689482 -0.126567 0.020309
H -1.798389 0.110910 3.825507
H -0.214689 0.014544 4.599950
C -1.116379 1.986981 4.635155
H -2.086049 3.877770 4.211639
H -2.933524 2.464949 3.578437
H -4.787247 1.792849 -1.446398
H -5.188682 3.465570 -1.087174
C -4.018050 3.205810 -2.893860
H -2.604643 2.658066 -4.445842
H -3.239485 1.300396 -3.524236
H 3.857187 3.837208 0.467934
H 3.200502 3.536923 -1.137526
C 2.429368 5.328879 -0.199424
H 1.266540 5.157642 -2.007300
H 0.723539 6.444965 -0.933403
F 7.686453 -0.933135 0.342717
F 6.876561 0.320593 -1.212087
F 6.677798 0.895861 0.858347
H -1.631135 1.836435 5.592209
H -0.157148 2.474255 4.869327
H -4.895988 3.136288 -3.548389
H -3.702181 4.260744 -2.911240
H 2.284377 5.708845 0.823704
H 3.176061 5.981635 -0.668932

4.6.2 Energies and coordinates of LAu^+ complexes:

	E(SCF)	Corr(H)	Corr(G)	ΔG [kJ/mol]
**$^{Cy}Y_SPCy_2$ (P1)**	-2856.12562787	0.999021	0.874168	–

**$^{Ph}Y_SPCy_2$ (A)**	-2845.25115669	0.785218	0.664004	–
**$^{Cy}Y_{oTol}PCy_2$ (P2)**	-2307.08317944	0.983983	0.866091	–
**$^{Cy}Y_{Mes}PCy_2$ (P3)**	-2385.67667538	1.043960	0.920194	–
**$^{Cy}Y_{SF}PCy_2$ (P5)**	-3637.27355916	0.939227	0.804440	–

$^{Cy}Y_SPCy_2$ (P1)

Au -2.452993 -0.169839 -1.339192
P -1.291987 -0.771131 0.562719
C 0.279704 0.038069 0.632347
C -2.417563 -0.451910 2.006625
C -1.174737 -2.603026 0.352548
S 1.134383 -0.453670 2.089730
P 0.897534 1.277269 -0.462779
H -2.302113 -1.386983 2.576421
C -3.878719 -0.292172 1.580264
C -2.005876 0.695628 2.927649
H -0.612119 -2.918057 1.241319
C -2.532997 -3.301447 0.369220
C -0.396925 -2.967899 -0.906560
O 0.234327 -1.464725 2.676121
O 1.537157 0.686439 2.914064
C 2.639534 -1.298468 1.670707
C -0.416173 2.397469 -1.099563
C 2.191453 2.229804 0.422693
C 1.707319 0.598711 -1.972616
H -3.970786 0.649666 1.013068
H -4.183330 -1.096316 0.897769
C -4.795191 -0.247326 2.796793
C -2.915816 0.721680 4.151480
H -0.964087 0.600941 3.239576
H -2.099060 1.652482 2.397906
H -3.081239 -3.073090 1.290521
H -3.144109 -2.927916 -0.470283
C -2.354209 -4.811930 0.243874
C -0.206233 -4.474478 -1.011976
H -0.963016 -2.616597 -1.785411
H 0.566349 -2.445075 -0.923135
C 3.862827 -0.673255 1.873633
C 2.575799 -2.594458 1.167641
H -0.957708 1.744449 -1.804176
C -1.442837 2.874219 -0.077822
C 0.120020 3.564355 -1.934033
H 2.705871 1.455373 1.003037
C 3.258453 2.902981 -0.445141
C 1.595943 3.227401 1.420405
H 2.187951 1.470217 -2.441535
C 0.730229 -0.006201 -2.979509
C 2.786578 -0.408598 -1.578570
H -5.833671 -0.110146 2.471269
H -4.754784 -1.220478 3.308457
C -4.380689 0.853186 3.762306
H -2.766245 -0.203913 4.726478
H -2.614998 1.545557 4.809762
H -1.841599 -5.182409 1.143629

H -3.337751 -5.296596 0.226448
C -1.547835 -5.192418 -0.989329
H 0.349707 -4.715576 -1.926875
H 0.411438 -4.822571 -0.169651
H 3.897081 0.309083 2.339589
C 5.028950 -1.329472 1.496457
C 3.746364 -3.236835 0.800584
H 1.616451 -3.096609 1.075969
H -1.809698 2.026716 0.509246
H -0.980638 3.568048 0.633400
C -2.597622 3.561513 -0.792538
C -1.038268 4.263972 -2.638761
H 0.632225 4.279363 -1.276364
H 0.858364 3.223039 -2.670661
H 3.705400 2.197252 -1.153337
H 2.815342 3.715268 -1.036760
C 4.355099 3.460825 0.459307
C 2.695868 3.760203 2.326428
H 1.150249 4.063931 0.863150
H 0.807410 2.752276 2.012495
H 0.152129 -0.795410 -2.479322
H 0.012368 0.748382 -3.326766
C 1.460088 -0.595211 -4.181914
C 3.505961 -0.976716 -2.792806
H 2.307201 -1.229527 -1.033590
H 3.503954 0.038270 -0.879021
H -4.542192 1.831620 3.284210
H -5.018603 0.836114 4.654120
H -1.403036 -6.278549 -1.028464
H -2.114615 -4.922296 -1.893322
H 5.989533 -0.840400 1.648914
C 4.992373 -2.610947 0.942601
H 3.698704 -4.249923 0.404105
H -3.337760 3.908460 -0.061284
H -3.114420 2.818977 -1.426758
C -2.109029 4.715734 -1.655588
H -0.658702 5.117073 -3.213631
H -1.480670 3.570431 -3.370919
H 5.116443 3.959353 -0.152889
H 4.858927 2.616722 0.955337
C 3.806646 4.410383 1.514164
H 3.089823 2.920947 2.918161
H 2.272793 4.474237 3.043128
H 0.731983 -1.053755 -4.862458
H 1.938692 0.220388 -4.744222
C 2.518270 -1.604363 -3.763897
H 4.064082 -0.176216 -3.301083
H 4.249373 -1.712288 -2.460832
C 6.250176 -3.323248 0.553507
H -2.947021 5.176314 -2.192120
H -1.693470 5.497305 -1.002352
H 3.412571 5.310517 1.018298
H 4.615480 4.751116 2.171693
H 3.038509 -1.997515 -4.645382
H 2.030510 -2.464940 -3.279337

H 6.145491 -3.815557 -0.420113
H 6.491559 -4.105894 1.284935
H 7.103068 -2.639600 0.505900

$^{Ph}Y_SPCy_2$ (A)

Au 2.274709 -0.863828 0.900496
P 1.229499 0.916127 -0.130863
C -0.389497 0.276105 -0.430932
C 2.243173 1.260115 -1.624921
C 1.167320 2.431497 0.931630
S -1.252302 0.947546 -1.784010
P -0.888090 -1.274670 0.188013
C 2.119492 0.131235 -2.641168
H 1.804139 2.170558 -2.053113
C 3.705224 1.509601 -1.252794
C 2.196075 2.433361 2.062768
H 0.163957 2.362561 1.378569
C 1.210479 3.731989 0.120474
O -0.497301 2.153579 -2.150975
C -2.855525 1.451692 -1.220280
O -1.481181 -0.078540 -2.811635
C -0.855585 -1.384167 1.996287
C -2.616389 -1.604438 -0.226801
C 0.186701 -2.599974 -0.441980
C 2.954429 0.426870 -3.879952
H 2.468095 -0.805140 -2.173575
H 1.069674 -0.013374 -2.917256
C 4.528257 1.812769 -2.499525
H 3.796571 2.328292 -0.528492
H 4.105430 0.607973 -0.759504
C 2.008182 3.649139 2.961707
H 3.209417 2.438638 1.635470
H 2.114477 1.504807 2.646556
C 1.012354 4.928421 1.043896
H 2.186996 3.820158 -0.375748
H 0.450754 3.707625 -0.667746
C -3.965161 1.111502 -1.980698
C -2.976544 2.212192 -0.062454
C -1.313144 -2.562325 2.598133
C -0.454852 -0.311521 2.791763
C -3.590518 -1.091543 0.639227
C -2.996548 -2.317781 -1.363634
C 0.367518 -2.665778 -1.825827
C 0.942775 -3.430768 0.402002
C 4.409730 0.695545 -3.525835
H 2.875012 -0.410014 -4.584855
H 2.530425 1.303262 -4.390871
H 5.576179 1.972111 -2.217482
H 4.176606 2.756613 -2.941189
C 2.038846 4.946657 2.166305
H 1.039806 3.563196 3.478902
H 2.777941 3.653505 3.743079
H 1.062987 5.853090 0.456457
H -0.001096 4.890115 1.472161

H -3.836784 0.506452 -2.874923
C -5.219197 1.535870 -1.560935
C -4.237043 2.614996 0.348353
H -2.091552 2.466527 0.518479
H -1.665257 -3.390745 1.984058
C -1.334365 -2.669942 3.982018
C -0.480452 -0.422799 4.178161
H -0.138410 0.615420 2.324868
H -3.299403 -0.528126 1.523067
C -4.934360 -1.298413 0.367947
H -2.251772 -2.712175 -2.047589
C -4.346092 -2.519835 -1.626953
H -0.174513 -1.976931 -2.474690
C 1.274738 -3.573063 -2.362201
C 1.865894 -4.319261 -0.147075
H 0.797259 -3.404464 1.480617
H 4.859588 -0.221337 -3.114431
H 4.983306 0.944295 -4.426745
H 3.042608 5.084830 1.737062
H 1.866728 5.802304 2.830217
H -6.095988 1.267674 -2.148141
C -5.378758 2.286895 -0.394437
H -4.342791 3.200535 1.260257
H -1.689845 -3.586842 4.446862
C -0.912976 -1.602660 4.772440
H -0.168612 0.418887 4.792836
C -5.312172 -2.014549 -0.764167
H -5.687844 -0.897243 1.041773
H -4.641050 -3.079214 -2.511719
H 1.405821 -3.625610 -3.440935
C 2.027514 -4.393167 -1.527299
H 2.450001 -4.961828 0.508431
C -6.733034 2.755758 0.040020
H -0.934514 -1.689332 5.856688
H -6.366714 -2.180342 -0.974899
H 2.744954 -5.091433 -1.952872
H -6.815974 2.794637 1.131520
H -7.526934 2.108573 -0.346537
H -6.927092 3.769657 -0.335610

$^{Cy}Y_{oTol}PCy_2$ (P2)

Au -1.828911 -1.916252 0.743382
P -1.260732 0.279741 -0.169826
C 0.420549 0.668073 -0.610055
C -1.800659 1.671040 0.958985
C -2.384470 0.387723 -1.645248
P 1.667151 -0.219207 0.201989
C 0.745131 2.003400 -1.207350
H -0.842993 1.877790 1.462824
C -2.226273 2.976665 0.281980
C -2.789010 1.278413 2.055903
H -2.175444 1.347182 -2.148956
C -2.139029 -0.770400 -2.612744
C -3.855950 0.344450 -1.221486

C 3.275956 0.456437 -0.404070
C 1.829547 -0.113002 2.056341
C 1.543936 -2.037658 -0.089245
C 1.072391 3.096018 -0.382553
C 0.743274 2.233077 -2.603547
H -1.529674 3.251922 -0.516539
H -3.208358 2.848911 -0.192230
C -2.322232 4.093220 1.314567
C -2.905481 2.396761 3.085010
H -3.775970 1.071105 1.618365
H -2.472057 0.341062 2.533180
H -1.093420 -0.807419 -2.920390
H -2.336432 -1.709202 -2.070098
C -3.043679 -0.684243 -3.834182
C -4.786073 0.435745 -2.425367
H -4.027390 -0.604798 -0.688063
H -4.095665 1.141778 -0.513342
H 3.094752 1.540766 -0.351628
C 3.527369 0.119352 -1.873688
C 4.530054 0.172242 0.425592
H 2.745514 -0.682827 2.277946
C 2.027717 1.321885 2.542377
C 0.684721 -0.751643 2.839480
H 0.717865 -2.333416 0.579546
C 2.777972 -2.842177 0.317813
C 1.083581 -2.404859 -1.496513
H 1.075492 2.942449 0.691923
C 1.371224 4.356186 -0.877847
C 1.038729 3.511893 -3.090600
C 0.466754 1.149417 -3.600651
H -1.319233 4.279133 1.730782
H -2.635777 5.029017 0.833360
C -3.281577 3.724310 2.438991
H -1.935786 2.507110 3.596616
H -3.635126 2.126592 3.859645
H -2.808418 0.234961 -4.394837
H -2.837501 -1.522595 -4.512571
C -4.511187 -0.669466 -3.433757
H -5.831147 0.399253 -2.090825
H -4.648376 1.414618 -2.910703
H 3.766934 -0.950534 -1.961929
H 2.622122 0.292618 -2.461369
C 4.687970 0.940218 -2.417325
C 5.704261 0.981304 -0.119101
H 4.380841 0.424389 1.480699
H 4.775115 -0.897302 0.388995
H 2.808126 1.842105 1.972893
H 1.093000 1.871157 2.363969
C 2.332677 1.362453 4.034322
C 0.960275 -0.715961 4.337994
H 0.501383 -1.784866 2.523684
H -0.244967 -0.204520 2.624516
H 3.611207 -2.603181 -0.359181
H 3.109437 -2.583308 1.331646
C 2.485395 -4.336137 0.229400

C 0.807686 -3.899709 -1.586177
H 1.842218 -2.113031 -2.237230
H 0.176195 -1.842132 -1.729431
H 1.614529 5.166400 -0.192722
C 1.347154 4.571188 -2.250971
H 1.038181 3.668392 -4.169207
H 1.087241 1.279141 -4.495084
H -0.579779 1.162542 -3.932320
H 0.659730 0.163527 -3.168108
H -4.299352 3.645174 2.026617
H -3.315281 4.521183 3.193636
H -4.766934 -1.639339 -2.981234
H -5.154150 -0.555935 -4.316446
H 4.863594 0.690144 -3.471511
H 4.410262 2.005083 -2.389768
C 5.949611 0.712455 -1.596995
H 5.490217 2.051649 0.023005
H 6.607180 0.764448 0.466190
H 3.284437 0.842506 4.224310
H 2.475030 2.401314 4.359076
C 1.220156 0.698849 4.832805
H 0.116183 -1.164508 4.876899
H 1.838303 -1.343776 4.555545
H 3.379093 -4.908529 0.510337
H 1.704134 -4.590276 0.961241
C 2.014598 -4.725325 -1.164770
H -0.045452 -4.130009 -0.929109
H 0.497365 -4.161484 -2.606089
H 1.574748 5.551642 -2.665087
H 6.770283 1.340468 -1.966819
H 6.275933 -0.331410 -1.723336
H 0.299482 1.292695 4.723560
H 1.462215 0.695440 5.903350
H 1.779525 -5.796781 -1.201593
H 2.836076 -4.561783 -1.880177

$^{Cy}Y_{Mes}PCy_2$ (P3)

Au 1.428000 -2.098884 -1.029927
P 1.376073 -0.053473 0.041824
C -0.208698 0.683928 0.134697
C 2.236236 -0.460082 1.636923
C 2.493476 1.119310 -0.855757
P -1.740936 -0.144582 0.010155
C -0.150599 2.171732 0.383165
H 2.276528 0.517042 2.148134
C 3.661249 -0.974595 1.438443
C 1.461139 -1.452683 2.492261
H 1.769032 1.632424 -1.493944
C 3.145496 2.180798 0.041380
C 3.513653 0.474819 -1.800492
C -3.172798 1.004744 -0.060799
C -2.149313 -1.252849 1.464126
C -1.839875 -1.261655 -1.461299
C -0.086253 3.112139 -0.671412
C -0.069913 2.662157 1.714241

H 4.256165 -0.287541 0.830961
H 3.614321 -1.925803 0.881699
C 4.350315 -1.198261 2.781681
C 2.125063 -1.654984 3.847153
H 0.419094 -1.138748 2.610575
H 1.436333 -2.416218 1.957830
H 2.401119 2.652316 0.692564
H 3.893732 1.712910 0.695170
C 3.829739 3.238141 -0.817171
C 4.211443 1.547026 -2.630583
H 4.259157 -0.099160 -1.233935
H 3.008341 -0.241113 -2.463082
H -3.994718 0.269540 -0.061004
C -3.333695 1.848136 -1.323024
C -3.355685 1.896051 1.172057
H -1.636121 -0.722027 2.280979
C -1.525950 -2.643250 1.327791
C -3.616850 -1.410945 1.885301
H -1.238900 -2.128260 -1.142638
C -3.251186 -1.784356 -1.738705
C -1.192290 -0.744416 -2.743742
C 0.087986 4.468604 -0.384064
C -0.183481 2.749162 -2.126653
C 0.108037 4.021199 1.949391
C -0.236474 1.770839 2.908134
H 5.361819 -1.587574 2.613338
H 4.472047 -0.226624 3.283291
C 3.558539 -2.136441 3.679822
H 1.542947 -2.370148 4.441982
H 2.112783 -0.704185 4.400964
H 3.063772 3.772849 -1.400085
H 4.301691 3.986556 -0.168760
C 4.850954 2.616453 -1.756964
H 3.471353 2.018147 -3.295772
H 4.959879 1.079540 -3.282088
H -2.584398 2.645427 -1.311558
H -3.165907 1.255602 -2.228377
C -4.726673 2.469217 -1.352163
C -4.751861 2.507151 1.162980
H -3.188730 1.340219 2.101226
H -2.609653 2.698565 1.144985
H -2.086624 -3.205679 0.566047
H -0.491908 -2.577633 0.963934
C -1.588086 -3.403949 2.645387
C -3.697204 -2.163659 3.210564
H -4.121994 -0.447102 1.989193
H -4.170121 -1.973351 1.120866
H -3.717956 -2.161713 -0.822010
H -3.883130 -0.959124 -2.097967
C -3.207472 -2.887363 -2.790950
C -1.135667 -1.860512 -3.778779
H -0.185453 -0.364602 -2.533438
H -1.767981 0.097123 -3.149960
H 0.141032 5.172422 -1.214778
C 0.203420 4.949937 0.912967

H -0.963836 3.338718 -2.622011
H -0.408799 1.692145 -2.267236
H 0.750598 2.978141 -2.656879
H 0.172131 4.369158 2.980192
H 0.186580 2.231708 3.806197
H 0.222405 0.793467 2.767294
H -1.300874 1.591392 3.111464
H 4.048690 -2.235013 4.655700
H 3.549142 -3.142439 3.233765
H 5.317834 3.387378 -2.381808
H 5.661325 2.165354 -1.164448
H -4.840194 3.078152 -2.257403
H -5.477132 1.666760 -1.421608
C -4.989821 3.307787 -0.109453
H -5.501166 1.703802 1.241995
H -4.883161 3.142627 2.047191
H -1.134709 -4.395948 2.525526
H -0.985333 -2.873061 3.397789
C -3.024186 -3.525568 3.134768
H -3.213564 -1.557925 3.991919
H -4.747376 -2.270283 3.508337
H -4.225099 -3.235060 -3.005969
H -2.665797 -3.751757 -2.376564
C -2.519941 -2.423315 -4.066701
H -0.664770 -1.495872 -4.699871
H -0.483875 -2.665756 -3.395636
C 0.418880 6.403993 1.198105
H -6.011308 3.706383 -0.126638
H -4.315510 4.177219 -0.114361
H -3.055390 -4.022982 4.111528
H -3.586738 -4.168547 2.441426
H -2.457801 -3.247323 -4.787523
H -3.130049 -1.641361 -4.543198
H 1.394901 6.569863 1.671875
H -0.340386 6.788913 1.889610
H 0.382718 7.004045 0.283775

$^{Cy}Y_{SF}PCy_2$ (P5)
Au -3.109711 -0.772264 1.003108
P -1.193100 -1.317953 -0.159117
C -0.212660 0.146189 -0.428593
C -0.268527 -2.568749 0.854125
C -1.832029 -2.134994 -1.679990
S 1.164825 -0.151289 -1.384826
P -0.751736 1.765617 0.083908
H 0.611826 -2.001434 1.187776
C -1.029801 -3.018527 2.106001
C 0.210474 -3.777355 0.039251
H -0.964838 -2.693317 -2.055710
C -2.969389 -3.106759 -1.363507
C -2.259078 -1.135999 -2.744606
O 0.941057 -1.380774 -2.145170
O 1.653507 1.057311 -2.037665
C 2.598361 -0.689964 -0.244922
C -1.158516 1.735392 1.882354

C -2.274790 2.095738 -0.894770
C 0.474487 3.069094 -0.303358
H -1.330693 -2.141988 2.698410
H -1.957091 -3.528302 1.799139
C -0.175865 -3.952036 2.954239
C 1.075891 -4.682126 0.908291
H -0.660126 -4.347428 -0.311608
H 0.761216 -3.445663 -0.845782
H -2.692443 -3.810818 -0.568407
H -3.830596 -2.530538 -0.984060
C -3.387306 -3.851205 -2.626617
C -2.694032 -1.865629 -4.009745
H -3.103095 -0.543579 -2.356052
H -1.435341 -0.447239 -2.960535
F 2.597587 -2.021456 -0.174148
F 2.391863 -0.205742 0.985607
C 3.967380 -0.233323 -0.788141
H -2.170713 1.291315 1.902338
C -0.240083 0.815773 2.686563
C -1.277037 3.102551 2.560176
H -2.729835 1.092225 -0.930982
C -3.327089 3.028829 -0.298296
C -1.904180 2.463515 -2.335896
H 0.702742 2.854754 -1.355071
C 1.775835 2.945480 0.486218
C -0.043325 4.511933 -0.230934
H 0.681884 -3.385295 3.346634
H -0.753250 -4.285589 3.825095
C 0.329825 -5.141205 2.151219
H 1.407301 -5.542163 0.314159
H 1.985080 -4.137029 1.199806
H -2.548072 -4.472300 -2.971913
H -4.209106 -4.538988 -2.393800
C -3.794385 -2.878900 -3.724871
H -3.024520 -1.136598 -4.759798
H -1.820107 -2.376999 -4.437940
F 4.104649 1.078561 -0.564399
F 4.010531 -0.479182 -2.094142
C 5.146883 -0.983394 -0.115854
H -0.144531 -0.159223 2.197304
H 0.772855 1.241332 2.709231
C -0.775697 0.647683 4.101387
C -1.823954 2.930148 3.973146
H -0.282605 3.564801 2.612605
H -1.913585 3.783202 1.984925
H -3.613697 2.690484 0.707108
H -2.927275 4.045121 -0.186392
C -4.555304 3.066606 -1.202551
C -3.148688 2.518644 -3.212607
H -1.417208 3.446698 -2.354655
H -1.168097 1.749367 -2.728766
H 1.596923 3.230089 1.533493
H 2.133885 1.917704 0.494895
C 2.828376 3.860160 -0.124117
C 1.004692 5.435114 -0.849009

H -0.995686 4.632372 -0.755384
H -0.210934 4.808478 0.810190
H 0.974596 -5.771446 2.774989
H -0.523565 -5.769016 1.853549
H -4.706330 -2.346636 -3.413107
H -4.054255 -3.425296 -4.639140
F 5.264353 -2.181781 -0.680765
F 4.889357 -1.133764 1.189332
C 6.490974 -0.231892 -0.263782
H -1.745512 0.123264 4.049399
H -0.105358 -0.001367 4.677864
C -0.958764 1.988393 4.797828
H -1.898591 3.909914 4.459599
H -2.850476 2.535327 3.915555
H -5.026066 2.070775 -1.208233
H -5.297544 3.758097 -0.786228
C -4.192768 3.459286 -2.627760
H -2.868953 2.831077 -4.225575
H -3.577274 1.510405 -3.306505
H 3.766648 3.769064 0.436211
H 3.037365 3.504295 -1.143332
C 2.352608 5.304899 -0.153250
H 1.114042 5.185480 -1.914737
H 0.647053 6.471039 -0.805506
F 7.482310 -1.060619 -0.002870
F 6.611267 0.222643 -1.501256
F 6.536056 0.782523 0.582039
H -1.392116 1.846094 5.794796
H 0.028332 2.448422 4.952446
H -5.090480 3.471424 -3.257006
H -3.799779 4.487133 -2.630943
H 2.261665 5.677102 0.878538
H 3.095423 5.942779 -0.646678

4.6.3 Energies and coordinates of different conformers

	E(SCF)	Corr(H)	Corr(G)	ΔG [kJ/mol]
**LAuCl complexes**				
**P1C1AuCl**	-3316.976272	1.002261	0.874006	–
**P1C2AuCl**	-3316.948685	1.002867	0.873975	72.3479
**P1C3AuCl**	-3316.959054	1.002696	0.874914	47.5899
**P3C1AuCl**	-2846.519865	1.046765	0.919248	0.0374
**P3C2AuCl**	-2846.494679	1.047168	0.918923	65.2733
**P5C1AuCl**	-4098.137436	0.942299	0.803451	0.0374
**P5C2AuCl**	-4098.094083	0.94318	0.804587	116.8057
**P5C3AuCl**	-4098.103397	0.94237	0.801452	84.1203
**LAu^+ complexes**				
**$P1C1Au^+$**	-2856.12562787	0.999021	0.874168	–
**$P1C2Au^+$**	-2856.11238752	1.000346	0.876022	39.6302
**$P1C3Au^+$**	-2856.12343492	0.999257	0.873303	3.4865
**$P3C1Au^+$**	-2385.676675	1.04396	0.920194	–
**$P3C2Au^+$**	-2385.663721	1.044548	0.921984	38.7115
**$P5C1Au^+$**	-3637.273559	0.939227	0.80444	–
**$P5C2Au^+$**	-3637.252123	0.940599	0.806427	61.5353
**$P5C3Au^+$**	-3637.242518	0.939482	0.804945	82.8613

P1-AuCl C1
Au -2.422917 -0.216145 -1.011903
Cl -3.632837 0.527609 -2.867380
P -1.099759 -0.863669 0.740814
C 0.474936 -0.009895 0.773411
C -1.984844 -0.719522 2.375438
C -0.832471 -2.684324 0.513335
S 1.526791 -0.508378 2.076176
P 0.892400 1.297659 -0.335651
H -1.726030 -1.656352 2.890737
C -3.498235 -0.652207 2.155256
C -1.546200 0.425817 3.286073
H -0.155236 -2.987688 1.321707
C -2.130428 -3.480069 0.645836
C -0.190774 -2.957078 -0.843469
O 0.812434 -1.589345 2.772320
O 2.020101 0.618351 2.879980
C 3.003539 -1.265254 1.411135
C -0.516472 2.399619 -0.774215
C 2.252744 2.278200 0.417693
C 1.547989 0.739153 -1.970574
H -3.732229 0.296651 1.646119
H -3.836050 -1.442327 1.473314
C -4.247180 -0.725760 3.479131
C -2.280992 0.343939 4.619168
H -0.467949 0.408187 3.455989
H -1.778744 1.389058 2.813178
H -2.578886 -3.329347 1.634804
H -2.857066 -3.110237 -0.096119
C -1.878448 -4.968754 0.427575
C 0.080551 -4.440427 -1.046573
H -0.877500 -2.607503 -1.630681
H 0.729651 -2.371056 -0.950984

C 4.218462 -0.596400 1.461311
C 2.921620 -2.539992 0.860438
H -1.115542 1.758932 -1.438953
C -1.444871 2.799745 0.368512
C -0.109335 3.614703 -1.611596
H 2.868512 1.509671 0.896490
C 3.177260 3.042790 -0.532076
C 1.739859 3.199751 1.527379
H 1.924610 1.659668 -2.441510
C 0.491048 0.134486 -2.892908
C 2.716955 -0.221134 -1.764999
H -5.328316 -0.659536 3.301332
H -4.065360 -1.709338 3.939211
C -3.791590 0.369134 4.433304
H -1.988144 -0.586323 5.128816
H -1.959430 1.167593 5.269551
H -1.237566 -5.342896 1.240354
H -2.825450 -5.518749 0.500159
C -1.203627 -5.245062 -0.908575
H 0.539029 -4.606633 -2.030530
H 0.810400 -4.787459 -0.297811
H 4.280537 0.362982 1.970333
C 5.338899 -1.174374 0.873207
C 4.044958 -3.105950 0.280538
H 1.980243 -3.081454 0.893522
H -1.735890 1.907661 0.930841
H -0.931035 3.466949 1.071054
C -2.685100 3.484062 -0.192842
C -1.361264 4.286039 -2.168042
H 0.442650 4.332109 -0.987910
H 0.556216 3.326734 -2.435238
H 3.566859 2.393157 -1.323019
H 2.632288 3.857529 -1.026907
C 4.348465 3.613641 0.264256
C 2.912886 3.746665 2.325696
H 1.191558 4.035580 1.070048
H 1.049876 2.656483 2.180394
H 0.015945 -0.713635 -2.384348
H -0.312775 0.848848 -3.105380
C 1.105201 -0.341700 -4.203514
C 3.316264 -0.685828 -3.083843
H 2.348888 -1.094346 -1.215677
H 3.489958 0.229082 -1.130092
H -4.086876 1.346923 4.022097
H -4.301375 0.272425 5.400758
H -1.002499 -6.318146 -1.021242
H -1.888050 -4.967136 -1.724240
H 6.291175 -0.646804 0.903867
C 5.267492 -2.422966 0.253895
H 3.976448 -4.100742 -0.157908
H -3.344919 3.785221 0.630824
H -3.244424 2.756748 -0.800426
C -2.319281 4.684254 -1.054161
H -1.076296 5.161029 -2.766205
H -1.868382 3.584499 -2.846754

H 5.011415 4.176635 -0.405186
H 4.943043 2.774245 0.657645
C 3.888642 4.487450 1.422789
H 3.412292 2.902340 2.822784
H 2.548098 4.407048 3.122351
H 0.323682 -0.800240 -4.821550
H 1.476595 0.528777 -4.766270
C 2.252954 -1.312982 -3.972204
H 3.770900 0.170520 -3.605558
H 4.129077 -1.396705 -2.882999
C 6.461763 -3.024984 -0.421580
H -3.223624 5.137781 -1.478540
H -1.845772 5.456309 -0.427136
H 3.396454 5.386113 1.019862
H 4.755188 4.840941 1.996159
H 2.686211 -1.631950 -4.928698
H 1.866056 -2.221605 -3.484653
H 6.358809 -2.969628 -1.514019
H 6.576102 -4.084042 -0.162959
H 7.384480 -2.503054 -0.148911

P1AuCl C2
P 0.752820 -1.056351 0.169665
C 1.013873 2.619422 -1.028581
C 0.745999 3.860640 -1.595024
C -0.429533 0.174810 -0.403598
C 1.127098 -1.196626 2.001658
C 0.124873 -2.740205 -0.278414
S 0.140105 1.198862 -1.698327
C 1.956693 2.502635 -0.015990
H 0.003025 3.935364 -2.384862
C 1.422248 4.983431 -1.132332
P -2.110823 0.439562 0.053510
H 1.869259 -2.006601 1.912579
C 0.062639 -1.689157 2.976347
C 1.899569 -0.005317 2.565382
H -0.816260 -2.914656 0.258280
C 1.095091 -3.850522 0.126895
C -0.141457 -2.774794 -1.783228
O -1.041109 1.810874 -2.338793
O 1.062970 0.469887 -2.567356
C 2.621325 3.630813 0.440063
H 2.170196 1.531340 0.417258
H 1.208552 5.954622 -1.576617
C 2.369635 4.891124 -0.111243
C -2.631795 -1.004555 1.078437
C -3.333977 0.670865 -1.306001
C -2.308246 1.963010 1.094408
H -0.445759 -2.577243 2.574794
H -0.703842 -0.915426 3.120638
C 0.667300 -2.009097 4.338599
C 2.461109 -0.310787 3.949438
H 1.251427 0.878715 2.613559
H 2.728418 0.233722 1.887771

H 2.081881 -3.639309 -0.314022
H 1.234079 -3.870071 1.215325
C 0.594589 -5.214052 -0.337515
C -0.646220 -4.138944 -2.228800
H -0.850552 -1.985269 -2.058367
H 0.793980 -2.526329 -2.307859
H 3.358459 3.526592 1.235030
C 3.114193 6.098483 0.371463
H -1.684317 -1.369574 1.479659
C -3.529994 -0.673268 2.273098
C -3.230632 -2.149723 0.255753
H -3.028278 1.646909 -1.694728
C -4.776748 0.813357 -0.814356
C -3.220755 -0.282770 -2.494003
H -3.253650 1.837850 1.642406
C -1.151898 2.028574 2.088546
C -2.404277 3.248902 0.267034
H 1.379203 -2.841361 4.232153
H -0.119158 -2.355387 5.022152
C 1.387314 -0.797604 4.910034
H 3.235510 -1.086216 3.852762
H 2.963806 0.579525 4.348153
H -0.336086 -5.457925 0.199749
H 1.323264 -5.986782 -0.061264
C 0.326671 -5.240049 -1.834531
H -0.812756 -4.137624 -3.313436
H -1.626184 -4.336381 -1.762638
H 3.125215 6.150247 1.466694
H 2.671629 7.023691 -0.011336
H 4.160740 6.067043 0.040539
H -4.486460 -0.264229 1.915489
H -3.071164 0.099350 2.900509
C -3.783280 -1.915273 3.122697
C -3.427454 -3.383311 1.126489
H -4.204625 -1.849509 -0.147427
H -2.596282 -2.379141 -0.605846
H -5.170211 -0.147677 -0.457769
H -4.840611 1.511068 0.031570
C -5.646375 1.313309 -1.964697
C -4.089902 0.238505 -3.631086
H -2.175000 -0.341634 -2.810847
H -3.550986 -1.293031 -2.225445
H -0.218232 2.027470 1.512905
H -1.128261 1.123325 2.709825
C -1.191141 3.275989 2.956692
C -2.413358 4.488635 1.152184
H -3.309247 3.236002 -0.351690
H -1.557393 3.299534 -0.425642
H 0.656368 0.008480 5.082024
H 1.824056 -1.035056 5.888372
H -0.056181 -6.222995 -2.137880
H 1.273839 -5.091279 -2.373770
H -2.834887 -2.224372 3.587372
H -4.462412 -1.662568 3.946572
C -4.335533 -3.073169 2.306314

H -2.449871 -3.731066 1.497648
H -3.837099 -4.202116 0.521628
H -5.323226 2.330180 -2.234156
H -6.690557 1.393536 -1.635961
C -5.536755 0.410993 -3.187026
H -4.030713 -0.442265 -4.489444
H -3.684042 1.203710 -3.967205
H -2.082453 3.258362 3.602695
H -0.319773 3.281326 3.624184
C -1.214288 4.522045 2.086390
H -2.435448 5.383349 0.517355
H -3.338356 4.506556 1.749585
H -5.336606 -2.811756 1.930850
H -4.462970 -3.959253 2.940484
H -6.145653 0.812560 -4.006993
H -5.959372 -0.576244 -2.942784
H -0.290760 4.558546 1.487994
H -1.231225 5.428531 2.704449
Au 2.859148 -0.846891 -0.723493
Cl 5.085010 -0.798633 -1.364193

P1AuCl C3
P -0.615785 -0.984291 0.150958
C -1.667554 2.139377 -0.989337
C -2.645045 2.313704 -1.963720
C 0.495019 0.346827 -0.291141
C -0.302562 -2.636270 -0.633807
C -0.546156 -1.286698 1.976024
S -0.121467 1.426834 -1.508826
C -1.890368 2.539069 0.322669
H -2.450491 1.979714 -2.979175
C -3.869020 2.854036 -1.601986
P 2.227467 0.591472 -0.061949
H -1.185866 -3.188119 -0.273249
C 0.930135 -3.427629 -0.209694
C -0.435912 -2.540878 -2.152608
H 0.476320 -1.571657 2.248957
C -1.464396 -2.440294 2.385164
C -0.923047 -0.010779 2.728719
O -0.414883 0.724329 -2.766095
O 0.863285 2.530691 -1.571631
C -3.127657 3.060658 0.670217
H -1.125029 2.385018 1.076838
H -4.648308 2.956616 -2.354652
C -4.143492 3.203925 -0.278449
C 2.768551 -0.744442 1.109114
C 2.737946 2.240208 0.584396
C 3.285438 0.392228 -1.580008
H 0.974466 -3.519143 0.885728
H 1.840836 -2.898347 -0.519263
C 0.942681 -4.805957 -0.861353
C -0.385080 -3.919598 -2.796649
H 0.364239 -1.908604 -2.551438
H -1.370659 -2.024459 -2.405838
H -2.488807 -2.214781 2.049899

H -1.166832 -3.369940 1.884795
C -1.443155 -2.645978 3.895232
C -0.897589 -0.224884 4.235475
H -0.252470 0.807996 2.437719
H -1.932952 0.292082 2.410233
H -3.320629 3.333924 1.706456
C -5.518356 3.633316 0.123482
H 2.019261 -1.523967 0.941740
C 4.129125 -1.381539 0.815328
C 2.668523 -0.339388 2.583600
H 2.576905 2.857797 -0.306228
C 4.222750 2.333856 0.943338
C 1.835873 2.834696 1.658602
H 4.258598 0.098207 -1.159802
C 2.756842 -0.747975 -2.443572
C 3.542967 1.626720 -2.448733
H 0.085118 -5.388447 -0.492057
H 1.845513 -5.354020 -0.560927
C 0.858449 -4.691002 -2.377141
H -1.278701 -4.490355 -2.501584
H -0.427479 -3.820107 -3.888547
H -0.434695 -2.967115 4.201985
H -2.124498 -3.462386 4.166034
C -1.810031 -1.372524 4.642171
H -1.185716 0.701107 4.749574
H 0.132999 -0.451476 4.554789
H -5.513658 4.193346 1.064579
H -6.140318 2.738271 0.263450
H -5.993739 4.248366 -0.648539
H 4.926407 -0.631185 0.928101
H 4.172916 -1.738746 -0.219812
C 4.388313 -2.556704 1.751972
C 2.924867 -1.529785 3.498809
H 3.406744 0.438676 2.808303
H 1.688589 0.096777 2.793891
H 4.450996 1.743585 1.839834
H 4.847674 1.928584 0.136763
C 4.590765 3.791056 1.205405
C 2.227766 4.284172 1.916387
H 0.799821 2.774916 1.311478
H 1.905315 2.266188 2.594916
H 1.789229 -0.430619 -2.854342
H 2.553670 -1.636681 -1.834276
C 3.719585 -1.091362 -3.570590
C 4.532088 1.279532 -3.556231
H 3.937517 2.456909 -1.851402
H 2.599561 1.975736 -2.878924
H 1.753655 -4.166432 -2.748716
H 0.869296 -5.687538 -2.836906
H -1.763894 -1.536338 5.726419
H -2.851052 -1.104492 4.408346
H 3.653241 -3.347503 1.535200
H 5.375491 -2.984876 1.537781
C 4.278724 -2.160252 3.215325
H 2.135324 -2.283988 3.352143

H 2.853124 -1.210327 4.546361
H 4.477914 4.359591 0.270061
H 5.648105 3.863712 1.490513
C 3.702050 4.406146 2.278351
H 1.599643 4.706926 2.710679
H 2.023841 4.871506 1.009225
H 4.655300 -1.485995 -3.143532
H 3.294116 -1.894774 -4.186000
C 4.026560 0.132825 -4.419698
H 4.716586 2.169358 -4.171349
H 5.501424 1.001855 -3.111967
H 5.073094 -1.438000 3.457826
H 4.442052 -3.031478 3.861753
H 3.971541 5.458089 2.436226
H 3.884521 3.892401 3.235232
H 3.108074 0.450558 -4.934155
H 4.757008 -0.113669 -5.200886
Au -2.826195 -0.547407 -0.287373
Cl -5.136083 -0.297343 -0.470606

P3AuCl C1
Au 2.359683 -0.807570 -0.540651
Cl 3.702095 -2.613097 -1.165581
P 0.971777 0.898047 0.096574
C -0.770755 0.532681 0.093299
C 1.564802 1.464025 1.763340
C 1.186237 2.389138 -0.996856
P -1.368783 -1.090601 0.003326
C -1.681759 1.729975 0.163254
H 0.927275 2.317227 2.044649
C 3.024099 1.920066 1.738714
C 1.421427 0.350494 2.794330
H 0.395495 2.216625 -1.734167
C 0.912464 3.744630 -0.336767
C 2.498822 2.419259 -1.782613
C -3.197261 -1.155380 -0.185319
C -1.110718 -2.140382 1.548452
C -0.625125 -2.118202 -1.349060
C -2.093796 2.421743 -1.003776
C -2.058907 2.290180 1.411581
H 3.182667 2.718151 1.007088
H 3.646630 1.072028 1.411021
C 3.473701 2.391873 3.117631
C 1.832143 0.816990 4.182971
H 0.399949 -0.042074 2.794485
H 2.076442 -0.480276 2.488284
H -0.025226 3.732038 0.229893
H 1.711091 3.973015 0.382790
C 0.873396 4.845936 -1.389322
C 2.468104 3.533466 -2.822740
H 3.349160 2.570840 -1.102763
H 2.668479 1.449192 -2.267071
H -3.365410 -2.244187 -0.160812
C -3.774494 -0.648234 -1.502873
C -3.977650 -0.540740 0.980567

H -1.172445 -1.372155 2.333599
C 0.289910 -2.747274 1.611151
C -2.133395 -3.222639 1.919674
H 0.350817 -2.402602 -0.927818
C -1.381095 -3.417029 -1.629822
C -0.319927 -1.377965 -2.648994
C -2.768178 3.640761 -0.898705
C -1.846139 1.924957 -2.400561
C -2.728441 3.510146 1.468633
C -1.800465 1.593862 2.715292
H 4.527366 2.697776 3.081604
H 2.898353 3.289653 3.392063
C 3.269123 1.319901 4.178812
H 1.712240 -0.000561 4.906522
H 1.159011 1.625383 4.510346
H 0.020407 4.663926 -2.062058
H 0.685360 5.815773 -0.910185
C 2.163593 4.887378 -2.196066
H 1.692010 3.299801 -3.568580
H 3.420864 3.565787 -3.366854
H -3.700712 0.443445 -1.521432
H -3.207551 -1.027108 -2.360304
C -5.240726 -1.050724 -1.614515
C -5.444365 -0.945063 0.893033
H -3.555762 -0.831869 1.949376
H -3.898530 0.551021 0.923139
H 0.354250 -3.579481 0.894050
H 1.048167 -2.021222 1.295324
C 0.601762 -3.268833 3.005473
C -1.842363 -3.750771 3.322875
H -3.162317 -2.852466 1.884059
H -2.075656 -4.057571 1.208458
H -1.595170 -3.954484 -0.699249
H -2.353027 -3.188926 -2.094021
C -0.566731 -4.305263 -2.564905
C 0.497827 -2.268469 -3.575788
H 0.230109 -0.454727 -2.429602
H -1.253945 -1.085958 -3.149251
H -3.057518 4.153937 -1.816654
C -3.074190 4.223202 0.323682
H -2.789732 1.837691 -2.953489
H -1.355405 0.952542 -2.397377
H -1.221345 2.627373 -2.967307
H -2.996724 3.912853 2.445997
H -0.871193 1.930408 3.190831
H -1.718757 0.514718 2.579000
H -2.613026 1.795013 3.422593
H 3.549672 1.701146 5.169232
H 3.940300 0.473823 3.967577
H 2.109974 5.664383 -2.969878
H 2.993147 5.169982 -1.529494
H -5.657726 -0.684416 -2.561342
H -5.312163 -2.149072 -1.646941
C -6.051093 -0.522545 -0.438337
H -5.526587 -2.037961 1.005543

H -6.004946 -0.503172 1.726643
H 1.611849 -3.696744 3.023226
H 0.606050 -2.424101 3.712104
C -0.427989 -4.298996 3.445120
H -1.979606 -2.930387 4.044301
H -2.577976 -4.521594 3.587179
H -1.119626 -5.230186 -2.774706
H 0.361785 -4.598973 -2.053453
C -0.217656 -3.582015 -3.857601
H 0.707676 -1.735535 -4.512139
H 1.470212 -2.470020 -3.102060
C -3.745793 5.560157 0.411608
H -7.093875 -0.857507 -0.509110
H -6.071612 0.576748 -0.485280
H -0.233355 -4.628350 4.473852
H -0.333515 -5.192391 2.809410
H 0.401717 -4.225118 -4.495376
H -1.143596 -3.382040 -4.420093
H -3.010769 6.355881 0.595523
H -4.468863 5.593900 1.234758
H -4.270735 5.808400 -0.517065

P3 AuCl C2
Au -2.871186 0.740406 -0.348788
Cl -4.643667 2.197497 -0.713799
P -1.123646 -0.692913 0.052186
C 0.337754 0.317216 0.209892
C -1.639874 -1.728778 1.517936
C -0.913649 -1.971339 -1.315839
P 2.014921 -0.094707 0.127767
C 0.049175 1.786214 0.015571
H -0.895937 -2.527928 1.665504
C -3.009482 -2.361828 1.237207
C -1.765441 -0.915965 2.802539
H -0.039189 -1.590636 -1.864535
C -0.596544 -3.402710 -0.871783
C -2.087825 -1.944697 -2.298122
C 3.150635 1.316690 0.497279
C 2.683360 -0.676169 -1.556310
C 2.480258 -1.487904 1.248913
C -0.101489 2.644519 1.126762
C -0.085318 2.347376 -1.275410
H -3.026887 -2.911235 0.291237
H -3.737603 -1.544792 1.118380
C -3.440652 -3.271514 2.380905
C -2.183476 -1.795838 3.973967
H -0.835342 -0.394875 3.035558
H -2.520266 -0.131736 2.634039
H 0.277684 -3.421804 -0.205712
H -1.423883 -3.803933 -0.276676
C -0.378088 -4.328090 -2.065367
C -1.852980 -2.880412 -3.475527
H -3.014194 -2.224322 -1.775320
H -2.249423 -0.916686 -2.646474
H 4.115561 0.832913 0.273384

C 3.247079 1.807601 1.940794
C 3.029100 2.538203 -0.424304
H 1.939685 -0.228310 -2.229940
C 2.626395 -2.192546 -1.760837
C 4.065136 -0.189430 -2.015156
H 2.072832 -2.367520 0.725827
C 3.988853 -1.694391 1.391763
C 1.817573 -1.458543 2.620742
C -0.277274 4.014395 0.932563
C -0.152821 2.128476 2.531762
C -0.282521 3.717835 -1.425568
C -0.024938 1.525181 -2.529783
H -4.417648 -3.717032 2.153059
H -2.729041 -4.107668 2.468949
C -3.498009 -2.510178 3.696990
H -2.260621 -1.190332 4.886450
H -1.393179 -2.540842 4.160767
H 0.519026 -4.013439 -2.617572
H -0.184228 -5.349367 -1.711979
C -1.568509 -4.300999 -3.012151
H -0.993041 -2.513689 -4.058956
H -2.718548 -2.856813 -4.149589
H 2.356578 2.396584 2.182354
H 3.286417 0.975830 2.651278
C 4.491726 2.678363 2.095869
C 4.293714 3.380073 -0.317312
H 2.818487 2.255895 -1.462134
H 2.178372 3.147057 -0.102328
H 3.412368 -2.671136 -1.159028
H 1.680294 -2.605483 -1.409915
C 2.834410 -2.557917 -3.225135
C 4.291756 -0.534823 -3.484209
H 4.189900 0.887134 -1.884437
H 4.847300 -0.669737 -1.408030
H 4.487370 -1.696505 0.415597
H 4.414787 -0.851618 1.955569
C 4.274480 -2.993664 2.137506
C 2.088166 -2.759264 3.364834
H 0.747962 -1.286533 2.506707
H 2.202526 -0.615347 3.209802
H -0.374016 4.658620 1.806892
C -0.362309 4.578801 -0.336079
H 0.132926 2.905142 3.250320
H 0.484931 1.252308 2.673927
H -1.180435 1.823050 2.769236
H -0.385057 4.125006 -2.431663
H -0.718150 1.924586 -3.277993
H -0.289501 0.480557 -2.349687
H 0.978739 1.544947 -2.980981
H -3.753755 -3.185064 4.524196
H -4.304040 -1.763670 3.640518
H -1.387517 -4.960267 -3.870835
H -2.455172 -4.698447 -2.494852
H 4.561802 3.044826 3.128108
H 5.384839 2.056445 1.925810

C 4.495153 3.846408 1.118756
H 5.165240 2.790666 -0.645837
H 4.225924 4.243003 -0.991673
H 2.784306 -3.647576 -3.348354
H 2.005124 -2.139541 -3.817248
C 4.160611 -2.026531 -3.744964
H 3.552213 0.009323 -4.091308
H 5.278691 -0.173799 -3.801022
H 5.357244 -3.128298 2.255725
H 3.920124 -3.838459 1.526862
C 3.582423 -3.023445 3.493984
H 1.614355 -2.731647 4.354367
H 1.614178 -3.589111 2.816735
C -0.604827 6.045908 -0.521762
H 5.426071 4.419977 1.214129
H 3.675901 4.532240 1.381068
H 4.270989 -2.242559 -4.815135
H 4.980857 -2.554125 -3.234528
H 3.763130 -3.984155 3.992526
H 4.027166 -2.250530 4.139294
H -1.679805 6.247177 -0.625331
H -0.114988 6.424770 -1.425940
H -0.243897 6.625600 0.334950

P5 AuCl C1
Au -3.025194 -0.702524 0.760643
Cl -4.966891 0.156842 1.720793
P -1.052100 -1.348126 -0.216006
C -0.001992 0.090991 -0.518792
C -0.104523 -2.560034 0.838681
C -1.483731 -2.226070 -1.782679
S 1.421515 -0.174594 -1.382862
P -0.588060 1.708292 -0.049799
H 0.732174 -1.962557 1.227212
C -0.931764 -3.025491 2.039271
C 0.482217 -3.753262 0.080611
H -0.580379 -2.749408 -2.113600
C -2.604520 -3.235120 -1.521214
C -1.904936 -1.254630 -2.875903
O 1.324719 -1.400763 -2.173141
O 1.945114 1.047356 -1.996638
C 2.811109 -0.661432 -0.164328
C -1.073431 1.719017 1.725667
C -2.072040 2.003739 -1.093006
C 0.617054 3.042502 -0.404305
H -1.316142 -2.157137 2.591327
H -1.818244 -3.570185 1.678474
C -0.108022 -3.916012 2.959507
C 1.316211 -4.619116 1.016909
H -0.335190 -4.361930 -0.329167
H 1.080458 -3.407796 -0.767387
H -2.340909 -3.927580 -0.711790
H -3.497316 -2.690226 -1.176595
C -2.938484 -4.001280 -2.795088
C -2.255804 -2.000179 -4.156673

H -2.787694 -0.695585 -2.526387
H -1.102761 -0.531499 -3.060100
F 2.853810 -1.993055 -0.058259
F 2.547363 -0.156451 1.049308
C 4.198582 -0.188495 -0.639013
H -2.072505 1.257881 1.698960
C -0.196211 0.823103 2.598189
C -1.266872 3.089374 2.376120
H -2.488356 0.985534 -1.141665
C -3.187066 2.883883 -0.533798
C -1.670750 2.380046 -2.521232
H 0.898383 2.828655 -1.442645
C 1.885098 2.961815 0.441193
C 0.062383 4.471100 -0.362204
H 0.700110 -3.314000 3.403256
H -0.730338 -4.264547 3.793564
C 0.500088 -5.093231 2.210578
H 1.724149 -5.474240 0.463051
H 2.179725 -4.036533 1.369905
H -2.062166 -4.591117 -3.103547
H -3.745417 -4.718348 -2.597496
C -3.332463 -3.050215 -3.916663
H -2.578555 -1.286595 -4.926108
H -1.346999 -2.485048 -4.542357
F 4.317251 1.128665 -0.425007
F 4.332256 -0.448833 -1.937303
C 5.356623 -0.905541 0.103524
H -0.058025 -0.152476 2.121854
H 0.807878 1.260985 2.697449
C -0.846379 0.647812 3.963962
C -1.935411 2.901169 3.734541
H -0.292880 3.580115 2.511959
H -1.875201 3.747181 1.746080
H -3.487710 2.532236 0.460907
H -2.852346 3.925965 -0.433681
C -4.389235 2.818767 -1.468638
C -2.889058 2.338364 -3.435457
H -1.247325 3.393130 -2.541311
H -0.879579 1.706956 -2.879761
H 1.651863 3.249962 1.476974
H 2.265368 1.942923 0.473708
C 2.940077 3.896745 -0.131602
C 1.106704 5.416697 -0.950008
H -0.876928 4.561789 -0.915131
H -0.145952 4.770925 0.670401
H 1.120353 -5.696202 2.886036
H -0.307609 -5.752903 1.857718
H -4.272536 -2.546849 -3.643221
H -3.535631 -3.608169 -4.839633
F 5.542813 -2.105937 -0.442498
F 5.046243 -1.053554 1.396928
C 6.689482 -0.126567 0.020309
H -1.798389 0.110910 3.825507
H -0.214689 0.014544 4.599950
C -1.116379 1.986981 4.635155

H -2.086049 3.877770 4.211639
H -2.933524 2.464949 3.578437
H -4.787247 1.792849 -1.446398
H -5.188682 3.465570 -1.087174
C -4.018050 3.205810 -2.893860
H -2.604643 2.658066 -4.445842
H -3.239485 1.300396 -3.524236
H 3.857187 3.837208 0.467934
H 3.200502 3.536923 -1.137526
C 2.429368 5.328879 -0.199424
H 1.266540 5.157642 -2.007300
H 0.723539 6.444965 -0.933403
F 7.686453 -0.933135 0.342717
F 6.876561 0.320593 -1.212087
F 6.677798 0.895861 0.858347
H -1.631135 1.836435 5.592209
H -0.157148 2.474255 4.869327
H -4.895988 3.136288 -3.548389
H -3.702181 4.260744 -2.911240
H 2.284377 5.708845 0.823704
H 3.176061 5.981635 -0.668932

P5 AuCl C2
P -1.596940 -0.921151 0.526835
C -0.067926 0.044768 0.161664
C -2.889845 0.215325 1.315894
C -1.284290 -2.133718 1.892324
S 1.001165 -0.843315 -0.820035
P -0.180572 1.800478 -0.007829
H -2.633434 1.229098 1.001759
C -4.286911 -0.031392 0.735902
C -2.943176 0.228583 2.841859
H -1.351354 -1.544291 2.815920
C -2.411846 -3.166971 1.883669
C 0.079421 -2.801465 1.920416
O 0.488381 -2.194719 -1.026265
O 1.502954 -0.085476 -1.968377
C 2.612735 -1.220073 0.168602
C -0.467231 2.518649 1.683790
C -1.617302 2.079719 -1.142777
C 1.295080 2.624428 -0.756099
H -4.249706 0.000609 -0.360596
H -4.623660 -1.047085 0.988889
C -5.279498 1.000504 1.257757
C -3.922625 1.284017 3.341076
H -3.269677 -0.754936 3.206710
H -1.948976 0.403790 3.267051
H -3.394221 -2.675020 1.885798
H -2.358374 -3.727870 0.939025
C -2.292367 -4.117534 3.068022
C 0.186276 -3.732670 3.121355
H 0.252472 -3.363252 0.996823
H 0.851735 -2.028550 1.982380
F 2.615761 -2.518902 0.451127
F 2.618158 -0.524055 1.321148

C 3.931663 -0.932776 -0.583070
H -1.518921 2.304412 1.907916
C 0.368470 1.792697 2.746082
C -0.314873 4.037866 1.800808
H -2.278987 1.244831 -0.873394
C -2.442668 3.352488 -0.976672
C -1.234962 1.767363 -2.594916
H 1.509170 1.987617 -1.623359
C 2.534539 2.632792 0.142139
C 1.001030 4.041118 -1.273415
H -4.992591 1.993323 0.875269
H -6.276613 0.790721 0.851294
C -5.315435 1.049526 2.777070
H -3.939552 1.286093 4.438640
H -3.568227 2.282724 3.035121
H -2.456409 -3.555197 4.000834
H -3.085580 -4.874168 3.017379
C -0.921195 -4.776855 3.113445
H 1.172305 -4.214094 3.124489
H 0.122816 -3.143616 4.050084
F 3.946688 0.285813 -1.124182
F 4.070018 -1.844736 -1.546286
C 5.159872 -0.988360 0.367870
H 0.211818 0.710073 2.668387
H 1.437405 1.949277 2.553501
C 0.021349 2.295486 4.142364
C -0.695523 4.501906 3.201794
H 0.726795 4.323969 1.614013
H -0.924963 4.552769 1.050210
H -2.761146 3.460989 0.070849
H -1.846332 4.243077 -1.216594
C -3.667071 3.296629 -1.885471
C -2.487596 1.737354 -3.460384
H -0.533326 2.512653 -2.990849
H -0.721533 0.798811 -2.637848
H 2.343909 3.252194 1.028320
H 2.761114 1.629564 0.508674
C 3.736530 3.209365 -0.597226
C 2.216641 4.594686 -2.009765
H 0.140137 4.042980 -1.945594
H 0.757271 4.715621 -0.445672
H -6.007439 1.828095 3.122143
H -5.701985 0.094492 3.163481
H -0.801925 -5.420094 2.228949
H -0.840673 -5.432178 3.990206
F 5.025305 -1.968549 1.263204
F 5.237190 0.181983 1.014341
C 6.495059 -1.206729 -0.377902
H -1.015489 2.013300 4.381607
H 0.657737 1.792834 4.881238
C 0.151922 3.806827 4.256091
H -0.586139 5.591420 3.270273
H -1.759238 4.282905 3.382946
H -4.330406 2.489893 -1.538682
H -4.236793 4.230358 -1.795969

C -3.283626 3.027700 -3.333875
H -2.203959 1.553105 -4.503180
H -3.107956 0.879047 -3.162887
H 4.607788 3.205961 0.069865
H 3.987946 2.555933 -1.441128
C 3.449540 4.607014 -1.120020
H 2.412804 3.974075 -2.896443
H 1.988951 5.603523 -2.376644
F 7.495870 -0.929399 0.442951
F 6.600183 -2.461180 -0.774104
F 6.568749 -0.405475 -1.429904
H -0.134119 4.140563 5.261264
H 1.205474 4.094866 4.119955
H -4.184620 2.982610 -3.957937
H -2.684543 3.871103 -3.712574
H 3.281716 5.292933 -0.274957
H 4.315447 4.996183 -1.669924
Au -2.469970 -1.688954 -1.437167
Cl -3.450658 -2.212062 -3.472544

P5 AuCl C3
P 1.081194 -1.368061 -0.442442
C 0.556416 0.366583 -0.759146
C 2.916365 -1.346837 -0.037134
C 1.073332 -2.443702 -1.958910
S -0.978609 0.537472 -1.455635
P 1.341979 1.715414 0.084446
H 3.085590 -0.471468 0.600803
C 3.283843 -2.566592 0.814211
C 3.870687 -1.241373 -1.225013
H 1.965712 -3.055670 -1.741020
C -0.093031 -3.429861 -2.019081
C 1.299726 -1.739795 -3.295256
O -1.172347 1.847385 -2.069958
O -1.318033 -0.684509 -2.184742
C -2.381875 0.536377 -0.191178
C 3.015182 2.049562 -0.628685
C 1.479914 1.147160 1.842854
C 0.379096 3.285695 0.031585
H 2.615942 -2.641608 1.680873
H 3.123924 -3.483876 0.225857
C 4.734146 -2.488829 1.275054
C 5.315803 -1.136106 -0.751890
H 3.772855 -2.134423 -1.856536
H 3.614381 -0.390961 -1.859566
H -0.192142 -3.944066 -1.054221
H -1.025815 -2.884578 -2.188670
C 0.134489 -4.438462 -3.138468
C 1.513341 -2.764631 -4.403462
H 0.419104 -1.130581 -3.519107
H 2.153979 -1.056059 -3.242047
F -2.425374 1.731561 0.426580
F -2.190883 -0.404565 0.731181
C -3.747496 0.263415 -0.871554
H 3.627782 1.197246 -0.305510

C 3.000687 2.058769 -2.161961
C 3.708038 3.315914 -0.113342
H 1.650994 0.069140 1.714359
C 2.649043 1.664088 2.676531
C 0.129507 1.205607 2.565636
H -0.648637 2.932223 0.173520
C 0.418760 4.063782 -1.291194
C 0.687179 4.270004 1.171534
H 4.840315 -1.629292 1.956315
H 4.982042 -3.380446 1.864098
C 5.700062 -2.327969 0.111152
H 5.985114 -1.049748 -1.617895
H 5.442329 -0.207355 -0.170495
H 1.015053 -5.056812 -2.901394
H -0.719687 -5.124960 -3.194300
C 0.354356 -3.748928 -4.477853
H 1.643973 -2.248763 -5.363619
H 2.448295 -3.317352 -4.216682
F -3.884278 -1.053882 -1.038960
F -3.811727 0.872862 -2.054127
C -4.931294 0.772937 -0.011163
H 2.462204 1.185183 -2.547804
H 2.444626 2.934563 -2.514785
C 4.425253 2.127406 -2.700269
C 5.138407 3.372259 -0.638688
H 3.166901 4.197712 -0.476845
H 3.700188 3.370936 0.979097
H 3.594388 1.496865 2.141704
H 2.563713 2.747312 2.841704
C 2.688840 0.946411 4.021725
C 0.223590 0.517533 3.922591
H -0.212947 2.238307 2.699801
H -0.623155 0.696483 1.956406
H 1.385932 4.577998 -1.378550
H 0.303515 3.400316 -2.147101
C -0.696804 5.099988 -1.280216
C -0.413863 5.326596 1.213830
H 0.760389 3.778056 2.143432
H 1.653586 4.757489 0.991653
H 6.729955 -2.225323 0.475651
H 5.677214 -3.237973 -0.507073
H -0.559831 -3.204796 -4.757081
H 0.532984 -4.490411 -5.267406
F -5.037770 2.094479 -0.166818
F -4.704741 0.496970 1.277734
C -6.286088 0.137487 -0.399782
H 4.969752 1.216221 -2.410871
H 4.405488 2.143286 -3.797037
C 5.168997 3.340733 -2.159891
H 5.635282 4.274634 -0.260974
H 5.703954 2.515311 -0.240869
H 2.906196 -0.119522 3.850100
H 3.515793 1.341848 4.625000
C 1.364873 1.071149 4.760536
H -0.734347 0.621144 4.445737

H 0.363318 -0.562004 3.768610
H -0.702555 5.648330 -2.230457
H -1.656855 4.567305 -1.220850
C -0.544860 6.063743 -0.111869
H -1.365474 4.830389 1.455781
H -0.214232 6.034126 2.028699
F -7.263701 0.852085 0.136059
F -6.429781 0.135486 -1.716765
F -6.364559 -1.100024 0.050784
H 6.204427 3.347800 -2.522555
H 4.697284 4.256720 -2.546668
H 1.417715 0.546853 5.722487
H 1.179018 2.131871 4.992879
H 0.356749 6.675883 -0.268884
H -1.389952 6.762625 -0.074318
Au -0.005350 -2.294787 1.330732
Cl -0.988754 -3.169191 3.243415

P1 Au^+ C1
Au -2.452993 -0.169839 -1.339192
P -1.291987 -0.771131 0.562719
C 0.279704 0.038069 0.632347
C -2.417563 -0.451910 2.006625
C -1.174737 -2.603026 0.352548
S 1.134383 -0.453670 2.089730
P 0.897534 1.277269 -0.462779
H -2.302113 -1.386983 2.576421
C -3.878719 -0.292172 1.580264
C -2.005876 0.695628 2.927649
H -0.612119 -2.918057 1.241319
C -2.532997 -3.301447 0.369220
C -0.396925 -2.967899 -0.906560
O 0.234327 -1.464725 2.676121
O 1.537157 0.686439 2.914064
C 2.639534 -1.298468 1.670707
C -0.416173 2.397469 -1.099563
C 2.191453 2.229804 0.422693
C 1.707319 0.598711 -1.972616
H -3.970786 0.649666 1.013068
H -4.183330 -1.096316 0.897769
C -4.795191 -0.247326 2.796793
C -2.915816 0.721680 4.151480
H -0.964087 0.600941 3.239576
H -2.099060 1.652482 2.397906
H -3.081239 -3.073090 1.290521
H -3.144109 -2.927916 -0.470283
C -2.354209 -4.811930 0.243874
C -0.206233 -4.474478 -1.011976
H -0.963016 -2.616597 -1.785411
H 0.566349 -2.445075 -0.923135
C 3.862827 -0.673255 1.873633
C 2.575799 -2.594458 1.167641
H -0.957708 1.744449 -1.804176
C -1.442837 2.874219 -0.077822
C 0.120020 3.564355 -1.934033

H 2.705871 1.455373 1.003037
C 3.258453 2.902981 -0.445141
C 1.595943 3.227401 1.420405
H 2.187951 1.470217 -2.441535
C 0.730229 -0.006201 -2.979509
C 2.786578 -0.408598 -1.578570
H -5.833671 -0.110146 2.471269
H -4.754784 -1.220478 3.308457
C -4.380689 0.853186 3.762306
H -2.766245 -0.203913 4.726478
H -2.614998 1.545557 4.809762
H -1.841599 -5.182409 1.143629
H -3.337751 -5.296596 0.226448
C -1.547835 -5.192418 -0.989329
H 0.349707 -4.715576 -1.926875
H 0.411438 -4.822571 -0.169651
H 3.897081 0.309083 2.339589
C 5.028950 -1.329472 1.496457
C 3.746364 -3.236835 0.800584
H 1.616451 -3.096609 1.075969
H -1.809698 2.026716 0.509246
H -0.980638 3.568048 0.633400
C -2.597622 3.561513 -0.792538
C -1.038268 4.263972 -2.638761
H 0.632225 4.279363 -1.276364
H 0.858364 3.223039 -2.670661
H 3.705400 2.197252 -1.153337
H 2.815342 3.715268 -1.036760
C 4.355099 3.460825 0.459307
C 2.695868 3.760203 2.326428
H 1.150249 4.063931 0.863150
H 0.807410 2.752276 2.012495
H 0.152129 -0.795410 -2.479322
H 0.012368 0.748382 -3.326766
C 1.460088 -0.595211 -4.181914
C 3.505961 -0.976716 -2.792806
H 2.307201 -1.229527 -1.033590
H 3.503954 0.038270 -0.879021
H -4.542192 1.831620 3.284210
H -5.018603 0.836114 4.654120
H -1.403036 -6.278549 -1.028464
H -2.114615 -4.922296 -1.893322
H 5.989533 -0.840400 1.648914
C 4.992373 -2.610947 0.942601
H 3.698704 -4.249923 0.404105
H -3.337760 3.908460 -0.061284
H -3.114420 2.818977 -1.426758
C -2.109029 4.715734 -1.655588
H -0.658702 5.117073 -3.213631
H -1.480670 3.570431 -3.370919
H 5.116443 3.959353 -0.152889
H 4.858927 2.616722 0.955337
C 3.806646 4.410383 1.514164
H 3.089823 2.920947 2.918161
H 2.272793 4.474237 3.043128

H 0.731983 -1.053755 -4.862458
H 1.938692 0.220388 -4.744222
C 2.518270 -1.604363 -3.763897
H 4.064082 -0.176216 -3.301083
H 4.249373 -1.712288 -2.460832
C 6.250176 -3.323248 0.553507
H -2.947021 5.176314 -2.192120
H -1.693470 5.497305 -1.002352
H 3.412571 5.310517 1.018298
H 4.615480 4.751116 2.171693
H 3.038509 -1.997515 -4.645382
H 2.030510 -2.464940 -3.279337
H 6.145491 -3.815557 -0.420113
H 6.491559 -4.105894 1.284935
H 7.103068 -2.639600 0.505900

P1Au$^+$ C2
P -0.504591 -1.373104 0.082727
C 2.858048 0.352606 -1.082882
C 3.775467 1.236839 -1.642975
C 0.012899 0.257163 -0.417666
C -0.294455 -2.002436 1.825097
C -2.274561 -1.690336 -0.330292
S 1.185220 0.393771 -1.715332
C 3.260532 -0.565869 -0.125005
H 3.443393 1.953583 -2.389925
C 5.098329 1.193992 -1.225137
P -0.596989 1.817919 0.194645
H -0.645366 -3.031310 1.647602
C -1.190140 -1.411481 2.913001
C 1.154356 -2.122840 2.287800
H -2.861920 -1.013778 0.304078
C -2.720126 -3.117497 -0.010170
C -2.532771 -1.351038 -1.797784
O 1.048504 1.732325 -2.299588
O 1.072980 -0.744670 -2.650260
C 4.586479 -0.596624 0.285290
H 2.535782 -1.238752 0.320577
H 5.813884 1.890195 -1.659700
C 5.530433 0.278574 -0.259668
C -2.055623 1.441336 1.263603
C -1.068940 3.066841 -1.070319
C 0.663383 2.664251 1.248503
H -2.235927 -1.377547 2.576815
H -0.885563 -0.378481 3.127717
C -1.081118 -2.218525 4.201977
C 1.251497 -2.891576 3.601171
H 1.592834 -1.124209 2.406763
H 1.737431 -2.646342 1.518471
H -2.076690 -3.822953 -0.561847
H -2.596756 -3.341086 1.056319
C -4.180483 -3.329173 -0.397825
C -3.996924 -1.555383 -2.158717
H -2.210274 -0.326264 -2.014834
H -1.901726 -2.002580 -2.423520

H 4.894221 -1.311588 1.046663
C 6.966655 0.228319 0.159518
H -1.870466 0.415687 1.593219
C -2.171704 2.296636 2.529675
C -3.383838 1.434542 0.498601
H -0.090112 3.334451 -1.479640
C -1.658913 4.339145 -0.452251
C -1.883853 2.567022 -2.262187
H 0.089837 3.369263 1.867597
C 1.336961 1.637182 2.155078
C 1.689753 3.472227 0.448050
H -1.465755 -3.234412 4.028050
H -1.723927 -1.769234 4.968978
C 0.360722 -2.293481 4.678298
H 0.954120 -3.936046 3.425221
H 2.296860 -2.918954 3.931702
H -4.813898 -2.702345 0.248476
H -4.464276 -4.368307 -0.191843
C -4.444127 -2.976833 -1.853318
H -4.152055 -1.316590 -3.217666
H -4.614945 -0.845515 -1.585791
H 7.094118 -0.309323 1.104054
H 7.385955 1.233937 0.273006
H 7.569880 -0.288037 -0.599447
H -2.326298 3.348964 2.250768
H -1.245504 2.256264 3.113863
C -3.328451 1.815311 3.401018
C -4.505993 0.911543 1.385619
H -3.632827 2.454250 0.184572
H -3.304498 0.845340 -0.419665
H -2.665783 4.155242 -0.055784
H -1.048949 4.691019 0.390577
C -1.734418 5.422507 -1.525144
C -1.942687 3.664831 -3.317622
H -1.411192 1.669724 -2.674444
H -2.904915 2.305975 -1.960740
H 1.808761 0.883014 1.514413
H 0.586202 1.107318 2.757392
C 2.402113 2.258259 3.045638
C 2.760145 4.062480 1.357145
H 1.192659 4.279001 -0.102387
H 2.160453 2.828584 -0.302518
H 0.714497 -1.281079 4.927436
H 0.429826 -2.881571 5.600961
H -5.507237 -3.105702 -2.088826
H -3.895148 -3.674994 -2.502770
H -3.089283 0.810985 3.783305
H -3.415457 2.465457 4.279688
C -4.643612 1.759079 2.640386
H -4.295925 -0.132583 1.672034
H -5.445581 0.893220 0.819846
H -0.711527 5.691471 -1.828478
H -2.183127 6.329615 -1.102112
C -2.516444 4.953567 -2.744753
H -2.538827 3.324888 -4.173089

H -0.925829 3.844026 -3.695862
H 1.935945 2.958067 3.755233
H 2.876427 1.472872 3.647618
C 3.432775 2.993501 2.203398
H 3.497757 4.594774 0.744476
H 2.303805 4.815230 2.017684
H -4.946383 2.777497 2.355399
H -5.439729 1.368457 3.285120
H -2.527677 5.740143 -3.508810
H -3.566284 4.787202 -2.457980
H 3.937771 2.272439 1.541424
H 4.207135 3.437121 2.840507
Au 0.683269 -2.838069 -1.237687

P1Au$^+$ C3

P 0.692751 1.088933 0.289309
C 2.033394 -1.780355 -1.280562
C 3.159266 -1.328148 -1.996997
C -0.265330 -0.271323 -0.342777
C 0.300950 2.820409 -0.224639
C 0.787293 1.052099 2.124360
S 0.418998 -1.106367 -1.712917
C 2.180618 -2.710579 -0.264142
H 3.009000 -0.701252 -2.876296
C 4.430848 -1.767117 -1.615353
P -1.977737 -0.681052 -0.090414
H 1.161760 3.354533 0.208918
C -0.964203 3.443634 0.362614
C 0.384157 2.982344 -1.741960
H -0.231422 1.194095 2.505234
C 1.647264 2.187006 2.680031
C 1.308728 -0.305716 2.595013
O 0.706532 -0.177930 -2.813924
O -0.455488 -2.262639 -1.954630
C 3.453937 -3.148976 0.085992
H 1.308502 -3.080231 0.266179
H 5.300456 -1.420749 -2.172015
C 4.598359 -2.676887 -0.562151
C -2.546543 0.435508 1.275098
C -2.332015 -2.436792 0.319177
C -3.069816 -0.330989 -1.545353
H -0.969034 3.350081 1.458604
H -1.849810 2.910624 -0.006180
C -1.086295 4.905116 -0.053229
C 0.216181 4.441393 -2.145428
H -0.386235 2.367222 -2.218628
H 1.342123 2.585989 -2.105334
H 2.659747 2.107598 2.250667
H 1.250032 3.163864 2.378853
C 1.715002 2.111050 4.201748
C 1.357703 -0.372085 4.114922
H 0.696387 -1.114387 2.176785
H 2.322319 -0.448897 2.184725
H 3.561131 -3.874579 0.890541
C 5.961079 -3.165533 -0.183112
H -1.862305 1.287246 1.200835

C -3.960174 1.003410 1.119849
C -2.361025 -0.173040 2.668978
H -2.109210 -2.914222 -0.642903
C -3.810641 -2.693160 0.630943
C -1.425862 -3.105465 1.346946
H -4.049708 -0.179256 -1.068935
C -2.649944 0.969384 -2.222575
C -3.262524 -1.429185 -2.595116
H -0.255206 5.478929 0.383102
H -2.009282 5.329475 0.360889
C -1.058336 5.040710 -1.568803
H 1.080952 5.019768 -1.786774
H 0.221794 4.521248 -3.239077
H 0.710457 2.298870 4.611249
H 2.357954 2.914126 4.581533
C 2.207961 0.754799 4.683501
H 1.742568 -1.348628 4.433504
H 0.333561 -0.295627 4.513875
H 5.989567 -3.516539 0.853034
H 6.717873 -2.383949 -0.307277
H 6.256177 -4.007740 -0.823474
H -4.694280 0.184368 1.148135
H -4.074322 1.500765 0.149979
C -4.259007 2.009004 2.227525
C -2.645271 0.858397 3.753454
H -3.053068 -1.013559 2.796514
H -1.353558 -0.585078 2.781376
H -4.074643 -2.272148 1.610097
H -4.465373 -2.209852 -0.104666
C -4.066436 -4.197530 0.647094
C -1.679553 -4.608285 1.345148
H -0.380370 -2.881909 1.112833
H -1.618003 -2.712616 2.352341
H -1.681778 0.793390 -2.710695
H -2.482776 1.759385 -1.479798
C -3.680008 1.416106 -3.249727
C -4.319258 -0.985537 -3.602058
H -3.564496 -2.375809 -2.132548
H -2.315612 -1.620823 -3.107949
H -1.930855 4.520648 -1.995314
H -1.152487 6.093793 -1.859338
H 2.210634 0.718581 5.779398
H 3.252018 0.613874 4.365526
H -3.597909 2.880426 2.099452
H -5.284040 2.381178 2.113345
C -4.050295 1.422007 3.614084
H -1.915273 1.681743 3.683217
H -2.500971 0.402886 4.741164
H -3.905526 -4.594214 -0.366422
H -5.116983 -4.391988 0.894808
C -3.145333 -4.914130 1.625372
H -1.037686 -5.097764 2.088225
H -1.397064 -5.018044 0.364192
H -4.620475 1.666307 -2.734708
H -3.337582 2.337429 -3.738134

C -3.935609 0.323511 -4.277180
H -4.460566 -1.774740 -4.350327
H -5.287218 -0.865354 -3.090957
H -4.780904 0.617566 3.785238
H -4.239256 2.180900 4.382482
H -3.322735 -5.995598 1.588380
H -3.389566 -4.595794 2.650388
H -3.022316 0.167375 -4.869615
H -4.716180 0.635854 -4.981343
Au 2.817189 0.642140 -0.507713

P3Au^{+} C1
Au 1.428000 -2.098884 -1.029927
P 1.376073 -0.053473 0.041824
C -0.208698 0.683928 0.134697
C 2.236236 -0.460082 1.636923
C 2.493476 1.119310 -0.855757
P -1.740936 -0.144582 0.010155
C -0.150600 2.171732 0.383165
H 2.276528 0.517042 2.148134
C 3.661249 -0.974595 1.438443
C 1.461139 -1.452683 2.492261
H 1.769032 1.632424 -1.493944
C 3.145496 2.180798 0.041380
C 3.513653 0.474819 -1.800492
C -3.172798 1.004744 -0.060799
C -2.149313 -1.252849 1.464126
C -1.839875 -1.261655 -1.461299
C -0.086253 3.112139 -0.671412
C -0.069913 2.662157 1.714241
H 4.256165 -0.287541 0.830961
H 3.614321 -1.925803 0.881699
C 4.350315 -1.198261 2.781681
C 2.125063 -1.654984 3.847153
H 0.419094 -1.138748 2.610575
H 1.436333 -2.416218 1.957830
H 2.401119 2.652316 0.692564
H 3.893732 1.712910 0.695170
C 3.829738 3.238141 -0.817171
C 4.211443 1.547026 -2.630583
H 4.259157 -0.099160 -1.233935
H 3.008341 -0.241113 -2.463082
H -3.994718 0.269540 -0.061004
C -3.333695 1.848136 -1.323024
C -3.355685 1.896051 1.172057
H -1.636121 -0.722027 2.280979
C -1.525950 -2.643250 1.327791
C -3.616850 -1.410945 1.885301
H -1.238900 -2.128260 -1.142638
C -3.251186 -1.784356 -1.738705
C -1.192290 -0.744416 -2.743742
C 0.087986 4.468604 -0.384064
C -0.183481 2.749162 -2.126653
C 0.108037 4.021199 1.949391
C -0.236474 1.770839 2.908134

H 5.361819 -1.587574 2.613338
H 4.472047 -0.226624 3.283291
C 3.558539 -2.136441 3.679822
H 1.542947 -2.370148 4.441982
H 2.112783 -0.704185 4.400964
H 3.063772 3.772849 -1.400085
H 4.301691 3.986556 -0.168760
C 4.850954 2.616453 -1.756964
H 3.471353 2.018147 -3.295772
H 4.959879 1.079540 -3.282088
H -2.584398 2.645427 -1.311558
H -3.165907 1.255602 -2.228377
C -4.726673 2.469217 -1.352163
C -4.751861 2.507151 1.162980
H -3.188730 1.340219 2.101226
H -2.609653 2.698565 1.144985
H -2.086624 -3.205679 0.566047
H -0.491908 -2.577633 0.963934
C -1.588086 -3.403949 2.645387
C -3.697204 -2.163659 3.210564
H -4.121994 -0.447102 1.989193
H -4.170121 -1.973351 1.120866
H -3.717956 -2.161713 -0.822010
H -3.883130 -0.959124 -2.097967
C -3.207472 -2.887363 -2.790950
C -1.135667 -1.860512 -3.778779
H -0.185453 -0.364602 -2.533438
H -1.767981 0.097123 -3.149960
H 0.141032 5.172422 -1.214778
C 0.203420 4.949937 0.912967
H -0.963836 3.338718 -2.622011
H -0.408799 1.692145 -2.267236
H 0.750597 2.978141 -2.656879
H 0.172131 4.369158 2.980192
H 0.186580 2.231708 3.806197
H 0.222405 0.793467 2.767294
H -1.300874 1.591393 3.111464
H 4.048690 -2.235013 4.655700
H 3.549142 -3.142439 3.233765
H 5.317834 3.387378 -2.381808
H 5.661325 2.165354 -1.164448
H -4.840194 3.078152 -2.257403
H -5.477132 1.666760 -1.421608
C -4.989821 3.307787 -0.109453
H -5.501166 1.703802 1.241995
H -4.883161 3.142627 2.047191
H -1.134709 -4.395948 2.525526
H -0.985333 -2.873061 3.397789
C -3.024186 -3.525568 3.134768
H -3.213564 -1.557925 3.991919
H -4.747376 -2.270283 3.508337
H -4.225099 -3.235060 -3.005969
H -2.665797 -3.751757 -2.376564
C -2.519941 -2.423315 -4.066701
H -0.664770 -1.495872 -4.699871

H -0.483875 -2.665756 -3.395636
C 0.418880 6.403993 1.198105
H -6.011308 3.706383 -0.126638
H -4.315510 4.177219 -0.114361
H -3.055390 -4.022982 4.111528
H -3.586738 -4.168547 2.441426
H -2.457801 -3.247323 -4.787523
H -3.130049 -1.641361 -4.543198
H 1.394901 6.569863 1.671875
H -0.340386 6.788913 1.889610
H 0.382718 7.004045 0.283775

P3 Au^{+} C2
Au -2.639284 1.460617 -0.772969
P -1.373257 -0.302251 0.030400
C 0.234076 0.385051 0.165565
C -2.328674 -0.822173 1.539235
C -1.460770 -1.766824 -1.122289
P 1.765172 -0.466177 0.179284
C 0.309011 1.867243 -0.100851
H -1.861782 -1.767454 1.861375
C -3.799089 -1.079483 1.197945
C -2.244986 0.184309 2.677877
H -0.486752 -1.721080 -1.629551
C -1.588815 -3.129910 -0.429456
C -2.525103 -1.593581 -2.215151
C 3.203834 0.660560 0.438574
C 2.216604 -1.413496 -1.396933
C 1.840283 -1.768317 1.483096
C 0.365341 2.818484 0.937001
C 0.331671 2.334957 -1.443058
H -3.912140 -1.776959 0.361845
H -4.238969 -0.126678 0.860185
C -4.566657 -1.585113 2.414051
C -3.012016 -0.300312 3.902387
H -1.204375 0.380918 2.939904
H -2.663828 1.141754 2.325516
H -0.789054 -3.266509 0.312012
H -2.527819 -3.173665 0.133443
C -1.583488 -4.274452 -1.438320
C -2.501386 -2.754827 -3.198907
H -3.519726 -1.513907 -1.752916
H -2.356372 -0.644504 -2.740894
H 4.013481 -0.069077 0.273538
C 3.420113 1.227105 1.842387
C 3.404525 1.798307 -0.572713
H 1.554111 -0.939139 -2.133740
C 1.876892 -2.908491 -1.370191
C 3.654874 -1.266905 -1.912759
H 1.260213 -2.588943 1.033807
C 3.257448 -2.298185 1.722590
C 1.155344 -1.440967 2.806267
C 0.490271 4.176646 0.625718
C 0.282564 2.453319 2.388463
C 0.430969 3.697712 -1.703839

C 0.308840 1.403855 -2.622031
H -5.615394 -1.753746 2.140533
H -4.163145 -2.563364 2.716213
C -4.464822 -0.608064 3.575098
H -2.945269 0.451126 4.698724
H -2.522917 -1.206988 4.290879
H -0.605526 -4.325535 -1.937210
H -1.712009 -5.225964 -0.908007
C -2.664863 -4.090630 -2.491069
H -1.542650 -2.746032 -3.740774
H -3.285429 -2.613563 -3.952610
H 2.730132 2.060813 2.002411
H 3.217797 0.487105 2.622061
C 4.856686 1.729621 1.960441
C 4.849802 2.276769 -0.502458
H 3.131294 1.507166 -1.592601
H 2.746596 2.633068 -0.307829
H 2.578041 -3.429798 -0.704643
H 0.881195 -3.091819 -0.962859
C 1.977494 -3.511891 -2.766207
C 3.782942 -1.862204 -3.311808
H 3.978877 -0.224953 -1.940977
H 4.342097 -1.790373 -1.231058
H 3.770074 -2.510996 0.776715
H 3.853219 -1.526023 2.228472
C 3.207084 -3.545075 2.597597
C 1.101042 -2.687345 3.681926
H 0.151953 -1.051177 2.619516
H 1.700645 -0.647096 3.333369
H 0.555209 4.894030 1.443653
C 0.528807 4.643198 -0.681777
H 0.913965 3.113645 2.993070
H 0.582896 1.418033 2.565667
H -0.743869 2.573241 2.756842
H 0.456113 4.033163 -2.740776
H -0.070008 1.914861 -3.513125
H -0.304835 0.513525 -2.447197
H 1.321171 1.052734 -2.867693
H -4.982043 -1.004025 4.457198
H -4.980346 0.325930 3.305534
H -2.635228 -4.914355 -3.214075
H -3.654923 -4.132324 -2.012541
H 5.022512 2.141824 2.963402
H 5.542886 0.874456 1.859478
C 5.179932 2.772812 0.899243
H 5.526260 1.452821 -0.779451
H 5.012803 3.074588 -1.237274
H 1.717089 -4.577222 -2.726724
H 1.228553 -3.032381 -3.417090
C 3.368189 -3.323596 -3.351010
H 3.150933 -1.281457 -4.001024
H 4.814308 -1.744645 -3.666003
H 4.224013 -3.910777 2.784233
H 2.683609 -4.344867 2.051479
C 2.489435 -3.269857 3.912196

H 0.618817 -2.450834 4.638666
H 0.464729 -3.440783 3.190795
C 0.663633 6.102124 -0.994052
H 6.233944 3.068592 0.965289
H 4.589096 3.679056 1.099226
H 3.410526 -3.708663 -4.376840
H 4.084944 -3.918862 -2.765954
H 2.424661 -4.186912 4.509852
H 3.083617 -2.556866 4.503245
H -0.210471 6.470035 -1.546044
H 1.541247 6.292044 -1.624158
H 0.763728 6.701208 -0.083885

P5 Au^+ C1
Au -3.109711 -0.772264 1.003108
P -1.193100 -1.317953 -0.159117
C -0.212660 0.146189 -0.428593
C -0.268527 -2.568749 0.854125
C -1.832029 -2.134994 -1.679990
S 1.164825 -0.151289 -1.384826
P -0.751736 1.765617 0.083908
H 0.611826 -2.001434 1.187776
C -1.029801 -3.018527 2.106001
C 0.210474 -3.777355 0.039251
H -0.964838 -2.693317 -2.055710
C -2.969389 -3.106759 -1.363507
C -2.259078 -1.135999 -2.744606
O 0.941057 -1.380774 -2.145170
O 1.653507 1.057311 -2.037665
C 2.598361 -0.689964 -0.244922
C -1.158516 1.735392 1.882354
C -2.274790 2.095738 -0.894770
C 0.474487 3.069094 -0.303358
H -1.330693 -2.141988 2.698410
H -1.957091 -3.528302 1.799139
C -0.175865 -3.952036 2.954239
C 1.075891 -4.682126 0.908291
H -0.660126 -4.347428 -0.311608
H 0.761216 -3.445663 -0.845782
H -2.692443 -3.810818 -0.568407
H -3.830596 -2.530538 -0.984060
C -3.387306 -3.851205 -2.626617
C -2.694032 -1.865629 -4.009745
H -3.103095 -0.543579 -2.356052
H -1.435341 -0.447239 -2.960535
F 2.597587 -2.021456 -0.174148
F 2.391863 -0.205742 0.985607
C 3.967380 -0.233323 -0.788141
H -2.170713 1.291315 1.902338
C -0.240083 0.815773 2.686563
C -1.277037 3.102551 2.560176
H -2.729835 1.092225 -0.930982
C -3.327089 3.028829 -0.298296
C -1.904180 2.463515 -2.335896
H 0.702742 2.854754 -1.355071

C 1.775835 2.945480 0.486218
C -0.043325 4.511933 -0.230934
H 0.681884 -3.385295 3.346634
H -0.753250 -4.285589 3.825095
C 0.329825 -5.141205 2.151219
H 1.407301 -5.542163 0.314159
H 1.985080 -4.137029 1.199806
H -2.548072 -4.472300 -2.971913
H -4.209106 -4.538988 -2.393800
C -3.794385 -2.878900 -3.724871
H -3.024520 -1.136598 -4.759798
H -1.820107 -2.376999 -4.437940
F 4.104649 1.078561 -0.564399
F 4.010531 -0.479182 -2.094142
C 5.146883 -0.983394 -0.115854
H -0.144531 -0.159223 2.197304
H 0.772855 1.241332 2.709231
C -0.775697 0.647683 4.101387
C -1.823954 2.930148 3.973146
H -0.282605 3.564801 2.612605
H -1.913585 3.783202 1.984925
H -3.613697 2.690484 0.707108
H -2.927275 4.045121 -0.186392
C -4.555304 3.066606 -1.202551
C -3.148688 2.518644 -3.212607
H -1.417208 3.446698 -2.354655
H -1.168097 1.749367 -2.728766
H 1.596923 3.230089 1.533493
H 2.133885 1.917704 0.494895
C 2.828376 3.860160 -0.124117
C 1.004692 5.435114 -0.849009
H -0.995686 4.632372 -0.755384
H -0.210934 4.808478 0.810190
H 0.974596 -5.771446 2.774989
H -0.523565 -5.769016 1.853549
H -4.706330 -2.346636 -3.413107
H -4.054255 -3.425296 -4.639140
F 5.264353 -2.181781 -0.680765
F 4.889357 -1.133764 1.189332
C 6.490974 -0.231892 -0.263782
H -1.745512 0.123264 4.049399
H -0.105358 -0.001367 4.677864
C -0.958764 1.988393 4.797828
H -1.898591 3.909914 4.459599
H -2.850476 2.535327 3.915555
H -5.026066 2.070775 -1.208233
H -5.297544 3.758097 -0.786228
C -4.192768 3.459286 -2.627760
H -2.868953 2.831077 -4.225575
H -3.577274 1.510405 -3.306505
H 3.766648 3.769064 0.436211
H 3.037365 3.504295 -1.143332
C 2.352608 5.304899 -0.153250
H 1.114042 5.185480 -1.914737
H 0.647053 6.471039 -0.805506

F 7.482310 -1.060619 -0.002870
F 6.611267 0.222643 -1.501256
F 6.536056 0.782523 0.582039
H -1.392116 1.846094 5.794796
H 0.028332 2.448422 4.952446
H -5.090480 3.471424 -3.257006
H -3.799779 4.487133 -2.630943
H 2.261665 5.677102 0.878538
H 3.095423 5.942779 -0.646678

P5 Au^{+} C2
Au -2.374567 -1.770024 -1.863231
P -1.721613 -0.989615 0.200480
C -0.220170 -0.030782 -0.002425
C -3.132362 0.046565 0.878239
C -1.560825 -2.388745 1.393307
S 0.923599 -0.862393 -0.979422
P -0.258918 1.753761 0.037659
H -2.817756 1.083300 0.748455
C -4.422533 -0.089489 0.063408
C -3.405829 -0.149535 2.371211
H -1.751841 -1.883787 2.351256
C -2.669704 -3.411617 1.151024
C -0.200543 -3.052455 1.525974
O 0.388142 -2.189627 -1.295679
O 1.441242 -0.036534 -2.066251
C 2.460533 -1.309604 0.083425
C -0.734961 2.272629 1.755778
C -1.570105 2.195313 -1.194078
C 1.335563 2.568401 -0.429886
H -4.213824 0.107821 -0.997671
H -4.790453 -1.124460 0.116972
C -5.480354 0.882242 0.573289
C -4.451282 0.848712 2.853875
H -3.774927 -1.168351 2.549590
H -2.484817 -0.039522 2.954262
H -3.649187 -2.921747 1.058081
H -2.477302 -3.904234 0.185429
C -2.692301 -4.450059 2.266511
C -0.239767 -4.072508 2.659318
H 0.075709 -3.547596 0.589834
H 0.558013 -2.290766 1.733562
F 2.468774 -2.626302 0.213033
F 2.333723 -0.746646 1.293988
C 3.806105 -0.883134 -0.546062
H -1.811977 2.073078 1.819877
C -0.058671 1.402370 2.824946
C -0.581282 3.767688 2.062922
H -2.290794 1.380151 -1.042994
C -2.346304 3.493365 -0.985014
C -1.082645 1.983852 -2.631677
H 1.675398 1.997027 -1.303379
C 2.412105 2.492693 0.660788
C 1.117224 4.026243 -0.871222
H -5.136870 1.910675 0.379290

H -6.404289 0.751459 -0.002488
C -5.742339 0.719491 2.062073
H -4.631249 0.697954 3.925126
H -4.052628 1.871431 2.748390
H -2.983446 -3.959860 3.208160
H -3.464747 -5.199112 2.054983
C -1.330974 -5.109406 2.435337
H 0.741033 -4.555210 2.744364
H -0.416143 -3.554616 3.614631
F 3.756714 0.385601 -0.955148
F 4.039363 -1.677888 -1.587756
C 4.975179 -0.973636 0.472690
H -0.230362 0.340083 2.610433
H 1.029243 1.536878 2.786437
C -0.572962 1.759234 4.215337
C -1.136246 4.073685 3.449234
H 0.476072 4.053409 2.040714
H -1.088534 4.377396 1.307533
H -2.759240 3.523923 0.033470
H -1.685783 4.364281 -1.084280
C -3.483570 3.590488 -1.998381
C -2.259013 2.093520 -3.593266
H -0.319569 2.722672 -2.904975
H -0.601815 1.002561 -2.718607
H 2.059840 3.013440 1.558457
H 2.601663 1.459372 0.956195
C 3.711036 3.155201 0.212448
C 2.434614 4.649538 -1.321095
H 0.393764 4.083287 -1.687764
H 0.710291 4.620960 -0.045510
H -6.477823 1.457517 2.402845
H -6.184325 -0.269889 2.250858
H -1.096246 -5.690658 1.531419
H -1.356705 -5.824434 3.266161
F 4.838085 -2.059497 1.234263
F 4.923086 0.115083 1.250228
C 6.366040 -1.018642 -0.201220
H -1.635549 1.481149 4.291622
H -0.041417 1.159572 4.963969
C -0.436998 3.243995 4.514205
H -1.028776 5.145071 3.655858
H -2.216647 3.859970 3.465545
H -4.223975 2.805617 -1.778453
H -4.002742 4.548569 -1.876726
C -2.991940 3.415961 -3.427443
H -1.903815 1.967838 -4.622842
H -2.957609 1.261833 -3.400635
H 4.435324 3.100491 1.034276
H 4.146534 2.590289 -0.619615
C 3.483038 4.592487 -0.222679
H 2.802289 4.112818 -2.207842
H 2.250638 5.684112 -1.635099
F 7.290607 -0.788798 0.711325
F 6.568515 -2.203382 -0.742464
F 6.436871 -0.085147 -1.139036

H -0.844073 3.470489 5.506614
H 0.628325 3.516975 4.545798
H -3.834349 3.478817 -4.126373
H -2.313188 4.242509 -3.686162
H 3.146254 5.193894 0.635708
H 4.422297 5.041221 -0.566665

P5 Au⁺ C3
Au 0.364852 -1.637821 -1.971884
P -0.431692 -1.447999 0.193726
C -0.505220 0.284473 0.636793
C -2.138592 -2.181791 0.203169
C 0.509242 -2.706372 1.224929
S 0.868792 1.086060 1.311725
P -1.923466 1.271646 0.108171
H -2.797026 -1.438014 -0.260666
C -2.236481 -3.447683 -0.648284
C -2.658139 -2.449074 1.618934
H -0.333666 -3.364696 1.471309
C 1.468502 -3.565323 0.396668
C 1.121151 -2.272766 2.550892
O 0.768624 2.521529 1.086530
O 1.241854 0.595097 2.625606
C 2.309933 0.621561 0.212276
C -3.342926 0.885148 1.222752
C -2.222447 0.721230 -1.629815
C -1.695225 3.104775 0.069540
H -1.870516 -3.253890 -1.666007
H -1.588806 -4.231320 -0.225115
C -3.683098 -3.931552 -0.692525
C -4.115496 -2.892982 1.557930
H -2.079192 -3.256274 2.084977
H -2.528297 -1.571678 2.259275
H 0.973057 -3.908698 -0.521856
H 2.338672 -2.972192 0.090858
C 1.922273 -4.772308 1.212898
C 1.544779 -3.506486 3.340194
H 1.995530 -1.640217 2.375612
H 0.415143 -1.665581 3.126287
F 2.072268 1.120147 -1.004387
F 2.378202 -0.715477 0.117951
C 3.668031 1.122224 0.764689
H -3.614370 -0.149036 0.973964
C -2.937503 0.918251 2.703559
C -4.602403 1.733943 1.013378
H -1.940320 -0.340087 -1.602401
C -3.652121 0.737274 -2.167419
C -1.189079 1.354019 -2.571139
H -0.700150 3.211061 -0.378286
C -1.680679 3.802801 1.437271
C -2.696882 3.843151 -0.835103
H -4.285847 -3.184084 -1.231591
H -3.743348 -4.856300 -1.278403
C -4.263868 -4.140566 0.698940
H -4.486804 -3.074398 2.574058

H -4.733790 -2.079529 1.144606
H 1.058299 -5.431528 1.388925
H 2.640290 -5.354954 0.623269
C 2.521824 -4.361207 2.548823
H 1.988133 -3.186552 4.290501
H 0.656399 -4.104652 3.596582
F 4.067929 0.255653 1.700512
F 3.532446 2.328013 1.302519
C 4.740962 1.194769 -0.351029
H -2.008956 0.358300 2.869883
H -2.724134 1.951860 2.998067
C -4.073808 0.383052 3.568440
C -5.739610 1.179474 1.865432
H -4.400576 2.764792 1.327809
H -4.897271 1.776470 -0.039558
H -4.309466 0.171905 -1.492553
H -4.050481 1.758379 -2.211478
C -3.686130 0.113107 -3.558809
C -1.284361 0.721761 -3.954965
H -1.343161 2.434749 -2.662165
H -0.182950 1.217702 -2.148769
H -2.706150 3.839634 1.829330
H -1.063228 3.269417 2.161010
C -1.168548 5.226371 1.263572
C -2.205493 5.277093 -1.028724
H -2.812438 3.368765 -1.811449
H -3.689951 3.859877 -0.371839
H -5.317540 -4.434980 0.629102
H -3.741384 -4.975392 1.188813
H 3.444567 -3.788404 2.374465
H 2.810681 -5.249240 3.123506
F 4.527091 2.294103 -1.069929
F 4.620689 0.118278 -1.142647
C 6.188038 1.231630 0.194471
H -4.243798 -0.677937 3.331823
H -3.779806 0.423056 4.623990
C -5.363942 1.157174 3.339510
H -6.643123 1.779830 1.705369
H -5.980605 0.159058 1.528554
H -3.436760 -0.957217 -3.477232
H -4.705498 0.163144 -3.959116
C -2.701290 0.785059 -4.504438
H -0.573849 1.207440 -4.634192
H -0.976592 -0.338583 -3.890038
H -1.134038 5.725097 2.239503
H -0.132584 5.179814 0.898826
C -2.042499 6.009245 0.295222
H -1.240883 5.249739 -1.557344
H -2.904354 5.813481 -1.682322
F 6.999819 1.617998 -0.770629
F 6.264417 2.085931 1.201457
F 6.543694 0.029301 0.608639
H -6.177703 0.726803 3.935104
H -5.234468 2.190802 3.693118
H -2.746893 0.318850 -5.495609

H -2.988701 1.838091 -4.645052
H -3.035359 6.157851 0.746047
H -1.628771 7.010304 0.124618

4.6.4 Energies and coordinates of the gold complexes including solvent effects

Table 337: Energies of the investigated gold complexes optimized with the PCM model (aniline as solvent):.

	**E(SCF)**	**Corr(H)**	**Corr(G)**	**ΔG [kJ/mol]**
$^{Cy}Y_S$-PCy_2-AuCl (**P1**)	-3313.700907	1.001774	0.873184	–
$^{Cy}Y_S$-PCy_2-Au^+pa	-3165.136051	1.119082	0.97899	–
$^{Cy}Y_S$-PCy_2-Au^+pa C2	-3165.124537	1.119961	0.978683	+29.4233
$^{Cy}Y_S$-PCy_2-Au^+pa C3	-3165.129991	1.119617	0.98017	+19.0078
$^{Cy}Y_S$-PCy_2-Au^+pa C4	-3165.111034	1.119607	0.978349	+64.8047
$^{Cy}Y_S$-PCy_2-Au^+an	-3140.765404	1.127773	0.991020	–
$^{Cy}Y_{oTol}$-PCy_2-AuCl (**P2**)	-2764.857435	0.987043	0.866086	–
$^{Cy}Y_{oTol}$-PCy_2-Au^+pa	-2612.728815	1.104998	0.970929	–
$^{Cy}Y_{Mes}$-PCy_2-AuCl (**P3**)	-2843.476089	1.046349	0.919539	–
$^{Cy}Y_{Mes}$-PCy_2-Au^+pa	-2694.68109	1.163441	1.023528	–
$^{Cy}Y_{Mes}$-PCy_2-Au^+dpa	-2922.492311	1.251064	1.100099	–
$^{Cy}Y_{Mes}$-PCy_2-Au^+an	-2670.543244	1.172736	1.037604	–
$^{Cy}Y_{Mes}$-PCy_2-Au^+pa C2	-2694.669121	1.163924	1.02621	+38.4658
$^{Cy}Y_{SF}$-PCy_2-AuCl (**P5**)	-4093.953782	0.941691	0.803334	–
$^{Cy}Y_{SF}$-PCy_2-Au^+pa	-3946.291152	1.059144	0.909671	–
$^{Cy}Y_{SF}$-PCy_2-Au^+pa C2	-3946.263332	1.060184	0.908069	+68.8346
$^{Cy}Y_{SF}$-PCy_2-Au^+pa C3	-3946.251489	1.060045	0.91031	+105.8132
$^{Cy}Y_{SF}$-PCy_2-Au^+an	-3921.015244	1.067745	0.919273	–
^{Cy}JohnPhos-AuCl (**L6**)	-1874.774934	0.599553	0.510493	–
^{Cy}JohnPhos-Au^+pa	-1722.645514	0.716914	0.616243	–
^{Cy}JohnPhos-Au^+pa C2	-1724.72125814	0.716777	0.615128	+97.2361
^{Cy}JohnPhos-Au^+dpa	-1953.784843	0.804206	0.692263	–
$^{Ph}Y_S$-PCy_2-AuCl (**A**)	-3303.046996	0.787903	0.663316	–

$^{Ph}Y_S$-PCy2-Au+pa	-3150.920510	0.905782	0.771943	–
$^{Ph}Y_S$-PCy2 -Au+dpa	-3382.065553	0.992832	0.847670	–
$^{Ph}Y_S$-PCy2 -Au+an	-3130.110924	0.913489	0.778135	–

CyYS-PCy2-AuCl (P1):

Au -2.473573 -0.165873 -0.982247
Cl -3.895874 0.445272 -2.760067
P -1.104394 -0.810292 0.742966
C 0.494071 -0.005853 0.766001
C -1.981206 -0.630349 2.379485
C -0.899030 -2.639724 0.522948
S 1.535720 -0.527681 2.064559
P 0.958657 1.274022 -0.359966
H -1.741844 -1.569968 2.898638
C -3.495262 -0.532010 2.177351
C -1.508574 0.511283 3.276833
H -0.217296 -2.954490 1.322565
C -2.216031 -3.397309 0.689058
C -0.283989 -2.947338 -0.838277
O 0.796646 -1.587467 2.774941
O 2.046008 0.586267 2.880389
C 2.995228 -1.316658 1.408472
C -0.426324 2.399735 -0.807109
C 2.344940 2.225645 0.380613
C 1.591540 0.674186 -1.986545
H -3.716166 0.419989 1.668298
H -3.857738 -1.320565 1.506498
C -4.227177 -0.584161 3.512130
C -2.226052 0.452296 4.620720
H -0.428316 0.473324 3.429498
H -1.727984 1.476082 2.801017
H -2.644388 -3.220781 1.682572
H -2.945037 -3.020680 -0.047247
C -2.006853 -4.894959 0.488704
C -0.059594 -4.440765 -1.025131
H -0.967999 -2.586229 -1.622740
H 0.653751 -2.393457 -0.962791
C 4.224673 -0.673919 1.463303
C 2.884863 -2.587532 0.851996
H -1.048439 1.756816 -1.447795
C -1.331724 2.863380 0.329821
C 0.019472 3.574298 -1.681971
H 2.937278 1.447217 0.872733
C 3.293605 2.944686 -0.581674
C 1.856727 3.183758 1.469623
H 2.002955 1.574272 -2.466449
C 0.511064 0.102643 -2.902574
C 2.723829 -0.326552 -1.772193
H -5.308989 -0.497222 3.348160
H -4.057811 -1.569119 3.973597

C -3.738035 0.506502 4.454364
H -1.946552 -0.481094 5.132499
H -1.879916 1.274038 5.260883
H -1.361392 -5.273690 1.295504
H -2.967085 -5.417400 0.586051
C -1.363282 -5.207134 -0.854533
H 0.377335 -4.630919 -2.014196
H 0.673094 -4.797474 -0.284250
H 4.310493 0.286709 1.965984
C 5.333899 -1.276494 0.878809
C 3.997954 -3.178150 0.276972
H 1.930774 -3.107394 0.869875
H -1.654363 1.998793 0.917645
H -0.789903 3.529172 1.011364
C -2.546845 3.583423 -0.242442
C -1.206083 4.285176 -2.245068
H 0.606289 4.283286 -1.082177
H 0.663567 3.237706 -2.503392
H 3.667760 2.267982 -1.356883
H 2.773169 3.763769 -1.094842
C 4.477743 3.498539 0.207605
C 3.043174 3.706860 2.263673
H 1.341708 4.029907 0.993710
H 1.140893 2.680158 2.125939
H -0.002720 -0.717740 -2.385117
H -0.252769 0.857066 -3.125153
C 1.106647 -0.410282 -4.208549
C 3.307178 -0.818058 -3.087981
H 2.323043 -1.183388 -1.220638
H 3.511308 0.101111 -1.139463
H -4.018687 1.487994 4.042102
H -4.236143 0.423569 5.429079
H -1.192698 -6.286591 -0.955131
H -2.054187 -4.921650 -1.662278
H 6.296202 -0.768354 0.911539
C 5.236324 -2.522527 0.256849
H 3.907364 -4.168548 -0.166500
H -3.189640 3.929506 0.576779
H -3.138396 2.862252 -0.827215
C -2.139923 4.746680 -1.135827
H -0.887966 5.134240 -2.862849
H -1.744725 3.592960 -2.909665
H 5.153087 4.033530 -0.471837
H 5.051685 2.651919 0.615065
C 4.040393 4.404551 1.350190
H 3.519988 2.856345 2.772785
H 2.695776 4.390691 3.048329
H 0.309884 -0.850752 -4.820723
H 1.505122 0.442282 -4.779196
C 2.222578 -1.416478 -3.970275
H 3.788759 0.020324 -3.613823
H 4.095123 -1.554711 -2.882716
C 6.417898 -3.148102 -0.417879
H -3.028490 5.227829 -1.563401
H -1.630277 5.511021 -0.529171

H 3.572157 5.308849 0.932317
H 4.915567 4.742394 1.919657
H 2.643495 -1.753568 -4.925886
H 1.807874 -2.309696 -3.477435
H 6.314298 -3.086151 -1.509719
H 6.506192 -4.210566 -0.163978
H 7.350437 -2.645770 -0.142939

$^{Cy}Y_S$-PCy_2-Au(pa)$^+$:

Au -1.925673 -0.504048 -1.001806
P -0.454409 -0.936256 0.751813
C 1.043278 0.027697 0.746783
C -1.390418 -0.807491 2.361304
C -0.087326 -2.739138 0.544763
S 2.116926 -0.364997 2.069680
P 1.384258 1.336089 -0.388883
H -1.088351 -1.723755 2.889417
C -2.901276 -0.834229 2.118607
C -1.030794 0.371504 3.263718
H 0.614577 -2.981777 1.351893
C -1.329137 -3.615035 0.705129
C 0.567774 -2.994843 -0.808522
O 1.469281 -1.485364 2.776790
O 2.487131 0.810674 2.871205
C 3.657083 -1.002983 1.442483
C -0.120995 2.291209 -0.841424
C 2.660195 2.433605 0.344160
C 2.075023 0.781365 -2.006804
H -3.186710 0.092867 1.593203
H -3.179050 -1.660309 1.451853
C -3.667365 -0.928992 3.430576
C -1.783452 0.262779 4.585338
H 0.044345 0.414472 3.447907
H -1.309360 1.315691 2.778998
H -1.784729 -3.471760 1.691596
H -2.082435 -3.316624 -0.043400
C -0.976171 -5.087555 0.520054
C 0.936542 -4.461270 -0.978120
H -0.141208 -2.711393 -1.603836
H 1.445987 -2.351121 -0.931529
C 4.809332 -0.230213 1.506455
C 3.690688 -2.284944 0.901874
H -0.665951 1.573088 -1.473033
C -1.061986 2.646862 0.304382
C 0.169628 3.505817 -1.726888
H 3.321510 1.726596 0.856609
C 3.536951 3.217173 -0.636099
C 2.073666 3.362895 1.409734
H 2.406145 1.709724 -2.494428
C 1.051420 0.110150 -2.920239
C 3.288836 -0.116446 -1.778148
H -4.746379 -0.923978 3.229782
H -3.439604 -1.893758 3.908388
C -3.289301 0.205322 4.371698

H -1.452166 -0.644675 5.112012
H -1.518369 1.110961 5.229063
H -0.308159 -5.395653 1.337884
H -1.883614 -5.697546 0.611845
C -0.289373 -5.347663 -0.812857
H 1.400994 -4.617297 -1.960231
H 1.691684 -4.737111 -0.226044
H 4.785269 0.738778 1.999612
C 5.985044 -0.716722 0.945292
C 4.869643 -2.758424 0.350075
H 2.799545 -2.906866 0.916750
H -1.269168 1.755264 0.902584
H -0.597070 3.377238 0.975783
C -2.362257 3.210488 -0.251058
C -1.140191 4.068426 -2.270002
H 0.680447 4.279687 -1.138353
H 0.836579 3.242775 -2.557448
H 3.984782 2.559658 -1.388158
H 2.938107 3.962896 -1.175749
C 4.654439 3.911520 0.138435
C 3.199479 4.030215 2.185152
H 1.468775 4.136215 0.916093
H 1.419786 2.801860 2.084350
H 0.606084 -0.744873 -2.393005
H 0.233615 0.800906 -3.159170
C 1.693251 -0.368991 -4.217634
C 3.914776 -0.574319 -3.086984
H 2.962308 -0.996212 -1.213407
H 4.035542 0.385888 -1.150609
H -3.627826 1.158809 3.937706
H -3.810445 0.096439 5.331293
H -0.014020 -6.406059 -0.899869
H -0.995321 -5.139276 -1.631465
H 6.886857 -0.108262 0.986192
C 6.030382 -1.973293 0.338088
H 4.893985 -3.758586 -0.079514
H -3.036376 3.472006 0.573923
H -2.868006 2.421442 -0.828581
C -2.112794 4.411550 -1.150329
H -0.933085 4.951525 -2.886557
H -1.598572 3.320744 -2.935754
H 5.274717 4.491329 -0.556232
H 5.309056 3.139800 0.571731
C 4.121698 4.801124 1.251945
H 3.761262 3.249161 2.718471
H 2.780566 4.695542 2.950271
H 0.939748 -0.881959 -4.828601
H 2.021097 0.507584 -4.796290
C 2.888224 -1.274695 -3.963260
H 4.323535 0.295030 -3.623207
H 4.762393 -1.237580 -2.870848
C 7.280888 -2.474696 -0.314769
H -3.057970 4.779711 -1.568669
H -1.696401 5.233031 -0.548533
H 3.564305 5.639612 0.807448

H 4.954691 5.243732 1.812382
H 3.339133 -1.585699 -4.913658
H 2.549103 -2.194208 -3.461297
H 7.202902 -2.386960 -1.407132
H 7.452657 -3.532892 -0.088561
H 8.158041 -1.902674 0.002619
C -3.991686 -0.117437 -2.066248
C -3.099374 -0.316622 -2.895038
C -5.064773 0.142921 -1.161177
C -5.625658 1.427016 -1.094740
C -5.541373 -0.879101 -0.325796
C -6.652726 1.679563 -0.197622
H -5.251446 2.213440 -1.745217
C -6.566225 -0.611918 0.568178
H -5.096372 -1.869915 -0.382714
C -7.119599 0.665102 0.634402
H -7.090022 2.673805 -0.145050
H -6.935205 -1.401886 1.218169
H -7.922105 0.870523 1.339592
H -2.570230 -0.481031 -3.818639

$^{Cy}Y_S$-PCy_2-Au(pa)$^+$ C2:

P 0.198038 -1.131306 -0.024121
C 0.571666 2.531725 -1.265063
C 0.351221 3.777902 -1.841344
C -0.943527 0.192858 -0.420479
C 0.931351 -1.310950 1.688249
C -0.595711 -2.768850 -0.348885
S -0.499037 1.185427 -1.775335
C 1.605050 2.353767 -0.353809
H -0.469769 3.908824 -2.541272
C 1.172912 4.844251 -1.495026
P -2.505764 0.589454 0.330229
H 1.568031 -2.182626 1.466223
C 0.012250 -1.717678 2.836801
C 1.891042 -0.195908 2.099537
H -1.439444 -2.874092 0.343651
C 0.352514 -3.941643 -0.096859
C -1.120406 -2.791005 -1.783842
O -1.700789 1.865778 -2.285150
O 0.258975 0.400375 -2.773791
C 2.412494 3.428087 -0.010563
H 1.761678 1.387442 0.112659
H 0.993296 5.820128 -1.942807
C 2.215128 4.691922 -0.577777
C -2.943037 -0.818773 1.437537
C -3.899791 0.921322 -0.823938
C -2.386327 2.114979 1.368425
H -0.628030 -2.559236 2.538607
H -0.649431 -0.881851 3.098820
C 0.811657 -2.091122 4.079261
C 2.653302 -0.563062 3.368280
H 1.338115 0.737637 2.261568

H 2.612696 -0.014353 1.292622
H 1.260649 -3.804317 -0.706246
H 0.673780 -3.968091 0.951109
C -0.315521 -5.267769 -0.445232
C -1.795467 -4.115726 -2.105822
H -1.803985 -1.950586 -1.951043
H -0.271907 -2.630235 -2.468413
H 3.204039 3.285180 0.723566
C 3.100683 5.841153 -0.207331
H -1.969825 -1.249483 1.684169
C -3.604895 -0.422207 2.760930
C -3.751937 -1.916543 0.738250
H -3.588567 1.869847 -1.273246
C -5.219887 1.177942 -0.089805
C -4.067563 -0.048660 -1.992501
H -3.239947 2.067292 2.059550
C -1.086565 2.077229 2.168834
C -2.509379 3.406647 0.553216
H 1.419008 -2.983266 3.865277
H 0.121998 -2.366096 4.887051
C 1.719861 -0.948415 4.504012
H 3.320321 -1.409098 3.147879
H 3.301328 0.273704 3.657564
H -1.147372 -5.440010 0.254658
H 0.396805 -6.087465 -0.288762
C -0.849533 -5.283155 -1.869089
H -2.152364 -4.107014 -3.143136
H -2.686716 -4.232580 -1.469053
H 3.214867 5.918626 0.880185
H 2.704808 6.789800 -0.582061
H 4.106179 5.707278 -0.627471
H -4.578154 0.048892 2.562392
H -2.997960 0.316890 3.295372
C -3.793688 -1.645426 3.653457
C -3.885141 -3.133765 1.643836
H -4.755516 -1.544778 0.503142
H -3.294779 -2.191354 -0.217267
H -5.607097 0.252432 0.354648
H -5.084062 1.889910 0.734746
C -6.244263 1.723074 -1.080213
C -5.091117 0.519070 -2.967941
H -3.102622 -0.188918 -2.489953
H -4.413047 -1.028657 -1.645990
H -0.256816 1.995299 1.456922
H -1.046551 1.177251 2.796393
C -0.882737 3.324518 3.014819
C -2.282282 4.637843 1.420512
H -3.498641 3.469039 0.086094
H -1.778231 3.397894 -0.262898
H 1.104520 -0.079773 4.784931
H 2.295717 -1.225852 5.395563
H -1.352370 -6.235843 -2.076882
H -0.007432 -5.212098 -2.574095
H -2.803690 -2.020168 3.954656
H -4.303703 -1.343772 4.576247

C -4.556441 -2.760404 2.955817
H -2.886611 -3.552178 1.851230
H -4.448151 -3.918315 1.123533
H -5.907704 2.710351 -1.430393
H -7.202622 1.880716 -0.570043
C -6.419049 0.796104 -2.275642
H -5.231412 -0.176282 -3.804865
H -4.694567 1.451846 -3.395355
H -1.654558 3.375620 3.797087
H 0.084104 3.256050 3.529697
C -0.949838 4.572041 2.148727
H -2.338114 5.534784 0.791323
H -3.095547 4.719926 2.157285
H -5.585464 -2.428967 2.751981
H -4.633566 -3.635858 3.611927
H -7.137721 1.226317 -2.984372
H -6.851498 -0.156159 -1.932230
H -0.135815 4.541789 1.407525
H -0.796156 5.473338 2.754916
Au 2.074199 -1.007365 -1.412617
C 4.354067 -0.803834 -1.621393
C 3.852457 -1.030081 -2.728192
C 5.001293 -0.476682 -0.388276
C 5.403565 -1.486685 0.496509
C 5.179223 0.871775 -0.047056
C 5.981513 -1.143618 1.709663
H 5.254498 -2.529319 0.226793
C 5.751645 1.200837 1.172998
H 4.858051 1.646576 -0.738789
C 6.150563 0.196216 2.051180
H 6.295357 -1.925339 2.397516
H 5.888036 2.246575 1.439915
H 6.596403 0.458846 3.008143
H 3.738430 -1.204331 -3.785137

$^{Cy}Y_S$-PCy$_2$-Au(pa)$^+$ C3:

P 0.013516 -1.056599 0.312203
C -1.247506 1.622200 -1.614469
C -2.123672 1.594798 -2.695895
C 0.996215 0.262358 -0.374071
C 0.563820 -2.808009 0.046623
C -0.200731 -0.874283 2.139664
S 0.373672 0.903319 -1.857920
C -1.622700 2.223227 -0.417863
H -1.822636 1.112506 -3.622237
C -3.391367 2.145722 -2.557203
P 2.666340 0.774703 -0.049771
H -0.301555 -3.346789 0.465076
C 1.800114 -3.286453 0.802319
C 0.617704 -3.152758 -1.441032
H 0.788023 -0.996239 2.599818
C -1.109051 -1.968596 2.706743
C -0.737690 0.511504 2.489877
O 0.158661 -0.151562 -2.867115

O 1.271763 2.013114 -2.236095
C -2.896765 2.754842 -0.290167
H -0.939107 2.233303 0.422440
H -4.082109 2.107672 -3.397821
C -3.805737 2.719924 -1.352900
C 3.162889 -0.140587 1.486551
C 2.904856 2.585139 0.155031
C 3.904442 0.312725 -1.353695
H 1.704105 -3.072436 1.876115
H 2.689199 -2.749390 0.448634
C 2.029537 -4.775844 0.577203
C 0.888674 -4.636696 -1.651105
H 1.394946 -2.555889 -1.929013
H -0.326708 -2.860563 -1.920268
H -2.081756 -1.933113 2.189596
H -0.688490 -2.962484 2.514868
C -1.316838 -1.789453 4.206537
C -0.922081 0.673143 3.991589
H -0.072108 1.287661 2.093350
H -1.705078 0.654428 1.986072
H -3.200046 3.189422 0.661062
C -5.180065 3.289388 -1.192300
H 2.514832 -1.022056 1.460563
C 4.602595 -0.662856 1.498973
C 2.844027 0.611040 2.783693
H 2.799214 2.926199 -0.881958
C 4.318588 2.949825 0.619866
C 1.823840 3.309706 0.947958
H 4.836472 0.238977 -0.775493
C 3.576157 -1.058517 -1.933312
C 4.173725 1.310545 -2.483022
H 1.192323 -5.341475 1.012610
H 2.934563 -5.093043 1.110073
C 2.137305 -5.089782 -0.908145
H 0.025378 -5.216741 -1.291063
H 0.980052 -4.844818 -2.724401
H -0.358109 -1.949330 4.722175
H -2.000279 -2.564832 4.573868
C -1.841412 -0.403404 4.547911
H -1.314078 1.674018 4.210659
H 0.059349 0.605389 4.486535
H -5.163067 4.381339 -1.309908
H -5.578304 3.075410 -0.194001
H -5.872835 2.884240 -1.936386
H 5.306517 0.182024 1.472720
H 4.800645 -1.272035 0.609985
C 4.858100 -1.513449 2.738519
C 3.103908 -0.264417 4.003494
H 3.468793 1.507677 2.859550
H 1.807980 0.957956 2.783465
H 4.484287 2.625984 1.654529
H 5.077307 2.442473 0.010683
C 4.508556 4.460788 0.537511
C 2.041259 4.815484 0.865684
H 0.847314 3.044522 0.531910

H 1.831030 2.998420 1.999863
H 2.642728 -0.963944 -2.505211
H 3.375699 -1.780655 -1.133067
C 4.692803 -1.569534 -2.831769
C 5.316297 0.801079 -3.355652
H 4.428463 2.299930 -2.087516
H 3.269970 1.433667 -3.086231
H 3.015600 -4.571637 -1.324785
H 2.306195 -6.162885 -1.061040
H -1.948944 -0.293355 5.633940
H -2.848488 -0.277620 4.120105
H 4.235073 -2.419353 2.678833
H 5.900805 -1.853110 2.734237
C 4.537093 -0.768809 4.023388
H 2.416463 -1.125411 3.997425
H 2.876001 0.305164 4.913000
H 4.461136 4.769555 -0.517422
H 5.510082 4.726104 0.897926
C 3.439284 5.201481 1.328867
H 1.279255 5.333416 1.461304
H 1.900323 5.138489 -0.176234
H 5.594196 -1.742397 -2.224194
H 4.407542 -2.541913 -3.253478
C 5.008476 -0.571812 -3.935420
H 5.507628 1.522901 -4.159472
H 6.239146 0.747080 -2.757769
H 5.220656 0.086394 4.131112
H 4.704705 -1.416166 4.892694
H 3.584466 6.285223 1.239411
H 3.547126 4.959008 2.397176
H 4.140633 -0.491428 -4.606672
H 5.847156 -0.928783 -4.546265
Au -2.106974 -1.058126 -0.671725
C -3.866465 -1.520684 -1.955568
C -4.392691 -0.899335 -1.028111
C -5.248497 -0.219721 -0.101489
C -6.579987 0.012330 -0.475912
C -4.794854 0.203953 1.153012
C -7.439522 0.655508 0.403553
H -6.931575 -0.314732 -1.451118
C -5.660724 0.850758 2.021949
H -3.761507 0.023254 1.436339
C -6.984118 1.075465 1.650453
H -8.472258 0.831720 0.111594
H -5.300294 1.176438 2.995320
H -7.662599 1.580104 2.334829
H -3.617260 -2.076138 -2.843446

$^{Cy}Y_S$-PCy_2-Au(an)$^+$:

Au 2.196008 0.896668 -0.369218
P 0.165476 1.596554 0.472495
C -1.081926 0.339648 0.684757
C 0.466578 2.562537 2.038713
C -0.389600 2.859408 -0.764925

S -2.592812 0.958337 1.310659
P -0.818538 -1.382515 0.404396
H -0.166059 3.451484 1.901562
C 1.930953 2.993624 2.151260
C 0.027654 1.894275 3.340117
H -1.361289 3.207730 -0.393525
C 0.548841 4.062468 -0.846107
C -0.556298 2.223284 -2.140918
O -2.408984 2.417738 1.411672
O -3.039949 0.254935 2.522087
C -3.895456 0.711070 0.121101
C 0.880291 -1.917029 0.854928
C -2.077475 -2.331968 1.343636
C -1.000379 -1.916624 -1.351935
H 2.545011 2.091427 2.307332
H 2.285169 3.446225 1.216548
C 2.125017 3.955797 3.316246
C 0.199827 2.863220 4.504596
H -1.009282 1.557386 3.282839
H 0.641793 1.004154 3.529869
H 0.636933 4.554471 0.129245
H 1.558833 3.717520 -1.124794
C 0.046115 5.069177 -1.875939
C -1.073791 3.228213 -3.160007
H 0.423392 1.844438 -2.475501
H -1.220434 1.353825 -2.076949
C -4.877026 -0.246194 0.341181
C -3.920796 1.504178 -1.021987
H 1.485945 -1.442584 0.065525
C 1.434259 -1.404141 2.179517
C 1.098500 -3.425442 0.714420
H -2.977072 -1.716360 1.235365
C -2.435830 -3.720662 0.809317
C -1.743544 -2.396086 2.835693
H -1.006423 -3.015160 -1.301402
C 0.167854 -1.497939 -2.241314
C -2.337106 -1.447136 -1.919211
H 3.182079 4.241255 3.392275
H 1.564604 4.880192 3.110170
C 1.637715 3.347873 4.623554
H -0.467655 3.724589 4.352781
H -0.121137 2.380475 5.436294
H -0.911379 5.482971 -1.526609
H 0.745512 5.912351 -1.938457
C -0.151873 4.435902 -3.245420
H -1.176085 2.745059 -4.140288
H -2.082175 3.559160 -2.867237
H -4.887540 -0.807559 1.272558
C -5.843589 -0.463166 -0.635206
C -4.890711 1.279885 -1.984497
H -3.185466 2.293217 -1.153091
H 1.265980 -0.326725 2.263935
H 0.913606 -1.872941 3.021884
C 2.924488 -1.707266 2.260876
C 2.590961 -3.719764 0.787030

H 0.575072 -3.951123 1.524572
H 0.690438 -3.804780 -0.230296
H -2.701900 -3.684746 -0.252242
H -1.579584 -4.401576 0.904660
C -3.623920 -4.267306 1.597569
C -2.946517 -2.915236 3.607462
H -0.898645 -3.083608 2.981982
H -1.443320 -1.409094 3.200588
H 0.289508 -0.406494 -2.192990
H 1.101846 -1.939234 -1.875042
C -0.050575 -1.926606 -3.687512
C -2.531332 -1.898099 -3.359195
H -2.363049 -0.352888 -1.882424
H -3.170102 -1.791521 -1.293596
H 2.283366 2.495331 4.884802
H 1.733274 4.073256 5.441490
H -0.548648 5.173971 -3.953591
H 0.823802 4.114970 -3.641666
H -6.608833 -1.219238 -0.468148
C -5.854269 0.275697 -1.820052
H -4.905586 1.896261 -2.881938
H 3.323890 -1.353887 3.219612
H 3.441849 -1.133122 1.475958
C 3.210684 -3.190083 2.071915
H 2.763435 -4.798869 0.692025
H 3.078145 -3.246583 -0.077501
H -3.871286 -5.272907 1.235446
H -4.500868 -3.635721 1.387906
C -3.366521 -4.286268 3.097557
H -3.769096 -2.194422 3.490123
H -2.712951 -2.957076 4.678602
H 0.782255 -1.566526 -4.305758
H -0.025447 -3.025427 -3.740626
C -1.379817 -1.429621 -4.235646
H -2.597925 -2.995833 -3.395232
H -3.488394 -1.513086 -3.734297
C -6.862936 0.005692 -2.892991
H 4.293514 -3.369893 2.068947
H 2.802816 -3.747772 2.928244
H -2.567532 -5.010834 3.316828
H -4.259768 -4.638394 3.628656
H -1.517346 -1.770753 -5.269099
H -1.372652 -0.328883 -4.266707
H -6.403924 -0.558997 -3.716027
H -7.250992 0.937351 -3.319483
H -7.704601 -0.582946 -2.515416
H 4.223141 1.062469 -2.047622
H 4.853720 0.894483 -0.556345
C 4.482480 -0.873266 -1.489605
C 5.433590 -1.552629 -0.735529
C 3.816609 -1.517238 -2.529058
C 5.729024 -2.878867 -1.036307
H 5.950538 -1.045309 0.077403
C 4.117519 -2.841532 -2.822748
H 3.069846 -0.977193 -3.108513

C 5.076180 -3.525702 -2.080021
H 6.477597 -3.405375 -0.448397
H 3.597870 -3.339268 -3.638825
H 5.310157 -4.561965 -2.312220
N 4.169167 0.500293 -1.198831

$^{Cy}Y_{oTol}$-PCy_2-AuCl (P2):

Au -1.871726 -1.485019 0.275803
Cl -2.677277 -3.665971 0.691190
P -1.136678 0.638887 -0.181524
C 0.600175 0.815575 -0.477995
C -1.548163 1.806943 1.216069
C -2.153569 1.188517 -1.629970
P 1.692408 -0.372923 0.171821
C 1.129208 2.183757 -0.800983
H -0.604300 1.751595 1.780921
C -1.739484 3.274436 0.822639
C -2.649337 1.350551 2.171315
H -1.775426 2.180392 -1.928617
C -2.027973 0.208957 -2.798013
C -3.635702 1.296214 -1.259203
C 3.394161 0.197341 -0.260205
C 1.780376 -0.640764 2.012311
C 1.379590 -2.071755 -0.484163
C 1.551036 3.044135 0.229636
C 1.237657 2.665523 -2.126100
H -0.966542 3.599217 0.118894
H -2.701609 3.402300 0.309972
C -1.730062 4.150642 2.069164
C -2.665436 2.234826 3.413178
H -3.626843 1.391785 1.670007
H -2.494331 0.300213 2.453544
H -0.983808 0.059169 -3.075784
H -2.401239 -0.771077 -2.459479
C -2.837397 0.669325 -4.002753
C -4.472290 1.759006 -2.446482
H -3.978182 0.301573 -0.929900
H -3.794776 1.971538 -0.414942
H 3.343780 1.265957 -0.004104
C 3.667144 0.110276 -1.762452
C 4.566953 -0.392834 0.527138
H 2.615802 -1.348242 2.129795
C 2.126948 0.643305 2.765418
C 0.545427 -1.276208 2.647540
H 0.522905 -2.416053 0.114838
C 2.512840 -3.070536 -0.245625
C 0.920032 -2.095503 -1.939023
H 1.471473 2.694948 1.254295
C 2.047137 4.317907 -0.003755
C 1.730962 3.957759 -2.348545
C 0.874597 1.841432 -3.323559
H -0.738368 4.084578 2.543989
H -1.873360 5.203010 1.791549

C -2.801425 3.709998 3.058211
H -1.724788 2.080537 3.965375
H -3.475547 1.925755 4.086320
H -2.434673 1.628884 -4.363956
H -2.720112 -0.047260 -4.825991
C -4.306576 0.838436 -3.646055
H -5.528012 1.821554 -2.152461
H -4.163953 2.778863 -2.723092
H 3.783928 -0.944911 -2.048209
H 2.815924 0.499612 -2.326635
C 4.940045 0.865595 -2.116366
C 5.851114 0.350042 0.167429
H 4.404395 -0.321351 1.607435
H 4.685024 -1.458704 0.294805
H 2.983332 1.160974 2.316212
H 1.270279 1.326675 2.685031
C 2.382807 0.366247 4.241061
C 0.774658 -1.554574 4.128888
H 0.264147 -2.206290 2.141228
H -0.307460 -0.589838 2.536697
H 3.380767 -2.794930 -0.860853
H 2.845329 -3.056697 0.799853
C 2.058500 -4.474121 -0.630882
C 0.479379 -3.500917 -2.325502
H 1.732146 -1.758237 -2.598185
H 0.094730 -1.389801 -2.069320
H 2.358810 4.942091 0.831720
C 2.131807 4.785538 -1.310788
H 1.813392 4.311203 -3.376096
H 1.565430 2.039327 -4.151096
H -0.133721 2.080449 -3.686267
H 0.896033 0.774263 -3.086051
H -3.791843 3.880734 2.609043
H -2.762391 4.325400 3.966451
H -4.730584 -0.147651 -3.403198
H -4.872900 1.220032 -4.505473
H 5.128531 0.795827 -3.195262
H 4.794681 1.933293 -1.890297
C 6.127021 0.329812 -1.329340
H 5.760537 1.393731 0.504955
H 6.694114 -0.084376 0.719615
H 3.260888 -0.289937 4.339395
H 2.631519 1.302260 4.757124
C 1.174022 -0.296524 4.884669
H -0.132298 -1.994505 4.562526
H 1.569022 -2.309382 4.230905
H 2.878314 -5.186240 -0.471802
H 1.239835 -4.780613 0.037417
C 1.577634 -4.524688 -2.074304
H -0.408588 -3.762474 -1.729280
H 0.168196 -3.518292 -3.378002
H 2.514874 5.782415 -1.521158
H 7.033005 0.906006 -1.556620
H 6.327685 -0.705520 -1.644557
H 0.330685 0.411587 4.875576

H 1.375566 -0.528319 5.938184
H 1.224841 -5.533900 -2.322310
H 2.426953 -4.316587 -2.743422

$^{Cy}Y_{oTol}$-PCy$_2$-Au(pa)$^+$:

Au -1.702663 0.238520 1.043459
P -0.061243 1.272756 -0.242567
C 1.316993 0.281794 -0.720843
C 0.603205 2.739620 0.698092
C -1.004474 1.945699 -1.688614
P 1.721707 -1.147325 0.188795
C 2.386933 0.935507 -1.552406
H 1.485524 2.274111 1.164005
C 1.116820 3.897985 -0.162341
C -0.273066 3.248058 1.840939
H -0.256891 2.414526 -2.349716
C -1.735610 0.833718 -2.440980
C -2.034915 2.994341 -1.259048
C 3.251236 -1.844943 -0.568548
C 2.155145 -0.989531 1.991028
C 0.343219 -2.374148 0.189883
C 3.462195 1.592399 -0.926876
C 2.377086 0.911822 -2.965865
H 1.735336 3.530539 -0.987520
H 0.271961 4.430364 -0.617624
C 1.904562 4.876096 0.701397
C 0.502362 4.243943 2.695713
H -1.180271 3.723237 1.441532
H -0.613466 2.404957 2.459011
H -1.045831 0.045650 -2.742521
H -2.456452 0.366243 -1.749981
C -2.478138 1.373124 -3.655490
C -2.783661 3.557088 -2.462009
H -2.751150 2.513979 -0.570747
H -1.574166 3.811711 -0.699329
H 3.867029 -0.944361 -0.712042
C 2.989373 -2.441591 -1.951885
C 4.068296 -2.820631 0.282401
H 2.481823 -2.002526 2.270795
C 3.320130 -0.026303 2.214717
C 0.999225 -0.609813 2.913015
H -0.369400 -1.924335 0.899442
C 0.687729 -3.757636 0.741278
C -0.393419 -2.477331 -1.141749
H 3.485360 1.624499 0.157714
C 4.484501 2.207711 -1.633231
C 3.411998 1.543603 -3.667604
C 1.317972 0.216506 -3.765997
H 2.800724 4.368367 1.090913
H 2.262325 5.712226 0.087318
C 1.062290 5.388741 1.861685
H 1.332682 3.712167 3.186504
H -0.138889 4.630396 3.498186

H -1.747588 1.781562 -4.370821
H -2.991903 0.550961 -4.169893
C -3.466530 2.460919 -3.264875
H -3.515966 4.301584 -2.124779
H -2.069145 4.091278 -3.105908
H 2.420295 -3.375222 -1.837618
H 2.374818 -1.763869 -2.549844
C 4.302553 -2.745373 -2.658469
C 5.379178 -3.150307 -0.426716
H 4.295526 -2.402650 1.268202
H 3.498835 -3.744007 0.451500
H 4.159951 -0.244896 1.543978
H 2.976837 0.987268 1.966300
C 3.776299 -0.036704 3.667509
C 1.438790 -0.602032 4.372996
H 0.152643 -1.296383 2.792356
H 0.641157 0.395900 2.642290
H 1.358844 -4.275694 0.041778
H 1.217555 -3.687568 1.699390
C -0.588556 -4.576929 0.908298
C -1.659419 -3.304658 -0.977281
H 0.256022 -2.932266 -1.901058
H -0.639859 -1.473466 -1.496696
H 5.294039 2.701869 -1.099571
C 4.456707 2.188366 -3.023385
H 3.394808 1.510961 -4.756592
H 1.754061 -0.253310 -4.654736
H 0.555038 0.921489 -4.122151
H 0.812999 -0.549151 -3.171611
H 0.227147 5.983492 1.461434
H 1.651215 6.065842 2.493377
H -4.268224 2.015747 -2.656400
H -3.950563 2.881392 -4.155322
H 4.101941 -3.170697 -3.649852
H 4.844482 -1.801608 -2.823990
C 5.156577 -3.693907 -1.830399
H 5.985862 -2.234099 -0.485346
H 5.951911 -3.865380 0.177008
H 4.160100 -1.036322 3.920633
H 4.611321 0.662648 3.798468
C 2.626704 0.320516 4.597558
H 0.594434 -0.306820 5.009100
H 1.710528 -1.626673 4.667584
H -0.341712 -5.575376 1.290165
H -1.220939 -4.096544 1.671264
C -1.363070 -4.680431 -0.398244
H -2.343524 -2.764385 -0.305746
H -2.178302 -3.392597 -1.940305
H 5.246322 2.664381 -3.601647
H 6.119531 -3.876851 -2.323579
H 4.648865 -4.668251 -1.764512
H 2.318090 1.359690 4.403302
H 2.949997 0.280866 5.645137
H -2.294418 -5.240623 -0.245196
H -0.768343 -5.256106 -1.123421

C -3.724758 -0.781443 1.682619
C -3.027771 -0.537020 2.670399
C -4.605089 -1.150019 0.619101
C -5.023646 -2.483455 0.503218
C -5.043700 -0.191884 -0.306242
C -5.873113 -2.847286 -0.531007
H -4.676561 -3.221681 1.221856
C -5.889302 -0.569708 -1.337991
H -4.710911 0.839155 -0.207530
C -6.302866 -1.894968 -1.451774
H -6.199241 -3.880763 -0.621559
H -6.229552 0.172385 -2.056535
H -6.965661 -2.187005 -2.263471
H -2.667461 -0.401553 3.676053

$^{Cy}Y_{Mes}$-PCy$_2$-AuCl (P3):

Au 2.353714 -0.879336 -0.531922
Cl 3.816935 -2.626260 -1.154790
P 0.981551 0.846457 0.103832
C -0.768552 0.545432 0.086685
C 1.594793 1.379501 1.773961
C 1.266531 2.328763 -0.986112
P -1.434634 -1.052515 -0.004602
C -1.627058 1.782254 0.158176
H 0.988721 2.256455 2.051129
C 3.070477 1.779690 1.761875
C 1.399303 0.272378 2.803152
H 0.472514 2.193003 -1.727250
C 1.045420 3.690462 -0.318501
C 2.582497 2.310555 -1.766458
C -3.261074 -1.044201 -0.205223
C -1.219124 -2.112770 1.536665
C -0.716311 -2.098447 -1.356902
C -2.003328 2.496206 -1.007609
C -1.985094 2.351916 1.407981
H 3.267939 2.569645 1.031010
H 3.663675 0.907028 1.443586
C 3.524102 2.235540 3.144898
C 1.814181 0.724008 4.195367
H 0.364144 -0.081979 2.794586
H 2.026865 -0.582141 2.504636
H 0.104165 3.710662 0.241834
H 1.848011 3.881801 0.407020
C 1.055609 4.798186 -1.364970
C 2.603392 3.432924 -2.798309
H 3.435761 2.420615 -1.082203
H 2.710520 1.339169 -2.261481
H -3.472547 -2.125396 -0.186928
C -3.808952 -0.506967 -1.523521
C -4.020824 -0.404285 0.961187
H -1.258657 -1.347119 2.325362
C 0.159494 -2.767398 1.604612
C -2.281097 -3.160760 1.895493

H 0.244663 -2.420918 -0.928342
C -1.521852 -3.364727 -1.649983
C -0.375794 -1.362484 -2.649944
C -2.621787 3.744720 -0.899553
C -1.776222 1.994678 -2.406070
C -2.598560 3.601323 1.468352
C -1.768292 1.637805 2.709584
H 4.588837 2.501486 3.118135
H 2.979546 3.154121 3.412198
C 3.268893 1.172861 4.204546
H 1.656885 -0.088762 4.916903
H 1.168930 1.557142 4.515625
H 0.200845 4.652484 -2.044154
H 0.903452 5.771623 -0.880479
C 2.351547 4.793521 -2.163157
H 1.822271 3.236704 -3.549340
H 3.560436 3.429683 -3.336015
H -3.689914 0.580682 -1.536978
H -3.255268 -0.905680 -2.380640
C -5.290138 -0.847241 -1.644240
C -5.502729 -0.745754 0.862620
H -3.618684 -0.719930 1.930613
H -3.894874 0.683465 0.911155
H 0.195588 -3.600805 0.886918
H 0.942492 -2.063844 1.296502
C 0.443774 -3.303446 2.999815
C -2.017449 -3.703609 3.298219
H -3.295557 -2.754108 1.855389
H -2.248084 -3.994136 1.181264
H -1.756903 -3.902422 -0.725069
H -2.483150 -3.092555 -2.110657
C -0.748014 -4.277955 -2.594806
C 0.409520 -2.278953 -3.579206
H 0.205938 -0.460533 -2.424570
H -1.294885 -1.034453 -3.154187
H -2.884608 4.273855 -1.816346
C -2.905431 4.335432 0.324906
H -2.722597 1.953063 -2.959200
H -1.331501 1.000605 -2.407043
H -1.120000 2.670800 -2.969216
H -2.854100 4.010417 2.446407
H -0.831323 1.934298 3.196511
H -1.732058 0.556962 2.568625
H -2.578535 1.869380 3.409922
H 3.553432 1.545021 5.197260
H 3.910282 0.302001 4.000621
H 2.333569 5.576758 -2.932267
H 3.187677 5.036883 -1.489489
H -5.684970 -0.458007 -2.591296
H -5.406545 -1.941270 -1.682185
C -6.083556 -0.290987 -0.469550
H -5.630660 -1.834563 0.968686
H -6.047509 -0.284228 1.695928
H 1.438754 -3.765787 3.024908
H 0.470058 -2.461954 3.709342

C -0.623569 -4.300349 3.426991
H -2.131795 -2.882319 4.022182
H -2.780035 -4.450880 3.552564
H -1.344836 -5.171631 -2.817820
H 0.162021 -4.626272 -2.083799
C -0.359988 -3.557772 -3.877974
H 0.651365 -1.747590 -4.508717
H 1.368312 -2.527935 -3.098917
C -3.515809 5.701331 0.416653
H -7.138810 -0.582346 -0.547402
H -6.057928 0.808495 -0.511063
H -0.446298 -4.639330 4.455691
H -0.554939 -5.193168 2.787227
H 0.232299 -4.220674 -4.521514
H -1.272700 -3.307913 -4.440883
H -2.745681 6.461071 0.608916
H -4.239568 5.763708 1.237418
H -4.023111 5.978096 -0.513668

$^{Cy}Y_{Mes}$-PCy_2-Au(pa)$^+$:

Au 1.822795 -1.105441 0.441262
P 0.452481 0.759584 0.592002
C -1.197611 0.609674 -0.020215
C 0.549901 1.267032 2.372768
C 1.255006 2.154858 -0.344897
P -1.951299 -0.920237 -0.337247
C -1.901871 1.926806 -0.231718
H -0.019061 2.207487 2.440719
C 1.984144 1.518166 2.840373
C -0.082036 0.217113 3.278840
H 0.733342 2.079025 -1.304020
C 0.971270 3.549635 0.225425
C 2.743510 1.972182 -0.654648
C -3.596576 -0.742751 -1.130185
C -2.353549 -1.947354 1.186684
C -0.929029 -2.066180 -1.378382
C -1.796416 2.639207 -1.452319
C -2.591828 2.561839 0.833928
H 2.490623 2.258109 2.213628
H 2.553142 0.579152 2.735406
C 2.009070 1.973499 4.295899
C -0.097001 0.672756 4.730440
H -1.088702 -0.035871 2.933313
H 0.517861 -0.704122 3.202914
H -0.096680 3.680875 0.432001
H 1.495158 3.675323 1.182328
C 1.453821 4.623506 -0.741260
C 3.235890 3.072733 -1.588015
H 3.336718 1.974619 0.271338
H 2.912049 0.994894 -1.125240
H -3.909212 -1.798117 -1.180386
C -3.628235 -0.200724 -2.555998
C -4.626729 0.003583 -0.276195
H -2.570769 -1.157427 1.920262

C -1.146228 -2.728555 1.702640
C -3.575258 -2.875766 1.174345
H -0.205339 -2.471266 -0.654615
C -1.720422 -3.255678 -1.925823
C -0.106406 -1.406796 -2.482448
C -2.291250 3.942496 -1.549405
C -1.164596 2.082189 -2.697028
C -3.065519 3.863665 0.692469
C -2.884662 1.870802 2.133227
H 3.046465 2.131777 4.617457
H 1.507408 2.950162 4.369037
C 1.313673 0.978505 5.214164
H -0.563356 -0.096232 5.360017
H -0.722649 1.574144 4.820962
H 0.872318 4.551860 -1.673804
H 1.249154 5.617591 -0.323564
C 2.935578 4.462929 -1.046835
H 2.741174 2.952465 -2.564338
H 4.311423 2.949683 -1.767324
H -3.405568 0.870478 -2.531531
H -2.867771 -0.677326 -3.184361
C -5.015815 -0.409898 -3.152692
C -6.021407 -0.208495 -0.851904
H -4.592331 -0.312084 0.772853
H -4.388603 1.073209 -0.288509
H -0.961909 -3.583309 1.034258
H -0.239368 -2.109642 1.673683
C -1.390014 -3.245540 3.113048
C -3.841332 -3.394683 2.585248
H -4.472218 -2.372257 0.803136
H -3.396447 -3.730981 0.509483
H -2.298687 -3.740105 -1.131982
H -2.444966 -2.902209 -2.673727
C -0.782657 -4.268039 -2.573102
C 0.831922 -2.425905 -3.116974
H 0.463417 -0.562849 -2.074334
H -0.769032 -0.997838 -3.256422
H -2.182368 4.468819 -2.498199
C -2.905893 4.590004 -0.485921
H -1.874737 2.101923 -3.532645
H -0.829970 1.055740 -2.553803
H -0.304807 2.688848 -3.009981
H -3.587718 4.322603 1.532446
H -2.145444 2.109287 2.907936
H -2.895513 0.786636 2.016576
H -3.860508 2.185712 2.518865
H 1.295603 1.356448 6.244170
H 1.893039 0.042729 5.234636
H 3.268068 5.228580 -1.759444
H 3.512099 4.624351 -0.122957
H -5.042004 -0.016485 -4.176652
H -5.216225 -1.489884 -3.226604
C -6.091484 0.251900 -2.301848
H -6.279897 -1.277288 -0.790127
H -6.760136 0.327752 -0.243270

H -0.511861 -3.802718 3.463959
H -1.511374 -2.389036 3.793456
C -2.634731 -4.119764 3.162782
H -4.101663 -2.543288 3.232229
H -4.716717 -4.056058 2.573926
H -1.364876 -5.103266 -2.981655
H -0.126930 -4.692499 -1.797377
C 0.068909 -3.626354 -3.658252
H 1.417001 -1.947705 -3.913079
H 1.551964 -2.768413 -2.356858
C -3.384593 6.005774 -0.596241
H -7.086158 0.051872 -2.719770
H -5.951177 1.342879 -2.335149
H -2.837781 -4.440578 4.192192
H -2.452979 -5.035219 2.579791
H 0.762945 -4.361768 -4.084091
H -0.582690 -3.297646 -4.481782
H -2.712450 6.688322 -0.058805
H -4.381261 6.126862 -0.155913
H -3.425720 6.335573 -1.639439
C 3.798335 -2.214373 -0.155928
C 3.068536 -2.989272 0.466503
C 4.646534 -1.316753 -0.873227
C 4.547122 -1.225033 -2.269363
C 5.516868 -0.470528 -0.170233
C 5.313078 -0.290871 -2.950217
H 3.862195 -1.877619 -2.804253
C 6.277537 0.458144 -0.863911
H 5.579207 -0.542302 0.912901
C 6.174376 0.550658 -2.250042
H 5.236103 -0.215640 -4.032306
H 6.951037 1.117329 -0.321323
H 6.768541 1.285851 -2.788547
H 2.677418 -3.862364 0.959954

$^{Cy}Y_{Mes}$-PCy$_2$-Au(pa)$^+$ C2:

Au -2.250690 -0.709956 -1.189637
P -0.255461 -1.246065 -0.111165
C 0.574189 0.312345 0.006039
C -0.797216 -2.135626 1.440007
C 0.747300 -2.531562 -1.035049
P 2.247572 0.654626 0.350007
C -0.204163 1.442167 -0.625844
H 0.104081 -2.544425 1.921735
C -1.735162 -3.291962 1.070623
C -1.527963 -1.231232 2.424591
H 1.544306 -1.925185 -1.489886
C 1.410962 -3.604047 -0.165962
C -0.035281 -3.160227 -2.192082
C 2.589784 2.459753 0.525833
C 3.501258 0.111229 -0.960874
C 2.869414 -0.177630 1.875797
C -1.011477 2.299645 0.157289
C -0.165914 1.668536 -2.023407

H -1.294405 -3.960608 0.325069
H -2.632879 -2.861551 0.597100
C -2.148299 -4.083420 2.305389
C -1.949686 -1.991895 3.674627
H -0.920165 -0.368095 2.697714
H -2.418094 -0.826951 1.921565
H 2.001602 -3.146365 0.639556
H 0.646270 -4.207625 0.335396
C 2.281300 -4.534132 -1.003956
C 0.845975 -4.094985 -3.009462
H -0.900823 -3.712280 -1.796905
H -0.446587 -2.365527 -2.829651
H 3.687380 2.424417 0.618761
C 2.068664 3.146058 1.787496
C 2.267126 3.351921 -0.681008
H 2.860960 0.037990 -1.849645
C 4.097604 -1.278911 -0.718183
C 4.651533 1.068485 -1.302627
H 3.019269 -1.215718 1.541152
C 4.227853 0.354110 2.339823
C 1.891056 -0.244097 3.041832
C -1.659292 3.383717 -0.435791
C -1.262379 2.058935 1.613504
C -0.852019 2.748027 -2.579305
C 0.601571 0.788537 -2.965864
H -2.814122 -4.904838 2.011490
H -1.256051 -4.548375 2.751181
C -2.824241 -3.188836 3.333486
H -2.475504 -1.313269 4.358908
H -1.047395 -2.332994 4.205585
H 3.121376 -3.968166 -1.430434
H 2.719858 -5.306560 -0.359836
C 1.484918 -5.164814 -2.136589
H 1.638048 -3.503288 -3.494165
H 0.257628 -4.551165 -3.815584
H 0.990102 3.308968 1.688690
H 2.221731 2.530022 2.678321
C 2.773929 4.488292 1.961163
C 3.016485 4.670959 -0.542476
H 2.493425 2.861493 -1.634319
H 1.192758 3.563557 -0.694684
H 4.810311 -1.230519 0.116684
H 3.332100 -1.997766 -0.422348
C 4.823226 -1.784485 -1.958423
C 5.391375 0.584796 -2.546551
H 4.303235 2.088830 -1.474199
H 5.355337 1.115576 -0.458849
H 4.937538 0.421753 1.507250
H 4.103356 1.375531 2.726141
C 4.789688 -0.533167 3.444587
C 2.448810 -1.134420 4.144682
H 0.932502 -0.623428 2.690521
H 1.700352 0.759808 3.443448
H -2.253158 4.043629 0.197070
C -1.591242 3.635701 -1.802922

H -1.524549 2.990235 2.127649
H -0.403287 1.600909 2.109268
H -2.111560 1.370947 1.732191
H -0.797829 2.904039 -3.656951
H 0.127088 0.776686 -3.952970
H 0.671262 -0.241162 -2.604283
H 1.626686 1.155393 -3.115616
H -3.069802 -3.758823 4.238447
H -3.780557 -2.828661 2.922674
H 2.130162 -5.816215 -2.739099
H 0.696935 -5.807034 -1.714584
H 2.394060 4.992177 2.858905
H 3.845369 4.305385 2.135849
C 2.604198 5.382414 0.739944
H 4.101383 4.481828 -0.535192
H 2.814032 5.307190 -1.413203
H 5.233526 -2.783793 -1.764629
H 4.094719 -1.898576 -2.776775
C 5.927523 -0.827740 -2.378687
H 4.698964 0.615015 -3.401592
H 6.206667 1.280647 -2.781363
H 5.752662 -0.134509 3.787095
H 4.993211 -1.532267 3.030139
C 3.816563 -0.653545 4.609707
H 1.744626 -1.170639 4.985749
H 2.532777 -2.163745 3.762369
C -2.329090 4.787281 -2.416881
H 3.174030 6.311628 0.866714
H 1.546066 5.672549 0.654989
H 6.403482 -1.171405 -3.305576
H 6.710873 -0.826029 -1.605883
H 4.220639 -1.327938 5.374999
H 3.706407 0.331590 5.087719
H -3.381099 4.526023 -2.599893
H -1.894267 5.077174 -3.379209
H -2.325099 5.662583 -1.757762
C -4.085833 -0.189525 -2.382174
C -4.362395 0.255796 -1.265750
C -4.726533 0.800976 0.005493
C -4.908619 -0.053289 1.103806
C -4.855733 2.186966 0.165352
C -5.198032 0.478893 2.350710
H -4.802856 -1.127650 0.969273
C -5.151855 2.707322 1.416861
H -4.711192 2.840975 -0.690037
C -5.315399 1.858043 2.508750
H -5.326822 -0.185228 3.202975
H -5.249049 3.783204 1.543132
H -5.537652 2.273798 3.489222
H -4.128396 -0.493220 -3.413988

$^{Cy}Y_{Mes}$-PCy_2-Au(dpa)$^{+}$:

Au 1.731872 0.544496 0.134588
P -0.448480 0.987145 0.799768

C -1.741015 0.038699 0.057069
C -0.372619 0.829498 2.648741
C -0.843996 2.776902 0.486693
P -1.445362 -1.413307 -0.846028
C -3.123979 0.599355 0.282233
H -1.372634 1.122886 3.005040
C 0.671627 1.751874 3.278918
C -0.079489 -0.604933 3.073821
H -1.377963 2.701382 -0.465302
C -1.801577 3.416894 1.497733
C 0.370108 3.669003 0.221944
C -2.959893 -2.063038 -1.652346
C -0.877312 -2.888254 0.170752
C -0.128594 -1.231530 -2.138269
C -3.688496 1.564970 -0.589021
C -3.859949 0.271554 1.450309
H 0.511197 2.796847 2.997312
H 1.664261 1.475313 2.884538
C 0.669157 1.622232 4.798513
C -0.115564 -0.755727 4.587148
H -0.771772 -1.300205 2.590575
H 0.928598 -0.870887 2.719136
H -2.676823 2.779368 1.667907
H -1.300658 3.532355 2.468233
C -2.240408 4.790768 1.006232
C -0.071834 5.056443 -0.229961
H 0.994039 3.749673 1.124220
H 0.999978 3.213659 -0.553340
H -2.565189 -2.985199 -2.108203
C -3.563662 -1.232946 -2.781495
C -4.062031 -2.467797 -0.667808
H -1.423018 -2.717119 1.110291
C 0.612799 -2.834080 0.499460
C -1.233784 -4.309098 -0.286573
H 0.799070 -1.292952 -1.548553
C -0.093281 -2.384622 -3.142887
C -0.091736 0.114058 -2.857313
C -4.878414 2.211361 -0.244771
C -3.081288 1.963028 -1.904743
C -5.040817 0.944904 1.754867
C -3.447783 -0.827544 2.384573
H 1.437889 2.277353 5.227896
H -0.297192 1.982098 5.182289
C 0.883986 0.183661 5.247182
H 0.092806 -1.797470 4.863864
H -1.130070 -0.531153 4.951003
H -2.816339 4.669057 0.075397
H -2.922194 5.246144 1.735772
C -1.042882 5.695185 0.752698
H -0.561808 4.966801 -1.211724
H 0.807147 5.697256 -0.378199
H -4.038810 -0.345710 -2.351896
H -2.794533 -0.885574 -3.480448
C -4.614720 -2.056182 -3.517867
C -5.113044 -3.303615 -1.387324

H -3.659614 -3.015338 0.192162
H -4.530680 -1.560751 -0.268773
H 1.193998 -3.085638 -0.401168
H 0.910512 -1.818268 0.785849
C 0.963402 -3.814785 1.607306
C -0.892189 -5.308742 0.815847
H -2.293124 -4.407496 -0.540111
H -0.667357 -4.570461 -1.190119
H -0.103657 -3.351623 -2.629060
H -0.994365 -2.351228 -3.772633
C 1.144456 -2.283234 -4.027350
C 1.152074 0.207180 -3.730052
H -0.105638 0.933180 -2.127802
H -0.984738 0.232452 -3.484724
H -5.279050 2.958439 -0.930687
C -5.561494 1.942750 0.934321
H -3.797765 1.811952 -2.721192
H -2.184154 1.385923 -2.123009
H -2.822263 3.029581 -1.917184
H -5.578503 0.669854 2.662616
H -2.891614 -0.450613 3.251781
H -2.815435 -1.560889 1.883421
H -4.331454 -1.343983 2.775272
H 0.819477 0.110705 6.340121
H 1.903524 -0.131771 4.975019
H -1.371168 6.674003 0.380449
H -0.524660 5.881282 1.705908
H -5.053346 -1.458585 -4.326841
H -4.125643 -2.919003 -3.995419
C -5.701297 -2.545567 -2.569948
H -4.651819 -4.239220 -1.740626
H -5.904680 -3.591124 -0.684194
H 2.034212 -3.753571 1.828798
H 0.433462 -3.521985 2.526757
C 0.572237 -5.233238 1.220937
H -1.524947 -5.099056 1.691542
H -1.147809 -6.322538 0.482820
H 1.145734 -3.102392 -4.757184
H 2.039743 -2.419567 -3.401281
C 1.221984 -0.936433 -4.730859
H 1.178889 1.176899 -4.243047
H 2.040254 0.174717 -3.082136
C -6.805616 2.689293 1.309110
H -6.422737 -3.173969 -3.107248
H -6.262182 -1.677548 -2.191931
H 0.777624 -5.927185 2.045784
H 1.197489 -5.558543 0.375635
H 2.141544 -0.868796 -5.325956
H 0.384187 -0.849761 -5.439023
H -6.577333 3.493494 2.021984
H -7.538500 2.032084 1.790818
H -7.276015 3.150690 0.434480
C 3.843614 -0.360037 0.065108
C 3.870286 0.589098 -0.728293
C 4.128104 1.632895 -1.673717

C 4.621173 1.311379 -2.946494
C 3.887579 2.973140 -1.335958
C 4.865664 2.321846 -3.865173
H 4.803788 0.271483 -3.205966
C 4.133876 3.973671 -2.264115
H 3.510662 3.217007 -0.345090
C 4.619583 3.650571 -3.528812
H 5.248395 2.070528 -4.851743
H 3.946149 5.011820 -1.999198
H 4.809232 4.438148 -4.254855
C 4.081753 -1.458872 0.953705
C 3.874969 -1.309337 2.333360
C 4.563066 -2.675244 0.449624
C 4.170735 -2.354612 3.195520
H 3.496115 -0.364948 2.717567
C 4.859317 -3.713025 1.323270
H 4.714539 -2.792280 -0.620574
C 4.669647 -3.554513 2.693747
H 4.013491 -2.230178 4.264771
H 5.241867 -4.652445 0.930721
H 4.904521 -4.371373 3.372579

$^{Cy}Y_{Mes}$-PCy$_2$-Au(an)$^+$:

Au -1.421474 -1.680372 -0.698147
P 0.720874 -1.284950 0.040020
C 1.231121 0.408392 0.118717
C 0.839167 -2.164404 1.670780
C 1.941918 -2.168676 -1.049939
P 0.129762 1.747117 0.022380
C 2.721731 0.590544 0.277871
H 1.886555 -2.039031 1.988558
C 0.526532 -3.657304 1.561323
C -0.087120 -1.551005 2.714201
H 2.221861 -1.364112 -1.736972
C 3.221112 -2.640736 -0.348575
C 1.347533 -3.279862 -1.918180
C 1.006458 3.359763 -0.027528
C -1.000708 1.963413 1.501928
C -1.030202 1.704265 -1.431322
C 3.591442 0.634290 -0.842250
C 3.322569 0.587406 1.562756
H 1.154853 -4.149679 0.813832
H -0.514219 -3.766670 1.213522
C 0.696028 -4.349489 2.909751
C 0.101638 -2.204561 4.075303
H 0.054731 -0.467418 2.768650
H -1.125748 -1.716166 2.386146
H 3.650665 -1.845283 0.270524
H 2.989842 -3.475112 0.326888
C 4.240532 -3.114042 -1.377140
C 2.377460 -3.771880 -2.929329
H 1.012879 -4.119501 -1.292605
H 0.455152 -2.907818 -2.439171
H 0.153223 4.052387 -0.093810

C 1.890372 3.625171 -1.242549
C 1.775442 3.704323 1.251267
H -0.308686 1.726316 2.324556
C -2.129874 0.937781 1.559186
C -1.586169 3.349558 1.800683
H -1.857280 1.066837 -1.077568
C -1.625809 3.071109 -1.784448
C -0.464729 1.049973 -2.689166
C 4.975255 0.591558 -0.660839
C 3.117824 0.733910 -2.264637
C 4.709320 0.542129 1.696144
C 2.524391 0.674090 2.830051
H 0.450600 -5.414997 2.813927
H 1.755936 -4.298990 3.201158
C -0.155904 -3.702522 3.992353
H -0.565857 -1.738013 4.811446
H 1.129710 -2.025549 4.425991
H 4.539804 -2.258973 -2.003042
H 5.149896 -3.458100 -0.867869
C 3.667548 -4.217505 -2.254687
H 2.602225 -2.953898 -3.631158
H 1.953345 -4.588615 -3.527632
H 2.799964 3.024457 -1.151151
H 1.396451 3.326199 -2.174160
C 2.276234 5.099005 -1.285315
C 2.171978 5.176104 1.230263
H 1.188744 3.484317 2.150628
H 2.677556 3.083058 1.301876
H -2.909410 1.202716 0.828467
H -1.767968 -0.056545 1.276865
C -2.741788 0.907732 2.952704
C -2.231145 3.353016 3.184771
H -0.830159 4.139829 1.755327
H -2.353341 3.596770 1.055348
H -2.032619 3.571143 -0.900019
H -0.830355 3.721508 -2.175110
C -2.715548 2.924947 -2.840097
C -1.562058 0.871335 -3.728458
H -0.001728 0.085701 -2.447767
H 0.325135 1.685225 -3.111902
H 5.614346 0.613919 -1.544133
C 5.562530 0.516273 0.596236
H 3.515393 1.638064 -2.741576
H 2.031185 0.765370 -2.322132
H 3.475421 -0.113715 -2.862971
H 5.136215 0.536507 2.699411
H 2.361792 -0.310037 3.286274
H 1.545019 1.120222 2.655071
H 3.054318 1.283396 3.570572
H 0.028977 -4.181450 4.962205
H -1.219123 -3.867920 3.759607
H 4.401715 -4.531849 -3.007542
H 3.461959 -5.101026 -1.630890
H 2.910793 5.291739 -2.159474
H 1.368645 5.708235 -1.415582

C 2.992926 5.513287 -0.007149
H 1.260196 5.793484 1.246858
H 2.732398 5.422484 2.140793
H -3.525670 0.143892 3.004629
H -1.967647 0.614262 3.678235
C -3.296746 2.274925 3.321401
H -1.447770 3.188732 3.940114
H -2.657206 4.343558 3.388360
H -3.105379 3.915425 -3.106107
H -3.555767 2.368032 -2.403133
C -2.211924 2.202005 -4.081264
H -1.153419 0.391953 -4.626935
H -2.319419 0.180817 -3.324605
C 7.048077 0.411585 0.763258
H 3.228060 6.584900 -0.029313
H 3.955227 4.982346 0.048637
H -3.702210 2.266587 4.341113
H -4.137810 2.507461 2.650092
H -3.032176 2.053469 -4.795035
H -1.468207 2.834468 -4.588749
H 7.356538 -0.639208 0.852705
H 7.387560 0.925151 1.669630
H 7.580073 0.834965 -0.095378
H -3.703165 -3.041512 -1.000045
H -3.562608 -2.054961 -2.295496
C -4.382307 -1.147932 -0.662703
C -4.793886 -1.347451 0.652042
C -4.788898 -0.015184 -1.360214
C -5.615592 -0.408067 1.263654
H -4.469713 -2.233084 1.196142
C -5.596301 0.928444 -0.734209
H -4.479192 0.126031 -2.393604
C -6.010435 0.737955 0.579932
H -5.943044 -0.571864 2.288025
H -5.908351 1.813952 -1.284212
H -6.645431 1.474376 1.066777
N -3.488191 -2.086330 -1.280562

$^{Cy}Y_{SF}$-PCy$_2$-AuCl (P5):

Au -3.049384 -0.698354 0.757320
Cl -5.084521 -0.032886 1.727179
P -1.068237 -1.311487 -0.229761
C 0.003340 0.115082 -0.519359
C -0.140330 -2.549339 0.812320
C -1.517860 -2.163667 -1.805616
S 1.422127 -0.160230 -1.379273
P -0.563083 1.737006 -0.031453
H 0.705457 -1.969542 1.207875
C -0.974347 -3.016622 2.007728
C 0.425717 -3.743552 0.039828
H -0.620323 -2.692934 -2.143085
C -2.645652 -3.168322 -1.556487
C -1.934029 -1.175920 -2.886422
O 1.318226 -1.372437 -2.194526

O 1.970826 1.057599 -1.983302
C 2.806560 -0.686157 -0.168957
C -1.053651 1.717997 1.742305
C -2.031231 2.056254 -1.089828
C 0.662081 3.058695 -0.357932
H -1.342490 -2.148710 2.571246
H -1.868251 -3.544142 1.640093
C -0.163919 -3.930640 2.916566
C 1.247196 -4.632944 0.965077
H -0.400795 -4.334585 -0.376065
H 1.029108 -3.398818 -0.804444
H -2.389614 -3.872100 -0.754806
H -3.535888 -2.620285 -1.210470
C -2.981330 -3.917413 -2.840084
C -2.285414 -1.904170 -4.177138
H -2.816670 -0.619530 -2.533008
H -1.131461 -0.450319 -3.059938
F 2.830007 -2.020409 -0.088555
F 2.551207 -0.199167 1.051536
C 4.200719 -0.227129 -0.637154
H -2.054378 1.260274 1.708475
C -0.175248 0.807789 2.599582
C -1.231975 3.083518 2.408351
H -2.461298 1.044794 -1.156788
C -3.138610 2.951694 -0.539672
C -1.595832 2.446608 -2.505025
H 0.943259 2.859183 -1.398880
C 1.923594 2.944242 0.493478
C 0.125610 4.494189 -0.296842
H 0.653479 -3.346415 3.366543
H -0.792311 -4.279116 3.745979
C 0.424003 -5.108580 2.153092
H 1.640083 -5.487657 0.399852
H 2.119452 -4.067871 1.325152
H -2.107597 -4.508620 -3.152680
H -3.792880 -4.631473 -2.651190
C -3.367884 -2.951325 -3.951256
H -2.603462 -1.178764 -4.937152
H -1.379179 -2.390506 -4.567348
F 4.339794 1.084710 -0.411365
F 4.328622 -0.474607 -1.941473
C 5.348943 -0.973793 0.092474
H -0.050642 -0.166218 2.116849
H 0.832453 1.237849 2.691314
C -0.807165 0.628390 3.973035
C -1.879301 2.892606 3.776077
H -0.253050 3.564843 2.536039
H -1.842442 3.752809 1.792881
H -3.463751 2.592522 0.444895
H -2.783643 3.983178 -0.411854
C -4.321075 2.936032 -1.502390
C -2.795008 2.452761 -3.444354
H -1.146879 3.448282 -2.498633
H -0.815812 1.759467 -2.860824
H 1.689493 3.216496 1.532726

H 2.289153 1.919325 0.509433
C 2.995023 3.875202 -0.054325
C 1.189299 5.434600 -0.857339
H -0.804051 4.607706 -0.861334
H -0.091604 4.779823 0.737472
H 1.035458 -5.728938 2.820718
H -0.394380 -5.749982 1.791833
H -4.305373 -2.445324 -3.674053
H -3.571177 -3.497584 -4.881115
F 5.516492 -2.166800 -0.476996
F 5.035536 -1.142640 1.381691
C 6.692738 -0.211783 0.021134
H -1.764315 0.097650 3.846001
H -0.170403 -0.014569 4.593622
C -1.055498 1.964672 4.657591
H -2.010461 3.868372 4.259908
H -2.886122 2.470436 3.636918
H -4.743573 1.919539 -1.522424
H -5.112193 3.595214 -1.124598
C -3.908202 3.343951 -2.910001
H -2.479221 2.782870 -4.441679
H -3.173060 1.427078 -3.559492
H 3.906409 3.790870 0.550988
H 3.257195 3.533476 -1.066318
C 2.503770 5.315011 -0.097187
H 1.354209 5.193655 -1.918063
H 0.819010 6.466817 -0.823214
F 7.679785 -1.037534 0.322142
F 6.880074 0.257877 -1.203648
F 6.695824 0.793618 0.878733
H -1.555963 1.812055 5.621869
H -0.088501 2.441835 4.877945
H -4.773980 3.314054 -3.583238
H -3.559874 4.388106 -2.895610
H 2.355065 5.674666 0.932346
H 3.263100 5.966311 -0.547977

$^{Cy}Y_{SF}$-PCy_2-Au(pa)$^+$:

Au -2.480049 -0.963898 1.026131
P -0.514693 -1.389074 -0.153462
C 0.449015 0.101104 -0.448080
C 0.520453 -2.613333 0.794665
C -1.064001 -2.209746 -1.713429
S 1.815154 -0.075445 -1.420095
P -0.162343 1.680329 0.115578
H 1.357199 -2.001553 1.160083
C -0.214799 -3.161794 2.020291
C 1.099187 -3.751320 -0.050090
H -0.166225 -2.658977 -2.151964
C -2.093481 -3.301799 -1.416342
C -1.640530 -1.201683 -2.698273
O 1.695874 -1.262352 -2.268426
O 2.254410 1.189720 -2.012086
C 3.294607 -0.573808 -0.319057

C -0.581592 1.568779 1.903932
C -1.675419 1.962933 -0.889424
C 0.992776 3.068122 -0.197506
H -0.596491 -2.332034 2.632048
H -1.095191 -3.734595 1.688791
C 0.703200 -4.048058 2.851892
C 2.028325 -4.612940 0.796932
H 0.283036 -4.380320 -0.428229
H 1.626341 -3.351341 -0.920520
H -1.714298 -4.030425 -0.690225
H -2.976141 -2.838478 -0.948661
C -2.514082 -4.000797 -2.703629
C -2.067439 -1.887090 -3.988874
H -2.522291 -0.723875 -2.238363
H -0.904985 -0.415314 -2.901488
F 3.385559 -1.906164 -0.298866
F 3.084300 -0.149459 0.932782
C 4.632018 -0.021768 -0.848835
H -1.557504 1.058157 1.895612
C 0.386236 0.686627 2.693477
C -0.809315 2.900921 2.620808
H -2.071309 0.937730 -0.968433
C -2.798778 2.814626 -0.302352
C -1.264504 2.398572 -2.301104
H 1.246339 2.922085 -1.253606
C 2.287823 2.977845 0.605742
C 0.387204 4.471801 -0.063729
H 1.509022 -3.425535 3.269422
H 0.148548 -4.455943 3.706203
C 1.311612 -5.167209 2.019388
H 2.431601 -5.427832 0.183075
H 2.888555 -4.007504 1.117526
H -1.643246 -4.519206 -3.130613
H -3.259509 -4.773192 -2.476979
C -3.062382 -3.006650 -3.717835
H -2.496170 -1.146375 -4.675834
H -1.176302 -2.297330 -4.485485
F 4.713411 1.285406 -0.576372
F 4.688720 -0.211398 -2.167322
C 5.858163 -0.734259 -0.219579
H 0.547315 -0.265737 2.178536
H 1.370789 1.171625 2.748027
C -0.162172 0.437600 4.091851
C -1.376525 2.636296 4.011084
H 0.145130 3.434335 2.717904
H -1.484086 3.550333 2.053441
H -3.130142 2.389017 0.655750
H -2.454579 3.836679 -0.098876
C -3.972599 2.856110 -1.275413
C -2.468327 2.427316 -3.231250
H -0.830469 3.405917 -2.267764
H -0.474811 1.737146 -2.682918
H 2.078841 3.182498 1.665483
H 2.707263 1.974384 0.554037
C 3.290651 3.993228 0.076772

C 1.389966 5.490130 -0.600086
H -0.555010 4.561723 -0.611288
H 0.171977 4.702977 0.984629
H 2.000541 -5.763195 2.630780
H 0.512966 -5.849969 1.691949
H -3.995609 -2.571436 -3.326964
H -3.326400 -3.519307 -4.650966
F 6.049712 -1.893214 -0.847489
F 5.628834 -0.969414 1.076974
C 7.154410 0.101609 -0.335862
H -1.090807 -0.149400 4.001412
H 0.542940 -0.179910 4.661497
C -0.455488 1.738751 4.824550
H -1.541031 3.589689 4.527425
H -2.363759 2.158730 3.909712
H -4.396187 1.846270 -1.357803
H -4.765431 3.493904 -0.866703
C -3.560938 3.321442 -2.663722
H -2.155863 2.772477 -4.224126
H -2.859739 1.407785 -3.361074
H 4.224397 3.923330 0.648455
H 3.536531 3.725608 -0.961025
C 2.724121 5.404147 0.128034
H 1.544547 5.306298 -1.673679
H 0.966366 6.498104 -0.511196
F 8.198403 -0.681624 -0.131371
F 7.240451 0.631611 -1.547065
F 7.157185 1.068667 0.564569
H -0.894259 1.532108 5.808393
H 0.490754 2.268569 5.009390
H -4.432948 3.329126 -3.329289
H -3.194652 4.358116 -2.615205
H 2.579375 5.697423 1.178973
H 3.436902 6.119466 -0.300607
C -4.697646 -0.456247 1.541769
C -4.088359 -0.799293 2.558842
C -5.528328 -0.001552 0.471210
C -6.353356 1.113801 0.675021
C -5.532704 -0.663836 -0.765354
C -7.177014 1.551530 -0.352047
H -6.343543 1.624788 1.634321
C -6.351859 -0.208246 -1.787139
H -4.894025 -1.530822 -0.912751
C -7.174387 0.897171 -1.581120
H -7.820243 2.414087 -0.194563
H -6.351137 -0.719572 -2.747120
H -7.817021 1.250710 -2.384394
H -3.822805 -1.108612 3.555996

$^{Cy}Y_{SF}$-PCy_2-Au(pa)$^+$ C2:

Au -2.732141 -0.372367 -1.106429
P -1.447702 0.425203 0.651338
C 0.300944 0.493632 0.118264
C -2.090018 2.121296 1.157095

C -1.670939 -0.583732 2.187721
S 0.791386 -0.914469 -0.688891
P 1.039739 2.065763 -0.267457
H -1.416052 2.855419 0.712343
C -3.462055 2.396504 0.532039
C -2.108589 2.395865 2.660298
H -1.402919 0.099798 3.004518
C -3.146692 -0.965631 2.321554
C -0.775274 -1.802440 2.327616
O -0.295665 -1.901104 -0.705685
O 1.498260 -0.664040 -1.948061
C 2.100728 -1.854971 0.366686
C 1.146179 3.043670 1.303542
C -0.141496 2.817507 -1.477524
C 2.710054 1.972548 -1.051089
H -3.409307 2.258588 -0.557212
H -4.192377 1.659989 0.897247
C -3.930899 3.811496 0.849603
C -2.543900 3.830490 2.933028
H -2.811806 1.714334 3.155877
H -1.124742 2.207293 3.102696
H -3.795660 -0.084988 2.227289
H -3.408669 -1.634711 1.487358
C -3.406417 -1.678454 3.642614
C -1.035544 -2.497666 3.658447
H -0.957062 -2.503795 1.505752
H 0.271166 -1.488079 2.271331
F 1.522202 -2.963661 0.817338
F 2.469352 -1.098133 1.410490
C 3.365541 -2.289404 -0.406487
H 0.119607 3.375634 1.498904
C 1.567365 2.166203 2.490446
C 1.987397 4.322160 1.218439
H -1.102839 2.450518 -1.094659
C -0.254296 4.338313 -1.538845
C -0.029698 2.148929 -2.851103
H 2.593230 1.181791 -1.802581
C 3.825583 1.562556 -0.084068
C 3.081054 3.270243 -1.787751
H -3.267901 4.527557 0.340410
H -4.932798 3.965482 0.430684
C -3.918005 4.102644 2.342026
H -2.538755 4.014097 4.014290
H -1.808845 4.525874 2.495352
H -3.231454 -0.977625 4.472724
H -4.461658 -1.973383 3.698389
C -2.499081 -2.889931 3.800217
H -0.387826 -3.379311 3.740837
H -0.756514 -1.824466 4.483185
F 3.886942 -1.282944 -1.105956
F 3.022780 -3.275678 -1.239691
C 4.487205 -2.779589 0.551004
H 0.909547 1.291106 2.564305
H 2.575170 1.767253 2.323426
C 1.543979 2.964165 3.789630

C 1.897446 5.095228 2.528263
H 3.038554 4.071522 1.037452
H 1.661728 4.950254 0.382592
H -0.426828 4.745741 -0.532850
H 0.678194 4.786490 -1.904777
C -1.410393 4.732296 -2.453817
C -1.210658 2.570338 -3.715537
H 0.906373 2.424168 -3.352436
H -0.011533 1.059542 -2.729909
H 3.933051 2.322697 0.698415
H 3.577617 0.629705 0.426038
C 5.161334 1.433753 -0.807564
C 4.421248 3.110731 -2.497706
H 2.316617 3.535699 -2.521341
H 3.151981 4.108404 -1.086603
H -4.218825 5.140502 2.529544
H -4.658888 3.464493 2.846382
H -2.750643 -3.630035 3.025378
H -2.675122 -3.375757 4.768021
F 3.967729 -3.472061 1.566792
F 5.116594 -1.704115 1.035101
C 5.532087 -3.674198 -0.155497
H 0.504886 3.231419 4.033954
H 1.901610 2.328893 4.608993
C 2.364841 4.240780 3.695654
H 2.495217 6.011268 2.450627
H 0.856218 5.411024 2.696127
H -2.356328 4.419149 -1.986125
H -1.453458 5.824652 -2.540249
C -1.300700 4.085157 -3.827021
H -1.123501 2.110019 -4.707171
H -2.137046 2.177025 -3.265234
H 5.934523 1.153502 -0.081377
H 5.106999 0.616789 -1.537044
C 5.525916 2.719979 -1.529469
H 4.326670 2.338571 -3.275283
H 4.665380 4.047951 -3.012663
F 6.609679 -3.763203 0.604019
F 5.037651 -4.883319 -0.349065
F 5.868086 -3.146935 -1.323356
H 2.298910 4.803212 4.634881
H 3.426106 3.986136 3.555487
H -2.157467 4.373961 -4.448123
H -0.402624 4.463124 -4.338266
H 5.671799 3.528068 -0.796549
H 6.477696 2.603012 -2.061996
C -3.877715 -0.945109 -2.946444
C -4.002391 -1.935163 -2.219605
C -4.229842 -3.104034 -1.425541
C -5.532172 -3.601754 -1.278740
C -3.155908 -3.727087 -0.769563
C -5.751332 -4.718855 -0.485527
H -6.358857 -3.109625 -1.784833
C -3.392879 -4.838355 0.025701
H -2.151349 -3.323782 -0.880077

C -4.686674 -5.334334 0.168338
H -6.760279 -5.108791 -0.372430
H -2.562397 -5.319263 0.537718
H -4.867141 -6.205891 0.794015
H -3.884862 -0.261270 -3.777914

$^{Cy}Y_{SF}$-PCy_2-Au(pa)$^+$ C3:

Au -0.324338 -1.684269 1.669495
P 0.778882 -1.458548 -0.374343
C 0.868821 0.293104 -0.895001
C 2.512588 -2.107280 -0.183622
C 0.079857 -2.757063 -1.545680
S -0.361314 1.014393 -1.816962
P 2.167566 1.329218 -0.212475
H 3.076619 -1.331168 0.347096
C 2.533252 -3.345086 0.715488
C 3.233882 -2.394049 -1.500414
H 0.959994 -3.404804 -1.635235
C -0.988663 -3.619401 -0.868764
C -0.310452 -2.372849 -2.965177
O -0.379446 2.465825 -1.639674
O -0.505599 0.500746 -3.175521
C -1.991699 0.542929 -1.021520
C 3.739766 0.970703 -1.118779
C 2.263677 0.835014 1.565947
C 1.892034 3.158397 -0.258887
H 2.030843 -3.133427 1.669078
H 1.969481 -4.160676 0.236037
C 3.971456 -3.783576 0.970711
C 4.681097 -2.786821 -1.227116
H 2.752539 -3.232287 -2.019304
H 3.166577 -1.540173 -2.180140
H -0.640238 -3.935580 0.123757
H -1.906390 -3.039329 -0.713286
C -1.288369 -4.851134 -1.717043
C -0.581972 -3.628911 -3.784344
H -1.211815 -1.753149 -2.954820
H 0.474059 -1.767223 -3.430310
F -2.086396 1.179333 0.148429
F -2.031831 -0.778236 -0.791429
C -3.194158 0.896071 -1.932031
H 4.008869 -0.045489 -0.804348
C 3.525632 0.947189 -2.638860
C 4.936809 1.866313 -0.779841
H 2.003401 -0.231744 1.542941
C 3.636292 0.900985 2.234682
C 1.129093 1.480955 2.373188
H 0.841733 3.250610 0.043478
C 2.052813 3.826219 -1.632527
C 2.745801 3.948112 0.748722
H 4.473993 -3.002718 1.561527
H 3.976244 -4.691503 1.586016
C 4.742327 -4.009554 -0.322172
H 5.197077 -2.980475 -2.175710

H 5.211354 -1.946239 -0.750900
H -0.397287 -5.496873 -1.736161
H -2.085811 -5.434446 -1.239945
C -1.666574 -4.480492 -3.142902
H -0.866458 -3.341905 -4.804165
H 0.343780 -4.218628 -3.870434
F -3.321414 -0.071217 -2.847529
F -2.991255 2.060532 -2.543114
C -4.518947 0.997974 -1.138221
H 2.645775 0.347021 -2.900449
H 3.317757 1.963064 -2.993004
C 4.777866 0.431413 -3.338784
C 6.187692 1.333211 -1.470686
H 4.746823 2.883373 -1.141584
H 5.102813 1.937683 0.298490
H 4.359458 0.315371 1.651268
H 4.015666 1.929729 2.267267
C 3.552090 0.350833 3.652398
C 1.130076 0.967347 3.808654
H 1.217417 2.571776 2.387236
H 0.167324 1.256249 1.888226
H 3.120780 3.885617 -1.879632
H 1.558601 3.260336 -2.421992
C 1.484824 5.237423 -1.569178
C 2.196459 5.370348 0.839167
H 2.748826 3.502625 1.744339
H 3.789264 3.983869 0.416597
H 5.784293 -4.270787 -0.100943
H 4.310395 -4.869062 -0.856068
H -2.610769 -3.915359 -3.134465
H -1.848045 -5.385847 -3.735541
F -4.538440 2.158277 -0.482172
F -4.581753 -0.007650 -0.251942
C -5.782875 0.917382 -2.026768
H 4.956151 -0.613812 -3.046460
H 4.615105 0.429296 -4.423338
C 5.999897 1.263067 -2.978489
H 7.043572 1.970070 -1.216044
H 6.416318 0.329639 -1.080060
H 3.298988 -0.720606 3.607641
H 4.535775 0.421361 4.131661
C 2.497310 1.085951 4.464101
H 0.375745 1.516036 4.383199
H 0.825415 -0.090053 3.816288
H 1.570862 5.712885 -2.554052
H 0.410771 5.169710 -1.343244
C 2.196492 6.068963 -0.512427
H 1.169707 5.325727 1.232158
H 2.786885 5.940595 1.567084
F -6.824362 1.332372 -1.326960
F -5.641604 1.687168 -3.093514
F -5.989359 -0.329321 -2.410602
H 6.896624 0.850722 -3.457109
H 5.873787 2.282358 -3.373312
H 2.458768 0.692400 5.487198

H 2.777245 2.146990 4.548184
H 3.237473 6.233542 -0.829012
H 1.736249 7.061262 -0.426766
C -1.049038 -2.544902 3.590642
C -1.696535 -1.495239 3.592964
C -2.462243 -0.297624 3.717114
C -2.267951 0.536686 4.827639
C -3.407613 0.039818 2.739081
C -3.012685 1.700337 4.946379
H -1.537404 0.264587 5.585036
C -4.142931 1.208151 2.866806
H -3.550568 -0.619543 1.887709
C -3.943690 2.038653 3.966893
H -2.865616 2.348859 5.806919
H -4.872049 1.473037 2.105322
H -4.521334 2.955470 4.063623
H -0.667398 -3.523366 3.828998

$^{Cy}Y_{SF}$-PCy$_2$-Au(an)$^+$:

Au 2.673521 -1.082494 -0.205813
P 0.480947 -1.550541 0.332533
C -0.462784 -0.067264 0.720170
C -0.351422 -2.466398 -1.060362
C 0.584522 -2.708589 1.769078
S -2.032865 -0.312104 1.286416
P 0.330491 1.526596 0.627405
H -1.063321 -1.729439 -1.458613
C 0.628742 -2.814086 -2.184220
C -1.140159 -3.704490 -0.625780
H -0.395727 -3.191922 1.840290
C 1.660370 -3.769878 1.527104
C 0.855812 -1.972871 3.074091
O -2.171533 -1.656096 1.850017
O -2.554324 0.836160 2.029799
C -3.224313 -0.441552 -0.202596
C 1.101853 1.740547 -1.027370
C 1.663867 1.481222 1.892379
C -0.796130 2.913599 1.022779
H 1.165651 -1.911890 -2.508331
H 1.391562 -3.506797 -1.794876
C -0.096575 -3.447169 -3.364443
C -1.873106 -4.308549 -1.817418
H -0.448276 -4.454035 -0.219121
H -1.843229 -3.449753 0.171944
H 1.503628 -4.292612 0.575035
H 2.639359 -3.270037 1.448235
C 1.697610 -4.760701 2.684137
C 0.913977 -2.949338 4.241567
H 1.820698 -1.447645 2.990469
H 0.081124 -1.218288 3.245741
F -3.352446 -1.727821 -0.540633
F -2.712449 0.216394 -1.248984
C -4.630296 0.096052 0.127282
H 2.041760 1.171459 -0.930767

C 0.314966 1.101914 -2.169110
C 1.502928 3.171291 -1.389063
H 1.987407 0.431201 1.814297
C 2.908172 2.329182 1.637045
C 1.101581 1.660866 3.304221
H -1.260861 2.568156 1.954035
C -1.906040 3.109975 -0.007849
C -0.121859 4.259187 1.319820
H -0.768000 -2.696636 -3.808404
H 0.628947 -3.716669 -4.142319
C -0.910055 -4.661401 -2.940979
H -2.429913 -5.196274 -1.492543
H -2.618773 -3.587939 -2.183906
H 0.740111 -5.300156 2.728074
H 2.474326 -5.513977 2.502031
C 1.942905 -4.045972 4.005717
H 1.135367 -2.404505 5.168062
H -0.080058 -3.400914 4.373450
F -4.603529 1.433541 0.143772
F -5.000707 -0.362025 1.323349
C -5.696440 -0.364093 -0.901745
H 0.004249 0.087055 -1.902984
H -0.608760 1.669825 -2.349219
C 1.181547 1.070330 -3.420554
C 2.381327 3.137778 -2.631968
H 0.601571 3.765517 -1.587765
H 2.035719 3.660696 -0.567662
H 3.307345 2.131100 0.634240
H 2.667544 3.399702 1.678133
C 3.966941 2.010655 2.686519
C 2.179881 1.368653 4.341251
H 0.745147 2.689623 3.441812
H 0.230128 1.007974 3.446493
H -1.478793 3.531661 -0.928822
H -2.362141 2.159210 -0.278418
C -2.957682 4.057669 0.550564
C -1.168613 5.211256 1.892303
H 0.707412 4.154893 2.025164
H 0.288649 4.693872 0.402497
H -1.455206 -5.072056 -3.799978
H -0.226788 -5.452393 -2.596175
H 2.949119 -3.599146 3.989253
H 1.934192 -4.762964 4.836044
F -6.063281 -1.611390 -0.611152
F -5.177780 -0.334576 -2.134264
C -6.959817 0.527707 -0.888599
H 2.042673 0.411849 -3.221368
H 0.624199 0.618169 -4.250225
C 1.684347 2.456092 -3.799975
H 2.680187 4.157664 -2.903459
H 3.304793 2.595035 -2.387275
H 4.296628 0.966649 2.555475
H 4.850557 2.639689 2.520571
C 3.429321 2.204363 4.097947
H 1.781921 1.554182 5.346094

H 2.442575 0.301820 4.302918
H -3.759416 4.196931 -0.185240
H -3.415637 3.580193 1.429020
C -2.343454 5.393968 0.940880
H -1.529694 4.810032 2.850789
H -0.699315 6.177975 2.112857
F -7.947296 -0.105718 -1.495992
F -7.314156 0.785986 0.361632
F -6.724030 1.664896 -1.518864
H 2.365792 2.389365 -4.657779
H 0.831999 3.072097 -4.123267
H 4.201354 1.956152 4.836496
H 3.187902 3.267866 4.244676
H -1.992282 5.907413 0.033016
H -3.100536 6.045646 1.394396
H 5.124811 -1.707808 -0.931956
H 5.234159 -0.653261 0.305207
C 5.145752 0.237346 -1.518340
C 4.923000 0.058160 -2.881085
C 5.716497 1.414141 -1.045044
C 5.303130 1.052906 -3.774183
H 4.464676 -0.861907 -3.239727
C 6.088825 2.406861 -1.946361
H 5.887257 1.546098 0.022504
C 5.891279 2.226887 -3.311157
H 5.138203 0.906683 -4.839447
H 6.542952 3.322678 -1.574718
H 6.188593 3.002302 -4.013120
N 4.785147 -0.807315 -0.596075

^{Cy}JohnPhos-AuCl (L6):

Au -0.538439 -1.416146 0.574089
Cl -1.847647 -3.208111 1.328043
P 0.865088 0.250664 -0.111551
C 2.187428 -0.510255 -1.149781
H 2.886935 0.285919 -1.441288
C 1.567048 -1.103079 -2.416033
C 2.961691 -1.566978 -0.362982
C 1.704819 0.936276 1.388107
C 0.252751 1.656104 -1.120486
H 1.048370 -0.319893 -2.984144
H 0.802202 -1.838566 -2.119953
C 2.619387 -1.785181 -3.280266
H 2.141540 -2.225369 -4.164493
H 3.328604 -1.029638 -3.650928
C 3.376565 -2.845959 -2.494608
C 4.002062 -2.252975 -1.240599
H 4.516779 -3.029139 -0.660496
H 4.769014 -1.518539 -1.529732
H 4.147438 -3.310699 -3.122240
H 2.680323 -3.648356 -2.207192
H 3.451108 -1.116151 0.509643
H 2.250729 -2.314979 0.022533

C 0.702870 1.552108 2.362773
H 0.246025 2.438633 1.896525
H -0.108734 0.840546 2.564045
C 1.383933 1.948749 3.667181
H 2.095786 0.018289 1.855124
C 2.875149 1.882968 1.138640
C 1.256223 2.475493 -1.661267
C -1.101886 1.947213 -1.401281
H 3.598708 1.437774 0.442241
H 2.501965 2.805271 0.673893
C 3.559887 2.243821 2.452467
H 3.992871 1.335283 2.897157
H 4.396004 2.927143 2.257673
C 2.573339 2.867635 3.428999
H 2.216132 3.823814 3.017246
H 3.070251 3.102653 4.378732
H 1.726827 1.036768 4.178451
H 0.654518 2.424945 4.334368
H 2.301710 2.239333 -1.485068
C 0.957535 3.588017 -2.432856
H 1.759918 4.202713 -2.834533
C -0.372373 3.895294 -2.684214
H -0.634133 4.764047 -3.285090
C -1.373875 3.078577 -2.179224
H -2.407519 3.325092 -2.403952
C -2.252537 1.110704 -0.895367
H -1.908361 0.066042 -0.871111
C -2.634348 1.488713 0.539892
C -3.495997 1.117855 -1.783218
H -1.743042 1.446749 1.177015
H -2.974759 2.535774 0.546446
C -3.718769 0.574877 1.093408
H -3.306327 -0.440318 1.196226
H -4.001980 0.899282 2.103246
C -4.937577 0.526611 0.183264
H -5.425844 1.513534 0.173761
H -5.676630 -0.183980 0.575075
C -4.543772 0.155343 -1.239933
H -4.138028 -0.867885 -1.250333
H -5.425019 0.149989 -1.894050
H -3.936480 2.125189 -1.811479
H -3.223854 0.860602 -2.815481

^{Cy}JohnPhos-Au(pa)$^{+}$:

Au -0.408761 0.763539 -0.941414
P 1.502232 0.109571 0.195776
C 2.085160 1.564248 1.162935
H 3.028583 1.277742 1.649141
C 1.058342 1.899450 2.246099
C 2.352414 2.763399 0.253729
C 2.768367 -0.239844 -1.105148
C 1.478207 -1.276176 1.387118

H 0.913098 1.037008 2.909029
H 0.087324 2.094268 1.761953
C 1.480432 3.125295 3.044581
H 0.713008 3.359755 3.792561
H 2.400199 2.892967 3.601623
C 1.729061 4.319430 2.134815
C 2.760230 3.986491 1.066744
H 2.911044 4.839296 0.393460
H 3.730960 3.791058 1.545890
H 2.055823 5.186467 2.721989
H 0.784341 4.608443 1.649146
H 3.132232 2.525766 -0.480775
H 1.436268 2.990222 -0.315364
C 2.346508 -1.409524 -1.993472
H 2.309026 -2.326772 -1.386129
H 1.332875 -1.240108 -2.381451
C 3.320219 -1.594814 -3.151450
H 2.722685 0.676597 -1.715079
C 4.201348 -0.414158 -0.609305
C 2.663493 -1.421249 2.125737
C 0.400446 -2.158893 1.619410
H 4.502846 0.431789 0.023154
H 4.263928 -1.320957 0.006144
C 5.156481 -0.562495 -1.788500
H 5.148302 0.363621 -2.382447
H 6.180895 -0.688435 -1.417224
C 4.756232 -1.737338 -2.668847
H 4.855440 -2.669381 -2.092338
H 5.437557 -1.825221 -3.524123
H 3.247611 -0.722941 -3.818651
H 3.022134 -2.468008 -3.744851
H 3.480780 -0.719331 1.987028
C 2.825213 -2.441586 3.049545
H 3.756100 -2.529547 3.604749
C 1.784162 -3.336985 3.252393
H 1.887804 -4.149495 3.968616
C 0.596071 -3.183331 2.552344
H -0.213412 -3.881080 2.744833
C -0.931580 -2.049272 0.916271
H -1.119107 -0.977219 0.740359
C -0.911842 -2.741158 -0.451175
C -2.124924 -2.548672 1.730354
H -0.068559 -2.367308 -1.044653
H -0.726043 -3.814075 -0.292681
C -2.219282 -2.541189 -1.205150
H -2.319445 -1.476439 -1.467911
H -2.190297 -3.088509 -2.156010
C -3.424317 -2.964613 -0.377935
H -3.400722 -4.055934 -0.237645
H -4.353587 -2.739179 -0.917363
C -3.425337 -2.287126 0.985162
H -3.554166 -1.203435 0.855592
H -4.278450 -2.631158 1.583346
H -2.035326 -3.630407 1.903264
H -2.135108 -2.068355 2.717502

C -2.536477 1.428357 -1.660646
C -1.682130 1.662725 -2.519949
H -1.168150 2.017542 -3.397680
C -3.631692 1.173422 -0.779447
C -4.722104 0.423147 -1.243810
C -3.623534 1.669450 0.532787
C -5.797332 0.185726 -0.400142
H -4.715944 0.034169 -2.258989
C -4.702745 1.419612 1.366521
H -2.770893 2.246322 0.884088
C -5.788573 0.681095 0.901335
H -6.645094 -0.393585 -0.758295
H -4.698289 1.802699 2.384181
H -6.632696 0.487431 1.559603

^{Cy}JohnPhos-Au(pa)$^{+}$ C2:

Au -0.397256 -1.356406 0.012976
P 0.349919 0.829698 -0.126145
C 1.131361 1.345563 1.464329
H 1.811846 0.509918 1.689386
C 0.103124 1.399526 2.594471
C 1.980981 2.613508 1.390902
C -1.213082 1.786529 -0.373401
C 1.569957 1.118679 -1.443457
H -0.467330 0.459893 2.629917
H -0.620252 2.204254 2.401265
C 0.796541 1.660201 3.926014
H 0.048775 1.731536 4.725491
H 1.437229 0.799744 4.170066
C 1.645349 2.923293 3.870270
C 2.657205 2.862652 2.734191
H 3.241937 3.789493 2.687343
H 3.374368 2.049961 2.927738
H 2.157366 3.078603 4.827938
H 0.988138 3.793854 3.722985
H 2.731623 2.520460 0.596439
H 1.354835 3.476501 1.129133
C -1.962568 1.332205 -1.631161
H -1.422544 1.657318 -2.530503
H -2.001647 0.234961 -1.671452
C -3.373352 1.904165 -1.647295
H -1.789163 1.402359 0.486079
C -1.188867 3.310635 -0.236656
C 1.356755 2.134040 -2.382699
C 2.738517 0.332832 -1.514443
H -0.703350 3.604275 0.699617
H -0.607918 3.770209 -1.045422
C -2.613080 3.856761 -0.268343
H -3.152481 3.494780 0.619939
H -2.586064 4.950767 -0.193503
C -3.359838 3.420451 -1.520170
H -2.870496 3.854713 -2.405138
H -4.384597 3.811957 -1.506973
H -3.940886 1.470229 -0.810749

H -3.885656 1.594950 -2.566752
H 0.462222 2.744444 -2.333569
C 2.278739 2.382727 -3.389511
H 2.092686 3.176913 -4.108823
C 3.433825 1.614214 -3.464161
H 4.167794 1.800131 -4.245498
C 3.652026 0.605270 -2.535974
H 4.560014 0.008187 -2.602614
C 3.041642 -0.781280 -0.545281
H 2.214436 -0.854875 0.173784
C 3.110721 -2.149689 -1.229106
C 4.303317 -0.526412 0.284187
H 2.190874 -2.319294 -1.805837
H 3.940541 -2.155376 -1.950930
C 3.312641 -3.254582 -0.202254
H 2.425972 -3.296371 0.451685
H 3.376868 -4.229227 -0.702306
C 4.553462 -3.005552 0.644703
H 5.443611 -3.065482 0.000380
H 4.664257 -3.791861 1.402040
C 4.511410 -1.634788 1.306841
H 3.686010 -1.608557 2.036054
H 5.431767 -1.454079 1.876147
H 5.172948 -0.472094 -0.387603
H 4.233092 0.451071 0.781034
C -2.184141 -2.795193 0.228157
C -1.141598 -3.455586 0.162842
H -0.445207 -4.277670 0.167371
C -3.419188 -2.075637 0.290622
C -3.694014 -1.256188 1.397456
C -4.335993 -2.161562 -0.766464
C -4.881786 -0.543649 1.446233
H -2.970155 -1.187000 2.206613
C -5.522484 -1.444818 -0.702805
H -4.111897 -2.789252 -1.625145
C -5.796573 -0.639562 0.399602
H -5.096658 0.089460 2.303926
H -6.236901 -1.513277 -1.519791
H -6.727638 -0.078402 0.441442

^{Cy}JohnPhos-Au(dpa)$^{+}$:

Au -0.428184 0.565382 0.152309
P 1.496231 -0.701913 0.321624
C 2.053546 -0.609235 2.073358
H 3.001293 -1.161899 2.146438
C 1.016248 -1.289934 2.968819
C 2.297467 0.838779 2.498811
C 2.753904 0.171779 -0.711606
C 1.505859 -2.479951 -0.092894
H 0.888700 -2.338105 2.669502
H 0.042271 -0.796182 2.815987
C 1.406494 -1.194107 4.437441
H 0.630468 -1.662496 5.055392
H 2.329013 -1.769854 4.604050

C 1.629642 0.251560 4.856979
C 2.671751 0.921792 3.973927
H 2.803615 1.972932 4.258698
H 3.645532 0.432735 4.124596
H 1.933743 0.301861 5.909754
H 0.679668 0.802408 4.778927
H 3.087228 1.293867 1.888080
H 1.380400 1.423074 2.317907
C 2.366526 0.183571 -2.189181
H 2.404776 -0.844537 -2.580266
H 1.331902 0.529749 -2.309959
C 3.308969 1.079874 -2.983500
H 2.664332 1.204687 -0.337487
C 4.201881 -0.277339 -0.535159
C 2.703552 -3.142106 0.219839
C 0.435206 -3.204780 -0.661119
H 4.481814 -0.299307 0.526615
H 4.313743 -1.298367 -0.923050
C 5.134555 0.646033 -1.311848
H 5.072456 1.660355 -0.890235
H 6.172303 0.315331 -1.181966
C 4.765129 0.682726 -2.787968
H 4.929137 -0.314050 -3.224046
H 5.423652 1.372612 -3.330124
H 3.166002 2.120226 -2.652925
H 3.039273 1.049135 -4.046447
H 3.516443 -2.600934 0.696494
C 2.881353 -4.489094 -0.054864
H 3.821278 -4.975396 0.195424
C 1.844544 -5.199086 -0.645069
H 1.961519 -6.256614 -0.872775
C 0.645426 -4.561498 -0.930245
H -0.159911 -5.140809 -1.372411
C -0.899224 -2.583824 -0.996015
H -1.090421 -1.798642 -0.246348
C -0.875821 -1.904005 -2.369218
C -2.093268 -3.533043 -0.907678
H -0.029392 -1.208121 -2.422481
H -0.692062 -2.673322 -3.134114
C -2.180870 -1.175264 -2.657632
H -2.278049 -0.332996 -1.954256
H -2.151462 -0.734836 -3.662518
C -3.387421 -2.089963 -2.504661
H -3.362516 -2.856457 -3.294175
H -4.315588 -1.522039 -2.649531
C -3.391080 -2.773729 -1.144723
H -3.515272 -2.016658 -0.356784
H -4.247523 -3.454330 -1.059840
H -2.005721 -4.320719 -1.669556
H -2.104798 -4.034424 0.068967
C -2.450289 1.620705 0.196599
C -1.621493 2.470780 -0.159246
C -3.592024 0.805114 0.495436
C -4.657784 0.752175 -0.413919
C -3.646065 0.058043 1.681184

C -5.764581 -0.035861 -0.131127
H -4.607076 1.326307 -1.335797
C -4.758814 -0.724583 1.953021
H -2.812865 0.097821 2.379990
C -5.816876 -0.773180 1.048854
H -6.590368 -0.076967 -0.837784
H -4.799680 -1.302440 2.873467
H -6.685635 -1.391700 1.263587
C -0.827495 3.622124 -0.480817
C 0.044545 3.605748 -1.579772
C -0.918797 4.768535 0.320706
C 0.811694 4.724081 -1.870129
H 0.111104 2.714104 -2.199082
C -0.147144 5.882011 0.018968
H -1.593005 4.776227 1.173471
C 0.718179 5.861350 -1.071664
H 1.486035 4.707412 -2.723423
H -0.220365 6.771309 0.640857
H 1.322540 6.736383 -1.300915

$^{Ph}Y_S$-PCy$_2$-AuCl (A):

Au -2.326005 -0.395500 -0.859723
Cl -3.700979 -1.892129 -2.058466
P -0.979391 1.081911 0.266209
C 0.597302 0.296348 0.540376
C -1.805842 1.536401 1.849627
C -0.715146 2.645292 -0.713081
S 1.675312 0.916332 1.729579
P 0.829081 -1.345955 0.002752
C -1.734750 0.369487 2.828586
H -1.246825 2.382962 2.266029
C -3.256673 1.945533 1.592144
C -1.739179 2.802417 -1.839705
H 0.267383 2.473606 -1.178687
C -0.611125 3.922235 0.121969
O 1.134868 2.213908 2.167874
C 3.247047 1.233258 0.954655
O 1.957904 -0.078020 2.783626
C 0.529932 -1.531114 -1.774214
C 2.578001 -1.818107 0.158456
C -0.211626 -2.521648 0.912851
C -2.465389 0.688914 4.125128
H -2.182746 -0.520504 2.356028
H -0.687478 0.124295 3.035810
C -3.965994 2.282626 2.897566
H -3.308944 2.794031 0.897822
H -3.776756 1.109080 1.099292
C -1.421498 4.014778 -2.705277
H -2.744787 2.906516 -1.404349
H -1.765786 1.891796 -2.453614
C -0.284569 5.118958 -0.762539
H -1.569788 4.104142 0.628368
H 0.145058 3.794997 0.901372
C 4.407559 0.815033 1.590441

C 3.298232 1.918496 -0.253786
C 0.545102 -2.807876 -2.341623
C 0.395073 -0.408365 -2.593368
C 3.440134 -1.400444 -0.862945
C 3.082105 -2.551469 1.231899
C -0.074304 -2.538261 2.306675
C -1.213627 -3.284226 0.306664
C -3.904645 1.114228 3.871326
H -2.431285 -0.182715 4.791700
H -1.932686 1.500582 4.643086
H -5.007761 2.560187 2.692042
H -3.489270 3.163945 3.352643
C -1.306376 5.286397 -1.877303
H -0.468823 3.836770 -3.228091
H -2.187683 4.125672 -3.483196
H -0.228575 6.027164 -0.148860
H 0.715080 4.978382 -1.202235
H 4.336525 0.268288 2.527115
C 5.634611 1.077454 0.994519
C 4.530069 2.162953 -0.841247
H 2.378370 2.235639 -0.740697
H 0.701794 -3.686530 -1.718755
C 0.361566 -2.958052 -3.710330
C 0.220457 -0.563811 -3.962872
H 0.431774 0.585294 -2.154668
H 3.055721 -0.828897 -1.704879
C 4.790860 -1.710838 -0.805437
H 2.427529 -2.888184 2.029108
C 4.437623 -2.857858 1.283857
H 0.655616 -1.887307 2.785640
C -0.904839 -3.341700 3.076500
C -2.051734 -4.072856 1.087665
H -1.378676 -3.241795 -0.765942
H -4.463401 0.265314 3.448334
H -4.399542 1.375430 4.815610
H -2.287143 5.519589 -1.435237
H -1.042607 6.137462 -2.518308
H 6.547521 0.742770 1.484857
C 5.717608 1.741325 -0.231390
H 4.573919 2.689390 -1.793368
H 0.357969 -3.954197 -4.146895
C 0.186596 -1.838472 -4.518840
H 0.105712 0.314595 -4.594044
C 5.291336 -2.440008 0.269463
H 5.454018 -1.378371 -1.601191
H 4.824533 -3.431060 2.123450
H -0.790732 -3.351099 4.158413
C -1.893740 -4.110394 2.468447
H -2.839800 -4.649013 0.608033
C 7.040932 1.960726 -0.898216
H 0.037323 -1.960453 -5.589548
H 6.350801 -2.683972 0.315455
H -2.552778 -4.728421 3.074959
H 7.287320 1.111022 -1.550418
H 7.849399 2.053107 -0.165471

H 7.032515 2.859239 -1.524177

$^{Ph}Y_S$-PCy$_2$-Au(pa)$^+$:

Au 1.762785 0.241433 1.083558
P 0.259442 1.362681 -0.287586
C -1.070854 0.224036 -0.570469
C 1.158218 1.860767 -1.817892
C -0.362009 2.920727 0.521885
S -2.133277 0.523631 -1.894393
P -1.074058 -1.343020 0.191902
C 1.363299 0.681810 -2.759650
H 0.512575 2.596313 -2.313063
C 2.493759 2.506852 -1.445855
C 0.490962 3.314537 1.730752
H -1.357476 2.627498 0.887793
C -0.557085 4.105720 -0.425760
O -1.806301 1.879866 -2.367874
C -3.802808 0.548199 -1.284922
O -2.085344 -0.566459 -2.885965
C -0.990727 -1.225883 1.999750
C -2.648205 -2.192520 -0.104500
C 0.273244 -2.406343 -0.409855
C 2.150572 1.098652 -3.995377
H 1.905101 -0.112877 -2.224305
H 0.392668 0.267199 -3.051430
C 3.254935 2.937113 -2.692675
H 2.345152 3.360831 -0.772821
H 3.098293 1.772580 -0.888780
C -0.102520 4.515514 2.455191
H 1.511231 3.549385 1.391089
H 0.587971 2.464383 2.420693
C -1.161500 5.290311 0.318774
H 0.415228 4.405830 -0.841182
H -1.190700 3.806370 -1.265542
C -4.782702 -0.145573 -1.982139
C -4.113570 1.282800 -0.146625
C -0.945928 -2.393087 2.769819
C -1.064451 0.015779 2.634340
C -3.702292 -1.938613 0.781466
C -2.836979 -3.056535 -1.183469
C 0.421504 -2.469534 -1.800052
C 1.136289 -3.137805 0.412256
C 3.472615 1.758374 -3.630008
H 2.318064 0.224010 -4.636859
H 1.542202 1.803269 -4.581405
H 4.214069 3.384779 -2.403522
H 2.684862 3.721150 -3.212765
C -0.310931 5.692834 1.514059
H -1.070738 4.223668 2.890366
H 0.545193 4.798219 3.294615
H -1.282417 6.134093 -0.371987
H -2.171958 5.021977 0.662980
H -4.508736 -0.719829 -2.863347
C -6.090702 -0.109619 -1.518027

C -5.422875 1.298651 0.310086
H -3.333189 1.818423 0.389798
H -0.964753 -3.370544 2.292407
C -0.901800 -2.307184 4.155733
C -1.024426 0.097251 4.021648
H -1.174856 0.916822 2.039627
H -3.564314 -1.262181 1.621893
C -4.933016 -2.548098 0.586577
H -2.028503 -3.259678 -1.879119
C -4.074302 -3.661355 -1.372298
H -0.233183 -1.875968 -2.437607
C 1.400733 -3.283416 -2.358421
C 2.115650 -3.945360 -0.154083
H 1.071890 -3.065261 1.493530
H 4.121762 1.021146 -3.133122
H 4.001473 2.081168 -4.535595
H 0.667741 6.048803 1.157821
H -0.772164 6.531856 2.049999
H -6.861628 -0.658849 -2.056236
C -6.431042 0.598941 -0.362878
H -5.669900 1.863711 1.207265
H -0.859910 -3.217357 4.749594
C -0.925991 -1.063170 4.782146
H -1.078518 1.070228 4.505182
C -5.119413 -3.410244 -0.490188
H -5.749565 -2.344196 1.275776
H -4.218096 -4.335279 -2.213718
H 1.505452 -3.331967 -3.440050
C 2.242635 -4.027551 -1.537478
H 2.788452 -4.504562 0.492306
C -7.833491 0.576616 0.162154
H -0.891221 -1.001385 5.867685
H -6.084968 -3.888149 -0.642490
H 3.010773 -4.660889 -1.976088
H -8.005617 -0.332131 0.755764
H -8.565837 0.573130 -0.652287
H -8.035466 1.435636 0.809908
C 2.899249 -0.860104 2.673009
C 3.657229 -0.930470 1.701529
C 4.649314 -1.073424 0.679623
C 6.006606 -1.011930 1.025940
C 4.274552 -1.277009 -0.656235
C 6.971931 -1.158855 0.040306
H 6.292652 -0.849983 2.062004
C 5.249208 -1.418826 -1.631885
H 3.219910 -1.327560 -0.916958
C 6.596692 -1.360162 -1.285760
H 8.024899 -1.113170 0.308373
H 4.953698 -1.577167 -2.666668
H 7.359184 -1.471476 -2.053633
H 2.390562 -0.972321 3.616013

$^{Ph}Y_S$-PCy$_2$-Au(dpa)$^+$:

Au -1.737723 0.323215 0.456612
P 0.149502 -0.480947 1.544500
C 1.390123 -0.685331 0.293864
C -0.341662 -2.053446 2.374741
C 0.746703 0.684737 2.869618
S 2.709023 -1.748780 0.616269
P 1.100045 -0.160720 -1.343942
C -0.503146 -3.182314 1.363639
H 0.484608 -2.304664 3.050171
C -1.622395 -1.843955 3.185030
C -0.297743 1.746376 3.220430
H 1.592585 1.190939 2.380952
C 1.295527 0.002374 4.124487
O 2.626658 -2.070668 2.051721
C 4.229628 -0.867244 0.352026
O 2.731588 -2.882668 -0.325589
C 0.720707 1.607583 -1.466738
C 2.609202 -0.328788 -2.336619
C -0.218326 -1.111601 -2.151559
C -0.960116 -4.467323 2.040860
H -1.238159 -2.884562 0.598557
H 0.446149 -3.348477 0.843700
C -2.055071 -3.137259 3.863712
H -1.492213 -1.048706 3.929378
H -2.423207 -1.499897 2.511032
C 0.257140 2.759444 4.213002
H -1.188177 1.256857 3.643257
H -0.633284 2.260303 2.309295
C 1.854102 1.038192 5.093001
H 0.489813 -0.553194 4.624127
H 2.062653 -0.724808 3.842623
C 5.251957 -1.490568 -0.348872
C 4.392019 0.409250 0.879787
C 0.534533 2.176041 -2.732773
C 0.730032 2.428323 -0.339213
C 3.513728 0.739605 -2.344399
C 2.890469 -1.486394 -3.062627
C -0.165170 -2.500041 -1.986540
C -1.241808 -0.540030 -2.912628
C -2.231803 -4.257666 2.849370
H -1.105407 -5.249634 1.285092
H -0.160282 -4.821292 2.707758
H -2.984467 -2.967797 4.421702
H -1.293454 -3.429190 4.601700
C 0.810363 2.082800 5.458644
H 1.061016 3.330477 3.723279
H -0.523918 3.483623 4.477427
H 2.228323 0.533723 5.992553
H 2.721241 1.533816 4.630449
H 5.092026 -2.483737 -0.760357
C 6.451634 -0.814054 -0.531581
C 5.590717 1.074741 0.678057
H 3.576526 0.885109 1.420523
H 0.589637 1.558512 -3.627461
C 0.313631 3.541093 -2.853667

C 0.505544 3.794816 -0.464412
H 0.937843 2.001386 0.635616
H 3.303228 1.642834 -1.776427
C 4.689007 0.646679 -3.075440
H 2.198117 -2.323010 -3.058894
C 4.071075 -1.571418 -3.791873
H 0.625902 -2.937677 -1.378808
C -1.119148 -3.307682 -2.593632
C -2.184552 -1.357327 -3.524657
H -1.325949 0.538281 -3.013731
H -3.058464 -4.000431 2.168372
H -2.524150 -5.187547 3.352847
H -0.013550 1.595733 6.002048
H 1.235617 2.829741 6.140676
H 7.254861 -1.294652 -1.087668
C 6.637992 0.477455 -0.034940
H 5.719363 2.078836 1.078930
H 0.168490 3.977084 -3.839455
C 0.290595 4.350055 -1.719888
H 0.505033 4.423565 0.423130
C 4.967545 -0.508627 -3.800364
H 5.391011 1.477667 -3.074646
H 4.287128 -2.474389 -4.358307
H -1.074502 -4.386170 -2.458753
C -2.126008 -2.738162 -3.367197
H -2.980402 -0.909203 -4.114403
C 7.911020 1.224363 -0.288901
H 0.115188 5.418784 -1.818218
H 5.889245 -0.580714 -4.374158
H -2.877540 -3.369706 -3.836431
H 7.795194 1.898584 -1.148948
H 8.737861 0.543489 -0.514394
H 8.192078 1.842620 0.570576
C -3.391394 1.587736 -0.496881
C -3.727970 0.404467 -0.646139
C -4.355509 -0.830885 -1.010829
C -5.212199 -0.844206 -2.121696
C -4.106493 -2.020231 -0.314217
C -5.792001 -2.035649 -2.531526
H -5.405564 0.078734 -2.662783
C -4.690055 -3.206535 -0.733651
H -3.453333 -2.007576 0.554295
C -5.527954 -3.218528 -1.845007
H -6.452213 -2.042154 -3.396005
H -4.487318 -4.127090 -0.190670
H -5.980503 -4.151078 -2.175138
C -3.179354 3.002362 -0.443713
C -3.275228 3.754928 -1.622582
C -2.893433 3.641480 0.771986
C -3.096985 5.130414 -1.576702
H -3.490833 3.255743 -2.564018
C -2.706357 5.015092 0.802285
H -2.826382 3.052423 1.683998
C -2.808820 5.761007 -0.369548
H -3.176801 5.713419 -2.491492

H -2.484609 5.507325 1.746623
H -2.665230 6.839028 -0.340763

$^{Ph}Y_S$-PCy$_2$-Au(an)$^+$:

Au 1.864786 -0.135279 0.963445
P 0.424519 1.229745 -0.181256
C -1.031820 0.272891 -0.520242
C 1.348675 1.737062 -1.697053
C -0.021525 2.783007 0.740832
S -2.031476 0.729026 -1.850959
P -1.236067 -1.304780 0.189481
C 1.470996 0.590072 -2.692905
H 0.753939 2.535559 -2.155658
C 2.730171 2.276143 -1.319920
C 0.921306 3.049767 1.916651
H -1.014818 2.544434 1.150112
C -0.184132 4.025994 -0.136370
O -1.557762 2.054514 -2.284217
C -3.697087 0.921567 -1.261898
O -2.084035 -0.330738 -2.874755
C -1.205504 -1.299981 2.002010
C -2.879129 -1.969662 -0.194191
C 0.006491 -2.492877 -0.403364
C 2.251974 1.023204 -3.927472
H 1.984308 -0.255326 -2.205488
H 0.473646 0.242126 -2.982300
C 3.489212 2.726717 -2.561412
H 2.650310 3.101599 -0.601546
H 3.301927 1.477303 -0.819662
C 0.453053 4.247605 2.732816
H 1.936263 3.233963 1.534052
H 0.992779 2.156604 2.552821
C -0.661617 5.207419 0.699640
H 0.782343 4.279449 -0.594511
H -0.881933 3.814948 -0.952205
C -4.738872 0.376706 -2.000007
C -3.938265 1.642957 -0.098465
C -1.465140 -2.496631 2.680813
C -1.023417 -0.123483 2.727503
C -3.936145 -1.651829 0.667309
C -3.110386 -2.761021 -1.319515
C 0.244328 -2.500027 -1.780979
C 0.715446 -3.364477 0.429206
C 3.616958 1.589477 -3.564249
H 2.356208 0.173333 -4.614159
H 1.670571 1.788090 -4.463277
H 4.479676 3.100595 -2.272562
H 2.956269 3.569763 -3.025747
C 0.276907 5.485070 1.864339
H -0.507895 3.998840 3.209124
H 1.163381 4.442383 3.546246
H -0.757625 6.094242 0.060754

H -1.669789 4.991129 1.085291
H -4.518035 -0.193015 -2.899010
C -6.041404 0.548183 -1.550934
C -5.244360 1.794378 0.342403
H -3.108480 2.060753 0.468153
H -1.667698 -3.411433 2.126211
C -1.491881 -2.516071 4.069097
C -1.054638 -0.145844 4.117603
H -0.876938 0.815541 2.206446
H -3.764552 -1.030500 1.543230
C -5.212205 -2.126836 0.402053
H -2.299280 -3.009609 -1.997304
C -4.392783 -3.230463 -1.579288
H -0.297422 -1.805032 -2.421666
C 1.168663 -3.386690 -2.322033
C 1.639654 -4.246758 -0.119584
H 0.562947 -3.352540 1.505182
H 4.234006 0.792458 -3.122741
H 4.141881 1.930150 -4.465706
H 1.257767 5.793475 1.471593
H -0.095140 6.323213 2.466978
H -6.862346 0.115200 -2.120320
C -6.315749 1.244752 -0.371126
H -5.439169 2.348657 1.258971
H -1.691867 -3.449338 4.590536
C -1.278129 -1.342832 4.788794
H -0.911652 0.778313 4.673354
C -5.440894 -2.916757 -0.721163
H -6.030725 -1.874591 1.072678
H -4.569766 -3.847854 -2.456979
H 1.346025 -3.389728 -3.395203
C 1.862934 -4.263259 -1.494088
H 2.187216 -4.925788 0.531026
C -7.719770 1.365160 0.136322
H -1.303303 -1.360676 5.876261
H -6.442036 -3.289085 -0.928541
H 2.586380 -4.956024 -1.918633
H -7.832972 2.219733 0.811024
H -8.002941 0.463006 0.696461
H -8.435250 1.471795 -0.686100
H 3.741763 -1.193572 2.636839
H 3.171662 -2.372287 1.650762
C 4.586163 -1.184924 0.774104
C 4.479382 -1.625374 -0.543999
C 5.706196 -0.477446 1.192926
C 5.508766 -1.364474 -1.438640
H 3.584208 -2.155426 -0.866514
C 6.727867 -0.211396 0.285596
H 5.777926 -0.129565 2.222165
C 6.634323 -0.653608 -1.029130
H 5.423747 -1.711209 -2.466315
H 7.603173 0.344364 0.614845
H 7.434695 -0.443672 -1.734835
N 3.484573 -1.402748 1.675073

5. References

[1] H. Darmandeh, T. Scherpf, K.-S. Feichtner, C. Schwarz, V. H. Gessner, *Z. Anorg. Allg. Chem.* **2020**, *646*, 835.

[2] I. Rodstein, D. S. Prendes, L. Wickert, M. Paaßen, V. H. Gessner, *J. Org. Chem.* **2020**, *85*, 14674.

[3] a) T. Scherpf, C. Schwarz, L. T. Scharf, J.-A. Zur, A. Helbig, V. H. Gessner, *Angew. Chem.* **2018**, *130*, 13041, b) H. Riihimäki, T. Kangas, P. Suomalainen, H. K. Reinius, S. Jääskeläinen, M. Haukka, A.O.I. Krause, T.A. Pakkanen, J.T. Pursiainen, *J. Mol. Cat. A. Chem.*, **2003**, *1*, 81-94.

[4] L. Chen, P. Ren, B. P. Carrow, *J. Am. Chem. Soc.* **2016**, *138*, 6392.

[5] J. Handelmann, C. N. Babu, H. Steinert, C. Schwarz, T. Scherpf, A. Kroll, V. H. Gessner, *Chem. Sci.* **2021**, *12*, 4329.

[6] R. Dennington, T. A. Keith, J. M. Millam, *GaussView, Version 6.0*, Semichem Inc., Shawnee Mission, **2016**.

[7] M. J. Frisch, G. W. Trucks, H. B. Schlegel, G. E. Scuseria, M. A. Robb, J. R. Cheeseman, G. Scalmani, V. Barone, G. A. Petersson, H. Nakatsuji et al., *Gaussian 16, Revision B.01*, Gaussian, Inc., Wallingford CT, **2016**.

[8] M. J. Frisch, G. W. Trucks, H. B. Schlegel, G. E. Scuseria, M. A. Robb, J. R. Cheeseman, G. Scalmani, V. Barone, G. A. Petersson, H. Nakatsuji et al., *Gaussian 16, Revision C.01*, Gaussian, Inc., Wallingford CT, **2016**.

[9] a) P. Hohenberg, W. Kohn, *Phys. Rev.* **1964**, *136*, B864-B871; b) W. Kohn, L. J. Sham, *Phys. Rev.* **1965**, *140*, A1133-A1138.

[10] Y. Zhao, D. G. Truhlar, *J. Phys. Chem. A* **2005**, *109*, 5656.

[11] F. Weigend, R. Ahlrichs, *Phys. Chem. Chem. Phys.* **2005**, *7*, 3297.

[12] A. Bergner, M. Dolg, W. Küchle, H. Stoll, H. Preuß, *Molecular Physics* **1993**, *80*, 1431.

[13] a) S. Grimme, J. Antony, S. Ehrlich, H. Krieg, *J. Chem. Phys.* **2010**, *132*, 154104; b) S. Grimme, S. Ehrlich, L. Goerigk, *J. Comput. Chem.* **2011**, *32*, 1456; c) D. G. A. Smith, L. A. Burns, K. Patkowski, C. D. Sherrill, *J. Phys. Chem. Lett.* **2016**, *7*, 2197.

[14] Jacopo Tomasi, Benedetta Mennucci, and Roberto Cammi, *Chemical Reviews* **2005,** *105* (8), 2999-3094

[15] NBO 7.0. E. D. Glendening, J. K. Badenhoop, A. E. Reed, J. E. Carpenter, J. A. Bohmann, C. M. Morales, P. Karafiloglou, C. R. Landis, and F. Weinhold, Theoretical Chemistry Institute, University of Wisconsin, Madison, WI (2018)

[16] a) Bader, R. F. W., A quantum theory of molecular structure and its applications. Chem. Rev. **1991**, *91*, 893-928. b) Kumar, P. S. V.; Raghavendra, V.; Subramanian, V., Bader's Theory of Atoms in Molecules (AIM) and its Applications to Chemical Bonding. J. Chem. Sci. 2016, 128, 1527-1536.

[17] Johnson, E. R.; Keinan, S.; Mori-Sánchez, P.; Contreras-García, J.; Cohen, A. J.; Yang, W., Revealing Noncovalent Interactions. J. Am. Chem. Soc. 2010, 132, 6498-6506.

[18] Lu, T.; Chen, F., Multiwfn: A multifunctional wavefunction analyzer. J. Comput. Chem. 2012, 33, 580-592.

[19] Humphrey, W., Dalke, A. and Schulten, K., "VMD - Visual Molecular Dynamics", J. Molec. Graphics, 1996, vol. 14, pp. 33-38.
